# CO_2_ hydrosilylation catalyzed by an N-heterocyclic carbene (NHC)-stabilized stannyliumylidene†

**DOI:** 10.1039/d4sc07116f

**Published:** 2025-01-22

**Authors:** Dechuang Niu, Arseni Kostenko, John A. Kelly, Debotra Sarkar, Huihui Xu, Shigeyoshi Inoue

**Affiliations:** a TUM School of Natural Sciences, Department of Chemistry, Institute of Silicon Chemistry and Catalysis Research Center, Technische Universität München Lichtenbergstraße 4 85748 Garching bei München Germany s.inoue@tum.de

## Abstract

The di-N-heterocyclic carbene (NHCs) stabilized stannyliumylidene, [^Mes^TerSn(IMe_4_)_2_][BArF], (^Mes^Ter = 2,6-Mes_2_C_6_H_3_, Mes = 2,4,6-Me_3_-C_6_H_2_, IMe_4_ = 1,3,4,5-tetramethylimidazol-2-ylidene, BArF = (3,5-(CF_3_)_2_-C_6_H_5_)_4_B), was isolated from the reaction of (^Mes^Ter)SnCl with two equivalents of IMe_4_, followed by one equivalent of Na[BArF]. This stannyliumylidene acts as a precatalyst for the homogeneous hydrosilylation of CO_2_. Experimental mechanistic studies and quantum chemical calculations have been conducted to elucidate the catalytically active species and the mechanism for the transformation, revealing the stannyliumylidene [^Mes^TerSn(CO_2_IMe_4_)_2_][BArF], which is formed in the presence of CO_2_, as the catalytically active species.

## Abstract 2

The di-NHCs-stabilized stannyliumylidene serves as an efficient pre-catalyst for the selective hydrosilylation of CO_2_ to silyl formate. Experimental mechanistic studies and quantum chemical calculations revealed the mechanism.

## Graphical abstract

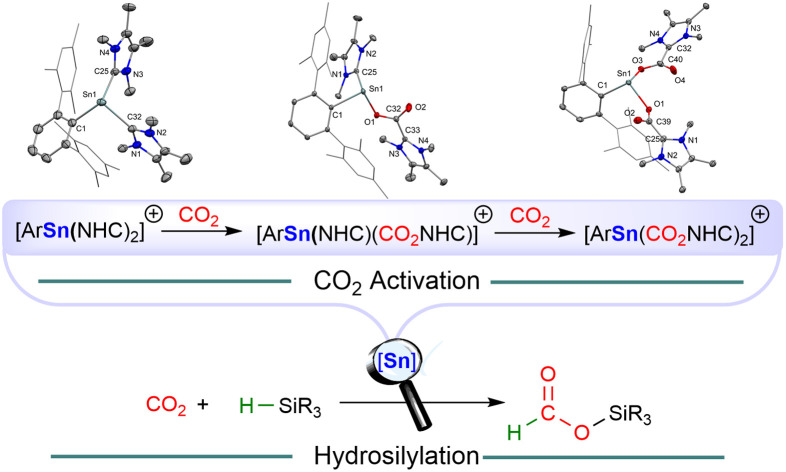

## Introduction

A major goal in contemporary chemical research is the utilization of carbon dioxide in the creation of fine chemicals, due to its high production/abundance and its status as a greenhouse gas. Recent advances in main group chemistry have seen the successful isolation of highly reactive compounds with unprecedented oxidation states and bonding modes, which in turn have demonstrated the remarkable capability of activating CO_2_.^1–5^ For example, silylenes can react with CO_2_ yielding the corresponding silicon^IV^ carbonates [C(=O)(O–)_2_]^2−^.^6,7^ Germylene and stannylene species tend to afford E^II^ (E = Ge and Sn) carboxylate compounds, owing to the weak nucleophilicity of their lone pair of electrons.^8,9^

**Fig. 1:**
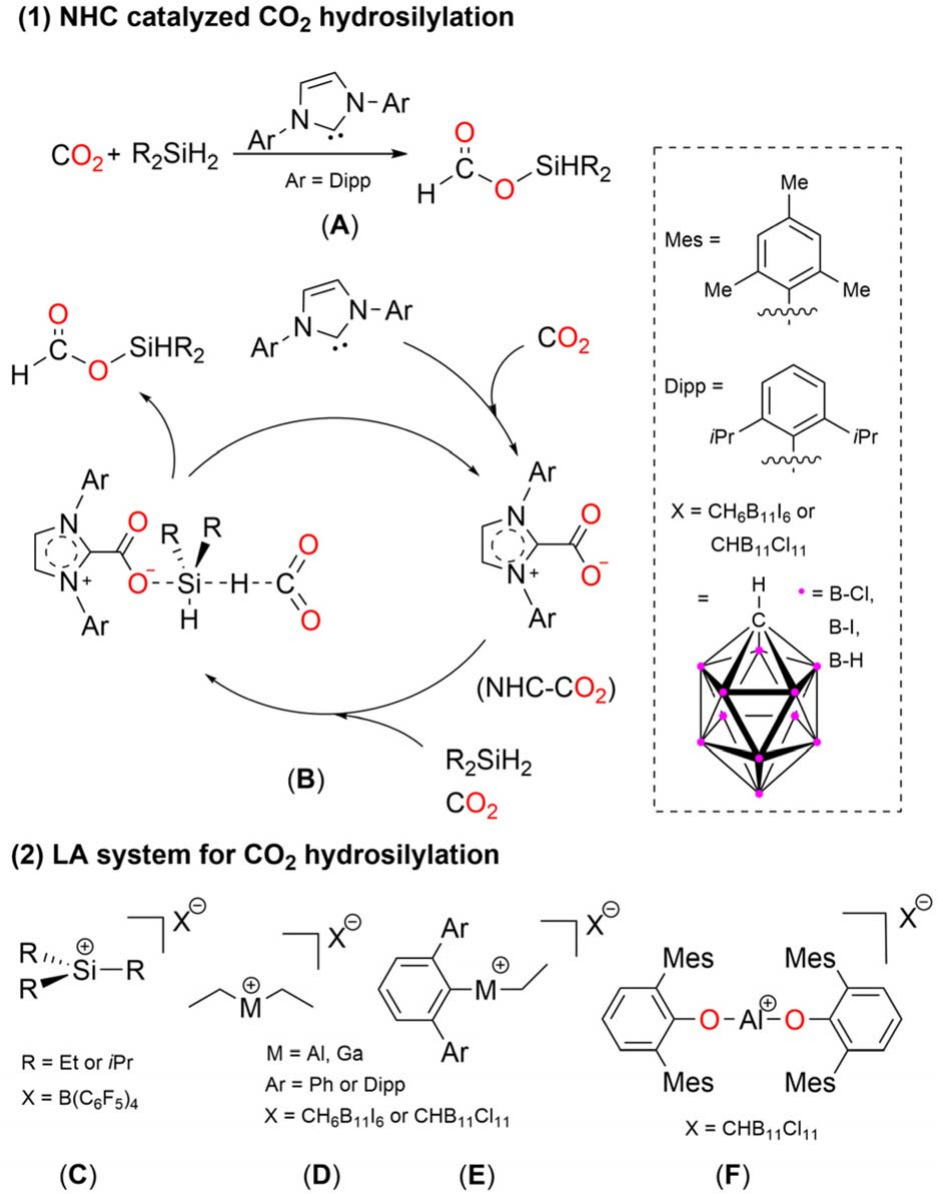
(1) Proposed mechanism for NHC-catalyzed hydrosilylation of CO_2_; (2) LA systems effective in CO_2_ hydrosilylation.

While main group-mediated CO_2_ activation is becoming more commonplace, the catalytic transformation of carbon dioxide is still rare, especially when it comes to hydrosilylation reactions. Main-group catalysis has attracted tremendous attention recently, due to the higher abundance and lower toxicity of such elements. Currently, the most potent catalysts for the hydrosilylation of CO_2_ using main group compounds are: Lewis bases (LBs), Lewis acids (LAs), and frustrated Lewis pairs (FLPs).^1^ The use of N-heterocyclic carbenes (NHCs) as catalysts for the hydrosilylation of CO_2_ was first reported by Zhang, Ying and coworkers (Fig. 1, A).^10^ Later, experimental and theoretical studies showed that the NHC–CO_2_ adduct acts as the active catalytic species in the hydrosilylation of CO_2_, wherein the Si–H bond is activated *via* coordination of the oxygen atom of the NHC–CO_2_ adduct (Fig. 1, B).^11,12^

Main-group Lewis acids have been shown to be capable of the catalytic hydrosilylation of CO_2_, examples include the silylium cation, C^13^ and two-coordinate group 13 cations D, E, and F (Fig. 1(2)).^14–16^ Okuda and coworkers found BPh_3_ could catalyze the hydrosilylation of CO_2_ to form silyl formate, but only in highly polar, aprotic solvents, such as acetonitrile.^17^ They posit the mechanism involves weak/dynamic coordination of BPh_3_ to CO_2_, as well as to the Si–H moiety, generating two partially polarized transient species.

**Fig. 2:**
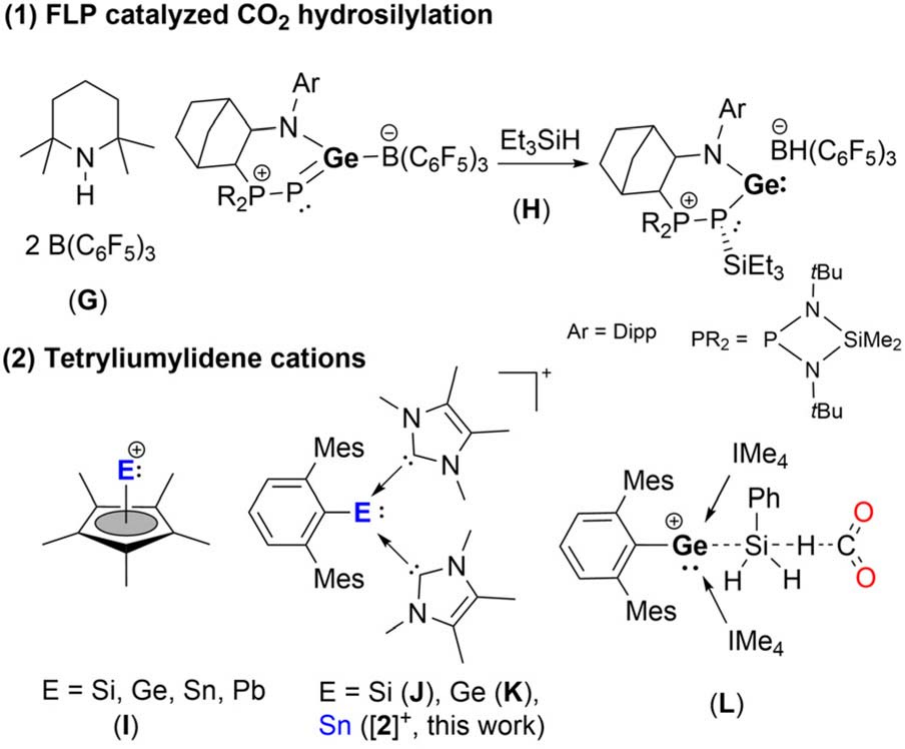
(1) FLP systems; (2) heavier tetryliumylidene cations and K catalyzed CO_2_.

Another pertinent contribution to main-group based catalysis was reported in 2009 by the Piers group, in which amine/borane FLP (Fig. 2, G) was effective in the hydrosilylation of CO_2_.^18^ Since then, a multitude of various FLP catalytic systems have been reported, such as {[Tism^Pribenz^]M} [HB(C_6_F_5_)_3_](M = Zn, Mg; Tism^Pribenz^ = tri[(1-isopropylbenzimidazol-2-yl)-dimethylsilyl]methyl)^19,20^ and [(^Dipp^nacnac)Ga(Ad)][HB(C_6_F_5_)_3_](^Dipp^nacnac = [{N(Dipp)CMe}_2_CH]^−^, Dipp = 2,6-diisopropylphenyl, Ad = 1-adamantyl).^21^ A noteworthy example from the Kato group is an N,P-heterocyclic germylene/B(C_6_F_5_)_3_ Lewis pair that can be used as a catalyst for the selective CO_2_ hydrosilylation to H_2_C(OSiEt_3_)_2_, through Si–H bond activation to give the highly reactive cationic (amino)(phosphino) germylene with HB(C_6_F_5_)_3_^−^ as a counter anion, followed by activation of CO_2_ (Fig. 2, H).^22^

An emerging class of compounds that is of high interest to main group chemists, from the point of view of their catalytic capabilities, are the tetryliumylidenes. Tetryliumylidenes (RE:^+^), are mono-substituted E^II^ (E = Si, Ge, Sn, Pb) atoms with a lone pair of electrons and two vacant orbitals, that exhibit high reactivity due to their unsaturated and amphiphilic nature.^4,23,24^ There has been a growing number of reports of these compounds ever since the first was reported by Jutzi and coworkers,^23,25^ whereby they used pentamethyl-cyclopentadienyl to stabilize the heavier tetryliumylidenes (Fig. 2, I, Cp*E:^+^, E = Si,^26^ Ge,^27^ Sn,^27^ Pb;^28^ Cp* = Me_5_C_5_). These were shown to be able to activate small molecules (*e.g.*, CO_2_, H_2_, N_2_O, alkenes, ketones, *etc.*).^24^ Silyliumylidene ions stabilized by Lewis bases (*e.g.*, N-heterocyclic carbene (NHC) (J),^29^ PMe_3_) could reduce CO_2_, resulting in the silaacylium ions, [RSi(O)(IMe_4_)_2_]Cl (R = ^Mes^Ter or Tipp; ^Mes^Ter = 2,6-Mes_2_-C_6_H_3_, Mes = 2,4,6-Me_3_-C_6_H_2_, Tipp = 2,4,6-iPr_3_-C_6_H_2_, IMe_4_ = 1,3,4,5-tetramethylimidazol-2-ylidene),^30,31^*via* the initial coordination of the C atom to the Si center. We reported on the CO_2_ insertion into the Si–C^NHC^ bond of the dicationic silicon^IV^ compound [^Mes^Ter(IMe_4_)_2_SiH][OTf]_2_ (OTf = CF_3_SO_3_^−^).^32^

Recently, our group has isolated the germyliumylidene ion (K, Fig. 2), which was shown to react with N_2_O to produce a germa-acylium ion [(^Mes^Ter)Ge(O)(IMe_4_)_2_]X (X = Cl or BArF, BArF = (3,5-(CF_3_)_2_C_6_H_5_)_4_B).^33^ This germa-acylium ion could then be utilized in the catalytic functionalization of CO_2_, forming the catalytically active germylene, (^Mes^Ter)Ge(OSiHPh_2_)(IMe_4_). It is also worth noting that K catalyzes the reduction of CO_2_ with silane *via* a three-component transition state (Fig. 2, L).^34^ The mechanism is analogous to the cooperative silane/CO_2_ mechanism for the hydrosilylation of CO_2_ catalyzed by NHC (Fig. 1, B).^12^

The heavier tetryliumylidene analogues, *i.e.* stannyliumylidene cations, are also an emerging class of low valent main group species that that may have the potential to be used in catalysis. However, examples of the utilization of stannyliumylidene in the activation of small molecules are rare. Herein, we present the first substantiated example of stannyliumylidene acting as a catalyst for CO_2_ hydrosilylation. We report the synthesis of the NHC-stabilized stannyliumylidene [2]^+^ – the tin analogue of J and K, which reacts with CO_2_ to form the catalytically active stannyliumylidene [4]^+^. We present the mechanism of conversion of [2]^+^ to the catalytically active stannyliumylidene species, isolating and fully describing the reactive intermediates of this process, as well as the catalytically active species. Additionally, we examine the catalytic performance of the stannyliumylidene species in the catalytic hydrosilylation CO_2_ using various silanes and conditions. Based on these studies and quantum chemical calculations we propose a mechanism for the catalytic reaction.

## Results and discussion

### Synthesis of stannyliumylidene

**Scheme 1:**
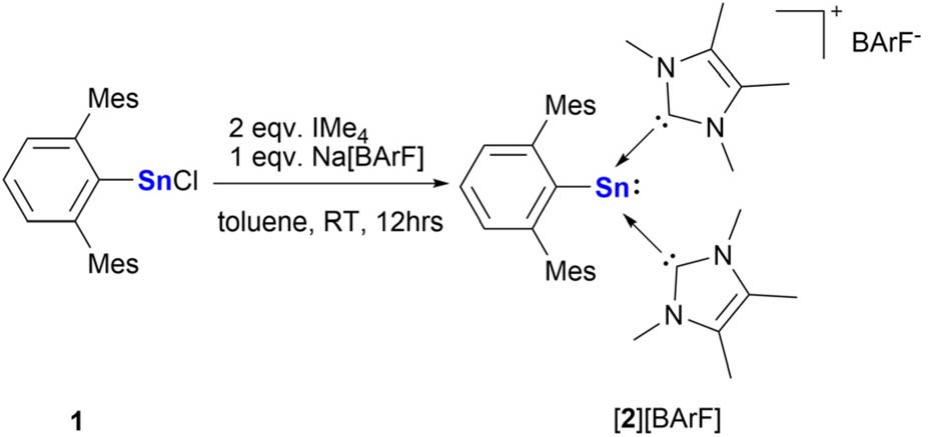
Synthesis of NHC-stabilized stannyliumylidene ion [2][BArF].

NHC-stabilized stannyliumylidene [^Mes^TerSn(IMe_4_)_2_][BArF] ([2][BArF]) was obtained by the reaction of chlorostannylene, ^Mes^TerSnCl (1) with two equivalents of IMe_4_ in toluene, followed by adding one equivalent of Na[BArF] in a one-pot procedure (Scheme 1). It was isolated as a colorless crystalline solid in a 72% yield. [2][BArF] has poor solubility in nonpolar organic solvents, *e.g.*, benzene and toluene, but it is soluble in fluorobenzene and THF. In acetonitrile, substitution occurs between the IMe_4_ and solvent molecules leading to a mixture of coordination compounds. This is in stark contrast to silyliumylidene (Fig. 2, J) and germyliumylidene ions (Fig. 2, K), where ligand substitution does not occur.^29,33^ In the ^119^Sn{^1^H} NMR spectrum, one sharp signal was observed at *δ* = −235.72 ppm in THF-d_8_, similar to the cationic stannylene, [^Tipp^TerSn(IMe_2_)_2_][HB(C_6_F_5_)_3_](^Tipp^Ter = 2,6-Tipp_2_-C_6_H_3_) (*δ* = −234 ppm), reported by the Wesemann group.^35^ The ^13^C{^1^H} NMR spectrum displays a resonance at *δ* = 170.18 ppm for the carbon of IMe_4_, which is downshifted compared to that of the germyliumylidene (K) (*δ* = 164 ppm) and the silyliumylidene (J) (*δ* = 160 ppm).^29,33^

**Fig. 3:**
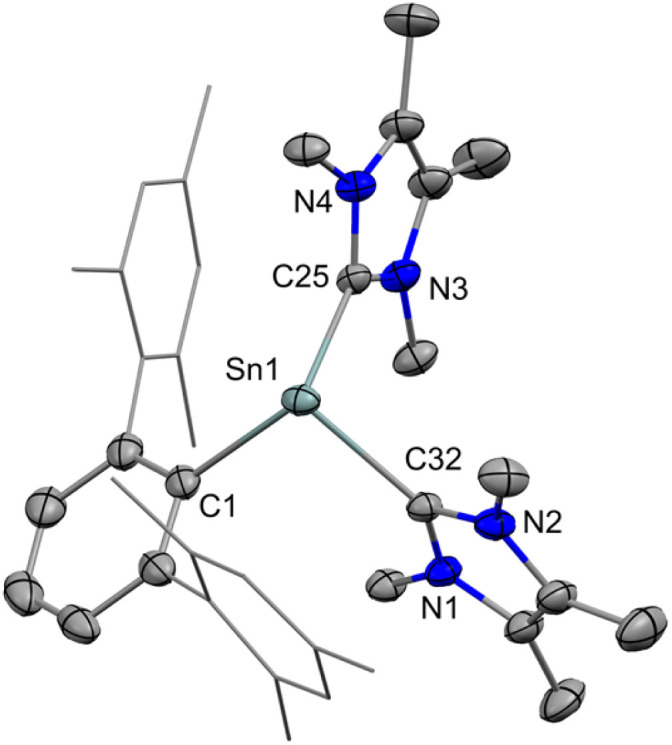
The molecular structure of [2]^+^. Ellipsoids are set at the 30% probability level; hydrogen atoms and [BArF]^−^ anion are omitted for clarity. Selected bond lengths [Å] and bond angles [°]: Sn1–C1 2.244(4), Sn1–C32 2.308(4), Sn1–C25 2.339(4), C1–Sn1–C25 107.7(1), C1–Sn1–C32 96.7(1), C25–Sn1–C32 96.0(1).

X-ray quality crystals of [2][BArF] were obtained from a toluene/fluorobenzene (C_6_H_5_F) solution stored at −35 °C. Single crystal X-ray diffraction (SC-XRD) analysis revealed that the Sn central is tricoordinate with two IMe_4_ moieties and one ^Mes^Ter-substituent (Fig. 3). The sum of the bond angles around the Sn1 atom is 300.4°. The Sn1–B1 distance is 8.587 Å, indicating no interaction between the cation and anion. Additionally, we found the shortest distance between the Sn atom and a fluorine atom at 4.032 Å. This also indicates there is no Sn–F interaction as the sum of the van der Waals radii of these atoms amounts to 3.56 Å (2.16 Å for Sn and 1.40 Å for F). The Sn–C^NHC^ bond distances are Sn1–C25 = 2.339(4) Å and Sn1–C32 = 2.308(4) Å. They are similar to that found in IPr_Me_SnCl_2_ (2.290(5) Å) (IPr_Me_ = 1,3-diisopropyl-4,5-dimethylimidazol-2-ylidene),^36^ IPr_Me_Sn[C(Cl)PMes*]_2_ (Mes* = 2,4,6-tri-*tert*-butylphenyl) (2.316(2) Å)^37^ and slightly longer than (^Dipp^Ter)Sn(IMe_4_)CH_2_P(CH_3_)_2_ = P(^Mes^Ter) (2.274(3) Å) (^Dipp^Ter = 2,6-Dipp_2_-C_6_H_3_, Dipp = 2,6-iPr_2_-C_6_H_3_).^38^ As expected, they are longer than the observed distances in the NHC-stabilized silyliumylidene ion (J) (1.948(19) Å, 1.966(19) Å) and NHC-stabilized germyliumylidene ion (K) (2.063(3) Å, 2.093(3) Å).

**Fig. 4:**
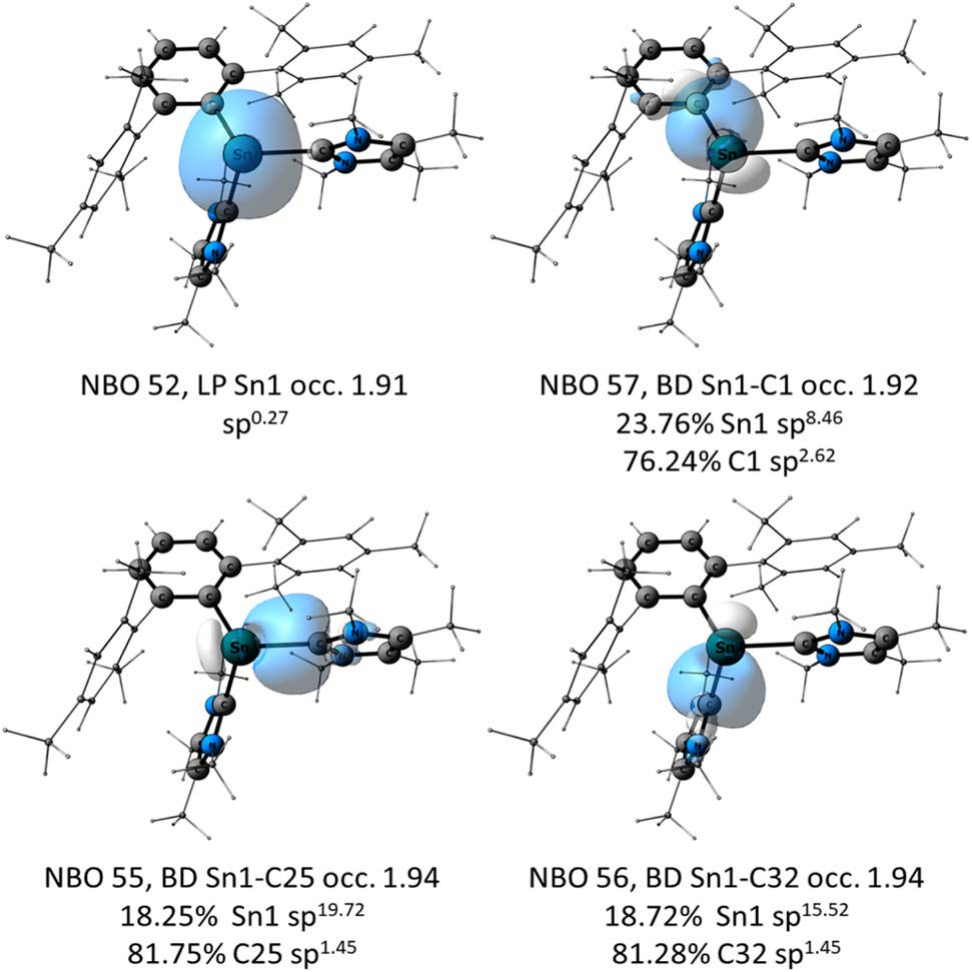
NBOs of [2]^+^ (iso = 0.04) at the PBE0/def2-TZVP//r^2^SCAN-3c level of theory. The mesityl substituents and the methyl substituents are shown as wireframes for clarity.

**Fig. 5:**
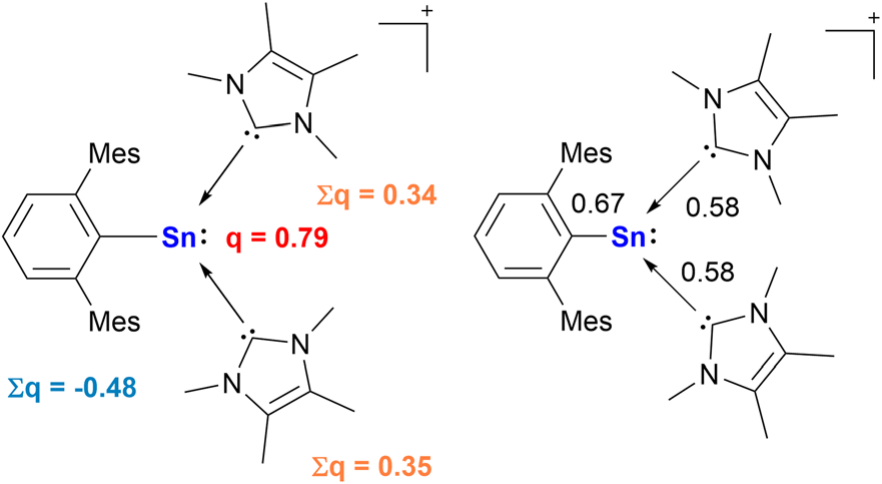
NPA charges and WBIs in [2]^+^.

To gain further insight into the electronic structure of [2]^+^, as well as to analyze the thermodynamics and kinetics in the processes involving this species, quantum chemical calculations were carried out. For details regarding the computational methods see the ESI.† Natural Bond Orbital (NBO) analysis of the optimized structure of [2]^+^ (Fig. 4) shows that the stannyliumylidene cation exhibits the expected bonding situation around the tin center with a σ-type lone pair of electrons at Sn (NBO 55), and a polarized Sn–C^Ar^ bond (NBO 57) with Wiberg Bond Index (WBI) of 0.67. The bonding between the Sn1 and C25 (of IMe_4_) where the WBI = 0.58, indicates a very polarized bonding interaction with electron density of 18.25% on Sn and 81.75% on C (NBO 55). This type of polarization, as well as the sp^1.45^ hybridization of the carbon, which interacts with the almost pure p orbital of Sn (sp^19.72^), can be interpreted as carbene coordination to the empty p-orbital of the Sn center. The situation is almost identical to that of the second carbene (Sn1–C32, NBO 56), in terms of bond polarization, hybridization of the interacting orbitals and the WBI (0.58) (Fig. 5). The Sn center of [2]^+^ is positively charged by 0.79 el. The aryl moiety of [2]^+^ withdraws substantial electron density being negatively charged with −0.48 el., while the Sn center is compensated by the donation from the NHC moieties with the sum of NPA charges of +0.34 and +0.35 el. In addition to the [2]^+^ representation as a bis(NHC)-stabilized stannyliumylidene – with dative bonds between the NHC ligands and the Sn atom – the NBO depiction may also hint at the stannyl anion character of the Sn center. In this case, the ionic representation of [2]^+^, where the negative charge is located on the Sn atom to which the two positively charged NHC moieties are bound covalently, should be considered. A detailed discussion of such stannyl-anion-type species in given in the ESI (Fig. S55 and S56†).

**Fig. 6:**
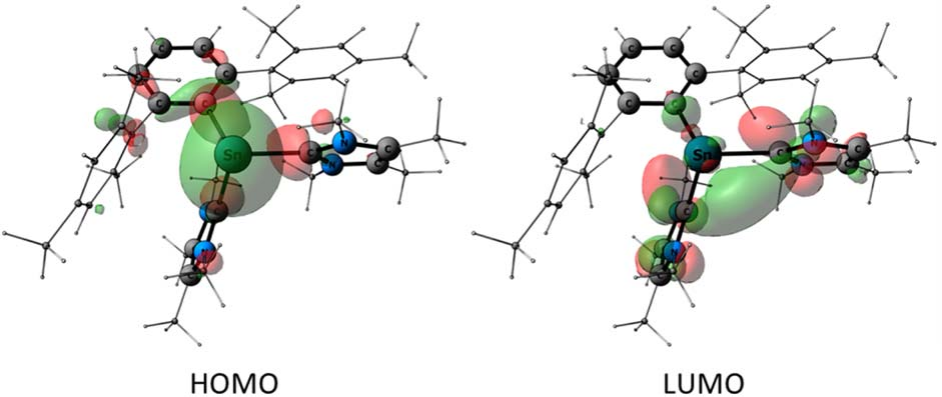
Frontier molecular orbitals of [2]^+^ (iso = 0.04) at the PBE0/def2-TZVP//r2SCAN-3c level of theory. The mesityl substituents and the methyl substituents are shown as wireframes for clarity.

The frontier orbitals of [2]^+^ are presented in Fig. 6. According to the NBO analysis of the canonical MOs, the HOMO has 52% non-bonding and 43% bonding character and consists mostly of the non-bonding lone pair of electrons at Sn (51%). Additional important contributions arise from the Sn–Ar and Sn–IMe_4_ bonding interactions (Sn–C1 (16%) and Sn–C25 (7%)). The LUMO is 73% anti-bonding and 19% non-bonding with the most significant contributions from π*(N3–C25) 25%, π*(N1–C32) 21%, σ*(Sn1–C1) 11%, σ*(Sn1–C32) 7%.

**Scheme 2:**
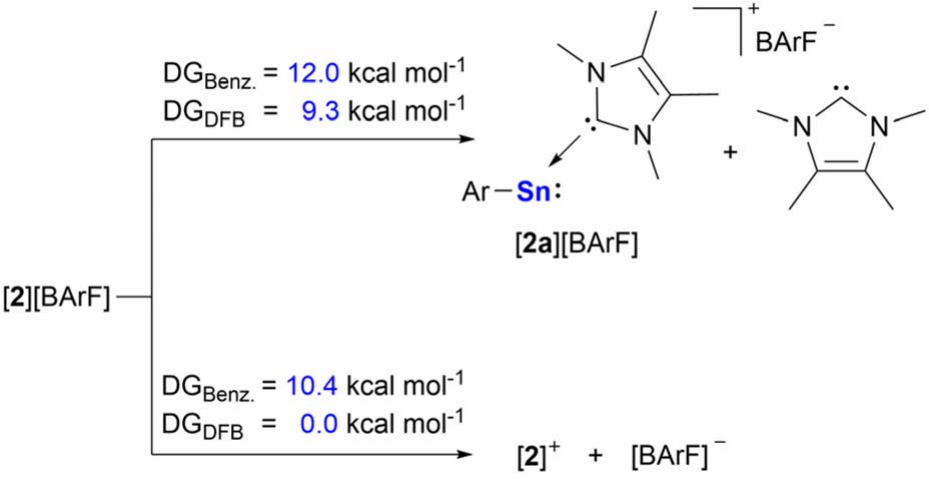
Calculated free energies for anion cation interaction and for NHC dissociation in [2][BArF].

Calculations at the PW6B95-D4/def2-QZVPP(CPCM)/r^2^SCAN-3c level of theory show that the anion–cation interaction in [2][BArF] is weak, and only 10.4 and 0.0 kcal mol^−1^ are required to fully separate the ions in benzene and difluorobenzene, respectively (Scheme 2). The charge transfer in [2][BArF] between [2]^+^ cation (Σ*q*_NPA_ = + 0.98 el.) and the [BArF]^−^ anion (Σ*q*_NPA_ = −0.98 el.) is negligible. The Sn–IMe_4_ bonding is also quite labile, with a dissociation free energy for [2][BArF] to form [2a][BArF] and free NHC of 12.0 and 9.3 kcal mol^−1^ in benzene and difluorobenzene, respectively (Scheme 2). This bond lability may imply that [2][BArF] may be capable of reacting as a two coordinated stannyliumylidene, upon dissociation of an IMe_4_ ligand in solution.

### Hydrosilylation of CO_2_ by stannyliumylidene

Inspired by the successful hydrosilylation of CO_2_ catalyzed by germyliumylidene (K),^34^ we attempted to investigate the catalytic potential of [2][BArF] in the same reaction.

We found the optimal conditions for the catalytic reaction of CO_2_ and diphenylsilane at room temperature in the presence of 5 mol% [2][BArF] in the C_6_D_6_ : C_6_H_5_F (4 : 1) solvent mixture. According to the ^1^H NMR spectrum, the consumption of the majority (94%) of the Ph_2_SiH_2_ was observed within 25 hours, resulting in silyl formate, Ph_2_SiH(OCHO), which is then transformed into silylated methanol (Table 1, Entry 1) (Fig. S35†). The catalyst [2][BArF] exhibited poor solubility in benzene, which led to lower catalytic efficiency (Table, Entry 2). To further investigate the impact of polar solvents on the CO_2_ hydrosilylation catalyzed by [2][BArF], 1,2-difluorobenzene (C_6_H_4_F_2_) (Table 1, Entry 4) in different ratios with C_6_D_6_ (Table 1, Entry 5) were selected as reaction solvents. The results indicated that polar solvents did not participate in the hydrosilylation reaction mechanism; their role was limited to enhancing the solubility of [2][BArF]. In comparison to fluorobenzene, the use of THF, a donor solvent, resulted in lower conversion (Table 1, Entry 3). Elevated temperature accelerated the catalytic rate, achieving 64% conversion in 2 hours (Table 1, Entry 6). However, the catalyst decomposed rapidly under these conditions (Fig. S40†). Furthermore, to examine the effect the counter anion has on the conversion we performed this reaction under the same conditions with [2][Al(OC(CF_3_)_3_)_4_], and a decrease in activity was observed (Table 1, Entry 10). This decrease in catalytic activity could indicate that the very weak coordination strength between Al(ORf)_4_^−^ and the Sn atom increases the electrophilicity of the Sn center, which suppresses NHC dissociation from Sn center, compared to BArF^−^.^39^ Furthermore, the hydrosilylation of CO_2_, catalyzed by the [2][BArF], exhibited minimal perturbation in the presence of excess mercury (Hg), strongly suggesting the nature of the catalytic cycle is homogeneous (Table 1, Entry 15).

**Table 1:** Screening of the conditions for the hydrosilylation (H_2_SiPh_2_) of CO_2_

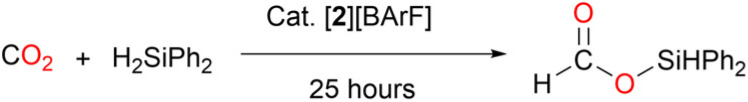		
Entry	Deviation from the standard conditions^a^	Conversion^b^ (%)
1	None	94
2	C_6_D_6_	78
3	THF-d_8_	75
4	C_6_D_6_/C_6_H_4_F_2_ (4 : 1)	97
5	C_6_D_6_/C_6_H_4_F_2_ (1 : 1)	94
6	50 °C	64 (2 hours)
7	0.5 mol%	4
8	1 mol%	11
9	2.5 mol%	74
10	[2][Al(OC(CF_3_)_3_)_4_]	70
11	[3][BArF]^c^	92
12	[4][BArF]^c^	93
13	IMe_4_ (10 mol%)	51
14	IMe_4_–CO_2_ (10 mol%), C_6_D_6_/C_6_H_4_F_2_ (1 : 1)	51
15	Hg^d^	89

a Standard reaction conditions: H_2_SiPh_2_ (80 μmol), 1,3,5-trimethoxylbenzene (8 μmol, internal standard), and 5 mol% [2][BArF] catalysis in C_6_D_6_ (0.32 mL) and C_6_H_5_F (0.08 mL) under 1 bar CO_2_ at RT for 25 hours.

b NMR yield was monitored by ^1^H NMR with methoxybenzene as an internal standard.

c Refer to Scheme 3 for the synthesis of [3][BArF] and [4][BArF].

d Hg to catalyst ratio 250 : 1.

**Scheme 3:**
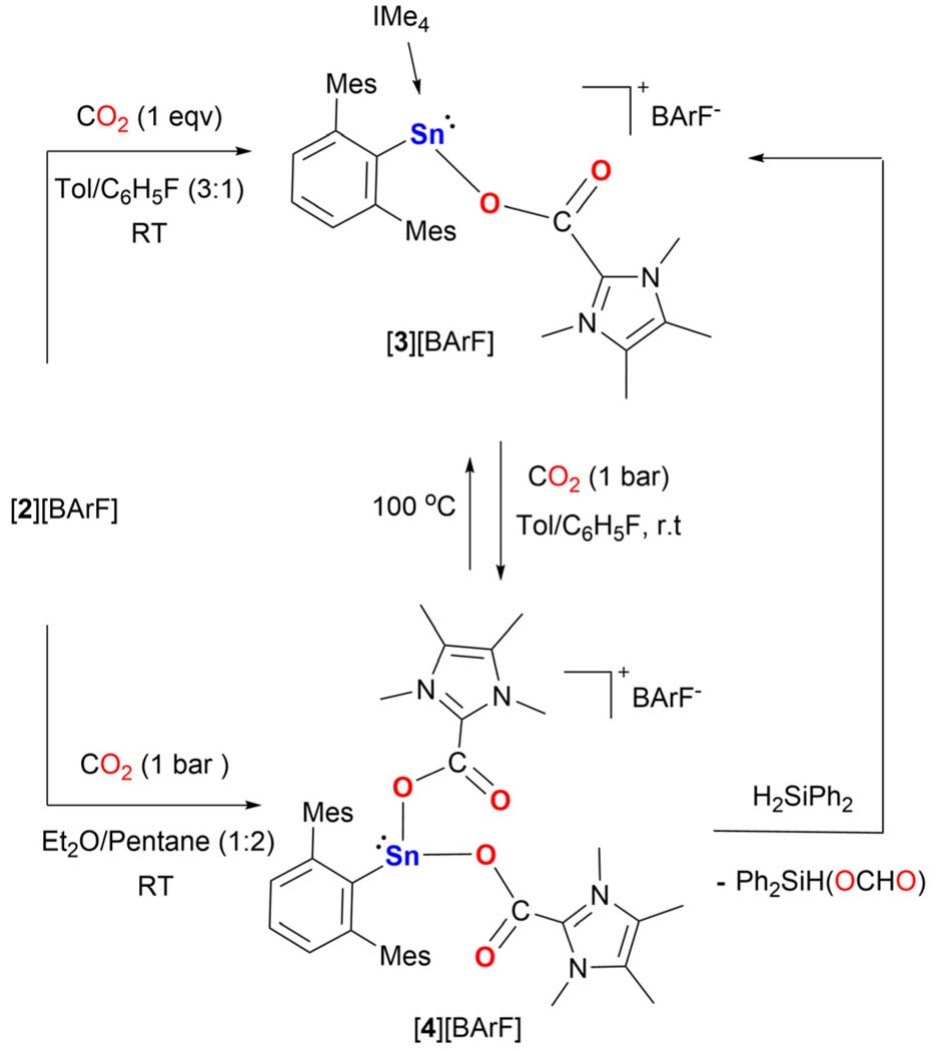
Reactions of NHC-stabilized stannyliumylidene ion [2][BArF] with CO_2_.

In addition to diphenylsilane we tested the catalytic performance of [2][BArF] toward a few other silanes (Table 2). In the case of HSiMe_2_Ph (Table 2, Entry 2), the corresponding silyl formate is formed with a 47% conversion within 25 hours. The catalyst deactivated after consumption of 71% of phenylsilane within 12 hours (Entry 5). The more sterically demanding silanes, HSiPh_3_ and HSiEt_3_, were less effective in the hydrosilylation of CO_2_ even at elevated temperatures (Table 2, Entries 4 and 5).

**Table 2:** Screening of the silanes for the hydrosilylation of CO_2_

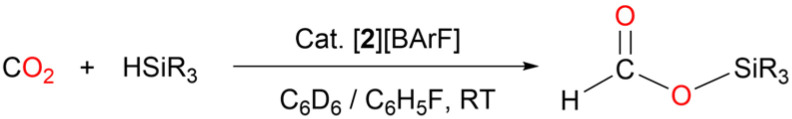				
Entry	Silanes^a^	*T* (°C)	Time (h)	Conversion^b^ (%)
1	H_2_SiPh_2_	RT	25	94
2	HSiMe_2_Ph	RT	25	47
3	H_3_SiPh	RT	12^c^	71
4	HSiEt_3_	60	25	29
5	HSiPh_3_	80	—d	—

a Standard reaction conditions: H_2_SiPh_2_ (80 μmol), 1,3,5-trimethoxylbenzene (8 μmol, internal standard), and 5 mol% [2][BArF] catalysis in C_6_D_6_ (0.32 mL) and C_6_H_5_F (0.08 mL) under 1 bar CO_2_ at RT for 25 hours.

b NMR yield was monitored by ^1^H NMR with methoxybenzene as an internal standard.

c Refer to Scheme 3 for the synthesis of [3][BArF] and [4][BArF].

d Hg to catalyst ratio 250 : 1.

[2][BArF] demonstrated superior performance in CO_2_ hydrosilylation compared to certain Lewis acids, such as [Et_2_Al]^+^ and [Et_3_Si]^+^.^14^ However, it was slightly less effective than [(2,6-Mes_2_C_6_H_3_O)_2_Al]^+^, [2,6-Ph_2_C_6_H_3_AlEt]^+^, and [(2,6-Dipp_2_C_6_H_3_)GaEt]^+^ at elevated temperatures, which produced methane, toluene, and diphenylmethane as products.^15,16^ [2][BArF] was less effective than some FLP catalytic systems, such as the N,P-heterocyclic germylene/B(C_6_F_5_)_3_ pair (H),^22^ and {[TismPribenz]M}[HB(C_6_F_5_)_3_] (M = Zn, Mg),^19^ which selectively produced bis(silyl)acetals or methane. Its performance was comparable to that of germa-acylium ions and germyliumylidene (L), which yielded silyl formate, bis(silyl)acetal, and silylated methanol.^33,34^

Although [2][BArF] exhibits moderate catalytic ability for CO_2_ hydrosilylation, its ability to selectively reduce CO_2_ to silyl formate in a one-step process is valuable for studying the mechanism of CO_2_ hydrosilylation.

### Mechanistic studies

To study the mechanism of the hydrosilylation of CO_2_ catalyzed by [2][BArF], stoichiometric reactivity studies were conducted. Exposing [2][BArF] to 1 equivalent of CO_2_ in toluene:C_6_H_5_F (3 : 1) at room temperature, afforded the mono-insertion product [^Mes^TerSn(CO_2_IMe_4_)IMe_4_][BArF], [3][BArF], after 4 hours. It bears one CO_2_ molecule inserted between the Sn center and one of the IMe_4_ ligands. It has increased solubility in nonpolar organic solvents such as benzene and toluene compared to [2][BArF]. The ^119^Sn{^1^H} NMR spectrum of [3][BArF] shows a resonance at *δ* = −10.61 ppm, which is downfield shifted compared to [2][BArF], due to the Sn being bound to the more electronegative oxygen atom. The ^13^C{^1^H} resonance of the corresponding carboxylate (IMe_4_–*C*(O)OSn) appears at *δ* = 157.48 ppm (THF-d_8_), which is comparable to the reported NHC–CO_2_ adducts, *i.e.*, IPrCO_2_ (IPr = 1,3-bis(2,6-diisopropylphenyl)imidazole-2-ylidene) (*δ* = 152.3 ppm, CD_2_Cl_2_), IMeCO_2_ (IMe = 1,3-dimethylimidazolium) (*δ* = 158 ppm, D_2_O),^40^ I*t*BuCO_2_ (I*t*Bu = 1,3-di-*tert*-butyl-4,5-dimethylimidazolin-2-ylidene) (*δ* = 160 ppm, CD_2_Cl_2_), IMe_4_CO_2_ (*δ* = 155.9 ppm, CD_2_Cl_2_).^41^

**Fig. 7:**
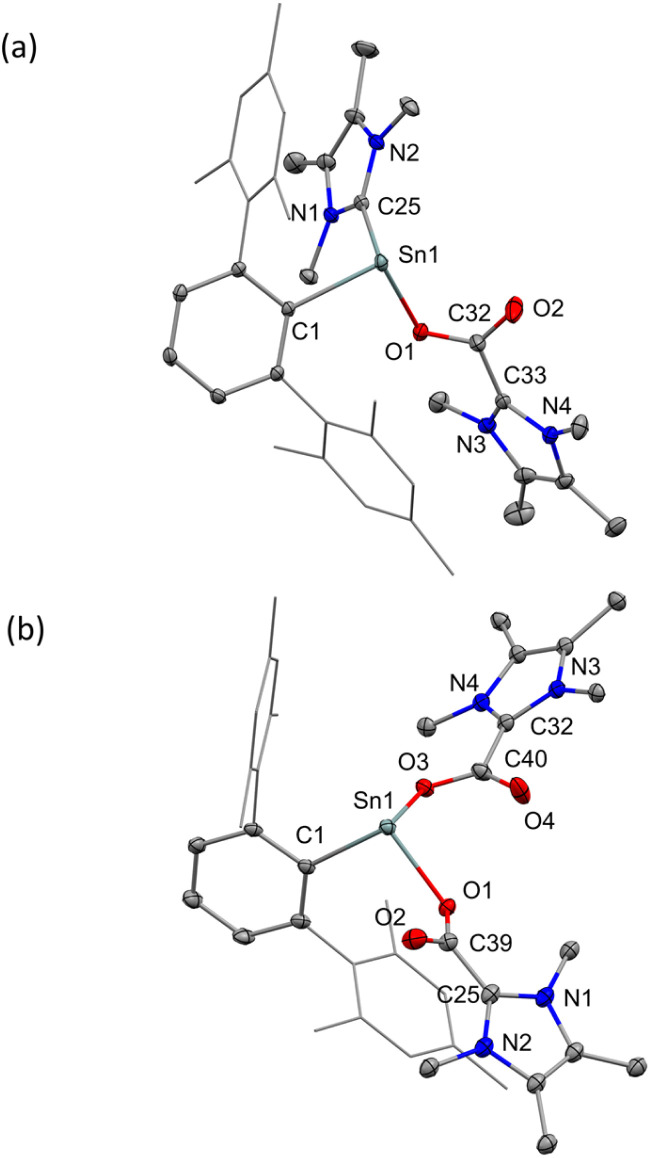
Molecular structures of [3]^+^ (a) and [4]^+^ (b) in the solid state. Ellipsoids are set at the 30% probability level; hydrogen atoms and counterions are omitted for clarity. Selected bond lengths [Å] and bond angles [°]: [3]^+^ C1–Sn1 2.237(3), C25–Sn1 2.283(3), O1–Sn1 2.231(2), O1–C32 1.263(4), C32–O2 1.211(4), C32–C33 1.508(5), C1–Sn1–C25 96.3(1), C1–Sn1–O1 93.38(9), C25–Sn1–O1 86.39(9). [4]^+^ C1–Sn1 2.231(2), Sn1–O1 2.168(2), O1–C39 1.270(3), O2–C39 1.223(3), C39–C25 1.450(1), Sn1–O3 2.230(2), O3–C40 1.279(2), C40–O4 1.220(3), C40–C32 1.522(5), C1–Sn1–O1 96.41(7), C1–Sn1–O3 90.89(7), O1–Sn1–O3 90.12(6).

The SC-XRD analysis of [3][BArF] revealed that the central Sn is tricoordinate with an IMe_4_ ligand, a terphenyl substituent, and an imidazolium carboxylate (Fig. 7(a)). The Sn1–O1 bond length of 2.231(2) Å is longer than that of the reported Gibson's (^Dipp^nacnac)SnO(iPr), (2.000(5) Å),^42^ tin-carbamate complex ((^Dipp^nacnac)SnOC(O)N(*i*Pr_2_)) (2.1346(16) Å)^43^ and shorter than (Ar^NEt2^Ge)(μ-*k*^1^(C)-*k*^2^(O,O′)-CO_2_)(SnAr^NEt2^) (2.301(3), 2.336(3) Å), (Ar^NEt2^ = [2,6-(Et_2_NCH_2_)_2_C_6_H_3_]).^44^ The C–O bond lengths (C39–O1 = 1.263(4), C39–O2 = 1.211(4) Å) with the C–O moiety bound to the Sn center being the longer of the two, are longer than those in free CO_2_ (1.16 Å). This is also in the same range as other imidazolium carboxylates,^45^ IPrCO_2_ (1.221(4) and 1.225(4) Å)^46^ and I*t*Bu_Me_-CO_2_-B(C_6_F_5_)_3_ (I*t*Bu_Me_ = 1,3-di-*tert*-butyl-4,5-dimethylimidazolin-2-ylidene) (1.302(3) and 1.211(3) Å).^46^ The C–C bond length between the IMe_4_ and CO_2_ is 1.508(5) Å in [3][BArF], which is close to the reported imidazolium carboxylates,^41,45,46^ IPrCO_2_ (1.511(4) Å), IMe_4_CO_2_ (1.521 Å) and IEt_Me_CO_2_ (IEt_Me_ = 1,3-diethyl-4,5-methyl-imidazolium) (1.535(15) Å). In addition, the carboxylic carbon atoms exhibit a trigonal-planar coordination environment, with the bond angles of 126.5(3)° for O1–C32–O2, 116.3(3)° for C33–C32–O2 and 117.0(3)° for C33–C32–O1.

The addition of excess CO_2_ (1 bar) to a solution of [2][BArF] in Et_2_O and pentane at room temperature results in the doubly CO_2_ inserted product [^Mes^TerSn(CO_2_IMe_4_)_2_][BArF], [4][BArF], after 21 hours. Yellow crystals suitable for SC-XRD were obtained from an Et_2_O/pentane (1 : 2) solution stored at −35 °C under a CO_2_ atmosphere. The ^119^Sn{^1^H} NMR spectrum of [4][BArF], shows a sharp singlet signal at *δ* = 55.9 ppm, which is shifted downfield compared to [2][BArF] and [3][BArF], due to the Sn center now being bound to two electronegative O atoms. The resonance of the carboxyl carbon atom appears at *δ* = 157.48 ppm in the ^13^C{^1^H} NMR spectrum, which is consistent with [3][BArF]. The SC-XRD of [4][BArF] (Fig. 7(b)) shows that the bond lengths of Sn1–O1 and Sn1–O3 are 2.168(2) Å and 2.230(2) Å, respectively. The C–O bond lengths are as follows: C39–O1 (1.270(3) Å), C40–O3 (1.279(2) Å) and C39–O2 (1.223(3) Å), C40–O4 (1.220(3) Å). The lengths of C–C bonds between the IMe_4_ carbon and the CO_2_ group C39–C25 (1.450(1) Å) and C40–C32 (1.522(5) Å) are different compared to [3][BArF] (1.508(5) Å), but comparable to imidazolium carboxylates.^41,45,46^

The isolation of large quantities of pure solid [4][BArF] is not possible as it decomposes to [3][BArF] under reduced pressure *via* the release of one molecule of CO_2_.

To study the stability of [4][BArF] in solution, ^1^H NMR-monitoring experiments were conducted in a toluene-d_8_/C_6_H_5_F solution under a CO_2_ atmosphere (Fig. S29†). At 100 °C, [4][BArF] slowly converts to [3][BArF], releasing one equivalent of CO_2_. Leaving the sample at ambient temperature for 15 hours resulted in the reformation of [4][BArF].

**Scheme 4:**
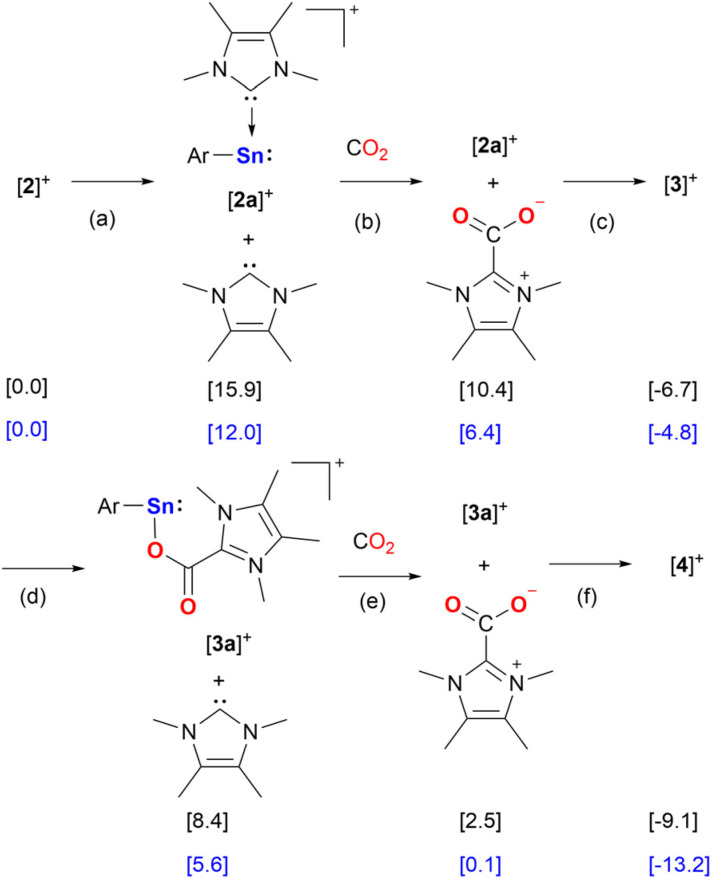
Calculated pathway and relative Gibbs energies for the reaction of [2]^+^ with CO_2_ to give [3]^+^ and [4]^+^ (black). Gibbs energies for the reactions including the counter anion, relative to [2][BArF] are shown in blue.

Calculations at the PW6B95-D4/def2-QZVPP(CPCM)/r^2^SCAN-3c level of theory show that the likely scenario for the formation of [3][BArF] and [4][BArF] is a stepwise reaction, in which the consecutive dissociation of a IMe_4_ ligands from the Sn center of [2][BArF] occurs (Scheme 4). The IMe_4_ ligands react with carbon dioxide to form the imidazolium carboxylates and reassociate with the Sn complex (Scheme 4). The IMe_4_ dissociation from [2][BArF] is endergonic by 12.0 kcal mol^−1^ and proceeds without a barrier yielding [2a][BArF] and free IMe_4_ (Scheme 4, path (a)). The IMe_4_ reacts with a molecule of CO_2_ to form the IMe_4_–CO_2_ imidazolium carboxylate at 6.4 kcal mol^−1^ (Scheme 4, path (b)). The barrier for the reaction of IMe_4_ with CO_2_ is only 9.3 kcal mol^−1^. [3][BArF] forms upon barrierless association of IMe_4_–CO_2_ with [2a][BArF] at −4.8 kcal mol^−1^ (Scheme 4, path (c)). We also considered a mechanism in which [2][BArF] reacts with CO_2_ concertedly, without IMe_4_ dissociation, to give [3][BArF]. However, this scenario requires overcoming a barrier of 29.6 kcal mol^−1^ (Fig. S57†). The formation of [4][BArF] proceeds *via* similar steps from [3][BArF], *i.e.* dissociation of the IMe_4_ from [3][BArF] giving [3a][BArF] + IMe_4_ at 5.6 kcal mol^−1^ (Scheme 4, path (d)), followed by the formation of the imidazolium carboxylate at 0.1 kcal mol^−1^ (Scheme 4, path (e)), and finally association of [3a][BArF] with IMe_4_–CO_2_ to give [4][BArF] at −13.2 kcal mol^−1^ (Scheme 4, path (f)).

Notably, comparison of the spectroscopic data indicated the formation of [3][BArF] and [4][BArF] during the [2][BArF] catalyzed hydrosilylation of CO_2_ (Fig. S35†). Furthermore, [4][BArF] could be converted to [3][BArF] by adding Ph_2_SiH_2_ under an argon atmosphere, with the simultaneous formation of silyl formate (Fig. S31†). DFT calculations for the proposed mechanism of this transformation were performed (see Fig. S58†). Conversely, [3][BArF] decomposed to unidentified compounds when treated with Ph_2_SiH_2_ at room temperature. Both [3][BArF] and [4][BArF] are efficient catalysts for CO_2_ hydrosilylation (Table 1, Entries 11 and 12), comparable to the performance of [2][BArF]. Thus, we propose [4][BArF] as the resting state of the active cycle. Furthermore, control experiments using IMe_4_ and IMe_4_–CO_2_ as catalysts both show lower conversion (Table 1, Entries 13 and 14), suggesting that these compounds are not the catalytically active species.

**Scheme 5:**
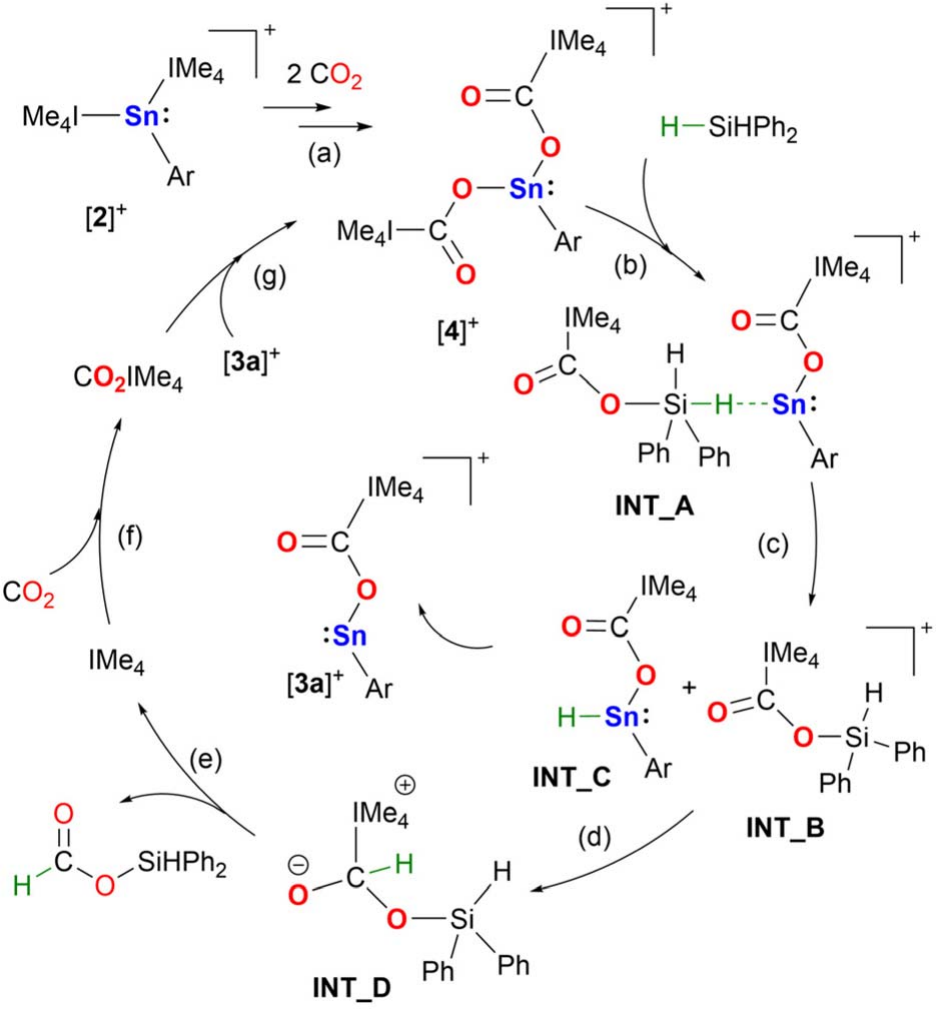
Proposed catalytic cycle of hydrosilylation of carbon dioxide by diphenylsilane in the presence of stannyliumylidene [2]^+^.

Based on our reactivity studies and the quantum chemical calculations (Scheme 4, Fig. S57 and S58†) a mechanism for the catalytic hydrosilylation of CO_2_ using [2][BArF] is proposed (Scheme 5). Under catalytic conditions, the stannyliumylidene [2]^+^ reacts with two equivalents of carbon dioxide to form the double insertion product, [4]^+^, *via* [3]^+^, as described above (Scheme 5, step (a)). Compound [4]^+^ reacts with a molecule of diphenylsilane to form intermediate INT_A (Scheme 5, step (b)), which dissociates to the ((silylcarbonyl)oxy)imidazolium intermediate (INT_B) and the stannyl hydride (INT_C). The former can abstract a hydride from the stannyl hydride, releasing the tin complex [3a]^+^ and forming a zwitterionic adduct, INT_D (Scheme 5, step (d)). This type of adduct is well known in NHC organocatalysis, as similar species can form upon the reaction of NHC with an aldehyde, which can later convert to amino enols, known as Breslow intermediates.^47^ In this case the reverse reaction of the zwitterionic adduct releasing the diphenylsilyl formate product, and the NHC (Scheme 5, step (e)). The NHC reacts with an additional equivalent of CO_2_ forming the imidazolium carboxylate (Scheme 5, step (f)), which reassociates with tin complex [3a]^+^ to regenerate the starting compound [4]^+^ (Scheme 5, step (g)).

**Scheme 6:**
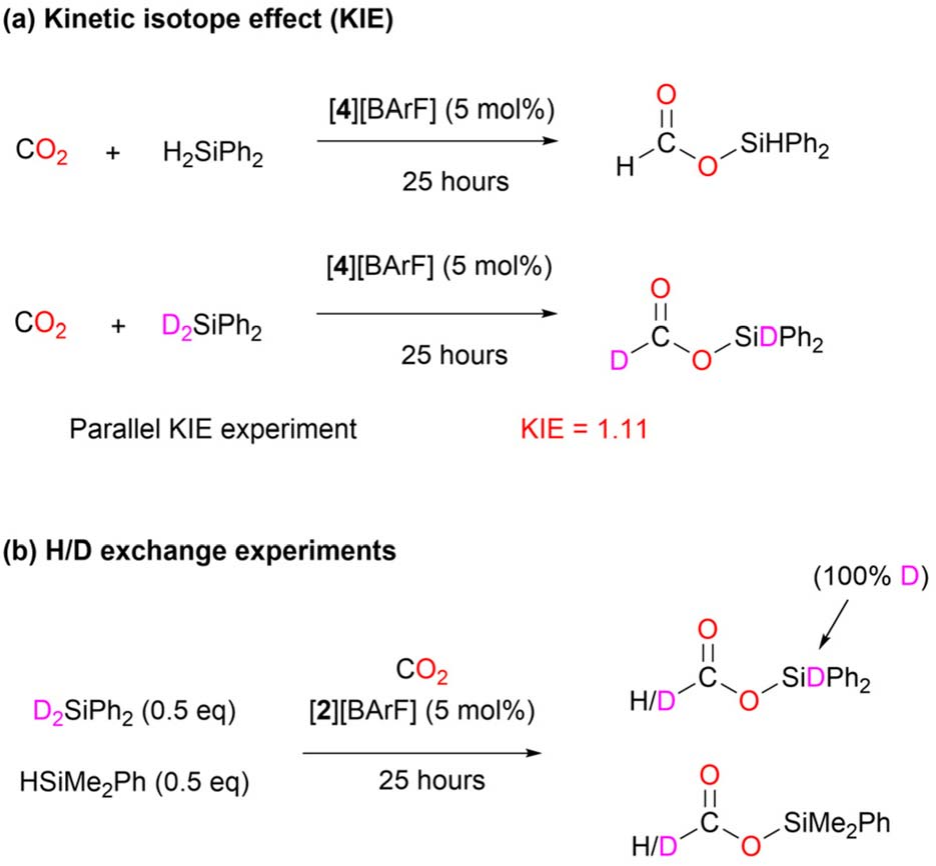
Mechanistic studies.

In addition to the isolation of the two key intermediates of the catalytic cycle ([3][BArF] and [4][BArF]) and the quantum chemical calculations, we carried out additional experiments (Scheme 6), using deuterated silanes (D_2_SiPh_2_), to support the reaction mechanism proposed. Under standard conditions, the rate of the reactions with H_2_SiPh_2_ and D_2_SiPh_2_ were compared, giving a kinetic isotope effect (KIE) of 1.11 (Scheme 6, (a)). This value indicates a secondary isotope effect, which is consistent with the rate-determining transition state TS4 in the proposed mechanism (Fig. S58†), in which the H/D atom is a substituent on carbon from which the NHC moiety is dissociated.^48^ Furthermore, CO_2_ hydrosilylation catalyzed by [2][BArF] was performed in the presence of 1 : 1 mixture of D_2_SiPh_2_ and HSiMe_2_Ph (Scheme 6, (b)). H/D exchange was observed at the carbon center, supporting the formation of the tin-hydride intermediate INT_C (Scheme 5) in the proposed catalytic cycle.^49^ This intermediate can form either *via* deuterium abstraction from D_2_SiPh_2_ or *via* hydrogen abstraction from HSiMe_2_Ph. Subsequently, INT_C, whether D/H-substituted, can transfer deuterium or hydrogen to the ((silylcarbonyl)oxy)imidazolium INT_B, which can also from in the previous step from either D_2_SiPh_2_ or HSiMe_2_Ph. The H/D transfer from INT_C to INT_B will ultimately produce Ph_2_SiD(OCDO), Ph_2_SiD(OCHO), Me_2_PhSi(OCDO), and Me_2_PhSi(OCHO).

## Conclusions

In summary, we reported on the synthesis of a di-NHCs-stabilized stannyliumylidene [2][BArF], which can act as an efficient carbon dioxide hydrosilylation catalyst under ambient conditions. Both experimental and computational studies have been performed to elucidate the reaction mechanism, revealing it to be distinct from those previously proposed for the lighter germyliumylidenes.^34^ In the latter case, [^Mes^TerGe(IMe_4_)_2_]^+^ (K, Fig. 2) was identified as the active catalytic species, with the Si–H bond activation occurring through coordination with the germanium lone pair (L, Fig. 2), which constitutes the rate-determining step. In comparison to K, [2][BArF] captures CO_2_*via* dissociation of NHC, followed by reassociation of the NHC–CO_2_ moiety to the Sn center, forming the catalytically active species, [4][BArF]. This divergent mechanism is another example of how the lighter and heavier congeners can behave differently and is something we hope to exploit in our further studies in applying tetryliumylidene cations to catalytic transformations.

## Data availability

The data supporting this article have been included as part of the ESI.†

## Author contributions

D. N. and D. S. carried out the synthetic and reaction studies, and D. N. wrote the original draft. A. K. carried out the computational studies. J. A. K. conducted the crystallographic studies. A. K., J. A. K, H. X., and D. S. reviewed and edited the draft. S. I. managed the project.

## Conflicts of interest

There are no conflicts to declare.

## Supplementary Material

SC-016-D4SC07116F-s001

SC-016-D4SC07116F-s002
