# Supplementary material for: CO2 hydrosilylation catalyzed by an N-heterocyclic carbene (NHC)-stabilized stannyliumylidene
Source: Chem Sci. 2025 Jan 22;16(9):4014–22. doi: 10.1039/d4sc07116f (PMC11789673; doi:10.1039/d4sc07116f)
Supplement: SC-016-D4SC07116F-s001 [file SC-016-D4SC07116F-s001.pdf]

Supplementary Information (SI) for Chemical Science.
This journal is © The Royal Society of Chemistry 2025

# Supporting Information

## $CO_2$ Hydrosilylation Catalyzed by an N-Heterocyclic Carbene (NHC)-stabilized stannyliumylidene

*Dechuang Niu, Arseni Kostenko, John A. Kelly, Debotra Sarkar, Huihui Xu, and Shigeyoshi Inoue**

TUM School of Natural Sciences, Department of Chemistry, Technische Universität München, Lichtenbergstraße 4, 85748 Garching bei München, Germany.

*E-mail: s.inoue@tum.de

1. Experimental Details .......... 3
1.1 General Methods and Instrumentation .......... 3
1.2 Synthesis of Stannyliumylidene [**2**]$^+$ .......... 3
1.2.1 Synthesis of [**2**][BArF] .......... 3
1.2.2 Synthesis of [**2**][$Al(OC(CF_3)_3)_4$] .......... 8
1.3 Synthesis of [**3**][BArF] .......... 12
1.4 Synthesis of [**4**][BArF] .......... 16
1.5 NMR Experiments .......... 22
1.5.1 The thermal stability of [**4**][BArF] .......... 22
1.5.2 [**4**][BarF] reaction with $Ph_2SiH_2$ .......... 24
1.6 Catalytic Experiments .......... 24
2. Mechanistic Studies .......... 39
2.1 Kinetic Isotope Experiments .......... 39
2.2 H/D Exchange Experiments .......... 41
3. X-Ray Crystallographic Details .......... 42
4. Quantum chemical calculations .......... 44
5. References .......... 66

## 1. Experimental Details

### 1.1 General Methods and Instrumentation

All experiments and manipulations were carried out under a dry oxygen-free argon atmosphere using standard Schlenk techniques or in a glovebox. All glass junctions were coated with PTFE-based grease Merkel Triboflon III. Solvents were dried by standard methods (withdrawn from MBraun Solvent Purification System (SPS) over molecular (3 Å)) or distilled from sodium/benzophenone or $CaH_2$ under argon atmosphere and degassed via freeze-pump-thaw cycling). The $^{1}H$, $^{13}C\{^{1}H\}$, $^{119}Sn\{^{1}H\}$, $^{11}B\{^{1}H\}$ and $^{19}F\{^{1}H\}$ NMR spectra of the compounds were measured on Bruker 400 MHz and 300 MHz spectrometer. Chemical shifts are referenced to (residual) solvent signals ($^{1}H$ and $^{13}C\{^{1}H\}$ NMR). Deuterated benzene ($C_6D_6$), deuterated toluene (toluene-$d_8$) and deuterated tetrahydrofuran (THF-$d_8$) were obtained from Sigma-Aldrich Deutero Chemie GmbH, stored over 4 Å molecular sieves in the glovebox. Carbon dioxide (5.0) was purchased from Westfalen AG and used as received. Unless otherwise stated, all reagents were purchased from commercial sources and used as received. Abbreviations: s = singlet, br = broadened, d = doublet, t = triplet, m = multiplet, dec = decompose. Some NMR spectra include resonances for silicone grease ($C_6D_6$: $\delta(^{1}H)$ = 0.29 ppm, $\delta(^{13}C)$ = 1.4 ppm; δ = THF-$d_8$: $\delta(^{1}H)$ = 0.11 ppm, $\delta(^{13}C)$ = 1.2 ppm). Liquid Injection Field Desorption Ionization Mass Spectrometry (LIFDI-MS) were measured directly from an inert atmosphere glovebox with a Thermo Fisher Scientific Exactive Plus Orbitrap equipped with an ion source from Linden CMS. The UV-vis spectra were taken on a *Agilent Cary 50* spectrophotometer with a *Schlenk* quartz cuvette as the Central Anlytic Department at the TUM Catalysis Research Center. Quantitative elemental analyses (EA) were carried out using an EURO EA (HEKA tech) instrument equipped with a CHNS combustion analyzer at the Laboratory for Microanalysis at the TUM Catalysis Research Center. For fluoride determination, the sample is digested in an alkaline medium. Fluoride content is then measured directly by potentiometry using a Titrando 904 (Metrohm). For tin determination, the sample is acid digested. Measurements are conducted at a wavelength of 235.5 nm in an acetylene/nitrous oxide flame using atomic absorption spectroscopy (Agilent 280 FS-AA). Melting Points (m.p.) were determined in sealed glass capillaries under inter gas by a Büchi M-540. $IMe_4$ (1,3,4,5-tetramethylimidazaol-2-ylidene), Chlorostannylene [$^{Mes}$TerSnCl] (**1**), Sodium tetrakis [3,5-bis(trifluoromethyl)phenyl] borate (NaBArF), and $Li[Al(OC(CF_3)_3)_4]$ were synthesized according to the literature procedures.[S1]

### 1.2 Synthesis of Stannyliumylidene [**2**]$^{+}$

#### 1.2.1 Synthesis of [**2**][BArF]

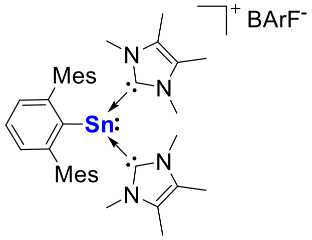


[**2**][**BArF**]: A solution of $IMe_4$ (2.00 eq, 531.0 mg, 4.28 mmol) in toluene (3 mL) was added dropwise to [$^{Mes}$TerSnCl] (1.00 eq, 1.00 g, 2.14 mmol) dissolved in toluene (10 mL) at room temperature. After stirring for 10 minutes, NaBArF ([BArF] = 1,3-bis(trifluoromethyl)-boron) (1.00 eq, 1.90 g, 2.14 mmol) was introduced, resulting in the immediate formation of a brown precipitate. Following this, fluorobenzene ($C_6H_5F$) (3 mL) was

added, and the reaction was allowed to stir overnight at room temperature. The mixture was then filtered, and the filtrate was concentrated to a volume of 5 mL. After 2 days at −35 °C, colourless crystals suitable for XRD analysis were obtained. Yield: 2.65 g (1.72 mmol, 80.4%).

**$^{1}$H NMR (400 MHz, 298 K, THF-d$_8$)**: δ[ppm] = 2.08 (s, 12 H, 4xC-C*H*$_3$, NHC), 2.09 (s, 6 H, 2xC$^{4}$-C*H*$_3$, Mes), 2.15 (s, 12H, 2xC$^{2,6}$-C*H*$_3$, Mes), 3.41 (s, 12 H, 4xN-C*H*$_3$, NHC), 6.69 (s, 4 H, 2xC$^{3,5}$-*H*, Mes), 7.04 (d, $^{3}J_{H-H}$ = 8 Hz, 2 H, C$^{3,5}$-*H*, C$_6$H$_3$), 7.38 (t, $^{3}J_{H-H}$ = 8 Hz, 1 H, C$^{4}$-*H*, C$_6$H$_3$), 7.59, 7.79 (s, 12 H, 4x*C*$^{2,4,6}$, 4C$_6$H$_3$(CF$_3$)$_2$).

**$^{13}$C{$^{1}$H} NMR (101 MHz, 298 K, THF-d$_8$):** δ[ppm] = 8.68 (2xC$^{4,5}$-*C*H$_3$, NHC), 20.99 (C$^{4}$-*C*H$_3$, Mes), 21.81 (C$^{2,6}$-*C*H$_3$, Mes), 36.38 (4xN-*C*H$_3$, NHC), 118.13 (*p*-*C*H$_{B-Ar}$), 121.52, 124.22, 126.93 (*C*, BArF), 128.01 (*C*$^{4,5}$-*C*H$_3$, NHC), 128.50 (*C*$^{4}$-H, C$_6$H$_3$), 128.56 (4x*C*$^{3,5}$-H, Mes), 129.61 (*C*$^{3,5}$-H, C$_6$H$_3$), 129.95-130.54 (m-*C*$_{B-Ar}$), 135.66 (o-CH$_{B-Ar}$), 136.52 (2x*C*$^{1}$-Mes), 137.22 (2x*C*$^{4}$-Mes), 141.73 (2x*C*$^{2,6}$-Mes), 150.00 (2xC$^{2,6}$-C$_6$H$_3$),159.66 (Sn-*C*, C$_6$H$_3$), 159.66-163.62 (ipso-CB-Ar), 170.18 (Sn-*C*, NHC).

**$^{119}$Sn{$^{1}$H} NMR (150 MHz, 298 K, THF-d$_8$):** δ[ppm] = −235.72 (Sn).

**$^{11}$B{$^{1}$H} NMR (128 MHz, 298 K, THF-d$_8$):** δ[ppm] = −6.52 (B, BArF).

**$^{19}$F{$^{1}$H} NMR (376 MHz, 298 K, THF-d$_8$):** δ[ppm] = −63.45 (C*F*$_3$, BArF).

**LIFDI-MS** m/z $C_{38}H_{49}N_4Sn$ calcd: 681.2979; found: 681.2932.

**Elemental analysis (%)** calcd for $C_{70}H_{61}BF_{24}N_4Sn$: C 54.46, H 3.98, N 3.63, F 29.54, Sn 7.69; found: C 54.42, H 4.25, N 3.72, F 26.5, Sn 7.3.

**m.p.**: 126 °C.

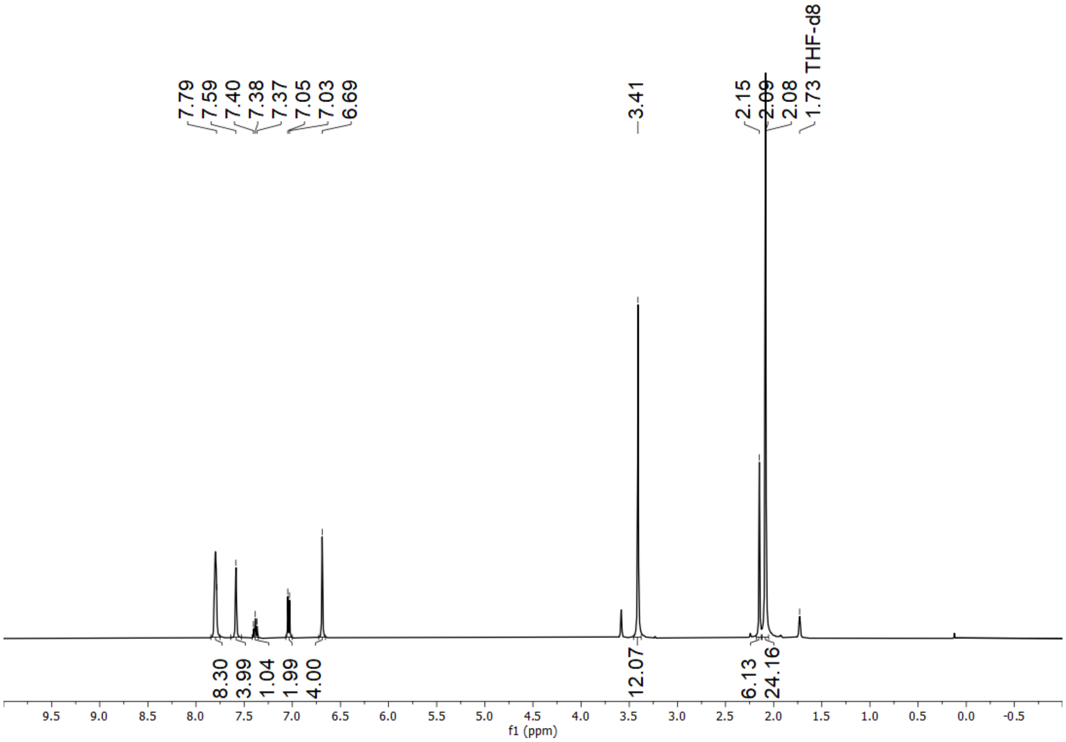


**Figure S1**. $^{1}$H NMR spectrum of [**2**][**BArF**] in THF-$d_8$ at 298 K.

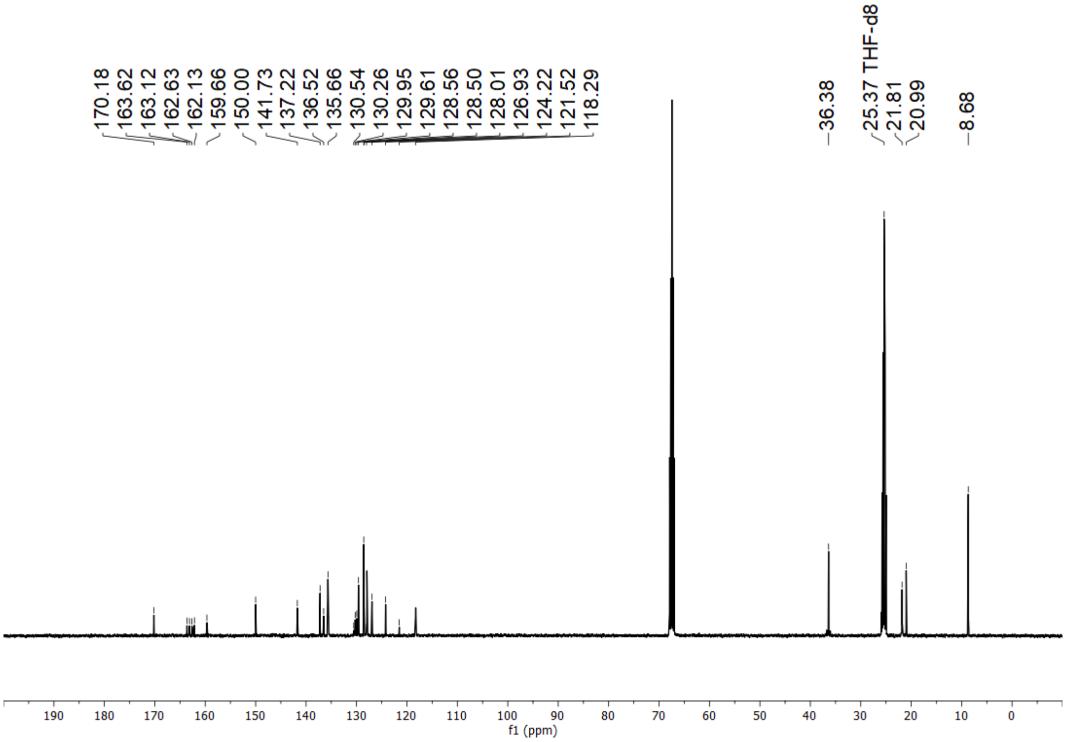


**Figure S2**. $^{13}$C{$^{1}$H} NMR spectrum of [**2**][**BArF**] in THF-$d_8$ at 298 K.

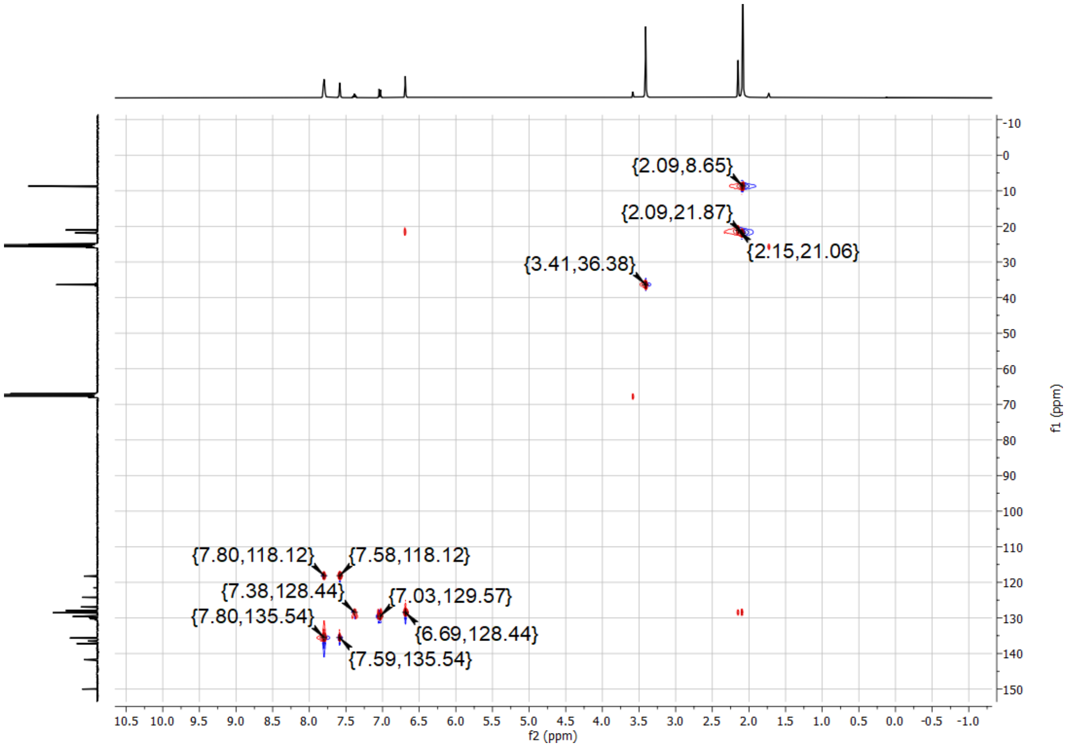


**Figure S3**. $^{1}H/^{13}C$ NMR HSQC spectrum of [**2**][**BArF**] in THF-$d_8$ at 298 K.

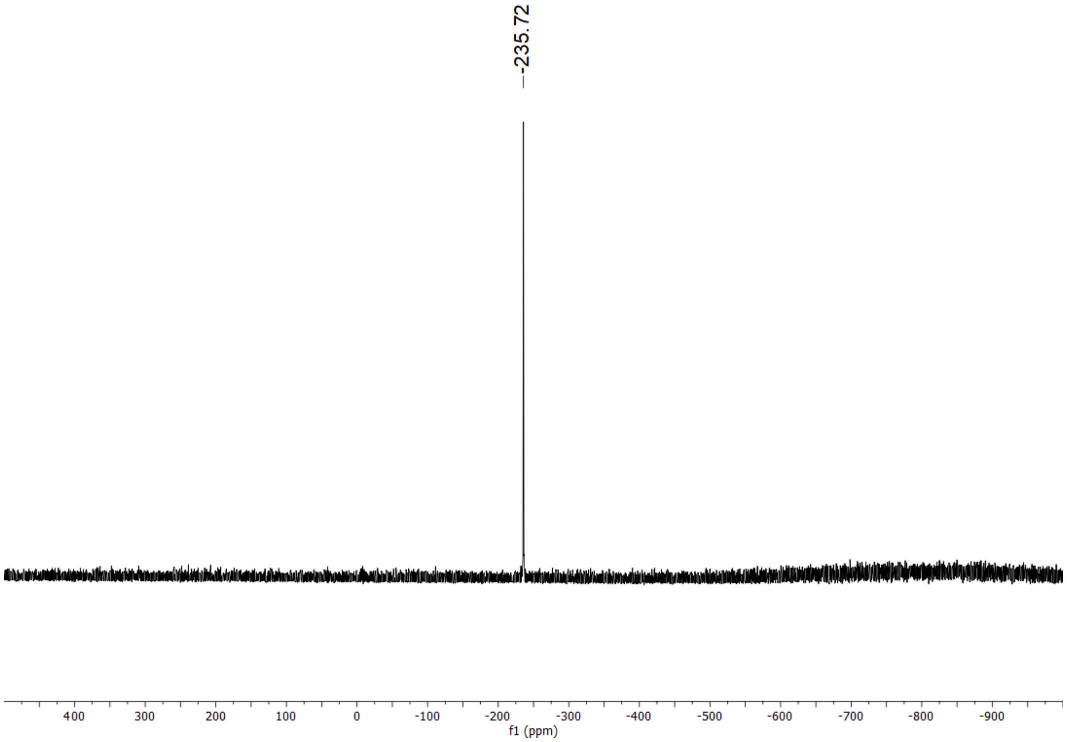


**Figure S4**. $^{119}Sn\{^{1}H\}$ NMR spectrum of [**2**][**BArF**] in THF-$d_8$ at 298 K.

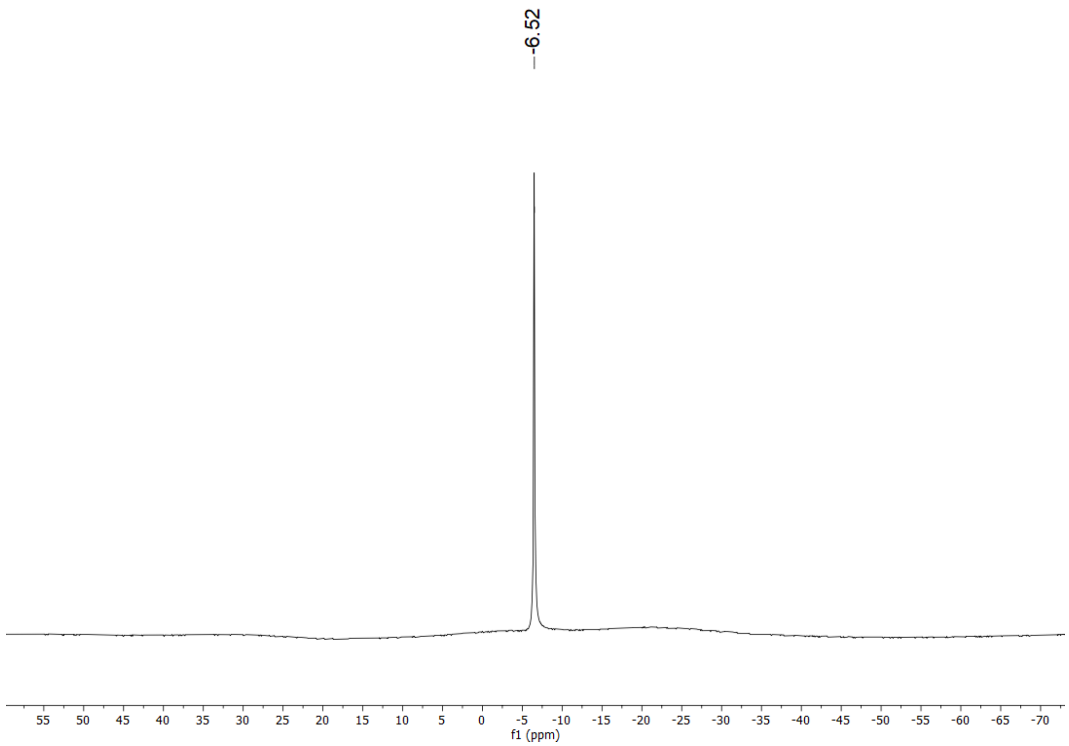


**Figure S5**. $^{11}B\{^{1}H\}$ NMR spectrum of [**2**][**BArF**] in THF-$d_8$ at 298 K.

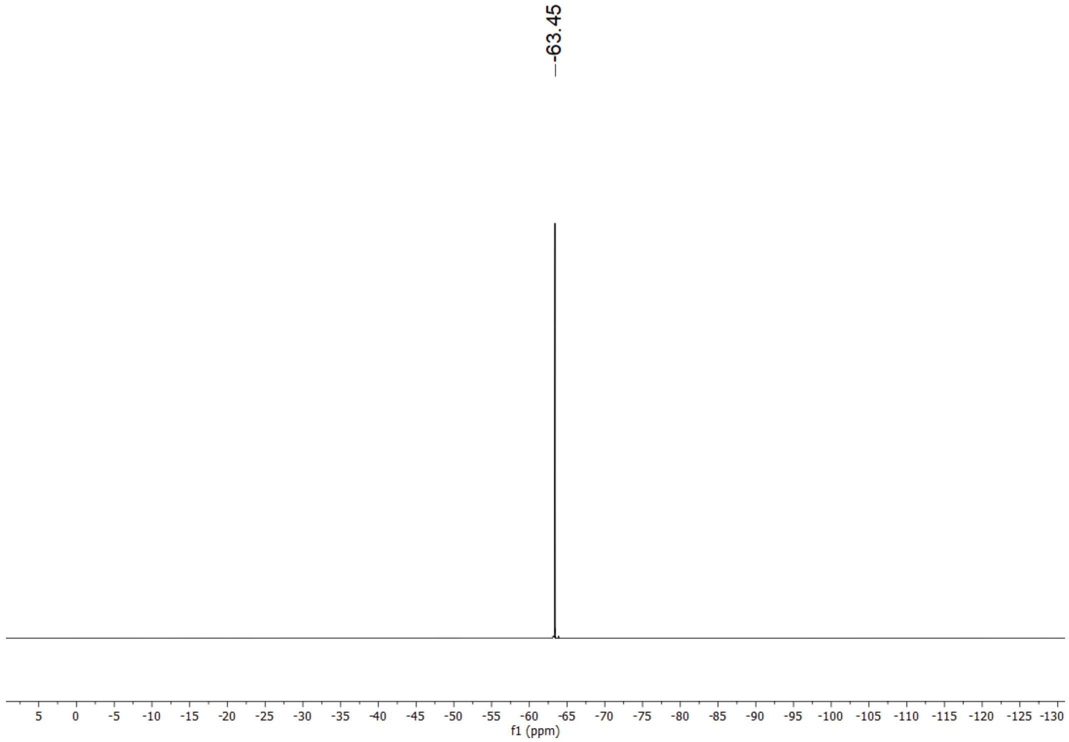


**Figure S6**. $^{19}F\{^{1}H\}$ NMR spectrum of [**2**][**BArF**] in THF-$d_8$ at 298 K.

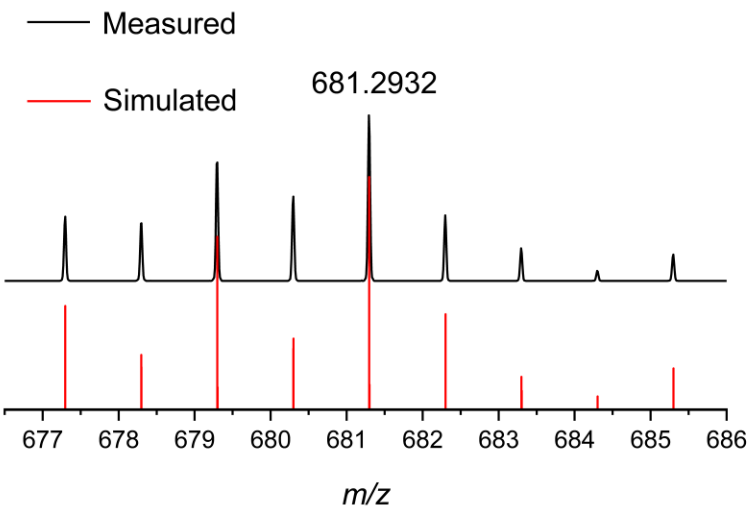


**Figure S7**. LIFDI-MS spectrum (detail view) of [**2**]$^+$ in [**2**][BArF] (measured spectrum: top; simulated spectrum: bottom).

1.2.2 Synthesis of [**2**][Al(OC(CF$_3$)$_3$)$_4$]

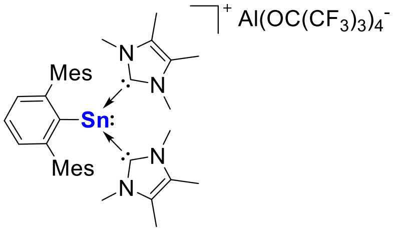


[**2**][Al(OC(CF$_3$)$_3$)$_4$]: A solution of IMe$_4$ (2.00 eq, 53.1 mg, 0.43 mmol) in toluene (2 mL) was added dropwise to [$^{Mes}$TerSnCl] (1.00 eq, 100.0 mg, 0.21 mmol) dissolved in toluene (3 mL) at room temperature. After stirring for 10 minutes, Li[Al(OC(CF$_3$)$_3$)$_4$] (1.00 eq, 229.9 mg, 0.21 mmol) was introduced, leading to the immediate formation of a brown precipitate. Subsequently, fluorobenzene (1 mL) was added, and the reaction was stirred overnight at room temperature, producing a colorless suspension. The mixture was filtered, and the filtrate was concentrated and stored at −35 $^o$C. After 2 days, colorless crystals formed. Yield: 281.0 mg (0.18 mmol, 86.3%).

**$^1$H NMR (400 MHz, 298 K, THF-d$_8$)**: δ[ppm] = 2.08 (s, 12 H, 2xC$^4$-C*H*$_3$, Mes), 2.09 (s, 12 H, 4xC-C*H*$_3$, NHC), 2.15 (s, 6 H, 2xC$^{2,6}$-C*H*$_3$, Mes), 3.41 (s, 12 H, 4xN-C*H*$_3$, NHC), 6.69 (s, 4 H, 2xC$^{3,5}$-*H*, Mes), 7.03 (d, $^3J_{H\text{-}H}$=8 Hz, 2 H, C$^{3,5}$-*H*, C$_6$H$_3$ ), 7.38 (t, $^3J_{H\text{-}H}$= 4 Hz, 1 H, C$^4$-*H*, C$_6$H$_3$).

**$^{13}$C{$^1$H} NMR (101 MHz, 298 K, THF-d$_8$):** δ[ppm] = 8.79 (2xC$^{4,5}$-*C*H$_3$, NHC), 21.10 (C$^4$-*C*H$_3$, Mes), 21.92 (C$^{2,6}$-*C*H$_3$, Mes), 36.49 (4xN-*C*H$_3$, NHC), 120.99, 123.90 (*C*,

Al(O*C*(*C*F$_3$)$_3$)$_4$), 128.07 ($C^{4,5}$-CH$_3$, NHC), 128.60 ($C^4$-H, C$_6$H$_3$), 128.68 (4x$C^{3,5}$-H, Mes), 129.72 ($C^{3,5}$-H, C$_6$H$_3$), 136.63 (2x$C^1$-Mes), 137.35 (2x$C^4$-Mes), 141.85 (2x$C^{2,6}$-Mes), 150.12 (2x$C^{2,6}$-C$_6$H$_3$),159.79 (Sn-*C*, C$_6$H$_3$), 170.30 (Sn-*C*, NHC).

**$^{119}$Sn{$^1$H} NMR (149 MHz, 298 K, THF-d8):** δ[ppm] = −235.31 (Sn).

**$^{19}$F{$^1$H} NMR (376 MHz, 298 K, THF-d$_8$):** δ[ppm] = −75.89 (C*F*$_3$, Al(OC(CF$_3$)$_3$)$_4$).

**LIFDI-MS** m/z $C_{38}H_{49}N_4Sn$ calcd: 681.2979; m/z found: 681.2949.

**m.p.**: 148 °C

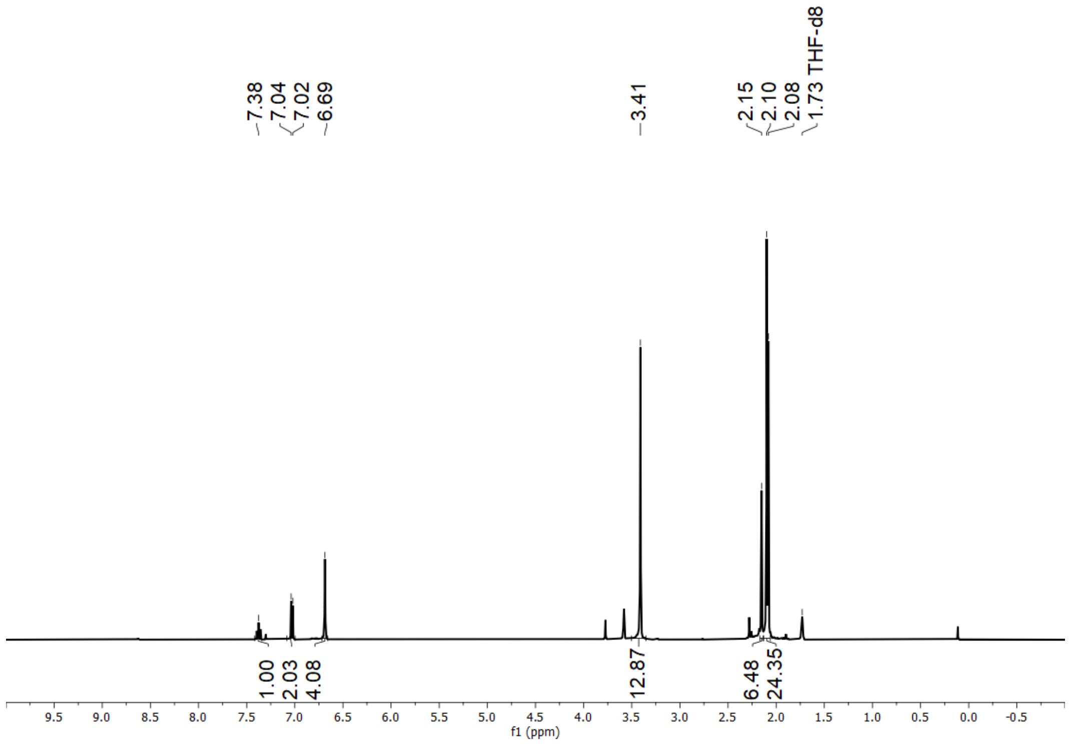


**Figure S8**. $^1$H NMR spectrum of [**2**][Al(OC(CF$_3$)$_3$)$_4$] in THF-d$_8$ at 298 K.

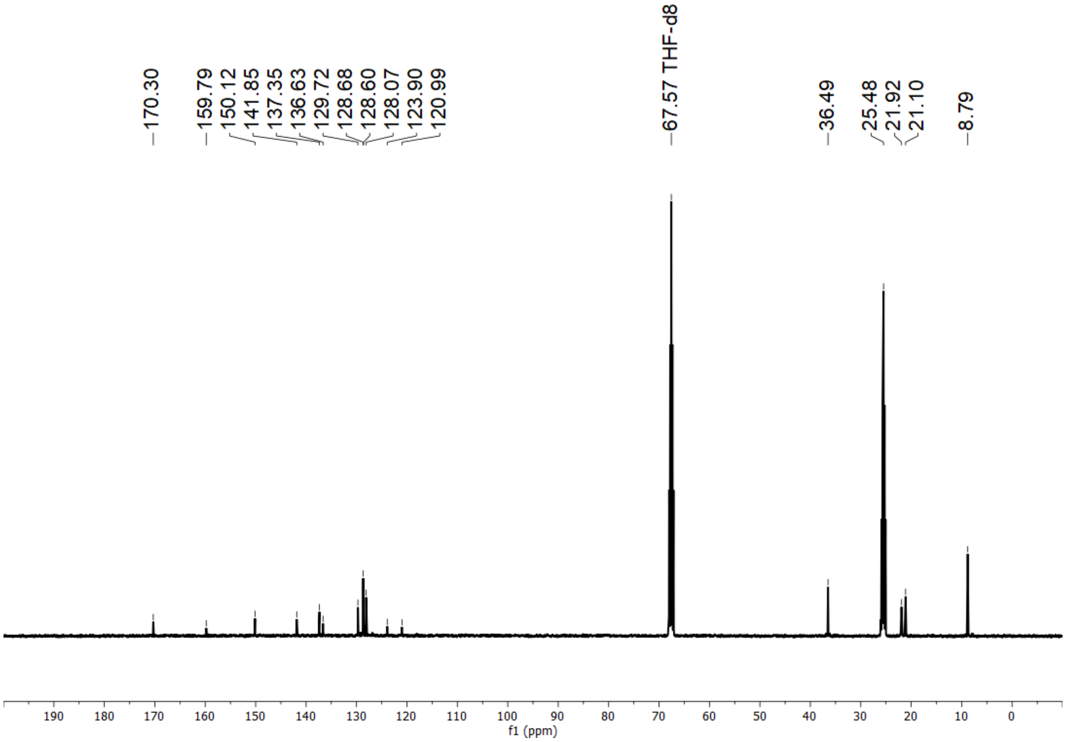


**Figure S9**. $^{13}C\{^1H\}$ NMR spectrum of [**2**][$Al(OC(CF_3)_3)_4$] in THF-$d_8$ at 298 K.

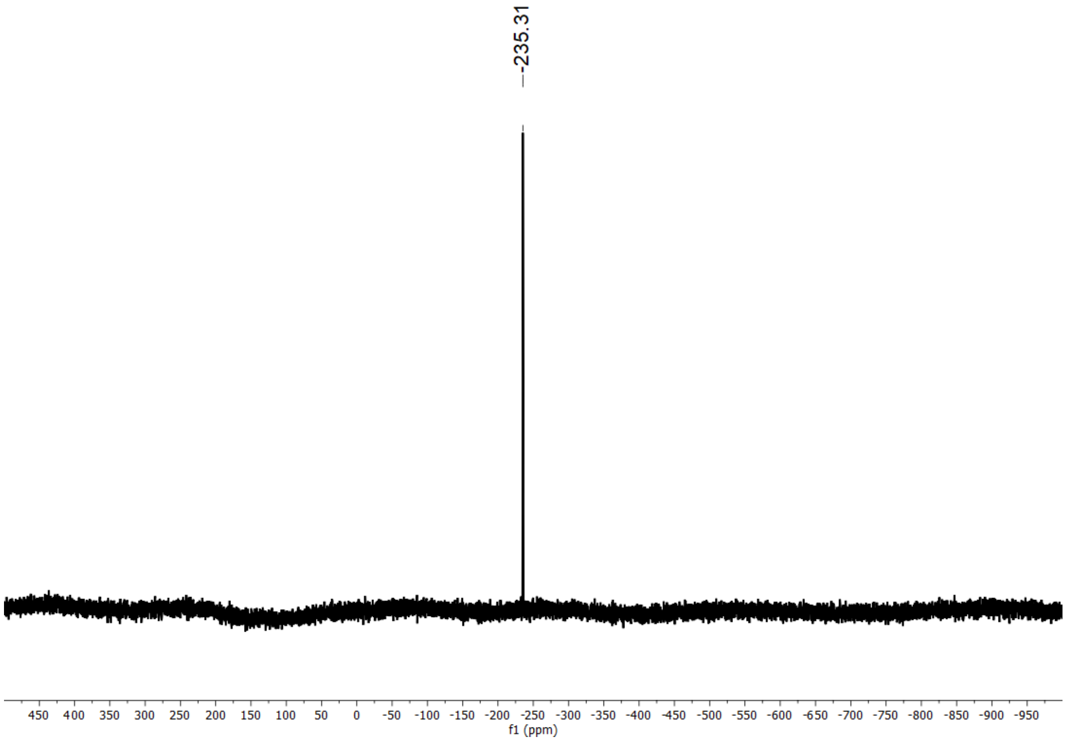


**Figure S10**. $^{119}Sn\{^1H\}$ NMR spectrum of [**2**][$Al(OC(CF_3)_3)_4$] in THF-$d_8$ at 298 K.

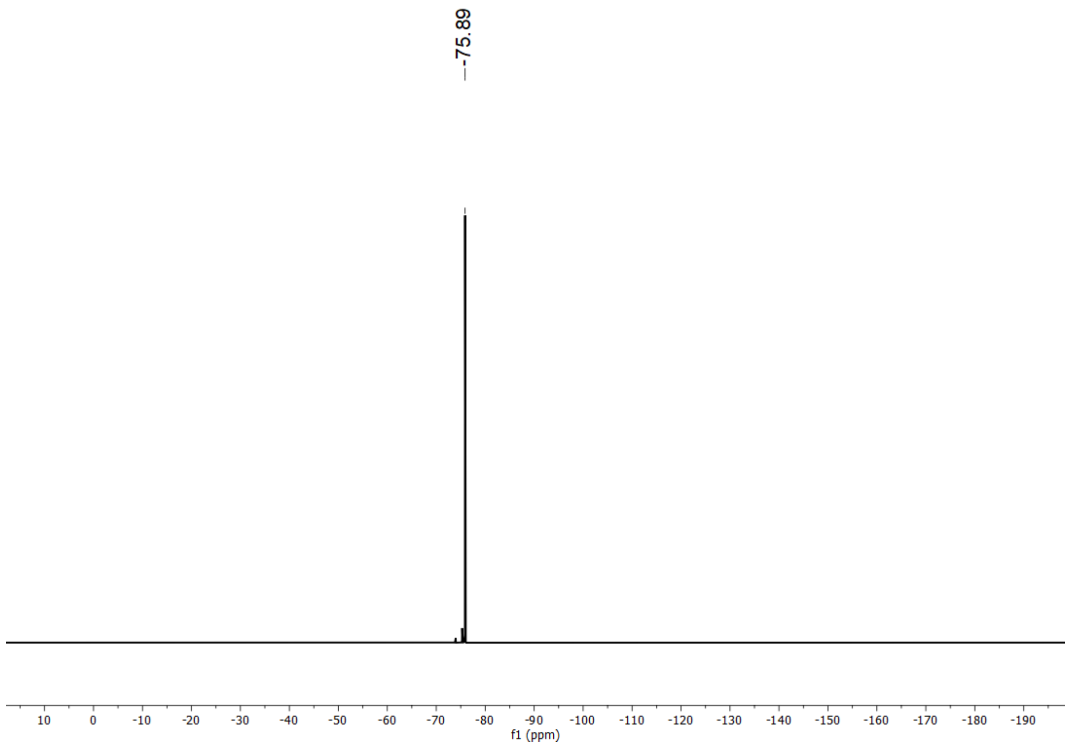


**Figure S11**. $^{19}F\{^{1}H\}$ NMR spectrum of [**2**][Al(OC($CF_3$)$_3$)$_4$] in THF-$d_8$ at 298 K.

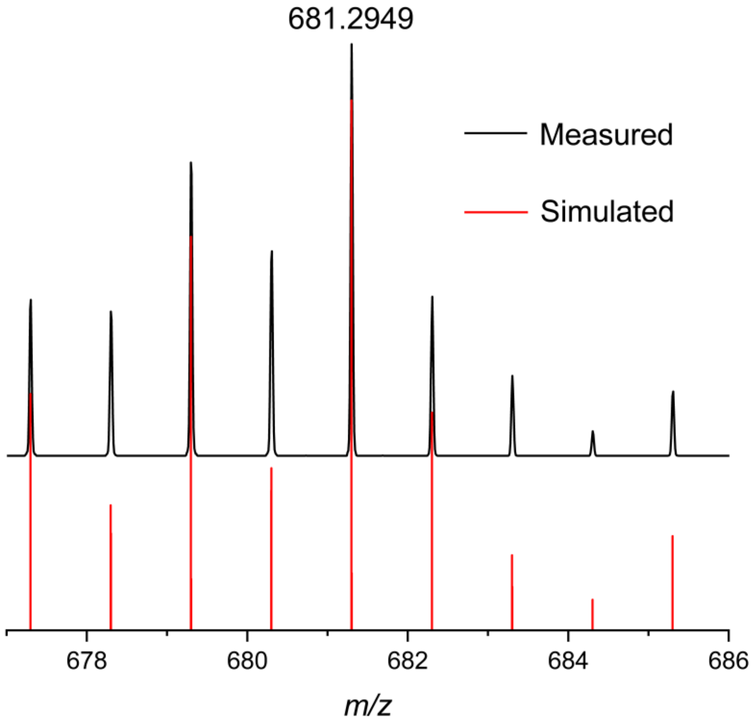

**Figure S12.** LIFDI-MS spectrum (detail view) of [**2**]$^+$ in [**2**][Al(OC(CF$_3$)$_3$)$_4$] (measured spectrum: top; simulated spectrum: bottom).

1.3 Synthesis of [**3**][BArF]

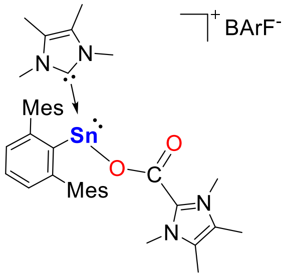


[**2**][BArF] (1.00 eq, 200.0 mg, 0.13 mmol) was dissolved in a mixture of toluene (3 mL) and fluorobenzene (1 mL). $CO_2$ (1.1 eq, 3.20 mL, 1 bar) was then added via a syringe at room temperature. After stirring for 4 hours, the solution changed from colorless to pale yellow. The mixture was filtered, and the filtrate was concentrated to 3 mL before being stored at −35 °C. After 2 days, yellow crystals suitable for XRD analysis were obtained after. Yield: 126.0 mg (0.080 mmol, 61.3%).

**$^1$H NMR (400 MHz, 298 K, THF-d$_8$):** δ[ppm] = 1.94, 2.21 (s, 12 H, 2xC$^{2,6}$-C*H*$_3$, Mes), 2.10 (s, 6 H, 2xC$^4$-C*H*$_3$, Mes), 2.11(s, 12 H, 2xC-C*H*$_3$, NHC), 2.28(s, 6 H, 2xC-C*H*$_3$, *NHC*-COO), 3.36 (s, 6 H, 2xN-C*H*$_3$, NHC), 3.74 (s, 6 H, 2xN-C*H*$_3$, *NHC*-COO), 6.69, 6.77 (s, 4H, 2xC$^{3,5}$-*H*, Mes), 6.91 (d, $^3J_{H-H}$= 7.5 Hz, 2 H, C$^{3,5}$ -*H*, C$_6$H$_3$), 7.30 (t, $^3J_{H-H}$= 8 Hz, 1 H, C$^4$-*H*, C$_6$H$_3$), 7.59, 7.80 (s, 12 H, 4x*C*$^{2,4,6}$, 4C$_6$H$_3$(CF$_3$)$_2$).

**$^{13}$C{$^1$H} NMR (101 MHz, 298 K, THF-d$_8$):** δ[ppm] = 8.32 (C$^{4,5}$-*C*H$_3$, *NHC*-COO), 8.39 (*C*$^{4,5}$-Me, NHC), 20.96 (C$^4$-*C*H$_3$, Mes), 21.60, 21.03 (C$^{2,6}$-*C*H$_3$, Mes), 34.34 (2xN-*C*H$_3$, *NHC*-COO), 35.65 (2xN-*C*H$_3$, NHC), 118.26 (*p*-CH$_{B-Ar}$), 121.52, 124.23, 126.93, 127.11 (*C*, BArF), 128.06 (*C*$^4$-H, C$_6$H$_3$), 128.81 (*C*$^{4,5}$-CH$_3$, *NHC*-COO), 128.87 (*C*$^{4,5}$-CH$_3$, NHC), 128.95 (4x*C*$^{3,5}$-H, Mes), 129.64 (2x*C*$^{2,6}$-Mes), 129.82 (*C*$^{3,5}$-H, C$_6$H$_3$), 129.95-130.55 (m-C$_{B-Ar}$), 135.67 (o-CH$_{B-Ar}$), 136.64 (2x*C*$^4$-Mes), 136.69 (N$_2$*C*, NHC), 137.28 (2x*C*$^{2,6}$-Mes), 140.98 (2x*C*$^4$-Mes), 148.86 (2x*C*$^{2,6}$-C$_6$H$_3$), 157.48 (C*C*O$_2$), 162.13-163.62 (ipso-CB-Ar), 164.94 (Sn-*C*, C$_6$H$_3$), 177.70 (Sn-*C*, NHC).

**$^{119}$Sn{$^1$H} NMR (149 MHz, 298 K, THF-d8):** δ[ppm] = −10.61 (Sn).

**$^{11}$B{$^1$H} NMR (128 MHz, 298 K, THF-d8):** δ[ppm] = −6.47 (B, BArF).

**$^{19}$F{$^1$H} NMR (376 MHz, 298 K, THF-d8):** δ[ppm] = −63.36 (C*F*$_3$, BArF).

**LIFDI-MS** m/z $C_{39}H_{49}N_4O_2Sn$ calcd: 725.2878; m/z found: 725.2868.

**Elemental analysis (%) calcd for $C_{71}H_{61}BF_{24}N_4O_2Sn$:** C 53.71, H 3.87, N 3.53, F 28.72, Sn 7.48; found: C 51.96, H 3.88, N 3.43, F 25.5, Sn 7.7.

m.p.: 128.9 °C.

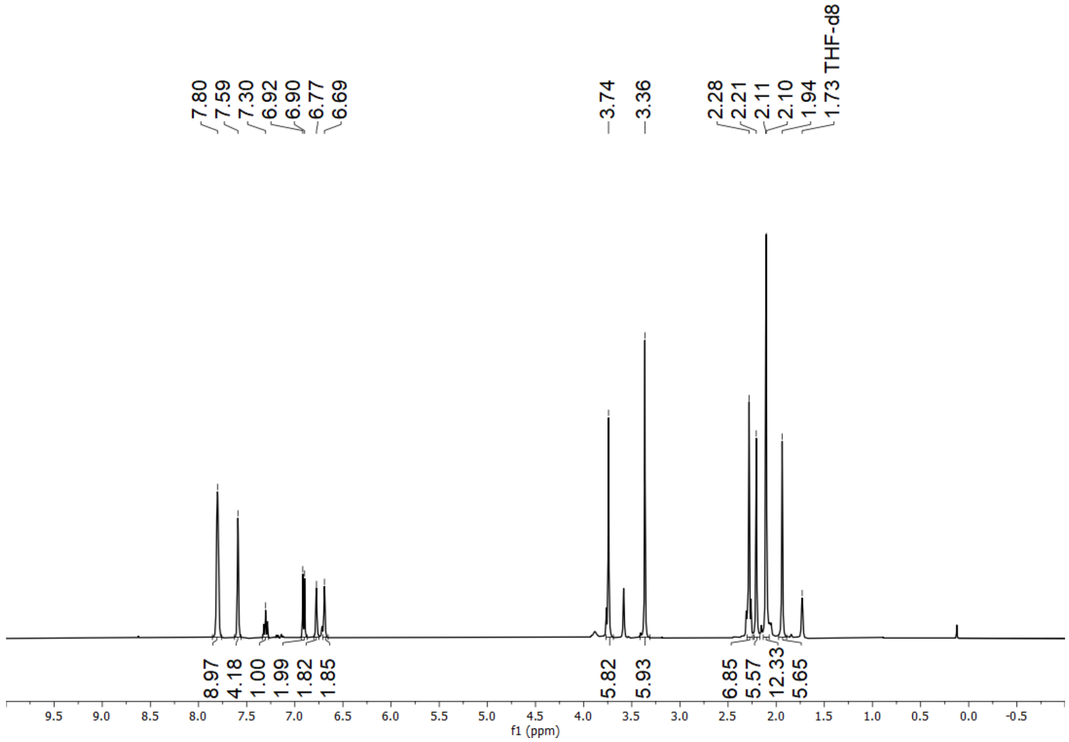


**Figure S13**. $^{1}$H NMR spectrum of [**3**][BArF] in THF-$d_8$ at 298 K.

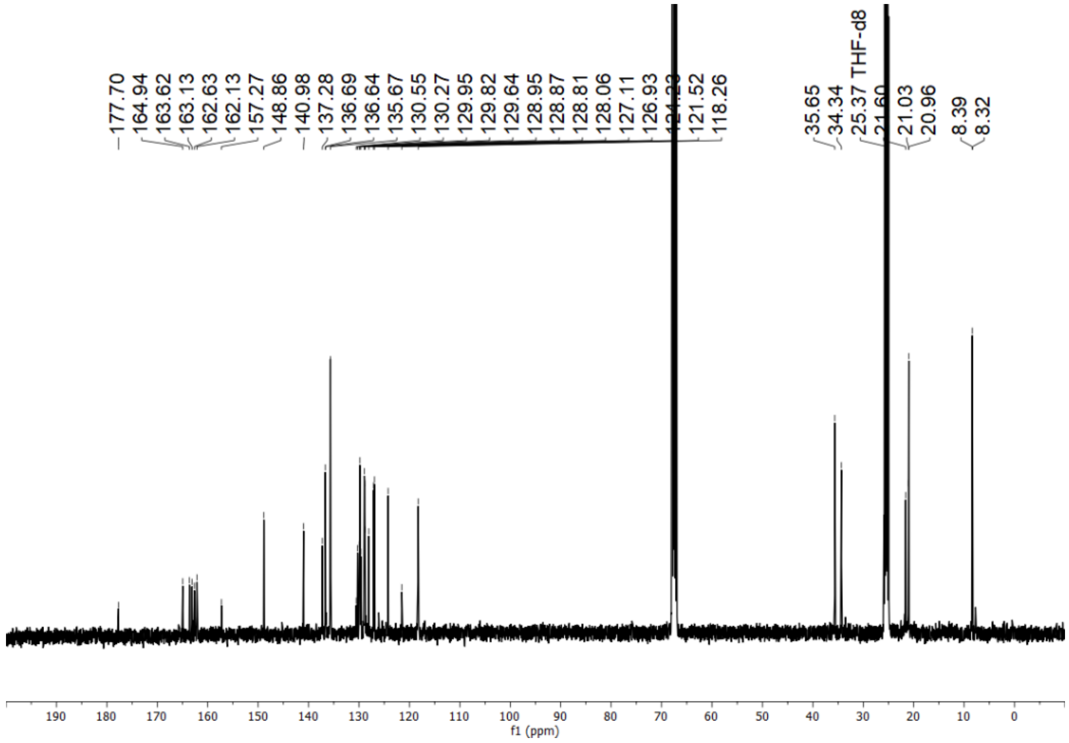


**Figure S14**. $^{13}$C{$^{1}$H} NMR spectrum of [**3**][BArF] in THF-$d_8$ at 298 K.

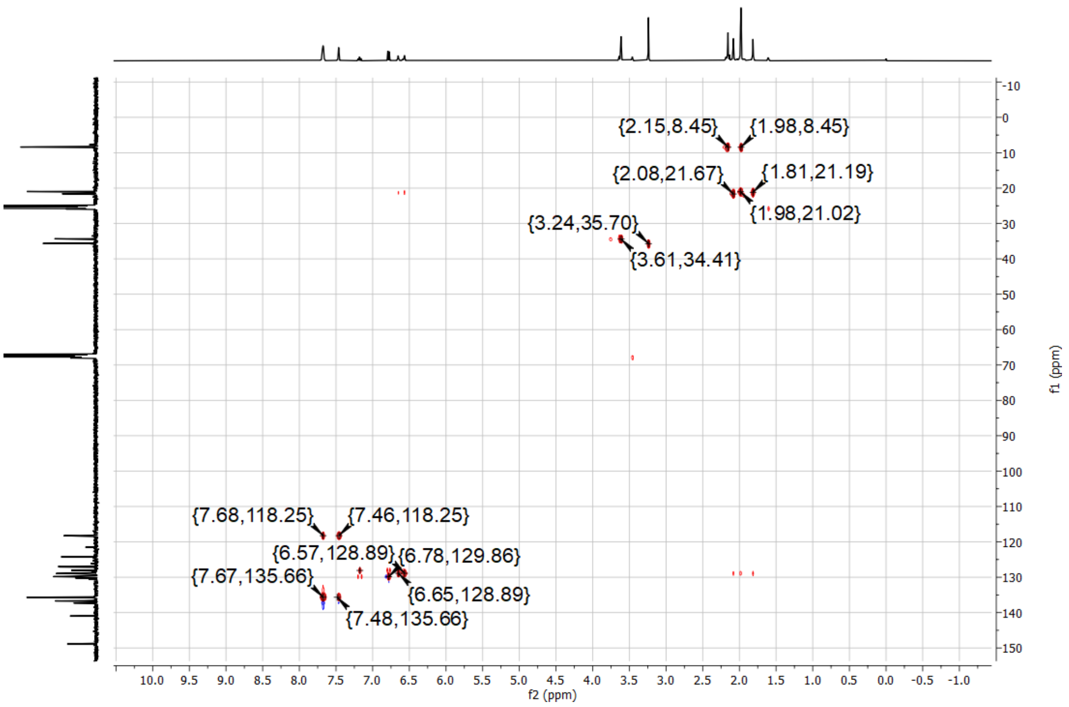


**Figure S15**. $^{1}H/^{13}C$ NMR HSQC spectrum of [**3**][**BArF**] in THF-d8 at 298 K.

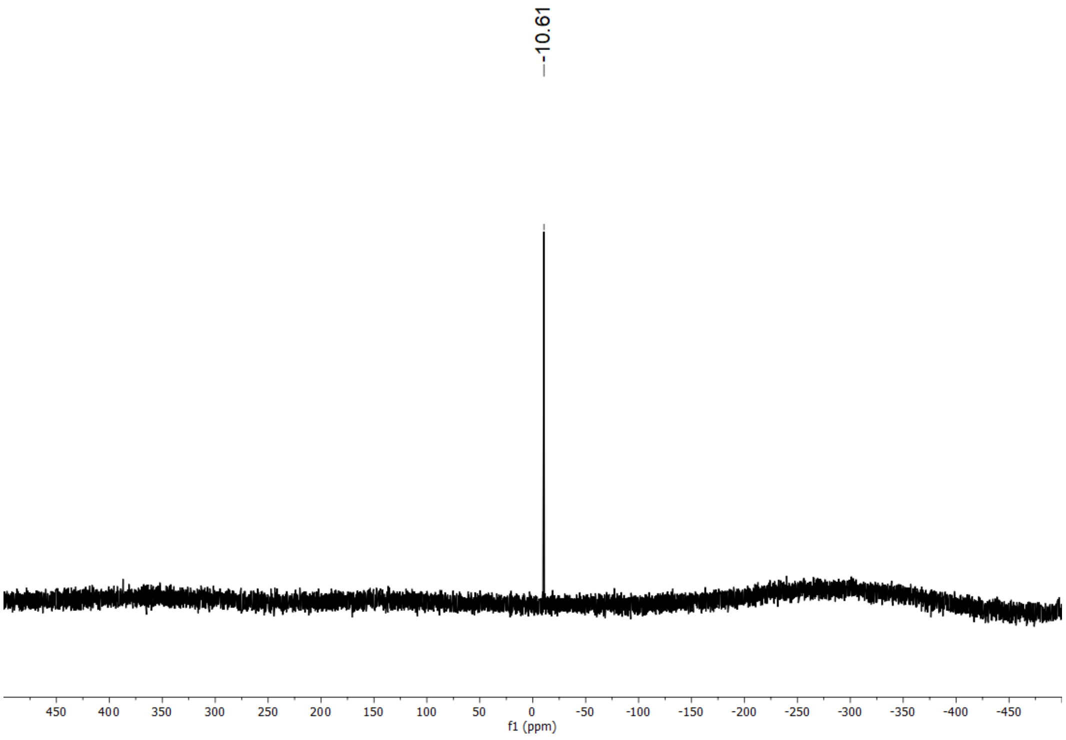


**Figure S16**. $^{119}Sn\{^{1}H\}$ NMR spectrum of [**3**][BArF] in THF-d8 at 298 K.

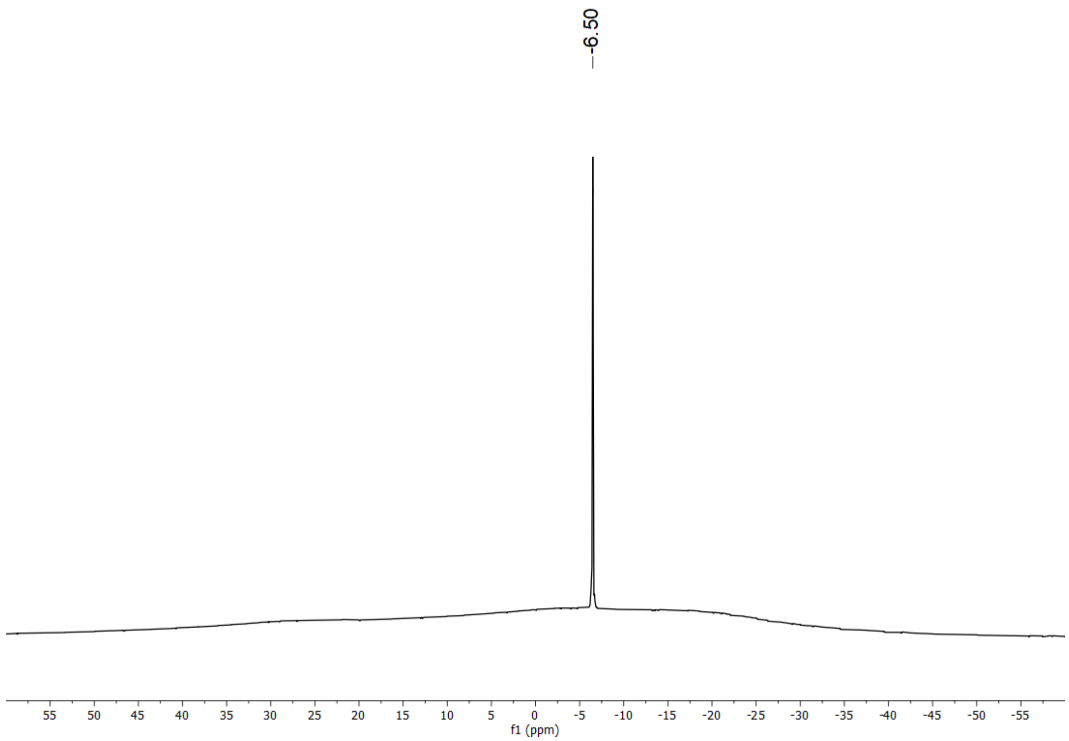


**Figure S17**. $^{11}B\{^1H\}$ NMR spectrum of [**3**][BArF] in THF-$d_8$ at 298 K.

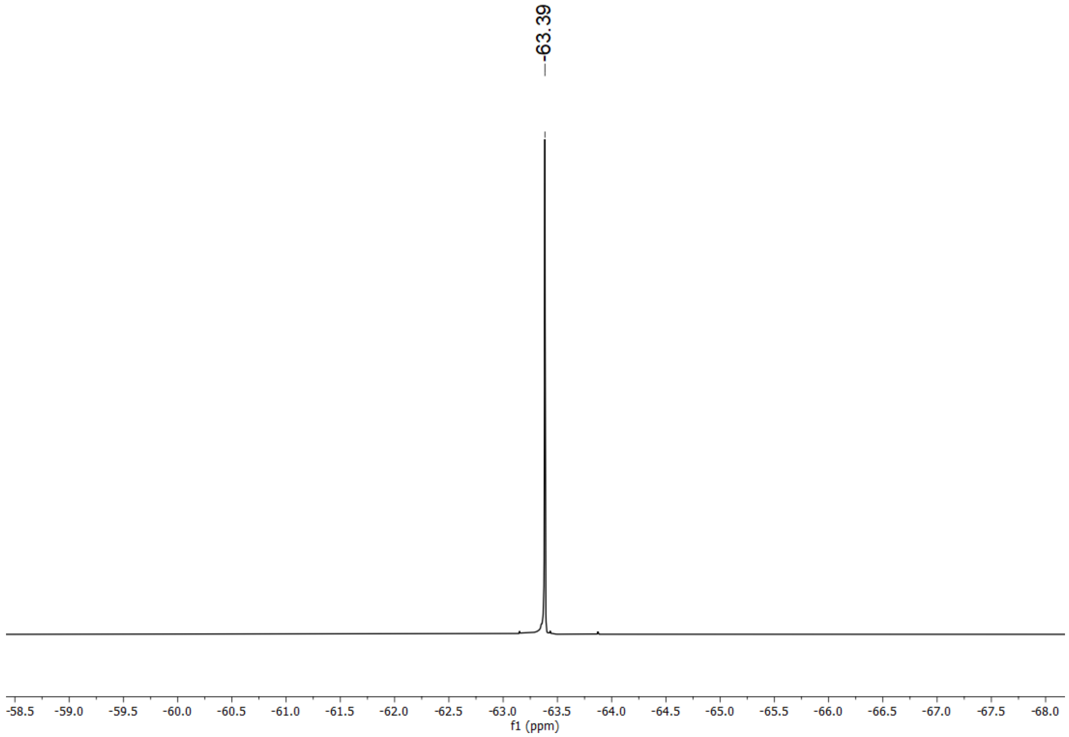


**Figure S18**. $^{19}F\{^1H\}$ NMR spectrum of [**3**][BArF] in THF-$d_8$ at 298 K.

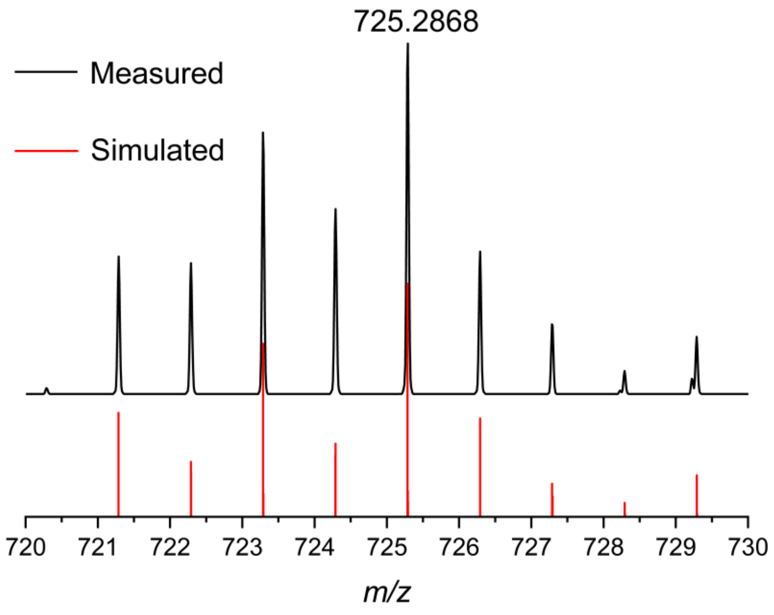


**Figure S19**. LIFDI-MS spectrum (detail view) of [**3**]$^+$ in [**3**][BArF] (measured spectrum: top; simulated spectrum: bottom).

## 1.4 Synthesis of [**4**][BArF]

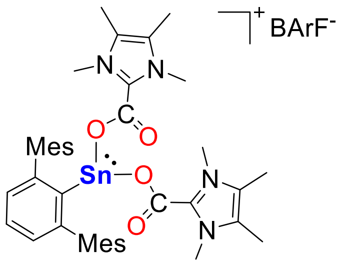


### 1.4.1 NMR Reaction

[**2**][BArF] (16.2 µmol, 25.0 mg) was dissolved in THF-d$_8$ (0.4 mL) in a J-Young NMR tube. The solution was degassed using a freeze-pump-thaw cycle and then pressurized with 1 bar of $CO_2$ at room temperature. The reaction to form [**4**][BArF] was completed after 21 hours, as confirmed by $^1$H, $^{13}$C{$^1$H}, $^{119}$Sn{$^1$H}, $^{11}$B{$^1$H}, and $^{19}$F{$^1$H} NMR spectroscopys.

### 1.4.2 Crystallisation of [**4**][BArF]

[**2**][BArF] (32.4 µmol, 50.0 mg) was dissolved in $Et_2O$ (1 mL) and pentane (2 mL). The solution was degassed freeze-pump-thaw cycle, followed by the addition of 1 bar of $CO_2$ at room temperature. After stirring for 24 hours, the solution's color changed from colorless to light yellow. The reaction solution was then stored at −35 °C under the $CO_2$ atmosphere. After an additional 24 hours, light-yellow crystals suitable for single-crystal X-ray analysis were obtained.

*Note: It was not possible to obtain solid samples of pure [**4**][BArF] in large quantities due to decomposition when placed under vacuum.*

**$^{1}$H NMR (400 MHz, 298 K, THF-$d_8$)**: δ[ppm] = 2.07 (s, 12 H, 2x$C^{2,6}$-C$H_3$, Mes), 2.11 (s, 6 H, 2x$C^{4}$-C$H_3$, Mes), 2.30 (s, 12 H, 4xC-C$H_3$, NHC), 3.92 (s, 12 H, 4xN-C$H_3$, NHC), 6.70 (s, 4 H, 2x$C^{3,5}$-*H*, Mes), 6.87 (d, $^{3}J_{H-H}$ = 8 Hz, 2 H, $C^{3,5}$ -*H*, $C_6H_3$), 7.29 (t, $^{3}J_{H-H}$ = 8 Hz, 1 H, $C^{4}$-*H*, $C_6H_3$), 7.60, 7.81 (s, 12 H, 4x$C^{2,4,6}$, 4$C_6H_3(CF_3)_2$).

**$^{13}$C{$^{1}$H} NMR (101 MHz, 298 K, THF-$d_8$):** δ[ppm] = 8.36 (2x$C^{4,5}$-*C*$H_3$, NHC), 21.14 ($C^{4}$-*C*$H_3$, Mes), 21.88 ($C^{2,6}$-*C*$H_3$, Mes), 34.37 (4xN-*C*$H_3$, NHC), 118.27 (*p*-$CH_{B-Ar}$), 121.54, 124.24, 126.95 (*C*, BArF), 125.94 (*C*$O_2$ (aq) ), 128.05 (4x$C^{3,5}$-H, Mes), 128.67 ($C^{4}$-H, $C_6H_3$), 129.04 ($C^{3,5}$-H, $C_6H_3$), 129.11 ($C^{4,5}$-$CH_3$, NHC), 129.65-130.59 (m-$C_{B-Ar}$), 135.68 (o-$CH_{B-Ar}$), 136.41 (2x$C^{4}$-Mes), 136.94 ($N_2$*C*, NHC), 137.77 (2x$C^{1}$-Mes), 141.74 (2x$C^{2,6}$-Mes), 149.45 (2x$C^{2,6}$-$C_6H_3$), 157.48 (C*C*$O_2$), 159.66-163.62 (ipso-CB-Ar), 172.98 (Sn-*C*, $C_6H_3$).

**$^{119}$Sn{$^{1}$H} NMR (149 MHz, 298 K, THF-$d_8$):** δ[ppm] = 46.41 (Sn).

**$^{11}$B{$^{1}$H} NMR (128 MHz, 298 K, THF-$d_8$):** δ[ppm] = −6.47 (B, BArF).

**$^{19}$F{$^{1}$H} NMR (376 MHz, 298 K, THF-$d_8$):** δ[ppm] = −63.36 (C*F*$_3$, BArF).

**LIFDI-MS** m/z $C_{40}H_{49}N_4O_4Sn$ calcd: 769.2776; *m/z* found: 769.2777.

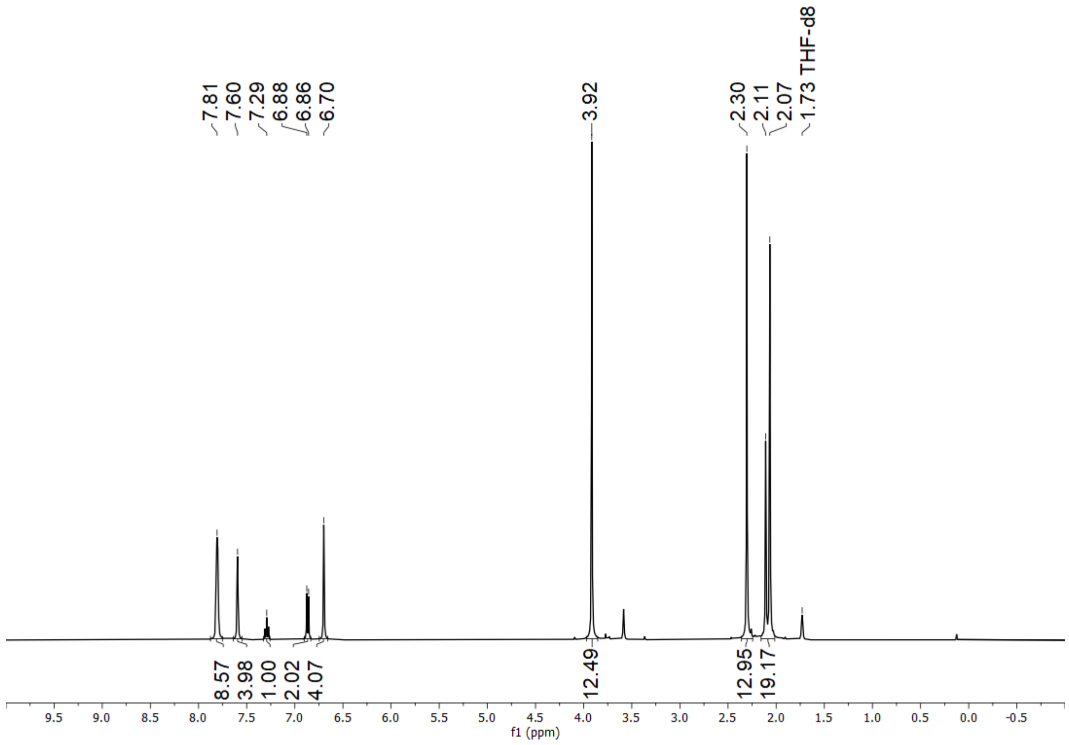


**Figure S20**. $^{1}$H NMR spectrum of [**4**][BArF] in THF-$d_8$ at 298 K.

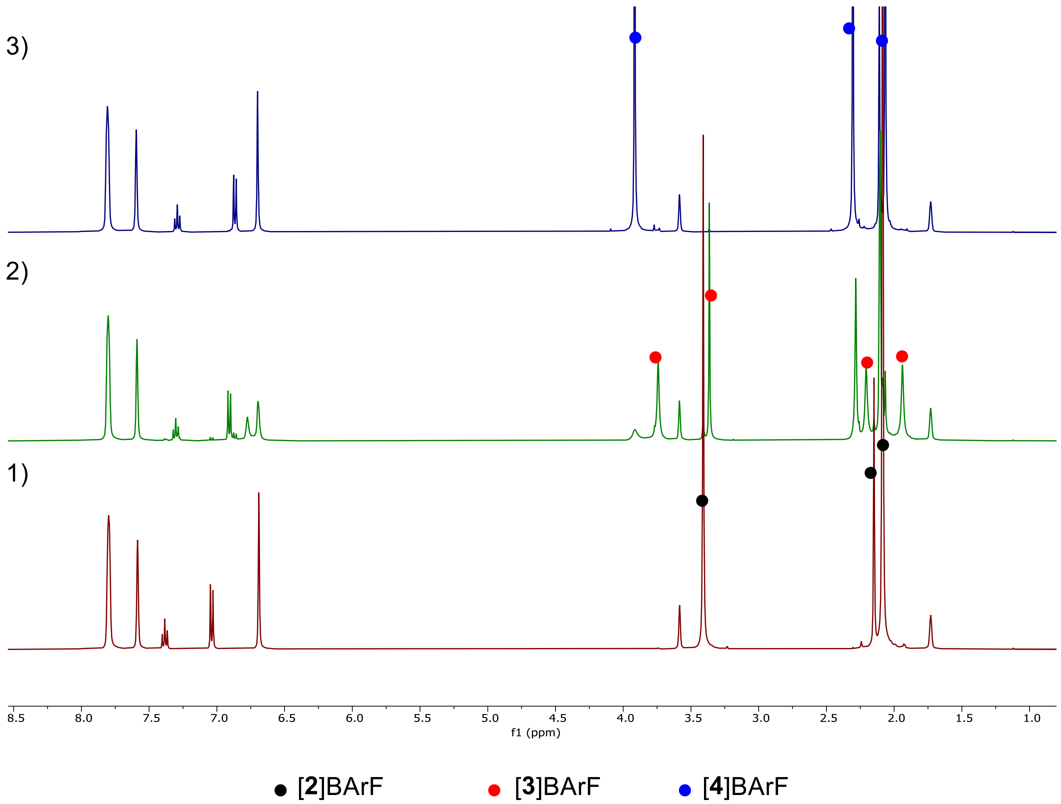


**Figure S21**. Stacked ¹H NMR spectra of the reaction of [**2**][BArF] with $CO_2$ (1 bar): 1) [**2**][BArF]; 2) [**2**][BArF] + $CO_2$, RT 6 hours; 3) [**2**][BArF] + $CO_2$, RT 21 hours.

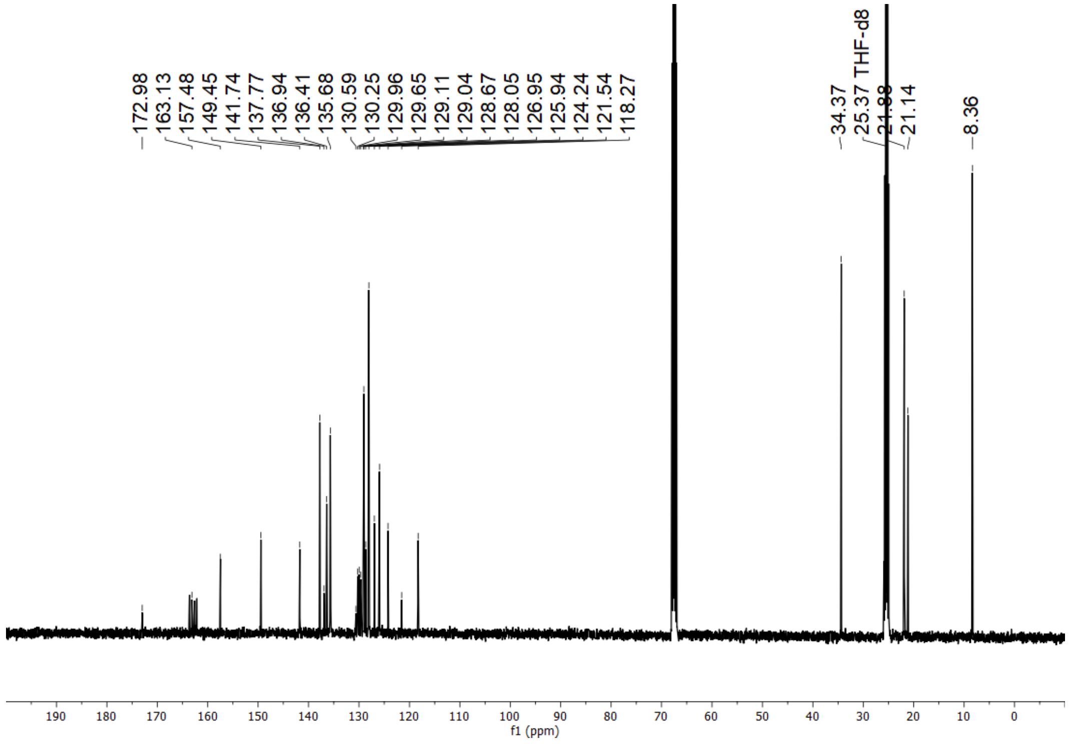

**Figure S22**. $^{13}C\{^{1}H\}$ NMR spectrum of [**4**][BArF] in THF-$d_8$ at 298 K.

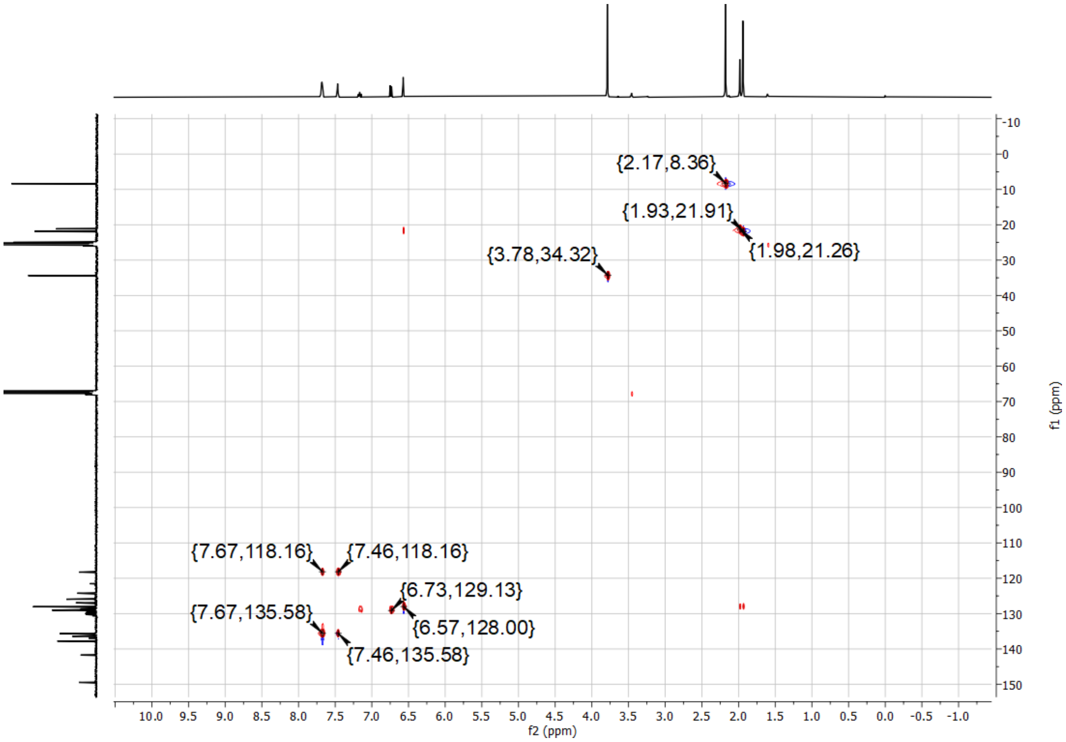


**Figure S23**. $^{1}H/^{13}C$ NMR HSQC spectrum of [**4**][BArF] in THF-$d_8$ at 298 K.

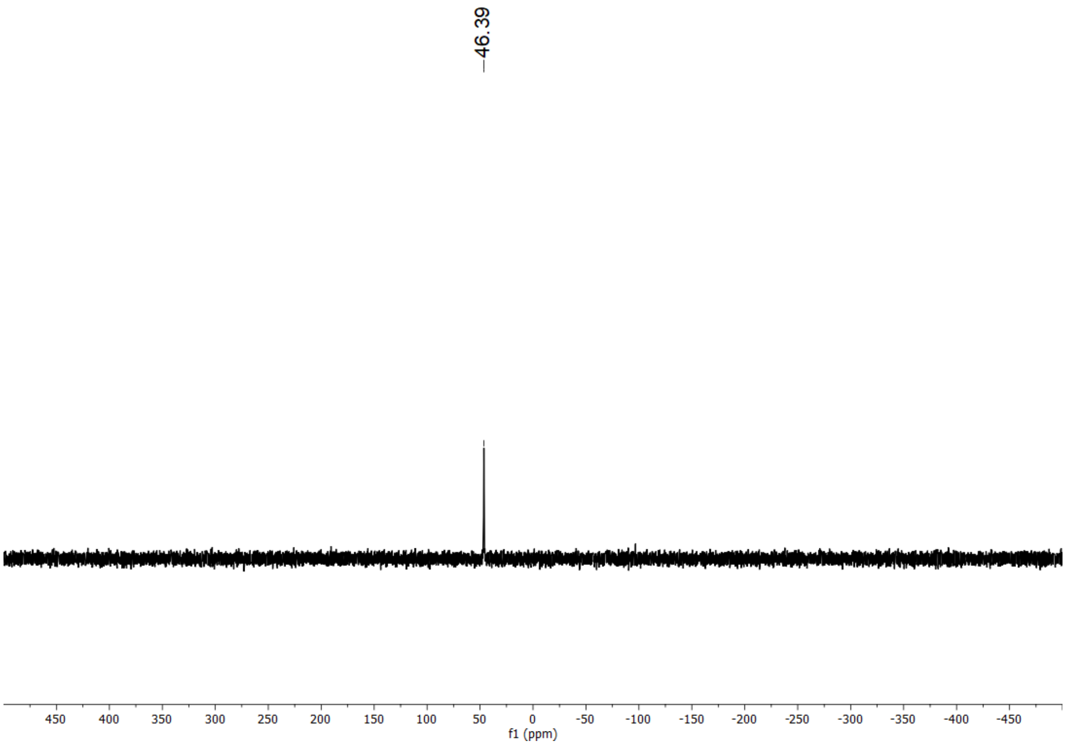


**Figure S24**. $^{119}Sn\{^{1}H\}$ NMR spectrum of [**4**][BArF] in THF-$d_8$ at 298 K.

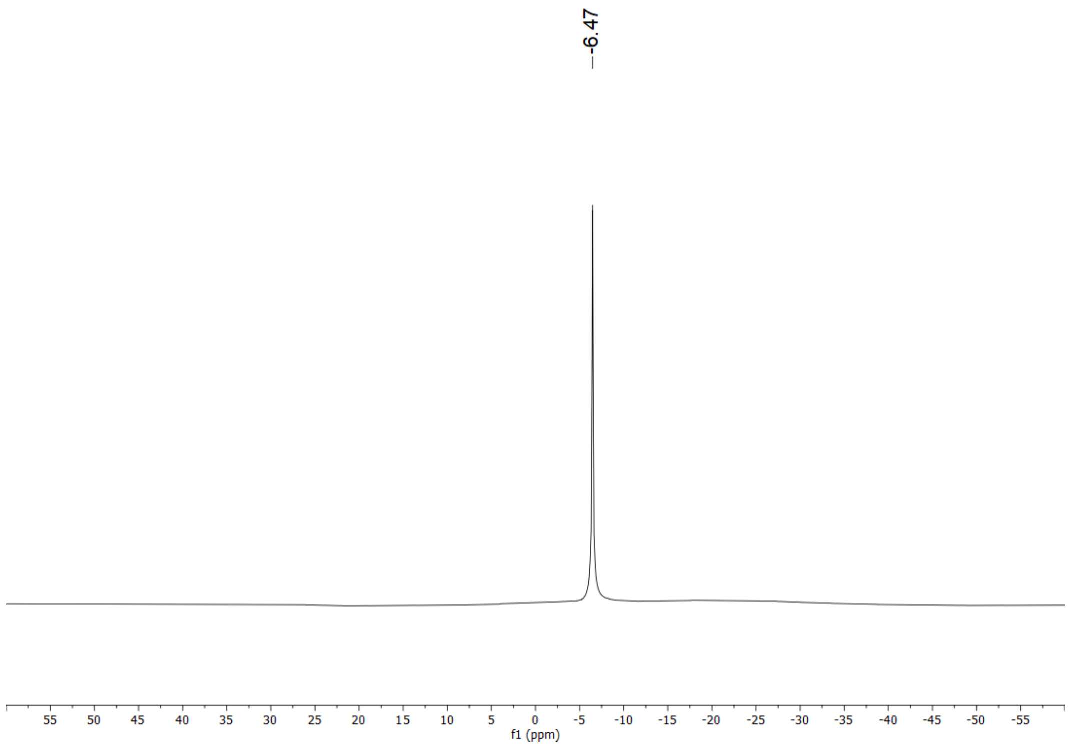


**Figure S25**. $^{11}B\{^1H\}$ NMR spectrum of [**4**][BArF] in THF-$d_8$ at 298 K.

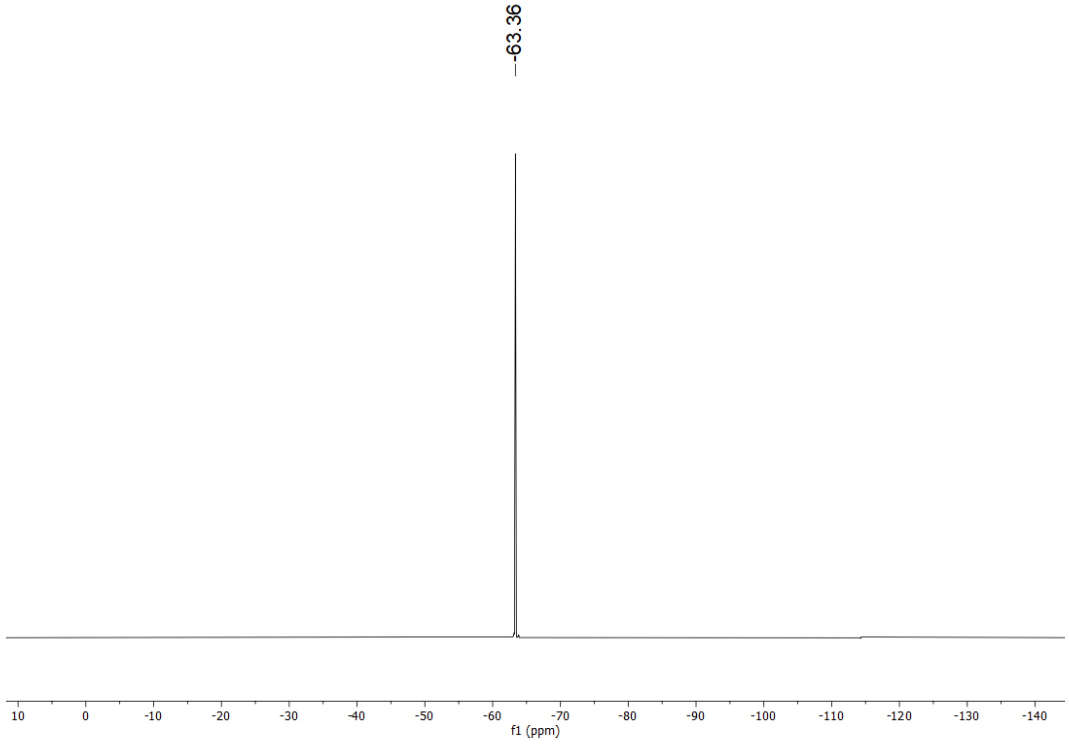


**Figure S26**. $^{19}F\{^1H\}$ NMR spectrum of [**4**][BArF] in THF-$d_8$ at 298 K.

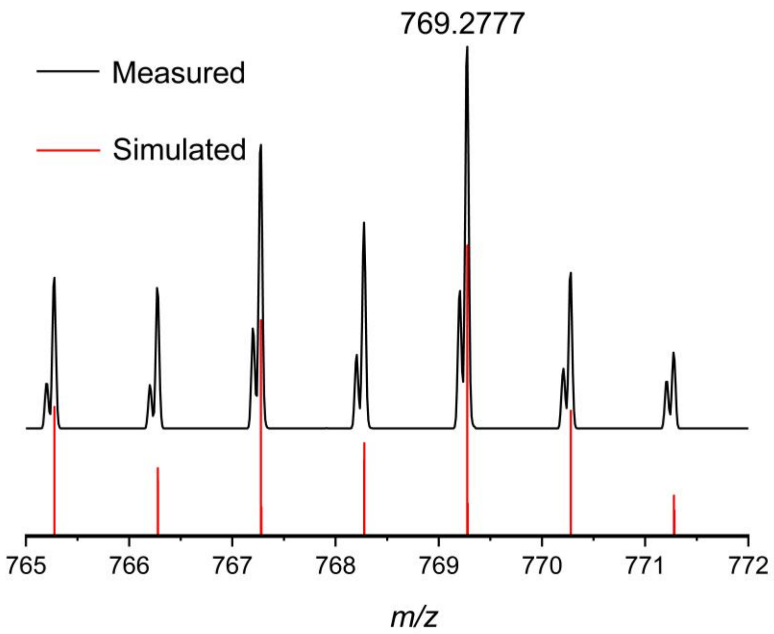


**Figure S27**. LIFDI-MS spectrum (detail view) of [**4**]$^+$ in [**4**][BArF] (measured spectrum: top; simulated spectrum: bottom).

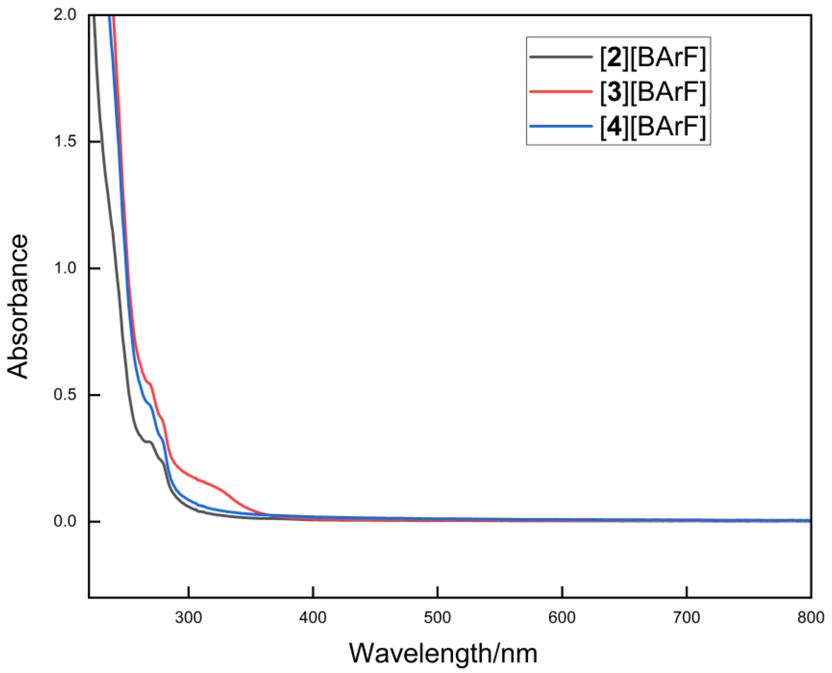


**Figure S28**. UV-vis spectrums of [**2**][BArF], [**3**][BArF], and [**4**][BArF] (r.t., THF, concentration: 3.125x10$^{-5}$ M). No abstraction signal is observed in the UV spectra of [**2**][BArF], [**3**][BArF], and [**4**][BArF].

1.5 NMR Experiments

1.5.1 The thermal stability of [**4**][BArF]

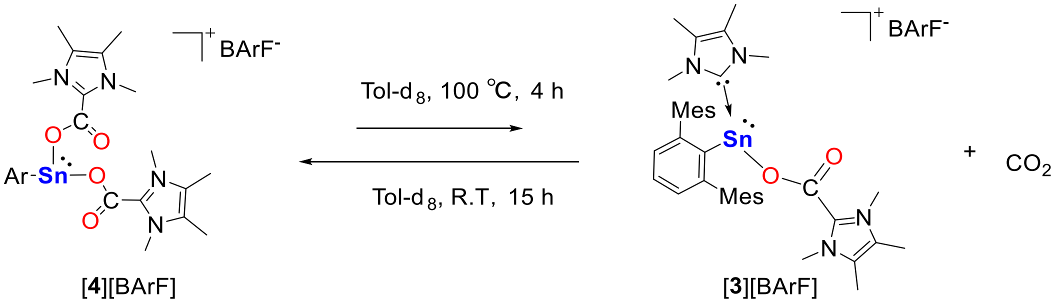


To test the reversibility of [**4**][BArF], a solution of [**4**][BArF] in toluene-d$_8$ (0.2 mL) and fluorobenzene (0.2 mL) was heated to 100 ºC under $CO_2$ or argon atmosphere in a J-Young NMR tube and monitored by $^1$H NMR.

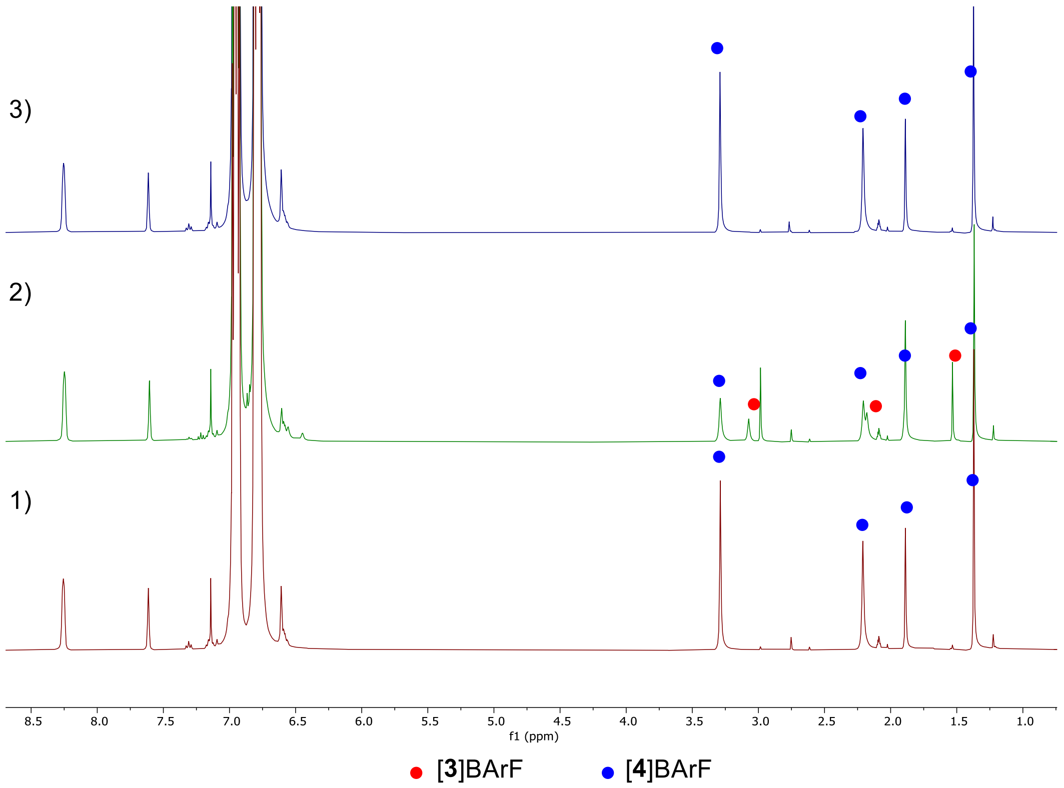


**Figure S29**. Stacked $^{1}$H NMR spectra showing reversibility of [**4**][BArF] under $CO_2$ atmosphere. 1) [**4**][BArF]; 2) [**4**][BArF] heating 4 hours at 100 °C; 3) cool to rt for 15 hours.

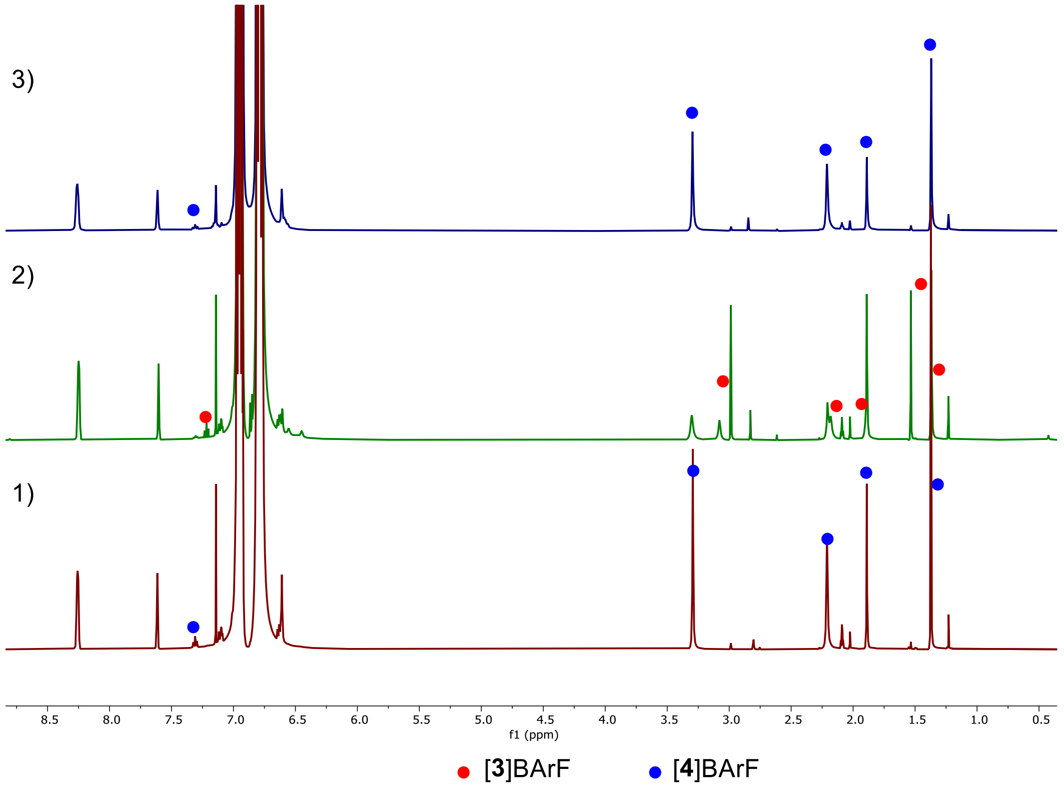

**Figure S30**. Stacked $^{1}H$ NMR spectra showing reversibility of [**4**][BArF] under argon atmosphere. 1) [**4**][BArF]; 2) [**4**][BArF] heating 4 hours at 100 °C; 3) cool to room temperature for 15 hours.

### 1.5.2 [**4**][BarF] reaction with $Ph_2SiH_2$

[**2**][BArF] (1.00 eq, 16.2 µmol, 25.0 mg) was dissolved in $C_6D_6$ (0.2 mL) and fluorobenzene (0.2 mL) in a J-Young NMR tube. The solution was then freeze-pump-thaw degassed and was then pressurised with 1 bar of $CO_2$ at room temperature. After 21 hours (when [**4**][BArF] was completely formed) the excess $CO_2$ was removed and $H_2SiPh_2$ (1.00 eq, 16.2 µmol, 2.43 µL) was added. The reaction progress was monitored by $^{1}H$ NMR spectroscopy.

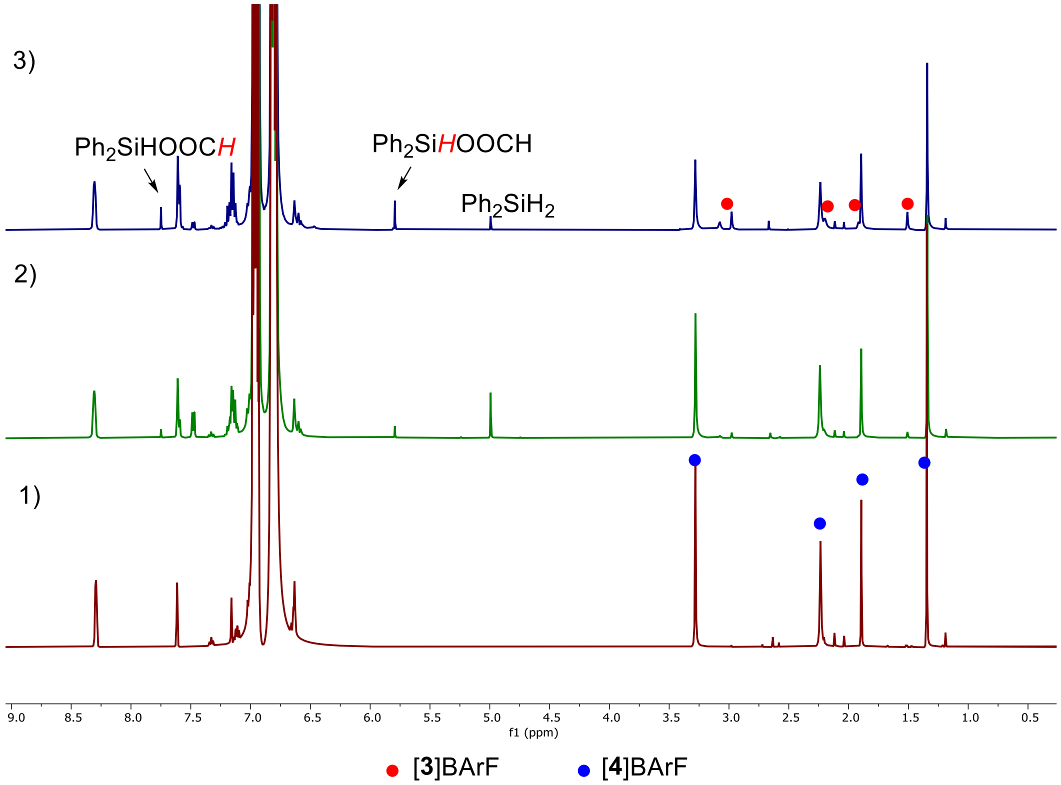


**Figure S31.** Stackthe$^{1}H$ NMR spectra showing [**4**][BArF] reaction with $Ph_2SiH_2$: 1) the formation of [**4**][BArF]; 2) [**4**][BArF] + $H_2SiPh_2$, RT 1 hour; 3) [**4**][BArF] + $H_2SiPh_2$, RT 5 hours.

## 1.6 Catalytic Experiments

**General Procedure for Hydrosilylation of $CO_2$:** Silane (80.0 µmol, 14.9 µL), 1,3,5-trimethoxybenzene (0.10 eq of the silane, as an internal standard),* and catalyst were dissolved in a mixture of $C_6D_6/C_6H_5F$ in a J-Young NMR tube under an argon atmosphere. An initial $^{1}H$ NMR spectrum was measured to determine the concentration of silane (by comparison to the internal standard). Subsequently, the solution was rapidly degassed using a Schlenk line and then refilled with 1 bar of $CO_2$. The progression of reaction was monitored via $^{1}H$ NMR spectroscopy by the consumption of silane alongside the

emergence of the respective hydrosilylation products resonances. After 25 hours, the conversion of silane was determined by comparing integrals of the unreacted silane resonances to the integrals of the internal standard. The yield was determined by comparing integrals of the $R_2Si\mathbf{H}(OCHO)$ or $R_3Si(OC\mathbf{H}O)$ resonances of the hydrosilylation product to the integral of the internal standard. The summary of the results was shown in **Table S1**.

* = 1,3,5-trimethoxybenzene was seleted as the internal standard, setting its concentration to 10 mol% of the added silane. This equivalent exhibits a strong signal-to-noise ratio, and the signal of internal standard is comparable to that of silane.

**Mercury Test Experiment**

[**2**][BArF] (5 mol%, 4.00 µmol, 80.0 µL, 0.05 M in $C_6H_5F$), $H_2SiPh_2$ (80.0 µmol, 14.9 µL), and 1,3,5-trimethoxybenzene (0.10 eq of the $Ph_2SiH_2$) as an internal standard were dissolved in $C_6D_6$ (0.32 mL) in a J-Young NMR tube. Then mercury (13.5 mg) was added to the solution. The solution was rapidly degassed using a Schlenk line and then refilled with 1 bar of $CO_2$ at room temperature. The progression of reaction was monitored via $^1$H NMR spectroscopy. The results are shown in Table S1 (Entry 11).

**Catalytic reduction of $CO_2$ with $Ph_2SiH_2$ by [4][BArF]**

[**2**][BArF] (5 mol%, 4.00 µmol, 80.0 µL, 0.05 M in $C_6H_5F$) and 1,3,5-trimethoxybenzene (0.10 eq, 8.0 µmol) were introduced into 0.32 mL of $C_6D_6$ within a J-Young NMR tube. The solution was degassed and refilled with 1 bar of $CO_2$ at room temperature. The solution of [**4**][BArF] was obtained after 21 hours and excess $CO_2$ was removed, then $H_2SiPh_2$ (1.0 eq, 80.0 µmol, 14.9 µL) was added in gloves box. The solution was degassed again and then pressurised with 1 bar of $CO_2$ at room temperature. The reaction was monitored via $^1$H NMR spectroscopy.

**Table S1.** Hydrosilylation of $CO_2$

| Entry | Catalysis (mol%) | Solvent | T /℃ | Silane | Conversion/% (time) | NMR Yield/% [$R_3Si(OCHO)$] | TOF /$h^{-1}$ | $^1H$ NMR Number |
|---|---|---|---|---|---|---|---|---|
| 1 | [**2**][BArF] (0.5) | $C_6D_6$:$C_6H_5F$ (4:1) | 25 | $H_2SiPh_2$ | 4 | <1 | 0.35 | Figure S32 |
| 2 | [**2**][BArF] (1) | $C_6D_6$:$C_6H_5F$ (4:1) | 25 | $H_2SiPh_2$ | 11 | 4 | 0.45 | Figure S33 |
| 3 | [**2**][BArF] (2.5) | $C_6D_6$:$C_6H_5F$ (4:1) | 25 | $H_2SiPh_2$ | 74 | 63 | 1.18 | Figure S34 |
| 4 | [**2**][BArF] (5) | $C_6D_6$:$C_6H_5F$ (4:1) | 25 | $H_2SiPh_2$ | 94 | 81 | 0.75 | Figure S35 |
| 5 | [**2**][BArF] (5) | $C_6D_6$ | 25 | $H_2SiPh_2$ | 78 | 71 | 0.62 | Figure S36 |
| 6 | [**2**][BArF] (5) | THF-$d_8$ | 25 | $H_2SiPh_2$ | 75 | 54 | 0.60 | Figure S37 |
| 7 | [**2**][BArF] (5) | $C_6D_6$:$C_6H_4F_2$ (4:1) | 25 | $H_2SiPh_2$ | 97 | 77 | 0.77 | Figure S38 |
| 8 | [**2**][BArF] (5) | $C_6D_6$:$C_6H_4F_2$ (1:1) | 25 | $H_2SiPh_2$ | 94 | 81 | 0.75 | Figure S39 |
| 9 | [**2**][BArF] (5) | $C_6D_6$:$C_6H_5F$ (4:1) | 50 | $H_2SiPh_2$ | 64 (2 hours) | 64 | 0.79 | Figure S40 |
| 10 | [**2**][$Al(OC(CF_3)_3)_4$] (5) | $C_6D_6$:$C_6H_5F$ (4:1) | 25 | $H_2SiPh_2$ | 70 | 60 | 0.56 | Figure S41 |
| 11 | [**2**][BArF] (5)+Hg | $C_6D_6$:$C_6H_5F$ (4:1) | 25 | $H_2SiPh_2$ | 89 | 69 | 0.71 | Figure S42 |
| 12 | [**3**][BArF] (5) | $C_6D_6$:$C_6H_5F$ (4:1) | 25 | $H_2SiPh_2$ | 92 | 83 | 0.74 | Figure S43 |
| 13 | [**4**][BArF] (5) | $C_6D_6$:$C_6H_5F$ (4:1) | 25 | $H_2SiPh_2$ | 93 | 93 | 0.74 | Figure S44 |
| 14 | $IMe_4$ (10) | $C_6D_6$:$C_6H_5F$ (4:1) | 25 | $H_2SiPh_2$ | 51 | < 1 | 0.39 | Figure S45 |
| 15 | $IMe_4$-$CO_2$ (10) | $C_6D_6$:$C_6H_4F_2$ (1:1) | 25 | $H_2SiPh_2$ | 51 | 9 | 0.41 | Figure S46 |
| 16 | [**2**][BArF] (5) | $C_6D_6$:$C_6H_5F$ (4:1) | 25 | $Me_2PhSiH$ | 47 | 46 | 0.37 | Figure S47 |
| 17 | [**2**][BArF] (5) | $C_6D_6$:$C_6H_5F$ (4:1) | 25 | $H_3SiPh$ | 48 (2 hours)<br>71 (12 hours) | 39<br><1 | 4.8<br>1.18 | Figure S48 |

| | | | | | | | | |
|---|---|---|---|---|---|---|---|---|
| 18 | [**2**][BArF] (5) | $C_6D_6$:$C_6H_5F$ (4:1) | 60 | $HSiEt_3$ | 29 | 27 | 0.23 | Figure S49 |
| 19 | [**2**][BArF] (5) | $C_6D_6$:$C_6H_5F$ (4:1) | 80 | $HSiPh_3$ | No reaction | - | - | Figure S50 |

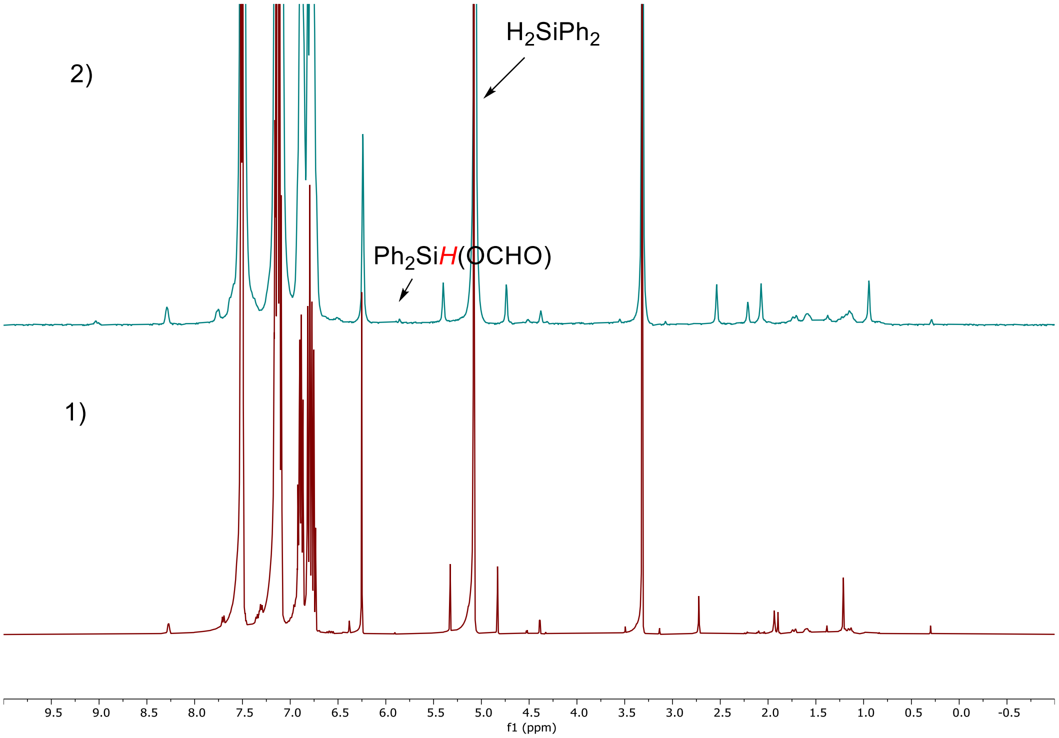


**Figure S32**. Stacked $^{1}$H NMR spectra of hydrosilylation of $CO_2$ with $H_2SiPh_2$ by [**2**][BArF] (0.5 mol%) in $C_6D_6$/$C_6H_5F$ (4:1): 1) [**2**][BArF] (0.5 mol%) + $H_2SiPh_2$; 2) [**2**][BArF] (0.5 mol%) + $H_2SiPh_2$ + $CO_2$, RT 25 hours.

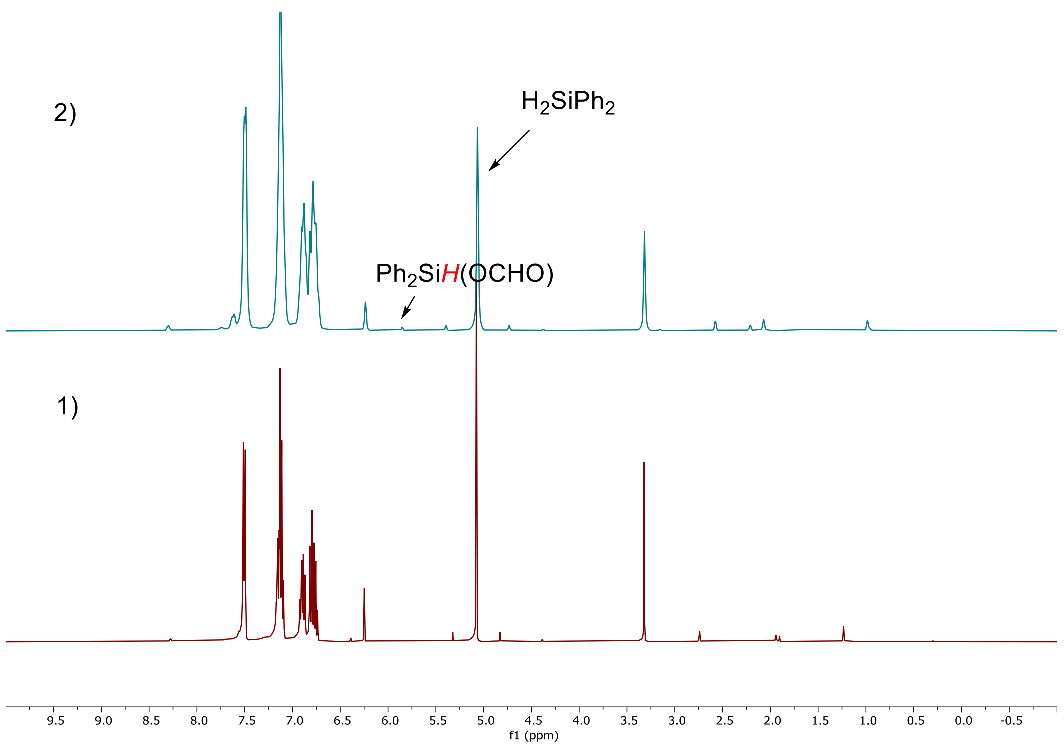


**Figure S33.** Stacked $^{1}$H NMR spectra of hydrosilylation of $CO_2$ with $H_2SiPh_2$ by [**2**][BArF] (1 mol%) in $C_6D_6/C_6H_5F$ (4:1): 1) [**2**][BArF] (1 mol%) + $H_2SiPh_2$; 2) [**2**][BArF] (1 mol%) + $H_2SiPh_2$ + $CO_2$, RT 25 hours.

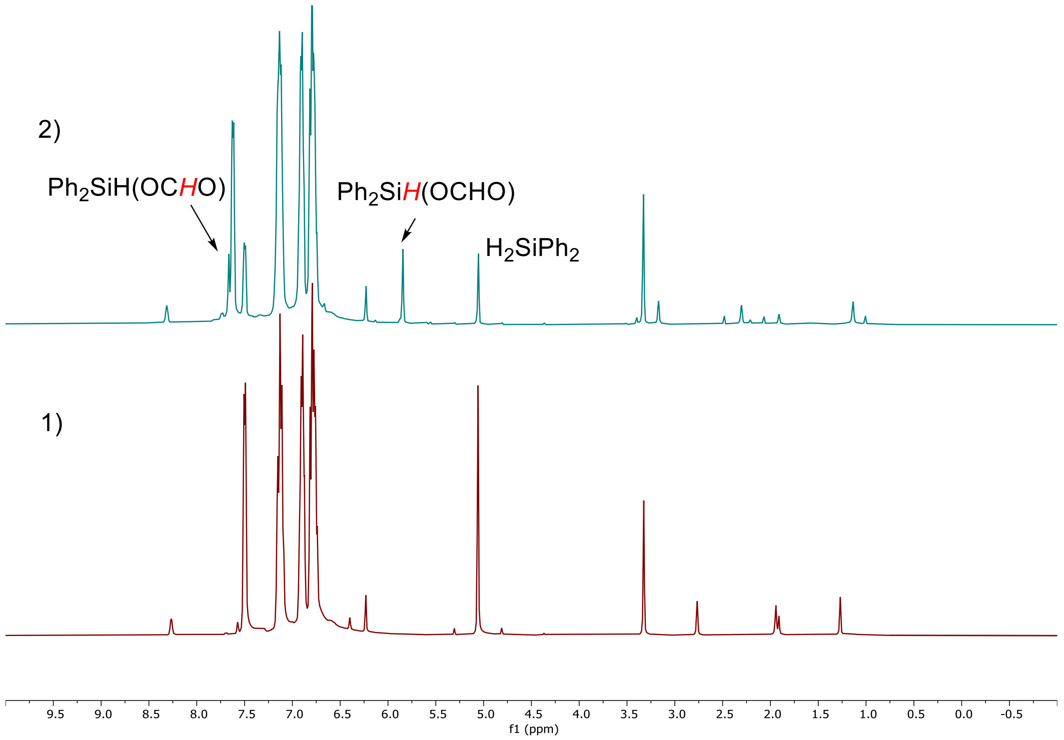

**Figure S34.** Stacked $^{1}$H NMR spectra of hydrosilylation of $CO_2$ with $H_2SiPh_2$ by [**2**][BArF] (2.5 mol%) in $C_6D_6$/$C_6H_5F$ (4:1): 1) [**2**][BArF] (2.5 mol%) + $H_2SiPh_2$; 2) [**2**][BArF] (2.5 mol%) + $H_2SiPh_2$ + $CO_2$, RT 25 hours.

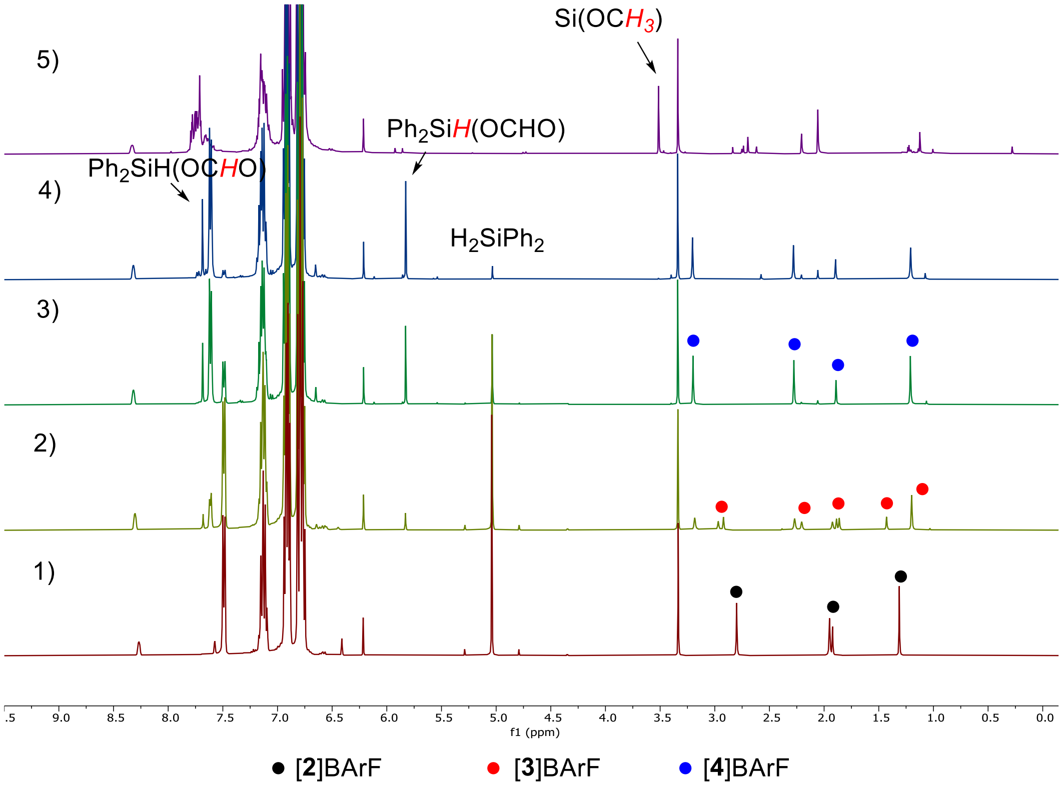


**Figure S35**. Stacked $^{1}$H NMR spectra of hydrosilylation of $CO_2$ with $H_2SiPh_2$ by [**2**][BArF]: 1) [**2**][BArF](5 mol%) + $H_2SiPh_2$; 2) [**2**][BArF] (5 mol%) + $H_2SiPh_2$ + $CO_2$, RT 2 hours; 3) [**2**][BArF] (5 mol%) + $H_2SiPh_2$ + $CO_2$, RT 12 hours; 4) [**2**][BArF] (5 mol%) + $H_2SiPh_2$ + $CO_2$, RT 25 hours; [**2**][BArF] (5 mol%) + $H_2SiPh_2$ + $CO_2$, RT 72 hours.

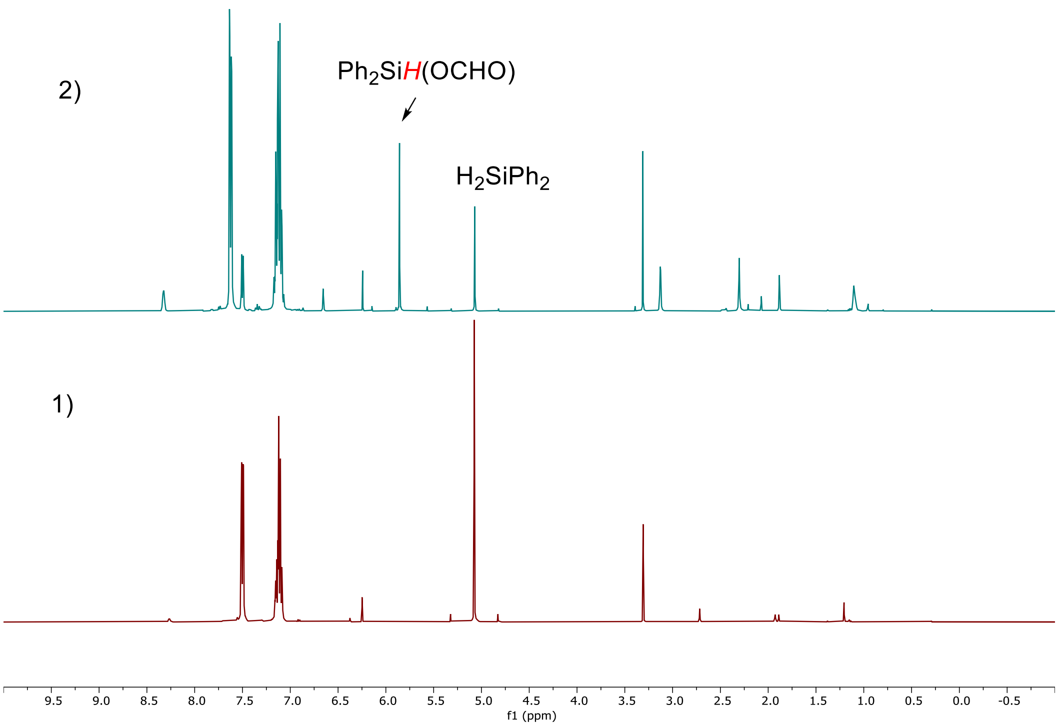


**Figure S36**. Stacked $^{1}$H NMR spectra of hydrosilylation of $CO_2$ with $H_2SiPh_2$ by [**2**][BArF] in $C_6D_6$: 1) [**2**][BArF] (5 mol%) + $H_2SiPh_2$; 2) [**2**][BArF] (5 mol%) + $H_2SiPh_2$ + $CO_2$, RT 25 hours.

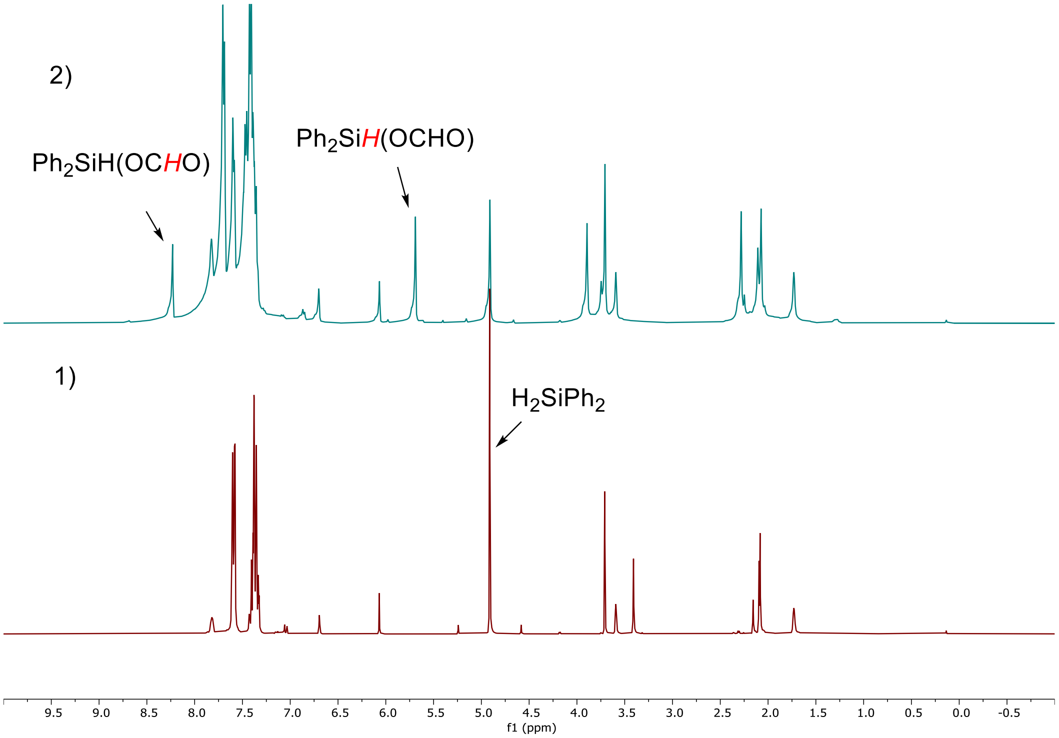

**Figure S37**. Stacked $^{1}$H NMR spectra of hydrosilylation of $CO_2$ with $H_2SiPh_2$ by [**2**][BArF] in THF-$d_8$: 1) [**2**][BArF] (5 mol%) + $H_2SiPh_2$; 2) [**2**][BArF] (5 mol%) + $H_2SiPh_2$ + $CO_2$, RT 25 hours.

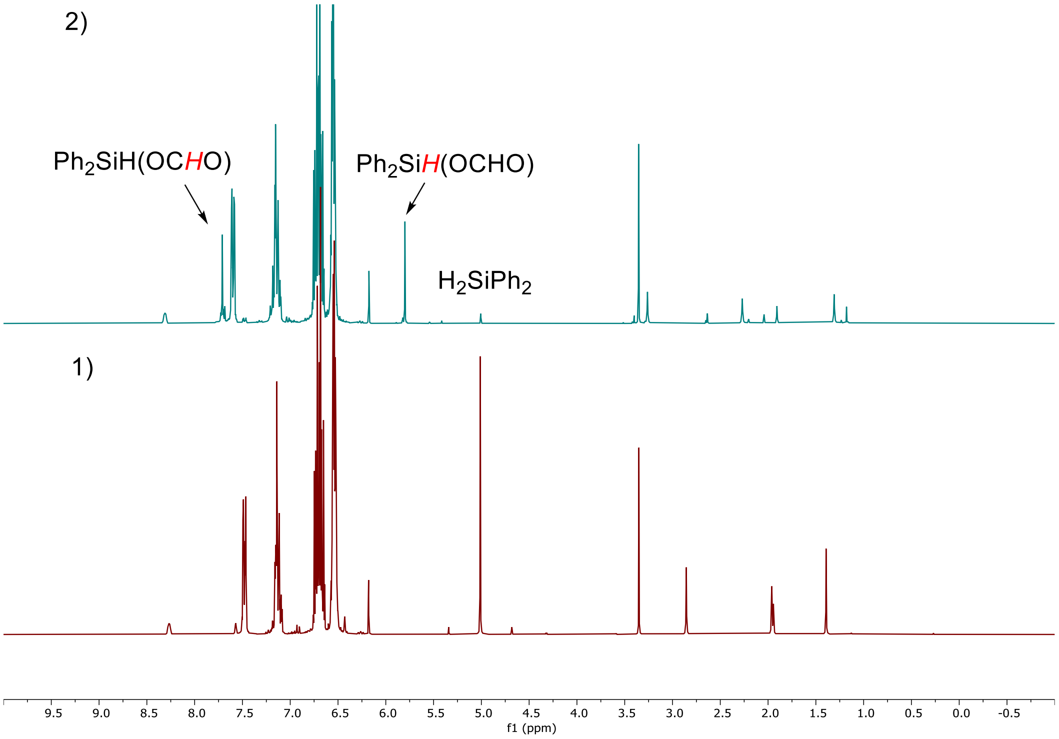


**Figure S38**. Stacked $^{1}$H NMR spectra of hydrosilylation of $CO_2$ with $H_2SiPh_2$ by [**2**][BArF] in $C_6D_6$/$C_6H_4F_2$ (4:1): 1) [**2**][BArF] (5 mol%) + $H_2SiPh_2$; 2) [**2**][BArF] (5 mol%) + $H_2SiPh_2$ + $CO_2$, RT 25 hours.

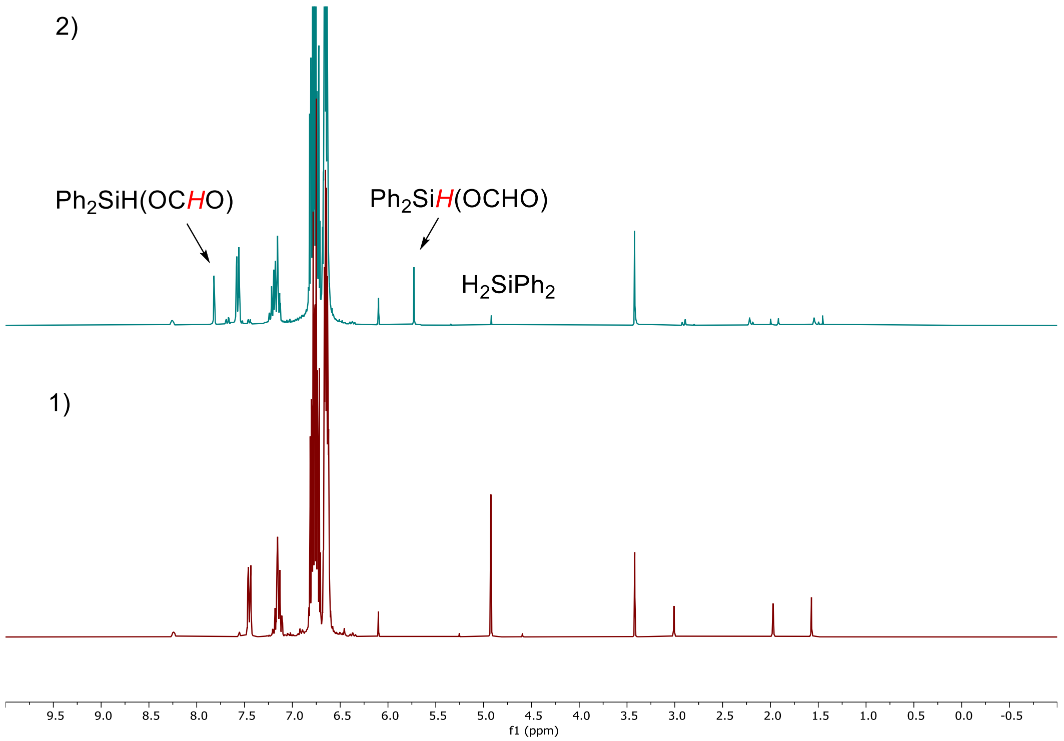


**Figure S39**. Stacked $^{1}$H NMR spectra of hydrosilylation of $CO_2$ with $H_2SiPh_2$ by [**2**][BArF] in $C_6D_6/C_6H_4F_2$ (1:1): 1) [**2**][BArF] (5 mol%) + $H_2SiPh_2$; 2) [**2**][BArF] (5 mol%) + $H_2SiPh_2$ + $CO_2$, RT 25 hours.

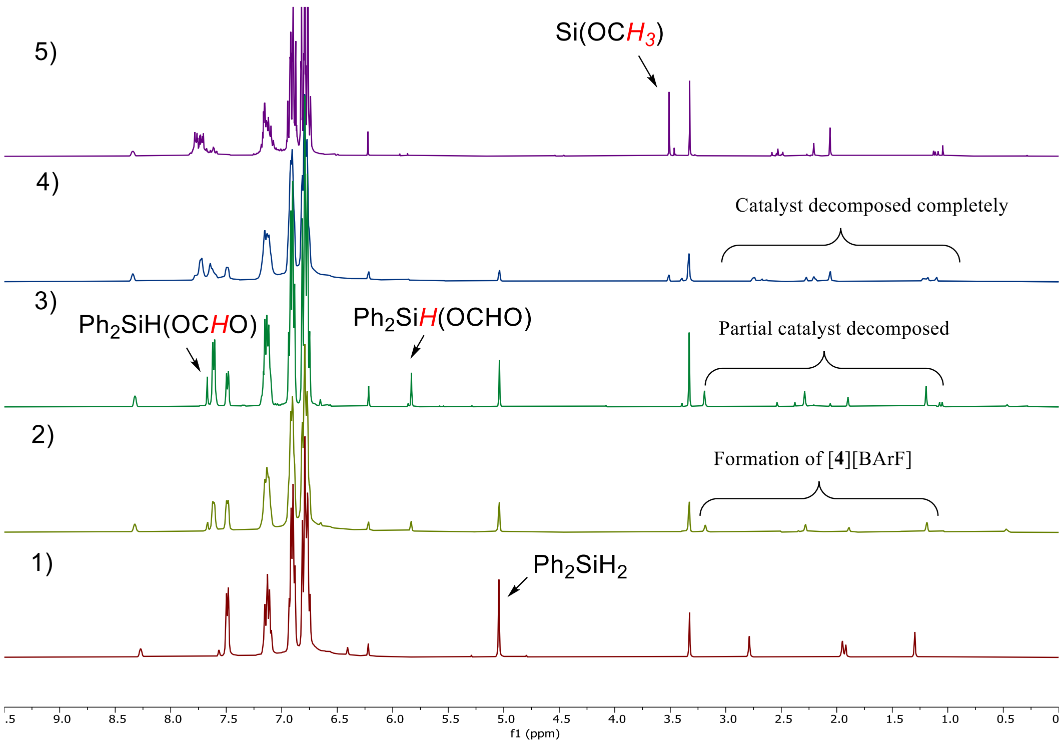

**Figure S40**. Stacked $^{1}$H NMR spectra of hydrosilylation of $CO_2$ with $H_2SiPh_2$ by [**2**][BArF] in $C_6D_6/C_6H_5F$ (4:1) at 50 °C: 1) [**2**][BArF] (5 mol%) + $H_2SiPh_2$; 2) [**2**][BArF] (5 mol%) + $H_2SiPh_2$ + $CO_2$, 50 °C 1 hour; 3) [**2**][BArF] (5 mol%) + $H_2SiPh_2$ + $CO_2$, 50 °C 2 hours; 4) [**2**][BArF] (5 mol%) + $H_2SiPh_2$ + $CO_2$, 50 °C 7 hours; 5) [**2**][BArF] (5 mol%) + $H_2SiPh_2$ + $CO_2$, 50 °C 25 hours.

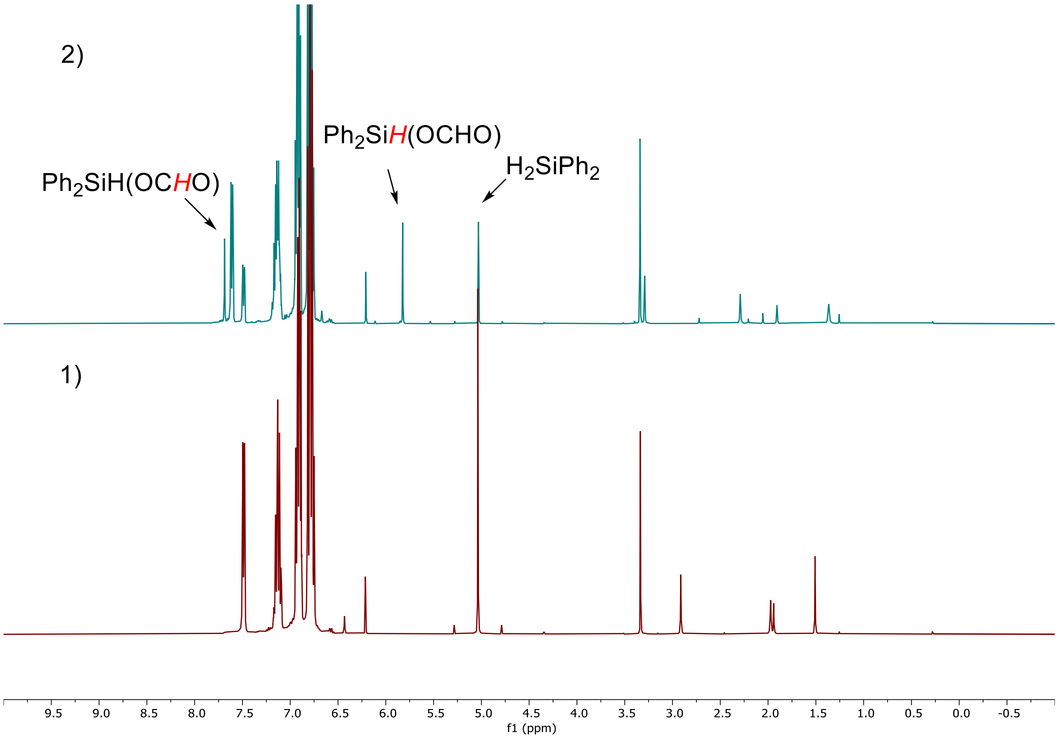


**Figure S41** Stacked $^{1}$H NMR spectra of hydrosilylation of $CO_2$ with $H_2SiPh_2$ by [**2**][$Al(OC(CF_3)_3)_4$] in $C_6D_6/C_6H_5F$ (4:1): 1) [**2**][$Al(OC(CF_3)_3)_4$] (5 mol%); 2) [**2**][$Al(OC(CF_3)_3)_4$] (5 mol%) + $H_2SiPh_2$ + $CO_2$, RT 25 hours.

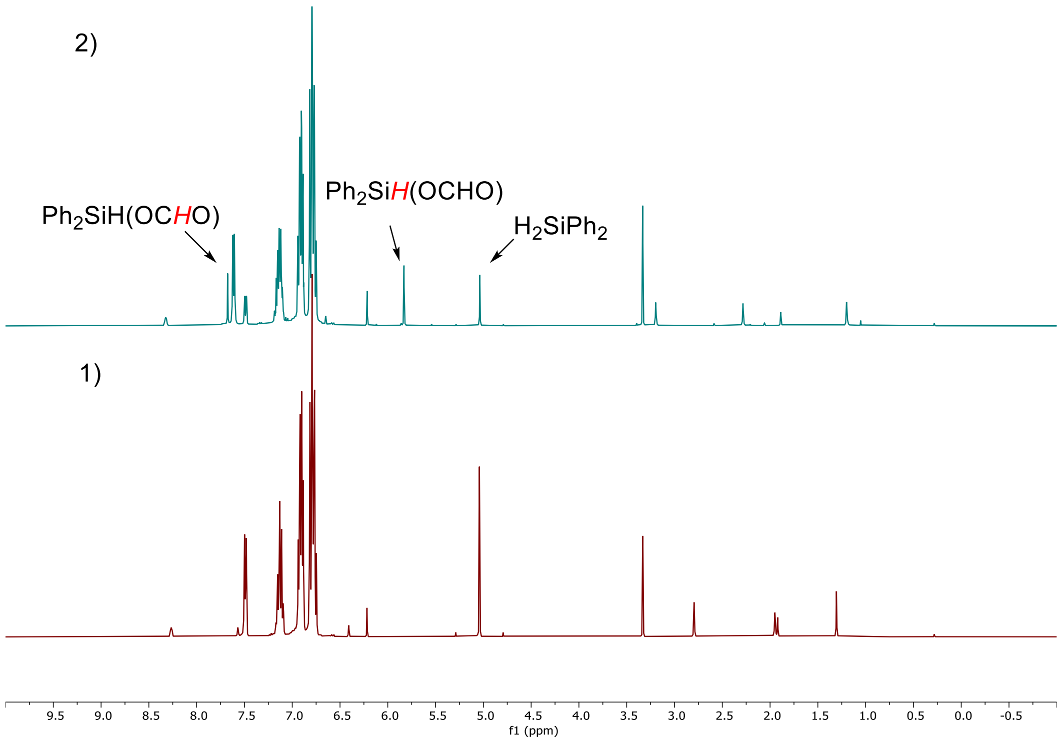


**Figure S42**. Stacked $^{1}$H NMR spectra of hydrosilylation of $CO_2$ with $H_2SiPh_2$ by [**2**][BArF] in $C_6D_6/C_6H_5F$ (4:1) in the presence of Hg: 1) [**2**][BArF] (5 mol%) + $H_2SiPh_2$; 2) [**2**][BArF] (5 mol%) + $H_2SiPh_2$ + $CO_2$, RT 25 hours.

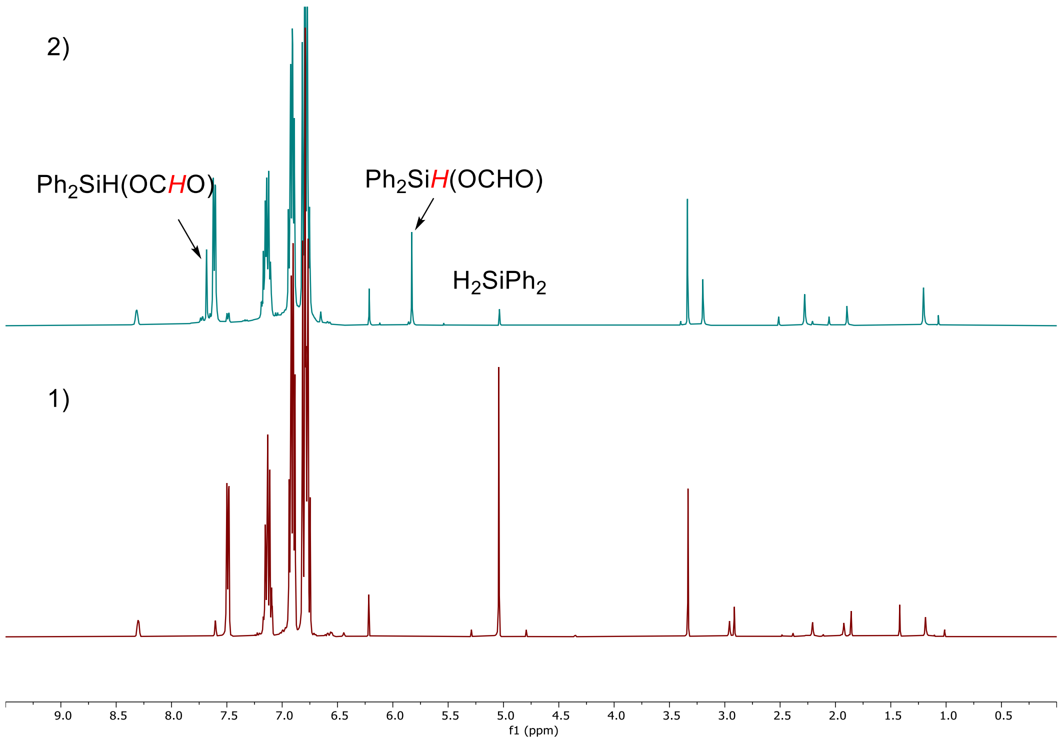

**Figure S43**. Stacked $^1$H NMR spectra of hydrosilylation of $CO_2$ with $H_2SiPh_2$ by [**3**][BArF]: 1) [**3**][BArF] (5 mol%) + $H_2SiPh_2$; 2) [**3**][BArF] (5 mol%) + $H_2SiPh_2$ + $CO_2$, RT 25 hours.

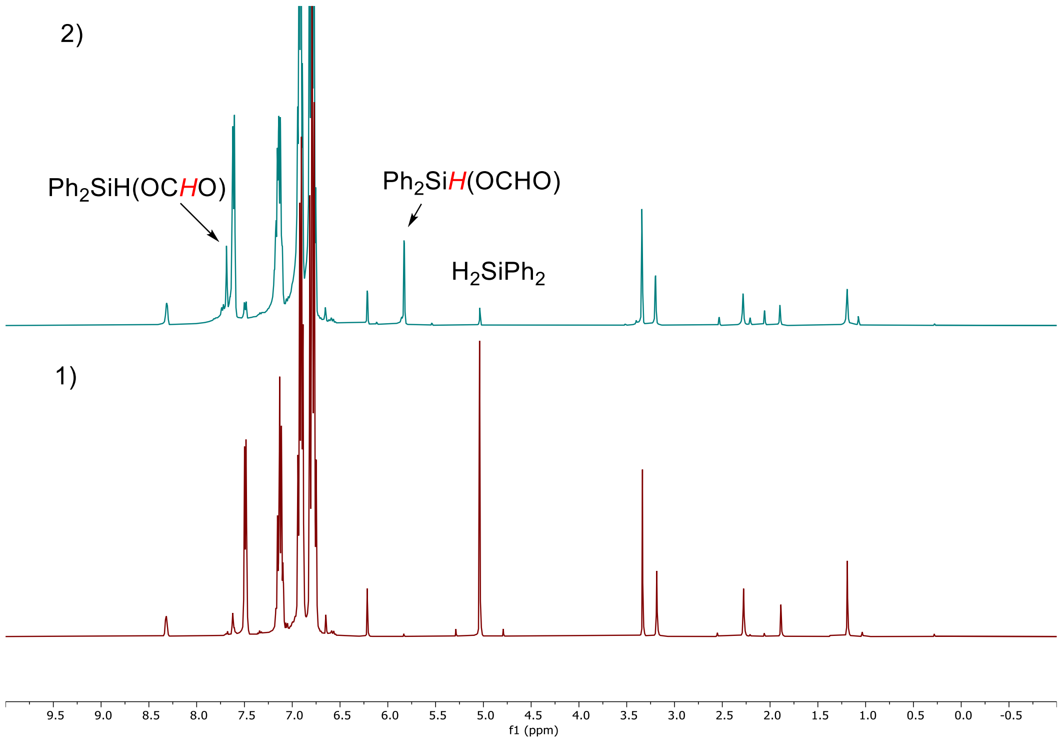


**Figure S44**. Stacked $^1$H NMR spectra of hydrosilylation of $CO_2$ with $H_2SiPh_2$ by [**4**][BArF]: 1) [**4**][BArF] (5 mol%) + $H_2SiPh_2$; 2) [**4**][BArF] (5 mol%) + $H_2SiPh_2$ + $CO_2$, RT 25 hours.

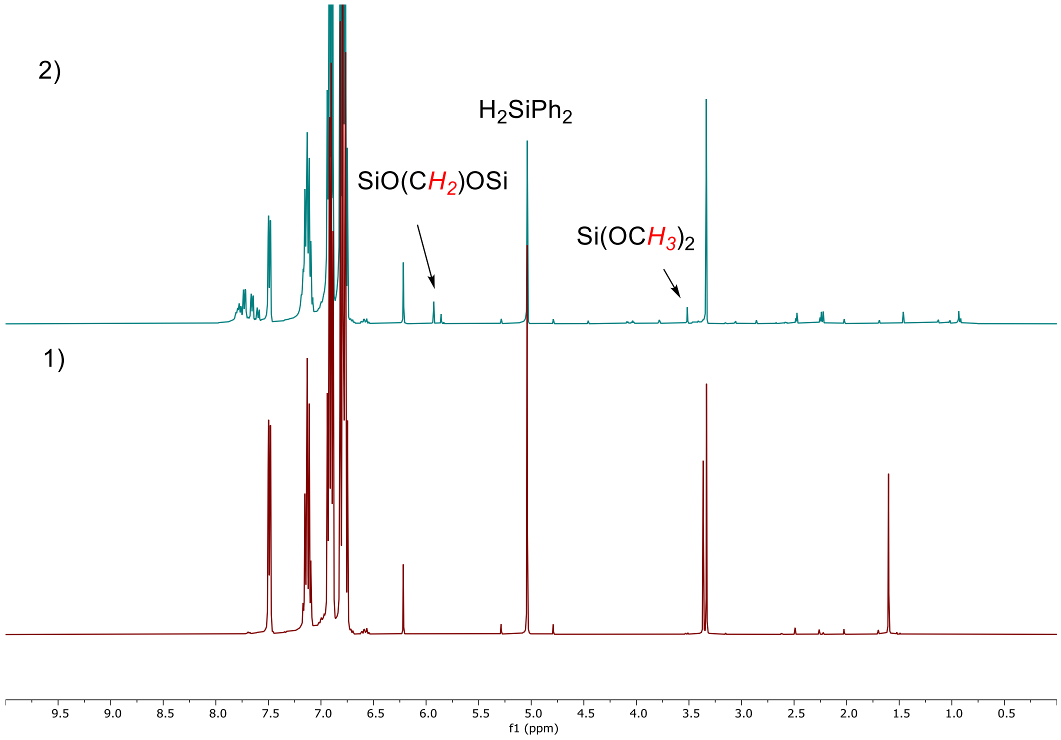

**Figure S45**. Stacked $^{1}$H NMR spectra of hydrosilylation of $CO_2$ with $H_2SiPh_2$ by $IMe_4$: 1) $IMe_4$ (10 mol%) + $H_2SiPh_2$; 2) $IMe_4$ + $H_2SiPh_2$ + $CO_2$, after 25 hours.

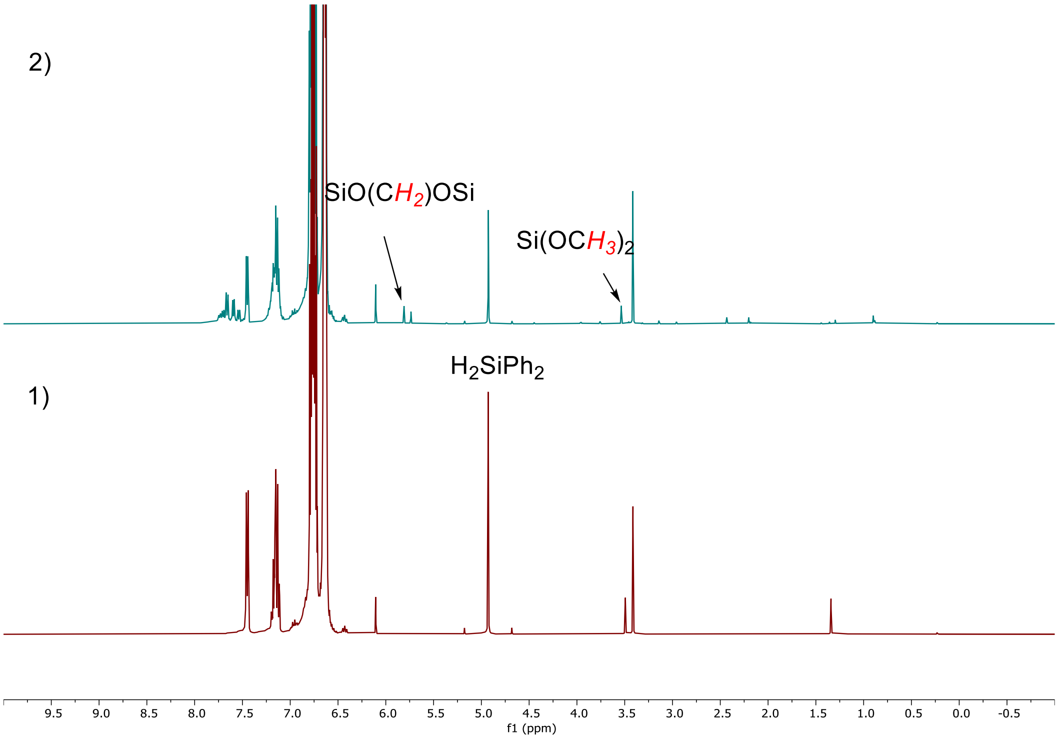


**Figure S46**. Stacked $^{1}$H NMR spectra of hydrosilylation of $CO_2$ with $H_2SiPh_2$ by $IMe_4$-$CO_2$: 1) $IMe_4$-$CO_2$ (10 mol%) + $H_2SiPh_2$; 2) $IMe_4$-$CO_2$ + $H_2SiPh_2$ + $CO_2$, RT 25 hours.

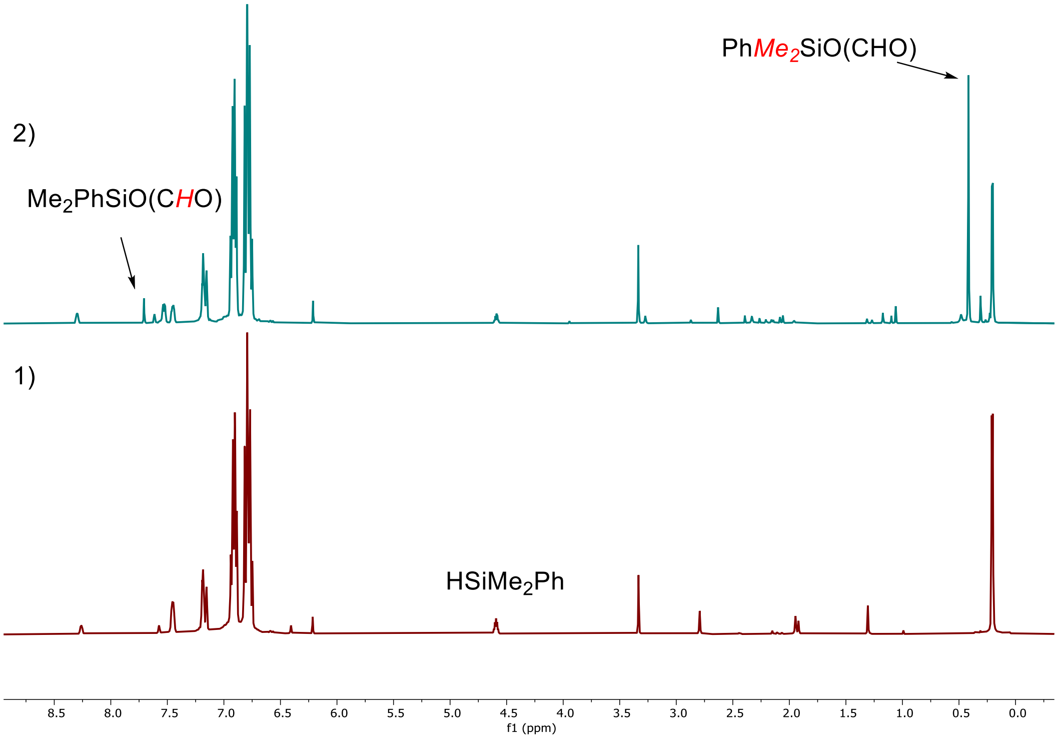

**Figure S47**. Stacked $^{1}$H NMR spectra of hydrosilylation of $CO_2$ with $HSiMe_2Ph$ by [**2**][BArF]: 1) [**2**][BArF] (5 mol%) + $HSiMe_2Ph$; 2) [**2**][BArF] (5 mol%) + $HSiMe_2Ph$ + $CO_2$, RT 25 hours.

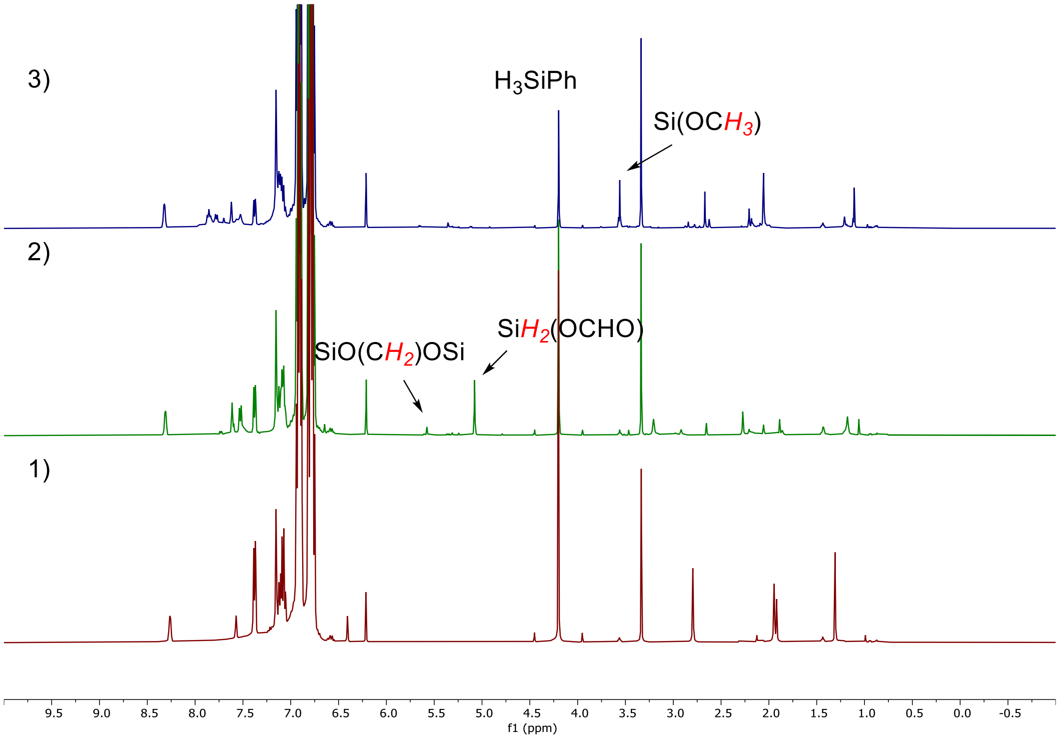


**Figure S48**. Stacked $^{1}$H NMR spectra of hydrosilylation of $CO_2$ with $H_3SiPh$ by [**2**][BArF]: 1) [**2**][BArF] (5 mol%) + $H_3SiPh$; 2) [**2**][BArF] (5 mol%) + $H_3SiPh$ + $CO_2$, RT 2 hours; [**2**][BArF] (5 mol%) + $H_3SiPh$ + $CO_2$, RT 12 hours.

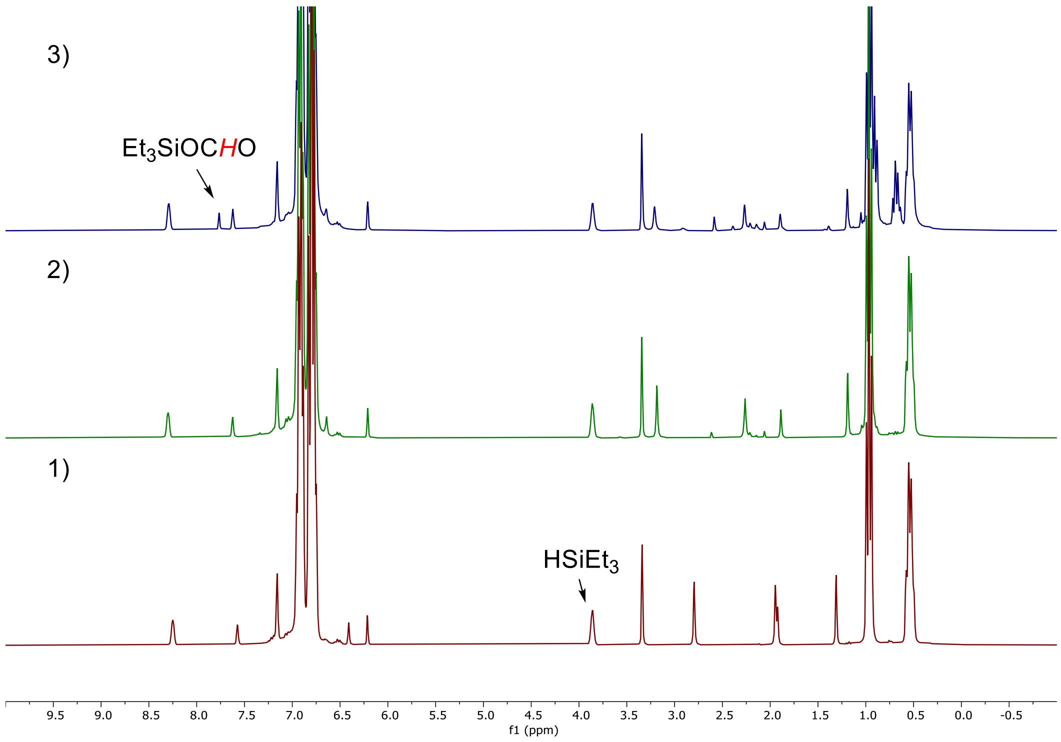


**Figure S49**. Stacked $^{1}H$ NMR spectra of hydrosilylation of $CO_2$ with $HSiEt_3$ by [**2**][BArF]: 1) [**2**][BArF] (5 mol%) + $HSiEt_3$; 2) [**2**][BArF] (5 mol%) + $HSiEt_3$ + $CO_2$, RT 21 hours; [**2**][BArF] (5 mol%) + $HSiEt_3$ + $CO_2$, RT 21 hours + 60 $^{o}C$ 25 hours.

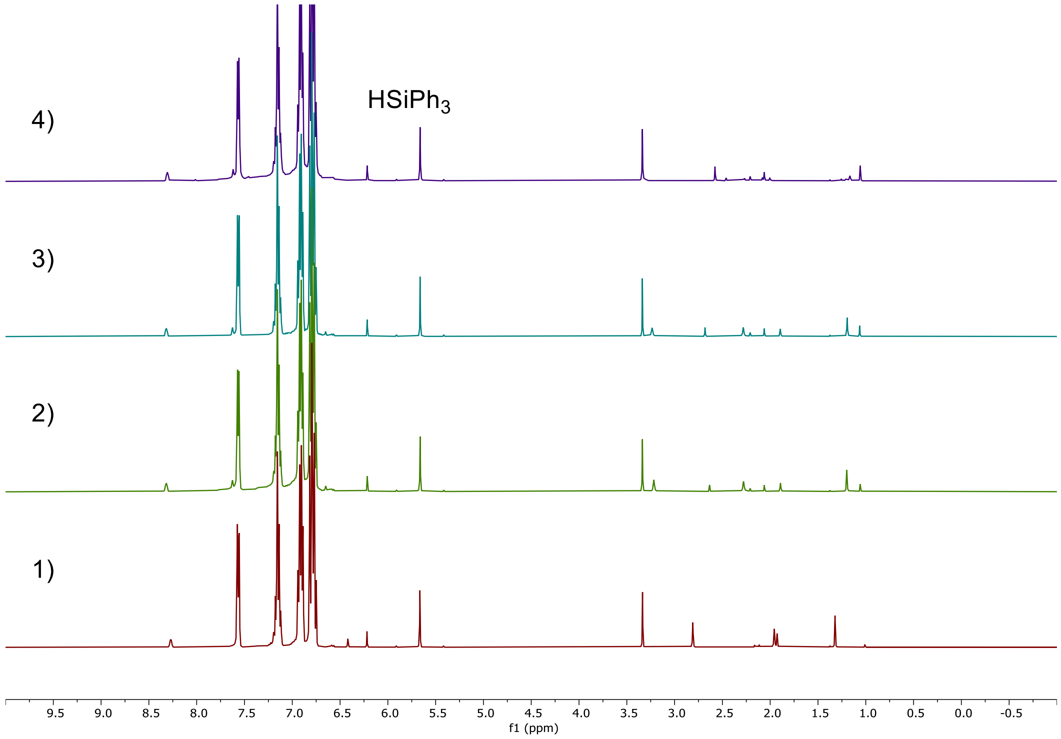

**Figure S50**. Stacked $^{1}$H NMR spectra of hydrosilylation of $CO_2$ with $HSiPh_3$ by [**2**][BArF]: 1) [**2**][BArF] (5 mol%) + $HSiPh_3$; 2) [**2**][BArF] (5 mol%) + $HSiPh_3$ + $CO_2$, RT 17 hours + 60 °C 18 hours + 80 °C 18 hours.

## 2. Mechanistic Studies

2.1 Kinetic Isotope Experiments

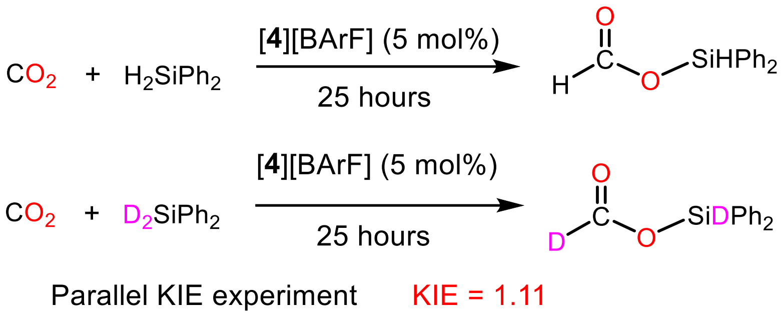


[**2**][BArF] (5 mol%, 4.00 µmol, 80.0 µL, 0.05 M in $C_6H_5F$) and 1,3,5-trimethoxybenzene (0.10 eq, 8.0 µmol) were introduced into 0.32 mL of $C_6D_6$ within a J-Young NMR tube. The solution was degassed and refilled with 1 bar of $CO_2$ at room temperature. The solution of [**4**][BArF] was obtained after 21 hours and excess $CO_2$ was removed, then $H_2SiPh_2$ (1.0 eq, 80.0 µmol, 14.9 µL) (or $D_2SiPh_2$ (1.0 eq, 80.0 µmol, 14.7 µL)) was added in gloves box. The solution was degassed again and then pressurised with 1 bar of $CO_2$ at room temperature. The reaction progression was monitored via $^{1}$H NMR spectroscopy by the consumption of silane alongside the emergence of the respective hydrosilylation products resonances. After 25 hours, the conversion of silane was determined by comparing integrals of the unreacted silane resonances to the integrals of the internal standard.

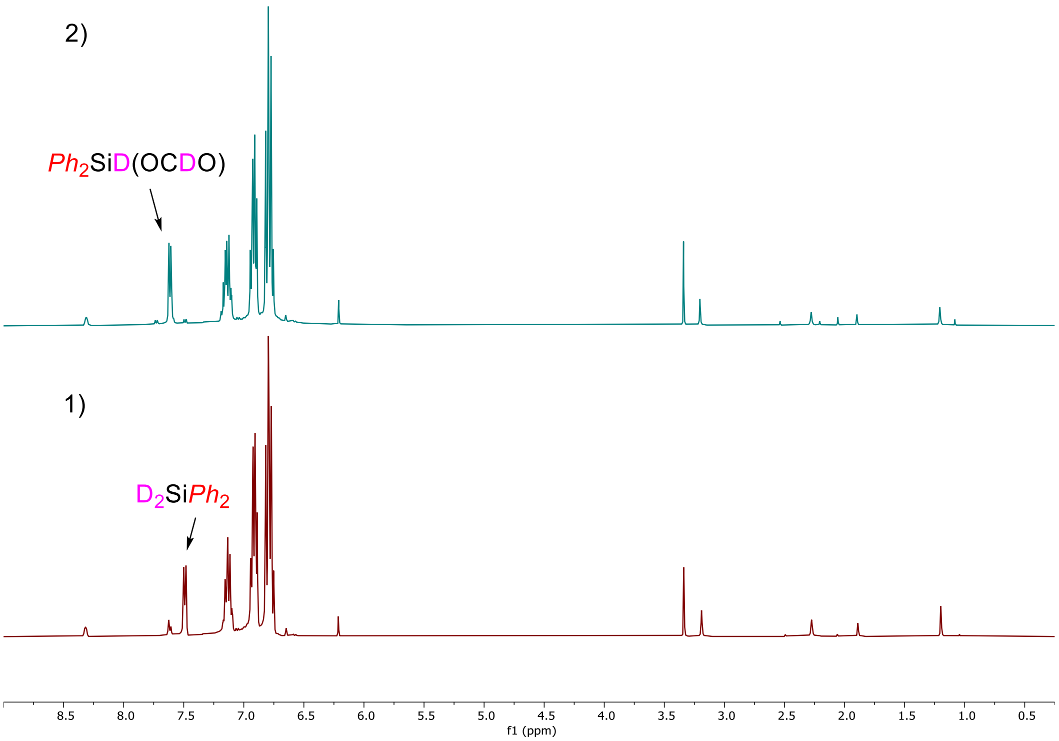


**Figure S51**. Stacked $^{1}$H NMR spectra of hydrosilylation of $CO_2$ with $D_2SiPh_2$ by [**4**][BArF]: 1) [**4**][BArF] (5 mol%) + $D_2SiPh_2$; 2) [**4**][BArF] (5 mol%) + $H_2SiPh_2$ + $CO_2$, RT 25 hours.

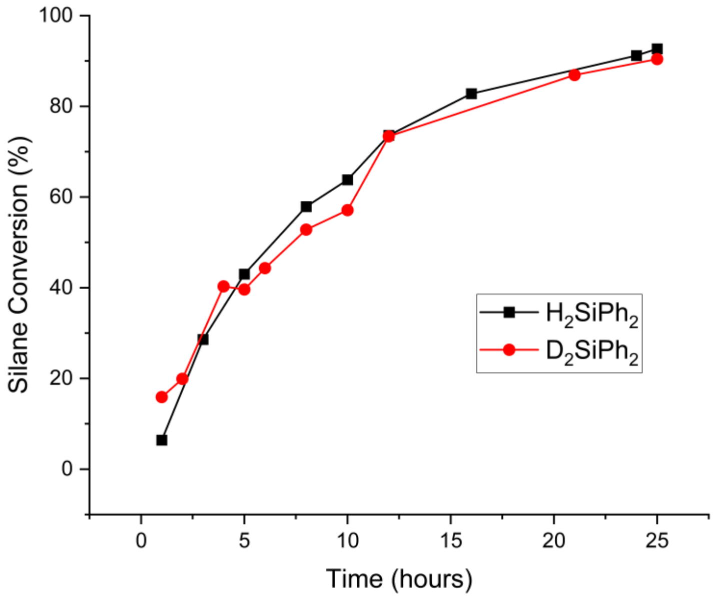


**Figure S52.** Kinetic isotope effect experiments.

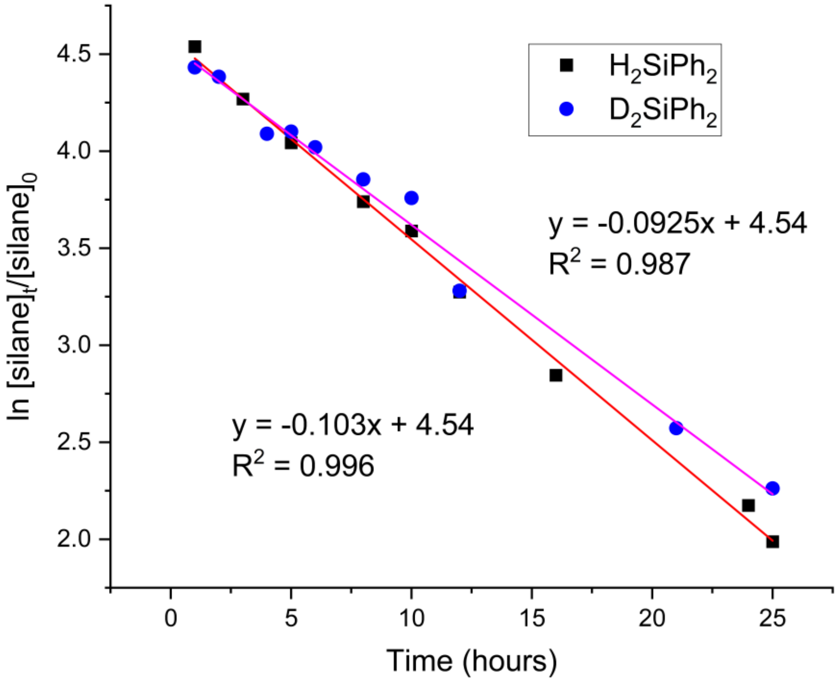


**Figure S53.** Kinetic isotope effect experiments.

2.2 H/D Exchange Experiments

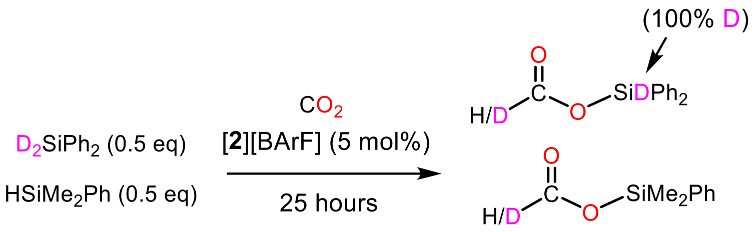


[**2**][BArF] (5 mol%, 4.00 μmol, 80.0 μL, 0.05 M in $C_6H_5F$), $D_2SiPh_2$ (0.5 eq, 40.0 μmol, 8.35 μL)), $HSiMe_2Ph$ (0.5 eq, 40.0 μmol, 6.13 μL), and 1,3,5-trimethoxybenzene (0.10 eq of the $H_2SiPh_2$) as an internal standard were dissolved in $C_6D_6$ (0.32 mL) in a J-Young NMR tube. The solution was rapidly degassed using a Schlenk line and then refilled with 1 bar of $CO_2$ at room temperature. The progression of reaction was monitored via $^1H$ NMR spectroscopy.

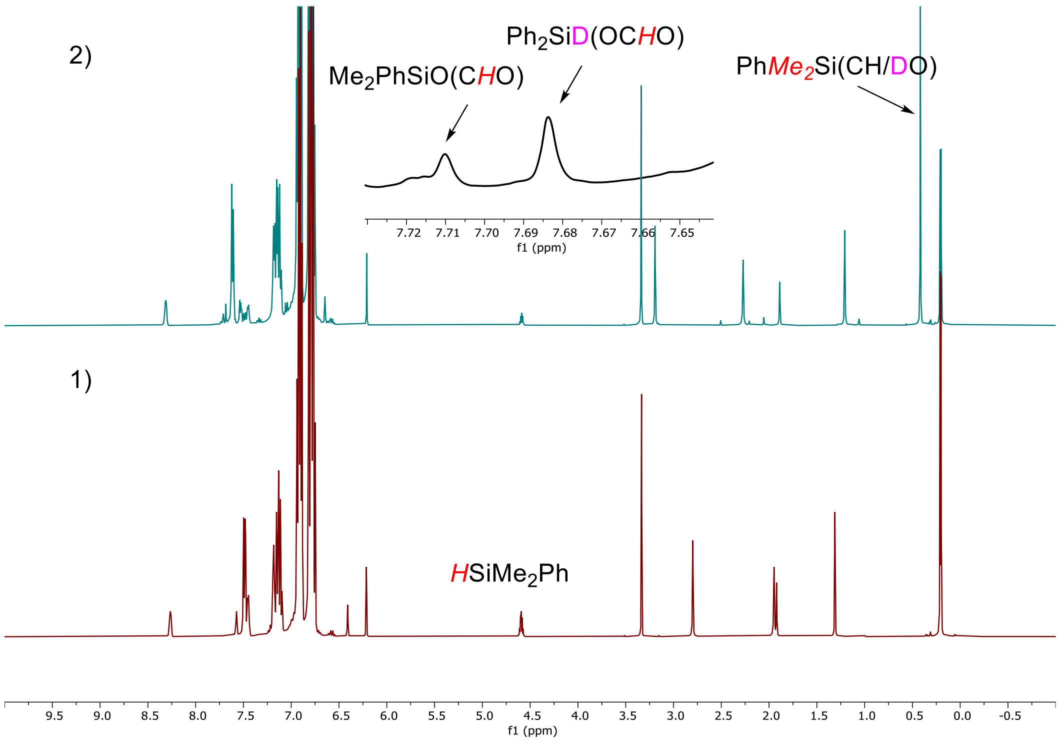


**Figure S54**. Stacked $^1$H NMR spectra of hydrosilylation of $CO_2$ with $D_2SiPh_2$/$HSiMe_2Ph$ by [**2**][BArF]: 1) [**2**][BArF] (5 mol%) + $D_2SiPh_2$ + $HSiMe_2Ph$; 2) [**2**][BArF] (5 mol%) + $D_2SiPh_2$ + $HSiMe_2Ph$ + $CO_2$, RT 25 hours.

## 3. X-Ray Crystallographic Details

General considerations

Single crystals diffraction data were recorded on a Bruker Photon D8 Venture DUO IMS system equipped with a Helios optic monochromator and a Mo IMS microsource (λ = 0.71073 Å) and an Atlas SuperNova system equipped with a mirror monochromator and a Cu micro-focus sealed X-ray tube (λ = 1.54178 Å). The data collection was performed, using the APEX IV software package[S4] and CrysAlisPro[S5] on single crystals coated with Fomblin®Y as perfluorinated ether. The single crystal was picked on a micro sampler, transferred to the diffractometer, and measured frozen under a stream of cold nitrogen. A matrix scan was used to determine the initial lattice parameters. Reflections were merged and corrected for Lorenz and polarization effects, scan speed, and background using SAINT.[S6] Absorption corrections, including odd and even ordered spherical harmonics were performed using SADABS.[S6] Space group assignments were based upon systematic absences, E statistics, and successful refinement of the structures. Structures were solved by direct methods with the aid of successive difference Fourier maps and were refined against all data using the APEX IV software in conjunction with SHELXL-2014[S7] and SHELXLE.[S8] H atoms were placed in calculated positions and refined using a riding model, with methylene and aromatic C–H distances of 0.99 and 0.95 Å, respectively, and Uiso(H) = 1.2 Ueq(C). Non-hydrogen atoms were refined with anisotropic displacement parameters. Fullmatrix least-squares refinements were carried

out by minimizing Σw(Fo2-Fc2)2 with the SHELXL weighting scheme.[S9] Neutral atom scattering factors for all atoms and anomalous dispersion corrections for the non-hydrogen atoms were taken from International Tables for Crystallography.[S10] The images of the crystal structures were generated by Mercury.[S11] The CCDC numbers 2255434, 2255353 and 2255355 contain the supplementary crystallographic data for the structures [**2**][BArF], [**3**][BArF] and [**4**][BArF], respectively. These data can be obtained free of charge from the Cambridge Crystallographic Data Centre via https://www.ccdc.cam.ac.uk/structures/.

**Table S2**. Crystallographic details

| **Compound #** | [**2**][BArF] | [**3**][BArF] | [**4**][BArF] |
|---|---|---|---|
| **CCDC-Number** | 2255434 | 2255353 | 2255355 |
| **Empirical formula** | $C_{70}H_{61}BF_{24}N_4Sn$ | $C_{79}H_{80}N_5O_2B_5F_{24}Sn$ | $C_{72}H_{61}N_4O_4BF_{24}Sn$ |
| **Formula weight** | 1543.84 | 1760.22 | 1631.74 |
| **Temperature/K** | 100.00 | 100.00 | 100.00 |
| **Crystal system** | triclinic | triclinic | triclinic |
| **Space group** | P-1 | P-1 | P-1 |
| **a/Å** | 13.4232(4) | 15.2450(13) | 10.4739(5) |
| **b/Å** | 17.0569(6) | 17.2704(13) | 16.0890(8) |
| **c/Å** | 18.5469(7) | 17.6243(14) | 23.3384(11) |
| **α/°** | 102.535(3) | 92.495(3) | 109.831(2) |
| **β/°** | 101.839(3) | 102.397(3) | 92.107(2) |
| **γ/°** | 109.969(3) | 112.807(2) | 102.860(2) |
| **Volume/Å$^3$** | 3711.1(2) | 4136.1(6) | 3579.7(3) |
| **Z** | 2 | 2 | 2 |
| **$\rho_{calc}$g/cm$^3$** | 1.382 | 1.413 | 1.514 |
| **μ/mm$^{-1}$** | 3.644 | 0.411 | 0.471 |
| **F(000)** | 1560.0 | 1792.0 | 1648.0 |
| **Crystal size/mm$^3$** | 0.1 × 0.07 × 0.05 | 0.382 × 0.287 × 0.198 | 0.347 × 0.26 × 0.106 |
| **Radiation** | CuKα (λ = 1.54184) | MoKα (λ = 0.71073) | MoKα (λ = 0.71073) |
| **2Θrange for data collection/°** | 5.124 to 147.842 | 4.684 to 50.7 | 3.864 to 50.7 |
| **Index ranges** | -16 ≤ h ≤ 11, -20 ≤ k ≤ 21, -23 ≤ l ≤ 22 | -18 ≤ h ≤ 18, -20 ≤ k ≤ 20, -21 ≤ l ≤ 21 | -12 ≤ h ≤ 12, -19 ≤ k ≤ 19, -27 ≤ l ≤ 28 |
| **Reflections collected** | 26916 | 93421 | 66144 |
| **Independent reflections** | 14585 [$R_{int}$ = 0.0442, $R_{sigma}$ = 0.0593] | 15133 [$R_{int}$ = 0.0264, $R_{sigma}$ = 0.0179] | 13105 [$R_{int}$ = 0.0252, $R_{sigma}$ = 0.0179] |
| **Data/restraints/parameters** | 14585/9/969 | 15133/272/1069 | 13105/737/1206 |
| **Goodness-of-fit on F$^2$** | 1.032 | 1.067 | 1.029 |
| **Final R indexes [I>=2σ (I)]** | $R_1$ = 0.0484, $wR_2$ = 0.1198 | $R_1$ = 0.0418, $wR_2$ = 0.1026 | $R_1$ = 0.0299, $wR_2$ = 0.0702 |
| **Final R indexes [all data]** | $R_1$ = 0.0649, $wR_2$ = 0.1323 | $R_1$ = 0.0457, $wR_2$ = 0.1061 | $R_1$ = 0.0325, $wR_2$ = 0.0719 |
| **Largest diff. peak/hole / e Å$^{-3}$** | 0.78/-0.96 | 1.24/-0.81 | 0.75/-0.52 |

## 4. Quantum chemical calculations

Calculations were carried out using ORCA 5.0.4 software.[S12]

Geometry optimizations were carried using the $r^2$SCAN-3c composite method, utilizing the regularized and restored SCAN functional,[S13-14] geometrical counterpoise correction gCP,[S15] the atom-pairwise dispersion correction based on tight binding partial charges (D4),[S16-18] the def2-mTZVPP basis set and def2-mTZVPP/J auxiliary basis set.[S19] Effective core potential def2-ECP was used for Sn (ECP parameters for Sn have been obtained from TURBOMOLE (7.0.2)).[S20] The optimized geometries were verified as minima or transition states by analytical frequency calculations. The transition states were additionally verified by IRC calculations. Single point calculations of the optimized geometries were carried out at the $r^2$SCAN-3c level using the SMD solvation module[S21] to obtain electrostatic contribution and the cavity term in order to account for the solvent effects. To get more accurate electronic energies for the mechanistic investigations, single point calculations of the $r^2$SCAN-3c optimized geometries were carried using the PW6B95 [S22] functional, with D4 dispersion correction, the def2-QZVPP[S23] basis set and def2/J[S24] and def2-QZVPP/C[S25] auxiliary basis sets. def2-ECP was used for Sn. The method is denoted as PW6B95-D4(SMD)/def2-QZVPP//$r^2$SCAN-3c. The summary of the thermochemistry results is presented in Table S2. The NBO analysis was done using the NBO7 software,[S26] at the PBE0[S27]/def2-TZVP[S28]/$r^2$SCAN-3c level of theory.

**Table S3**. Calculated energies (Eh). $E_{PW6B95}$ - electronic energy at the PW6B95-D4/def2-QZVPP//$r^2$SCAN-3c level; G-$E_{el}$ - Gibbs energy minus the electronic energy at the $r^2$SCAN-3c// $r^2$SCAN-3c level; $G_{cds}$ (cavity term) and $G_{enp}$ (electrostatic contribution) at $r^2$SCAN-3c (SMD=Benzene)// $r^2$SCAN-3c level; $G_{conc}$ - concentration-induced free-energy shift ($G_{conc}$ = RTln(24.5)); G – free energy at the PW6B95-D4(SMD)/def2-QZVPP//$r^2$SCAN-3c level, G = $E_{PW6B95}$ + [G-$E_{el}$] + $G_{cds}$ + $G_{enp}$ + $G_{conc}$. Thermochemistry at 298.15 K.

| Compound | ID | $E_{PW6B95}$ | G-$E_{el}$ | $G_{cds}$ | $G_{enp}$ | $G_{conc}$ | G |
|---|---|---|---|---|---|---|---|
| [**2**]$^+$ | 3525538 | -1913.90390 | 0.70795 | -0.04503 | -0.02061 | 0.00302 | -1913.25857 |
| [**2**][BArF] | 3536119 | -5567.93743 | 1.05894 | -0.03668 | -0.01860 | 0.00302 | -5566.93074 |
| [**2a**]$^+$ | 3531069 | -1529.73254 | 0.53363 | -0.04332 | -0.01812 | 0.00302 | -1529.25733 |
| [**2a**][BArF] | 3539097 | -5183.77457 | 0.88298 | -0.03103 | -0.01624 | 0.00302 | -5182.93584 |
| [**3**]$^+$ | 3525578 | -2102.82456 | 0.71945 | -0.04727 | -0.02122 | 0.00302 | -2102.17058 |
| [**3**][BArF] | 3746351 | -5756.86611 | 1.07621 | -0.03610 | -0.01667 | 0.00302 | -5755.83965 |
| [**3a**]$^+$ | 3529038 | -1718.65656 | 0.54497 | -0.04506 | -0.01757 | 0.00302 | -1718.17119 |
| [**3a**][BArF] | 3690093 | -5372.69690 | 0.89536 | -0.03274 | -0.01600 | 0.00302 | -5371.84726 |
| [**4**]$^+$ | 3525581 | -2291.73103 | 0.72772 | -0.05029 | -0.02500 | 0.00302 | -2291.07558 |
| [**4**][BArF] | 3748659 | -5945.78061 | 1.08218 | -0.03854 | -0.02036 | 0.00302 | -5944.75431 |
| **TS(2_3)** | 3527035 | -2102.76624 | 0.71701 | -0.04662 | -0.01985 | 0.00302 | -2102.11268 |
| **TS(3_4)** | 3529058 | -2291.69025 | 0.72861 | -0.04819 | -0.02048 | 0.00302 | -2291.02728 |
| **TS1** | 4191715 | -3046.84610 | 0.91624 | -0.05369 | -0.02890 | 0.00302 | -3046.00943 |
| **INT_A** | 3685470 | -3046.84678 | 0.91442 | -0.05369 | -0.03156 | 0.00302 | -3046.01460 |

| | | | | | | | |
|---|---|---|---|---|---|---|---|
| **TS2** | 3969171 | -3046.84872 | 0.91828 | -0.05673 | -0.02918 | 0.00302 | -3046.01333 |
| **INT_B** | 597622 | -1327.40280 | 0.33756 | -0.04675 | -0.01672 | 0.00302 | -1327.12569 |
| **INT_C** | 3549415 | -1719.40308 | 0.54938 | -0.02562 | -0.01758 | 0.00302 | -1718.89388 |
| **TS3** | 3607573 | -3046.84986 | 0.91654 | -0.05162 | -0.02707 | 0.00302 | -3046.00898 |
| **INT_D** | 612540 | -1328.16718 | 0.34679 | -0.01659 | -0.01457 | 0.00302 | -1327.84852 |
| **TS4** | 3734923 | -1328.15477 | 0.34547 | -0.01265 | -0.01423 | 0.00302 | -1327.83316 |
| **$IMe_4$** | 592934 | -384.10329 | 0.14625 | -0.01132 | -0.01048 | 0.00302 | -383.97582 |
| **$IMe_4CO_2$** | 592935 | -573.02057 | 0.15737 | -0.01695 | -0.00877 | 0.00302 | -572.88590 |
| **$H_2SiPh_2$** | 3543195 | -755.11620 | 0.16050 | -0.00590 | -0.00935 | 0.00302 | -754.96794 |
| **$HCO_2SiHPh_2$** | 3614592 | -944.04318 | 0.17260 | -0.00777 | -0.00900 | 0.00302 | -943.88432 |
| **$[BArF]^-$** | 3536159 | -3653.94294 | 0.31746 | -0.03055 | -0.00259 | 0.00302 | -3653.65559 |
| **$CO_2$** | 592933 | -188.89490 | -0.00901 | -0.00094 | 0.00057 | 0.00302 | -188.90126 |

According to the request by one of the referees, in addition to the Lewis structure of **$[2]^+$** presented in the main text, also shown in **Figure S55 $[2]^+_a$**, here we additionally discuss the ionic representation, i.e. **$[2]^+_b$**. The dative form, **$[2]^+_a$** is a bis(NHC) stabilized stannyliumylidene in which the interactions between the NHC units and the Sn centre are depicted as dative bonds. In **$[2]^+_b$** the negative charge is located on the Sn atom to which the two positively charged NHC moieties are bound covalently **$[2]^+_b$**, which results a stannyl-anion-type species.

As discussed in the main text, the NBO depiction of bonding between the NHCs and the low valent Sn centre in **$[2]^+$** is that of polarized bonds between the carbons and the tin. This may hint to the stannyl anion character of **$[2]^+$**, which is why **$[2]^+_b$** is considered.

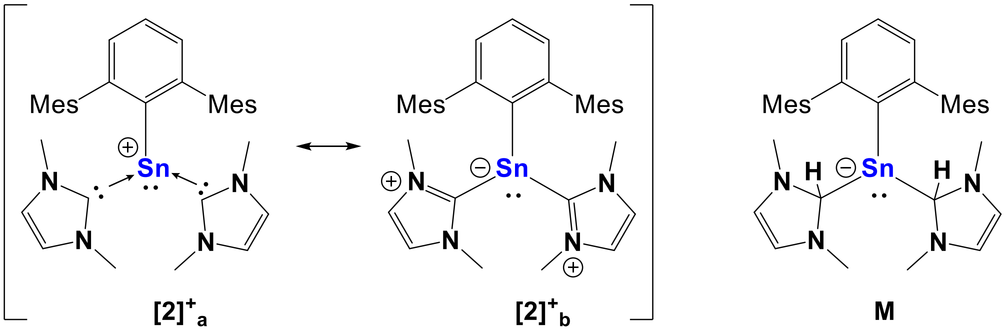


**Figure S55.** Resonance structures of donor stabilized stannyliumylidene [**2**]$^+$ and the related model system stannyl anion **M**.

In the main text, we depicted **$[2]^+$** as **$[2]^+_a$** because we accounted for the following considerations. In general, it is a common situation in the NBO analysis for a dative bond to be shown as a bonding orbital polarized toward the donor atom, reflecting that the electron pair is largely provided by the donor. Additionally, it is also worth looking at the

Wiberg bond index and Mayer bond order. In this case, the bond is strongly polarized toward carbon and the Wiberg bond index is low for each Sn-C bond (0.58 for both). The Mayer bond orders are also quite low (0.48, 0.42). In **[2]$^+$** the charge distribution is such that the NPA charge on Sn centre in +0.79. Additionally, the NHC-Sn bonds are longer 2.34 and 2.33 Å in comparison to the covalent Sn-C$^{Ar}$ bond (2.24 Å). Furthermore, the reactivity of the complex, where NHC behaves as a donor ligand, which can facilely dissociate from the metal centre, intuitively leads to the dative bond representation being more plausible.

In order to distinguish between the electronic properties of donor stabilized stannyliumylidene and stannyl anion we carried out additional calculation of compound **M**, which is obtained by substituting the carbenic carbons with H$^-$ (**Figure S55, S56**).

As in **[2]$^+$**, in **M** the NPA charge on the Sn centre is positive (+0.74 el.). However, the Sn-C Wiberg bond indexes of 0.63 and 0.65 are somewhat higher than those in **[2]$^+$** (0.58 and 0.58). The Mayer bond orders in the more covalent bonds Sn-C of **M** are also higher than in **[2]$^+$**: 0.73 and 0.75 vs 0.48 and 0.42.

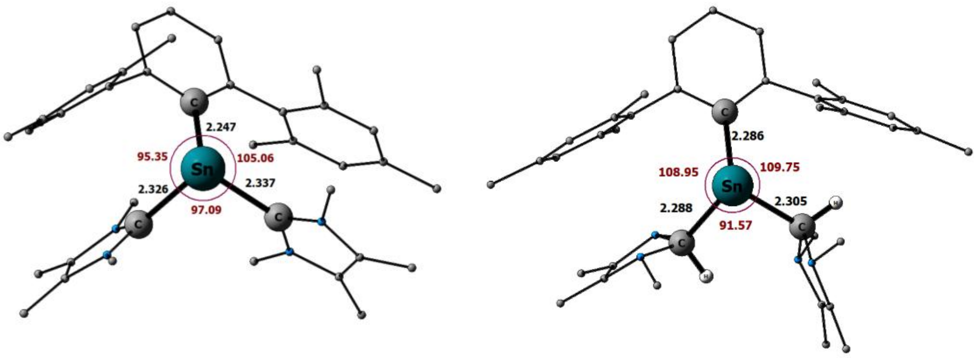


**Figure S56.** Optimized geometries of **[2]$^+$** and **M**.

In term of geometries, the optimized structure of **M** exhibits a substantial shortening of the Sn-C bonds by 0.038 and 0.031 Å (**Figure S56**)., which would be typical for more covalent bonds. Also, the geometry of the Sn centre in **M** is less pyramidalized than in **[2]$^+$** $\Sigma\alpha$=310.27° vs $\Sigma\alpha$=305.46°, respectively, which would be typical for a stannyl anion.

According to the NBO analysis in **M** the Sn-C bonds are slightly less polarized than in **[2]$^+$** : Sn(22.54%)-C(77.46%) and Sn(21.12%)-C(78.88%) in **M** vs Sn(18.25%)-C(81.75%) and Sn(18.72%)-C(81.28%).

The very important difference in the electronic structure between the two species is in the hybridization of the orbitals that constitute the Sn-C interaction. In **M**, in comparison to **[2]$^+$**, there is significant increase of the s character of the Sn hybrid orbitals and a significant increase in the p character of the C hybrid orbitals in comparison to **[2]$^+$**: Sn(sp$^{9.11}$)-C(sp$^{2.61}$) and Sn(sp$^{9.91}$)-C(sp$^{2.50}$) in **M** vs Sn(sp$^{19.72}$)-C(sp$^{1.45}$) and Sn(sp$^{15.52}$)-

C(sp$^{1.45}$) in **[2]⁺**. And this is the distinction between the formal C(sp$^2$)→Sn(p) interaction between a donor and stannyliumylidene, and the C(sp$^3$)-Sn(sp$^3$) interaction expected in a stannyl anion.

In general, we are not strictly in favour of either dative or ionic representations. We think that both annotations could be correct, and each case should be looked at separately. In this case, when comparing **$[2]^+_a$** with **$[2]^+_b$**, we think that the representation of **$[2]^+_a$** with dative bonds between the Sn-centre and the NHCs is the more intuitive and reflects better the electronic properties and the reactivity of [**2**]$^+$.

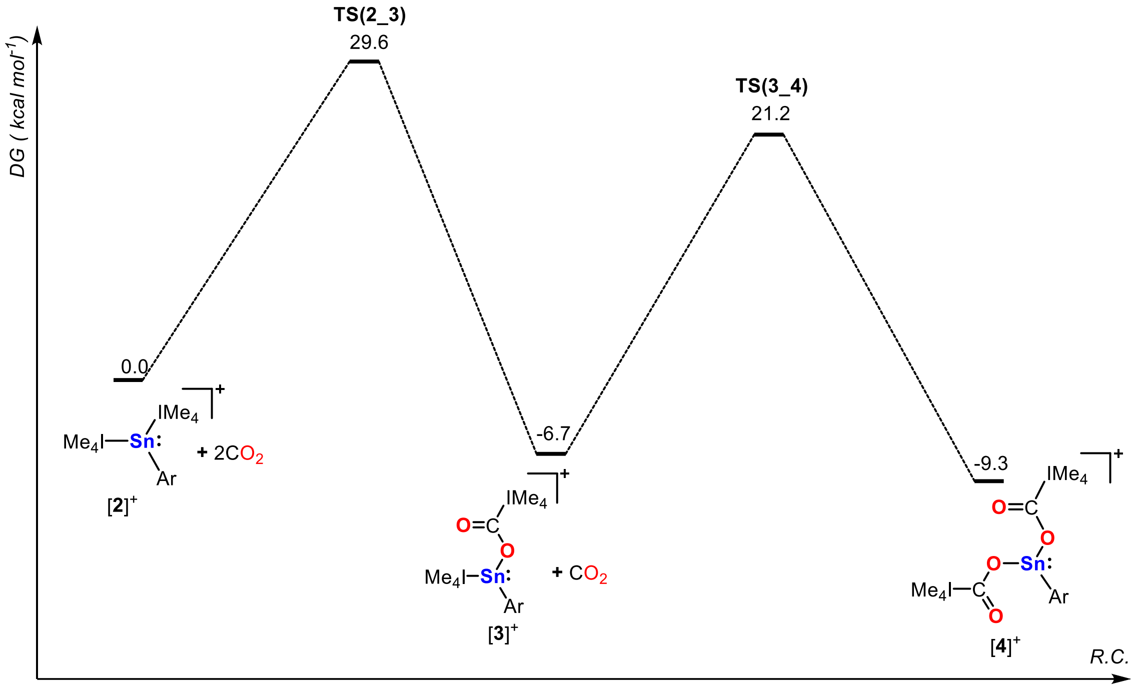


**Figure S57**. Calculated reaction coordinate diagram for the concerted reactions of [**2**]$^+$ with $CO_2$ to form [**3**]$^+$ and [**4**]$^+$.

In addition to the reaction of [**2**]$^+$ to form [**3**]$^+$ and [**4**]$^+$ via the stepwise dissociation of $IMe_4$ described in Scheme 4, we also considered a mechanism in which [**2**]$^+$ and [**3**]$^+$ react with $CO_2$ concertedly (Scheme S57). In this case, the barrier for the first reaction is 29.6 kcal mol$^{-1}$ (**TS(2_3)**). For the second reaction the barrier (**TS(3_4)**) (at 21.2 kcal mol$^{-1}$) is 27.9 kcal mol$^{-1}$ (**TS(3_4)**). This makes this mechanism kinetically less preferable to the one which involves the $IMe_4$ dissociation, presented in Scheme 4 in the main text.

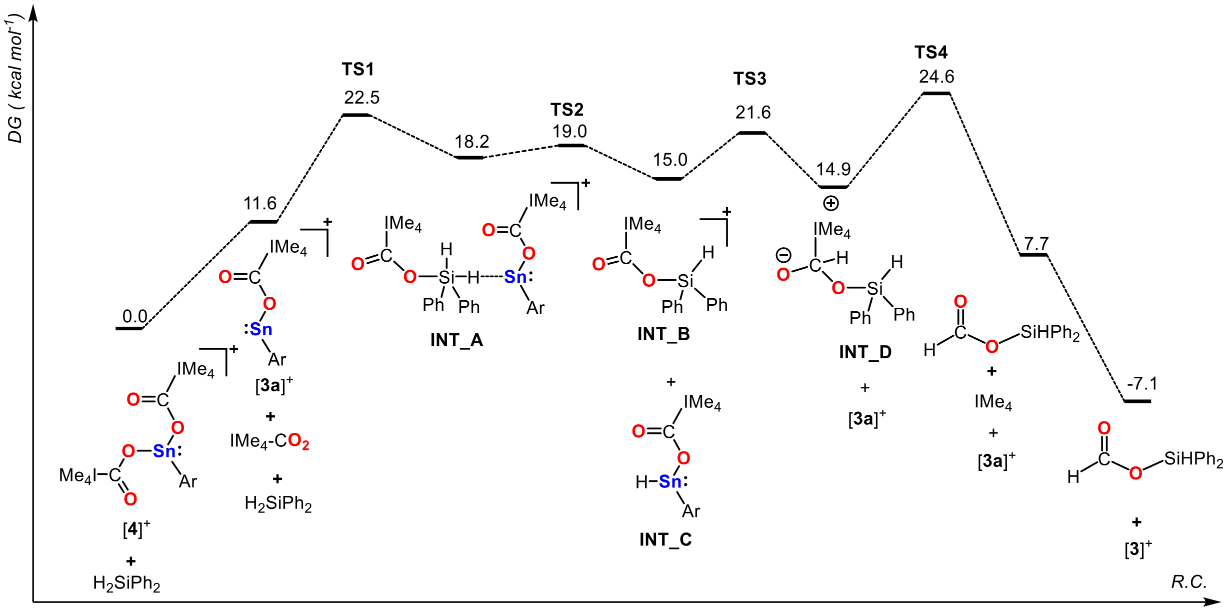


**Figure S58**. Calculated reaction coordinate diagram for the reaction of [**4**]+ with $H_2SiPh_2$.

The proposed mechanism for the reaction of [**4**]+ with $H_2SiPh_2$ to form the $CO_2$ hydrosilylation product $Ph_2SiH(OCHO)$ and [**3**]+ as observed experimentally (Scheme 3) is presented in Figure S58. Drawings of the geometries of key intermediates and transition states are shown in Figure S59. In the first step of the proposed mechanism, compound [**4**]+ dissociates to the imidazolium carboxylate and [**3a**]+ at 11.6 kal mol$^{-1}$. The Si-H bond in $Ph_2SiH_2$ is activated by the stannylium center of [**3a**]+ via a **TS1** at 22.5 kcal mol$^{-1}$ to generate intermediate **INT_A** at 18.2 kcal mol$^{-1}$. This is followed by the hydride transfer from the hyper-coordinate silane to the stannylium center, generating ((silylcarbonyl)oxy)imidazolium intermediate (**INT_B**) and the stannyl hydride (**INT_C**) at 15.0 kcal mol$^{-1}$. The next step is a hydride abstraction from **INT_C** by **INT_B**, releasing [**3a**]+ and forming a zwitterionic adduct **INT_D**. In the last step, **INT_D** releases diphenylformoxysilane ($Ph_2SiH(OCHO)$) and free $IMe_4$, which then bonds to the Sn center of [**3a**]+ to generate the [**3**]+.

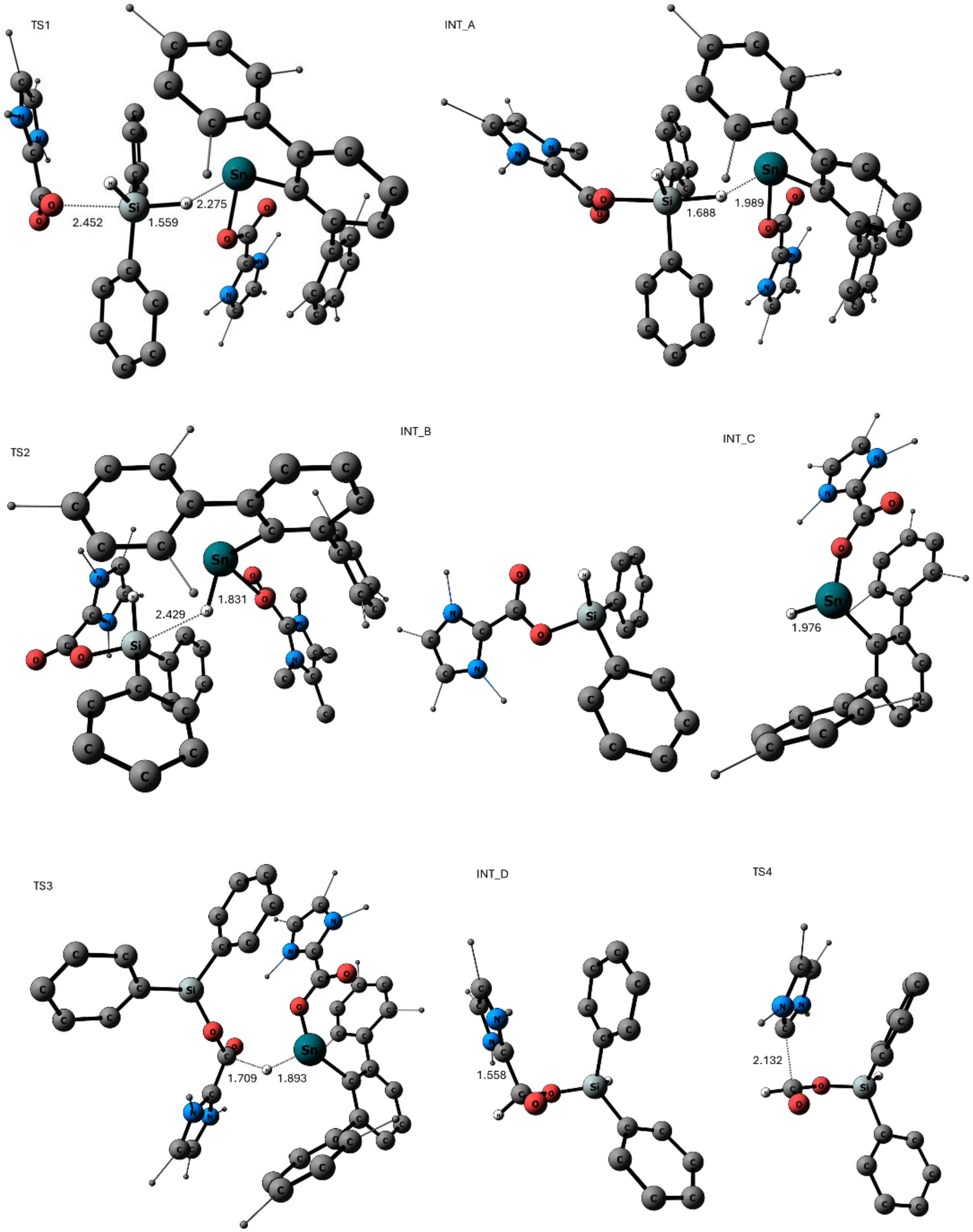


**Figure S59**. Geometries of key intermediates and transition states.

# Cartesian coordinates and energies of the optimized geometries at the r$^2$SCAN-3c level

Calculated energies and coordinates of [**2**]$^+$
Electronic energy ... -1910.38008407 Eh
Total Enthalpy ... -1909.54907147 Eh
Final Gibbs free energy ... -1909.67212994 Eh
CARTESIAN COORDINATES (ANGSTROEM)

| | | | |
|---|---|---|---|
| Sn | 1.871044 | 9.948868 | 3.058533 |
| N | -1.118512 | 10.207896 | 4.729189 |
| N | 2.696946 | 7.297550 | 4.680012 |
| N | 2.796556 | 8.836635 | 6.166870 |
| N | -1.310525 | 10.271967 | 2.600358 |
| C | -0.384233 | 10.244358 | 3.593601 |
| C | -0.548118 | 10.140676 | 6.062437 |
| H | -0.413238 | 9.100421 | 6.382225 |
| H | -1.198793 | 10.660855 | 6.768573 |
| H | 0.411173 | 10.651365 | 6.041013 |
| C | 2.425454 | 8.612786 | 4.879415 |
| C | 3.232073 | 6.705182 | 5.816781 |
| C | 2.739055 | 10.115617 | 6.861134 |
| H | 2.601099 | 10.907065 | 6.122225 |
| H | 3.679255 | 10.290695 | 7.389561 |
| H | 1.924928 | 10.124815 | 7.594302 |
| C | 2.900219 | 11.791546 | 3.829720 |
| C | 4.303630 | 11.707444 | 3.683588 |
| C | -2.482386 | 10.233915 | 4.465463 |
| C | 2.348401 | 13.004503 | 4.289694 |
| C | 0.874714 | 13.224023 | 4.380692 |
| C | 3.287508 | 7.682067 | 6.766564 |
| C | 0.130137 | 13.371672 | 3.189565 |
| C | 0.227812 | 13.360676 | 5.617078 |
| C | 2.552142 | 6.574842 | 3.419231 |
| H | 2.060577 | 7.223436 | 2.692108 |
| H | 1.954777 | 5.671254 | 3.570664 |
| H | 3.540606 | 6.305930 | 3.031370 |
| C | 5.123036 | 12.781455 | 4.038995 |
| H | 6.199902 | 12.691545 | 3.915606 |
| C | -2.604482 | 10.285565 | 3.109043 |
| C | -1.242961 | 13.577646 | 3.261829 |
| H | -1.808148 | 13.713712 | 2.340435 |
| C | 0.972292 | 13.353212 | 6.928594 |
| H | 1.988769 | 12.967985 | 6.831960 |
| H | 0.436429 | 12.768424 | 7.684810 |
| H | 1.057134 | 14.373398 | 7.321996 |
| C | 4.928654 | 10.475521 | 3.102948 |
| C | 4.567935 | 13.958676 | 4.524832 |
| H | 5.206761 | 14.794804 | 4.794268 |
| C | 5.565462 | 9.524805 | 3.917852 |
| C | -1.912859 | 13.651349 | 4.485738 |
| C | -1.027668 | 10.399607 | 1.175767 |
| H | -1.210618 | 11.426655 | 0.842614 |
| H | -1.664271 | 9.716453 | 0.607379 |
| H | 0.018679 | 10.143139 | 0.994690 |
| C | -1.159262 | 13.552326 | 5.648221 |
| H | -1.652323 | 13.656602 | 6.614115 |
| C | 6.029309 | 8.335217 | 3.349510 |
| H | 6.510553 | 7.598706 | 3.991968 |
| C | 5.836833 | 9.780569 | 5.375628 |
| H | 6.877598 | 10.101286 | 5.508708 |
| H | 5.703089 | 8.869582 | 5.968459 |
| H | 5.207353 | 10.576310 | 5.775311 |
| C | 3.188471 | 14.070363 | 4.636419 |
| H | 2.743794 | 15.000092 | 4.984050 |
| C | 4.887469 | 10.272478 | 1.705446 |
| C | 0.816243 | 13.377877 | 1.849347 |
| H | 1.680276 | 14.050449 | 1.855178 |
| H | 0.130096 | 13.703316 | 1.062802 |
| H | 1.205814 | 12.390361 | 1.572060 |
| C | 3.636389 | 5.277056 | 5.860675 |
| H | 2.787864 | 4.607397 | 5.680120 |
| H | 4.050109 | 5.031722 | 6.840131 |
| H | 4.402378 | 5.053642 | 5.109411 |
| C | 4.330321 | 11.321519 | 0.782078 |
| H | 3.233513 | 11.270839 | 0.739239 |
| H | 4.704469 | 11.176218 | -0.234479 |
| H | 4.592096 | 12.330119 | 1.113917 |
| C | -3.806499 | 10.374076 | 2.241519 |
| H | -3.779305 | 11.268632 | 1.608158 |
| H | -4.708940 | 10.428146 | 2.853060 |
| H | -3.904845 | 9.502593 | 1.584141 |
| C | 5.931691 | 8.089060 | 1.983710 |
| C | -3.509119 | 10.250868 | 5.536790 |
| H | -3.405656 | 9.399182 | 6.218243 |
| H | -4.508921 | 10.205382 | 5.101284 |
| H | -3.446553 | 11.171351 | 6.131064 |
| C | 3.765175 | 7.650288 | 8.172238 |
| H | 4.638195 | 8.297576 | 8.318838 |
| H | 4.055911 | 6.635387 | 8.448273 |
| H | 2.988380 | 7.976260 | 8.873118 |
| C | 5.381772 | 9.084402 | 1.174432 |
| H | 5.343794 | 8.933559 | 0.097146 |
| C | -3.401484 | 13.856146 | 4.527637 |
| H | -3.698622 | 14.721230 | 3.925488 |
| H | -3.757103 | 14.018718 | 5.548642 |
| H | -3.929039 | 12.984257 | 4.119616 |
| C | 6.454051 | 6.814186 | 1.379986 |
| H | 7.358050 | 7.004461 | 0.790096 |
| H | 5.720350 | 6.363423 | 0.703041 |
| H | 6.710915 | 6.080862 | 2.149543 |

Calculated energies and coordinates of [**2**][BArF]
Electronic energy ... -5558.27970811 Eh
Total Enthalpy ... -5556.99596624 Eh
Final Gibbs free energy ... -5557.22076880 Eh
CARTESIAN COORDINATES (ANGSTROEM)

| | | | |
|---|---|---|---|
| Sn | 1.831134 | 9.677246 | 3.045045 |
| F | -1.429303 | -3.143716 | 7.834758 |
| F | 1.661373 | -2.003442 | 1.296276 |
| F | -0.210833 | -3.036877 | 6.031564 |
| F | 0.575682 | -2.298085 | 7.919080 |
| F | 2.095163 | -0.304976 | 0.011166 |
| F | 0.573854 | -1.750208 | -0.574505 |
| F | 3.970498 | 3.549019 | 1.733213 |
| F | -3.795816 | 2.014004 | 0.517970 |
| F | 5.480192 | 1.058728 | 7.343180 |
| F | 3.568238 | 0.208003 | 7.948186 |
| N | -1.017001 | 10.183730 | 4.881356 |
| N | 2.506524 | 7.158114 | 4.925875 |
| F | -1.884278 | 7.367920 | 6.991424 |
| F | -4.461092 | 0.545034 | 1.975717 |
| N | 2.826758 | 8.841472 | 6.213399 |
| N | -1.382452 | 10.056532 | 2.777060 |
| F | 3.994614 | 2.306764 | 8.333013 |
| C | -0.442598 | 1.206872 | 3.091818 |
| C | -0.533924 | 3.527784 | 4.477973 |
| C | -0.847669 | 1.104121 | 5.645884 |
| C | 1.550421 | 2.003177 | 4.740413 |
| C | -0.557088 | 4.272264 | 5.667695 |
| H | -0.290628 | 3.782607 | 6.602568 |
| C | -1.096450 | -1.002997 | 6.860135 |
| C | 0.448037 | 0.411652 | 2.365197 |
| H | 1.477052 | 0.306859 | 2.702047 |
| C | 0.056112 | -0.266960 | 1.209662 |
| C | 2.171433 | 1.615059 | 5.930417 |
| H | 1.567897 | 1.232840 | 6.750149 |
| C | -0.379008 | 10.094626 | 3.691309 |
| C | -1.999615 | 1.543793 | 6.303733 |
| H | -2.396577 | 2.535283 | 6.097401 |
| C | -1.760012 | 1.259695 | 2.611760 |
| H | -2.505662 | 1.831798 | 3.162139 |
| F | 5.627633 | 2.328914 | 2.447170 |
| C | 2.391537 | 2.517701 | 3.736260 |
| H | 1.951914 | 2.859050 | 2.801899 |

| | | | |
|---|---|---|---|
| C | -0.429613 | -0.203864 | 5.937910 |
| H | 0.436793 | -0.614405 | 5.422051 |
| C | -0.883911 | 4.217650 | 3.309426 |
| H | -0.881481 | 3.689641 | 2.360086 |
| F | -4.842615 | 1.665024 | 6.988063 |
| C | -2.675395 | 0.742437 | 7.225922 |
| C | -1.244241 | -0.176599 | 0.732548 |
| H | -1.547245 | -0.698079 | -0.167098 |
| C | -0.955044 | 5.608239 | 5.695491 |
| C | 3.558321 | 1.684428 | 6.095557 |
| F | -1.322061 | 5.540404 | 8.035196 |
| C | -0.549820 | -2.369476 | 7.159343 |
| C | -0.334377 | 10.258999 | 6.162116 |
| H | -0.135465 | 9.260942 | 6.563290 |
| H | -0.946540 | 10.823541 | 6.868563 |
| H | 0.597892 | 10.797094 | 6.008493 |
| C | -1.265813 | 5.558189 | 3.332760 |
| C | -2.151347 | 0.594645 | 1.454684 |
| C | 2.383233 | 8.508485 | 4.971737 |
| C | 1.085293 | -1.083574 | 0.483858 |
| F | 5.285621 | 4.300048 | 3.300860 |
| C | 3.014300 | 6.648614 | 6.112130 |
| C | 2.939638 | 10.186352 | 6.754217 |
| H | 2.820259 | 10.902643 | 5.939740 |
| H | 3.928967 | 10.323575 | 7.199241 |
| H | 2.185226 | 10.359561 | 7.530086 |
| C | -2.228322 | -0.536733 | 7.524636 |
| H | -2.749175 | -1.158354 | 8.242641 |
| C | 2.932132 | 11.564228 | 3.614549 |
| C | 4.152710 | 1.302169 | 7.420095 |
| C | 4.374310 | 2.155965 | 5.079714 |
| H | 5.451683 | 2.201080 | 5.202087 |
| F | -3.879445 | -0.093987 | -0.024332 |
| C | -1.312320 | 6.272478 | 4.525286 |
| H | -1.643876 | 7.306332 | 4.543905 |
| B | -0.066451 | 1.959058 | 4.493464 |
| C | 3.769246 | 2.588108 | 3.898423 |
| C | 4.321477 | 11.427746 | 3.390832 |
| F | 0.216756 | 6.895649 | 7.314241 |
| C | -2.397407 | 10.217507 | 4.726397 |
| F | -3.601683 | 2.432532 | 8.598649 |
| C | 2.448429 | 12.821189 | 4.035581 |
| C | 0.989578 | 13.105546 | 4.179646 |
| C | 3.212959 | 7.717760 | 6.933390 |
| C | 0.182189 | 13.157443 | 3.022002 |
| C | 0.415095 | 13.389342 | 5.427982 |
| F | -4.473190 | 0.441386 | 8.752374 |
| C | 2.229799 | 6.306986 | 3.769419 |
| H | 1.682969 | 6.880737 | 3.020558 |
| H | 1.627507 | 5.448268 | 4.078720 |
| H | 3.172532 | 5.957688 | 3.334257 |
| C | 5.196096 | 12.484717 | 3.655657 |
| H | 6.260560 | 12.349014 | 3.477037 |
| C | -2.629008 | 10.147356 | 3.384394 |
| C | -1.179199 | 13.413226 | 3.145776 |
| H | -1.791574 | 13.466260 | 2.246253 |
| C | 1.229703 | 13.475691 | 6.694413 |
| H | 2.198927 | 12.982928 | 6.601825 |
| H | 0.686655 | 13.040999 | 7.540881 |
| H | 1.430869 | 14.523412 | 6.949541 |
| C | 4.870409 | 10.170186 | 2.785223 |
| C | -3.895031 | 1.307394 | 7.892025 |
| C | 4.709479 | 13.702243 | 4.115695 |
| H | 5.390439 | 14.525234 | 4.314035 |
| C | 5.505947 | 9.183209 | 3.555312 |
| C | -1.778008 | 13.626115 | 4.389055 |
| C | -1.213559 | 10.045622 | 1.329249 |
| H | -1.386458 | 11.047877 | 0.921770 |
| H | -1.916452 | 9.340841 | 0.880063 |
| H | -0.196782 | 9.725174 | 1.091386 |
| C | -0.995208 | 6.336925 | 7.003714 |
| C | -1.611605 | 6.259303 | 2.055467 |
| C | -0.962244 | 13.625271 | 5.513517 |
| H | -1.399263 | 13.831641 | 6.489890 |
| C | 5.920774 | 7.995890 | 2.943747 |
| H | 6.396010 | 7.228504 | 3.553148 |
| C | 5.818745 | 9.386511 | 5.013723 |

| | | | |
|---|---|---|---|
| H | 6.897654 | 9.533571 | 5.146898 |
| H | 5.536513 | 8.508923 | 5.606049 |
| H | 5.319358 | 10.269550 | 5.413805 |
| C | -3.566528 | 0.753856 | 0.980522 |
| C | 3.341566 | 13.871831 | 4.284044 |
| H | 2.947569 | 14.835571 | 4.599223 |
| C | 4.649751 | 3.174993 | 2.837654 |
| C | 4.762126 | 9.992946 | 1.387272 |
| C | 0.783374 | 12.994521 | 1.651898 |
| H | 1.724239 | 13.546468 | 1.564177 |
| H | 0.093722 | 13.351471 | 0.881658 |
| H | 1.022190 | 11.947540 | 1.426677 |
| C | 3.238303 | 5.201281 | 6.329781 |
| H | 2.317772 | 4.621940 | 6.190289 |
| H | 3.598545 | 5.015362 | 7.342828 |
| H | 3.979251 | 4.802102 | 5.629851 |
| C | 4.165954 | 11.062913 | 0.514362 |
| H | 3.071437 | 11.077470 | 0.603657 |
| H | 4.410049 | 10.884115 | -0.535911 |
| H | 4.521383 | 12.058882 | 0.795870 |
| C | -3.896801 | 10.188935 | 2.612684 |
| H | -3.918507 | 11.043345 | 1.925207 |
| H | -4.746121 | 10.286113 | 3.291569 |
| H | -4.045379 | 9.278672 | 2.022175 |
| C | 5.770438 | 7.783778 | 1.577624 |
| C | -3.334784 | 10.355671 | 5.868999 |
| H | -3.177945 | 9.576125 | 6.621402 |
| H | -4.366120 | 10.276690 | 5.519753 |
| H | -3.221471 | 11.331315 | 6.359328 |
| C | 3.715533 | 7.790815 | 8.328214 |
| H | 4.659947 | 8.344881 | 8.395538 |
| H | 3.893387 | 6.784848 | 8.712074 |
| H | 2.993032 | 8.277650 | 8.993565 |
| C | 5.211561 | 8.809407 | 0.812386 |
| H | 5.123390 | 8.680303 | -0.264760 |
| C | -3.260041 | 13.856490 | 4.491553 |
| H | -3.591329 | 14.628053 | 3.788519 |
| H | -3.550331 | 14.167708 | 5.498851 |
| H | -3.812986 | 12.939715 | 4.249030 |
| C | 6.204042 | 6.498001 | 0.930451 |
| H | 6.713405 | 6.688327 | -0.019636 |
| H | 5.339306 | 5.859361 | 0.714413 |
| H | 6.876437 | 5.929493 | 1.577521 |
| F | -2.637165 | 7.142325 | 2.221190 |
| F | -1.966568 | 5.420241 | 1.069681 |
| F | -0.559219 | 6.996253 | 1.586277 |

Calculated energies and coordinates of [**2a**]$^{+}$
Electronic energy ... -1526.98783236 Eh
Total Enthalpy ... -1526.35173142 Eh
Final Gibbs free energy ... -1526.45419801 Eh
CARTESIAN COORDINATES (ANGSTROEM)

| | | | |
|---|---|---|---|
| Sn | -1.475789 | 9.810765 | 2.693709 |
| N | -0.793559 | 12.664716 | 4.335156 |
| N | -2.512344 | 11.669422 | 5.145700 |
| C | -0.720087 | 10.999834 | 0.995815 |
| C | -1.650132 | 11.882307 | 0.433843 |
| C | -1.324536 | 12.606490 | -0.712650 |
| H | -2.045262 | 13.290540 | -1.153874 |
| C | -0.074173 | 12.427506 | -1.300126 |
| H | 0.180427 | 12.977653 | -2.201315 |
| C | 0.848728 | 11.539175 | -0.752048 |
| H | 1.817336 | 11.400704 | -1.226236 |
| C | 0.533179 | 10.814882 | 0.401394 |
| C | -2.958553 | 11.966623 | 1.140832 |
| C | -3.319249 | 13.120010 | 1.865953 |
| C | -4.474042 | 13.099966 | 2.643220 |
| H | -4.744082 | 13.994269 | 3.202804 |
| C | -5.290212 | 11.969235 | 2.735513 |
| C | -4.930750 | 10.840088 | 2.010300 |
| H | -5.572820 | 9.961200 | 2.033601 |
| C | -3.792341 | 10.820053 | 1.187797 |
| C | -2.478869 | 14.366912 | 1.820695 |
| H | -1.417729 | 14.132948 | 1.706942 |
| H | -2.624232 | 14.965530 | 2.724474 |
| H | -2.755870 | 14.993332 | 0.964424 |

| | | | |
|---|---|---|---|
| C | -6.552435 | 11.996877 | 3.552803 |
| H | -6.461929 | 12.666805 | 4.413662 |
| H | -6.825989 | 10.999611 | 3.909478 |
| H | -7.390091 | 12.365790 | 2.949142 |
| C | -3.639446 | 9.674797 | 0.210004 |
| H | -3.939156 | 8.721924 | 0.655953 |
| H | -2.627553 | 9.582890 | -0.187782 |
| H | -4.303446 | 9.856781 | -0.643971 |
| C | 1.477062 | 9.854596 | 1.041961 |
| C | 2.623381 | 10.307261 | 1.724475 |
| C | 3.402537 | 9.386076 | 2.423088 |
| H | 4.285469 | 9.740396 | 2.952694 |
| C | 3.098531 | 8.025284 | 2.444580 |
| C | 2.000890 | 7.586494 | 1.708157 |
| H | 1.783706 | 6.521455 | 1.659525 |
| C | 1.189183 | 8.473000 | 0.998274 |
| C | 3.079476 | 11.743521 | 1.667009 |
| H | 3.535693 | 12.049640 | 2.613581 |
| H | 2.273088 | 12.436137 | 1.416869 |
| H | 3.845699 | 11.863030 | 0.891261 |
| C | 3.947179 | 7.062468 | 3.226975 |
| H | 3.736512 | 7.141612 | 4.300183 |
| H | 5.012510 | 7.271498 | 3.089729 |
| H | 3.759148 | 6.028607 | 2.927125 |
| C | 0.086857 | 7.924137 | 0.125941 |
| H | -0.884607 | 7.885093 | 0.638616 |
| H | 0.320903 | 6.899772 | -0.175385 |
| H | -0.047155 | 8.528706 | -0.775130 |
| C | -1.560832 | 11.559915 | 4.187226 |
| C | -1.259981 | 13.470276 | 5.364610 |
| C | -2.360229 | 12.839930 | 5.875692 |
| C | 0.440758 | 12.921909 | 3.602119 |
| H | 1.308091 | 12.728682 | 4.242219 |
| H | 0.469797 | 13.960283 | 3.262043 |
| H | 0.473187 | 12.261925 | 2.734553 |
| C | -0.601038 | 14.745729 | 5.742290 |
| H | 0.447761 | 14.590890 | 6.019304 |
| H | -1.107040 | 15.195137 | 6.598215 |
| H | -0.625327 | 15.471691 | 4.921238 |
| C | -3.282650 | 13.223684 | 6.973860 |
| H | -2.983272 | 14.182371 | 7.399851 |
| H | -3.281394 | 12.484739 | 7.782963 |
| H | -4.314037 | 13.325688 | 6.616790 |
| C | -3.553526 | 10.686212 | 5.430619 |
| H | -3.419248 | 10.282830 | 6.439218 |
| H | -3.488561 | 9.871222 | 4.708178 |
| H | -4.540775 | 11.147280 | 5.349780 |

Calculated energies and coordinates of [**2a**][BArF]
Electronic energy ... -5174.89548763 Eh
Total Enthalpy ... -5173.80705990 Eh
Final Gibbs free energy .... -5174.01250912 Eh
CARTESIAN COORDINATES (ANGSTROEM)

| | | | |
|---|---|---|---|
| Sn | -2.758607 | 1.755398 | 0.310386 |
| F | 5.986825 | 1.860066 | -2.036746 |
| F | 1.316993 | -7.187328 | -0.720350 |
| F | 7.623358 | 0.504508 | -2.518411 |
| F | 5.723919 | 0.344825 | -3.572538 |
| F | 2.872156 | -5.928611 | -1.582083 |
| F | 0.805252 | -5.695846 | -2.222838 |
| F | 1.688890 | -1.367739 | -4.993973 |
| F | -1.583282 | -5.352068 | 2.813323 |
| F | -1.423522 | 3.002643 | -1.774686 |
| F | 0.326231 | 3.711825 | -2.855300 |
| N | -2.389849 | -1.171813 | -0.998717 |
| F | 4.152290 | 1.628982 | 5.813774 |
| F | -1.218474 | -3.334380 | 3.555063 |
| N | -3.198825 | 0.064530 | -2.551610 |
| F | 0.364365 | 3.634461 | -0.681874 |
| C | 1.711991 | -2.608813 | 0.714484 |
| C | 2.195264 | -0.208633 | 1.813764 |
| C | 3.863231 | -1.199125 | -0.035854 |
| C | 1.449278 | -0.393504 | -0.781102 |
| C | 3.302754 | 0.119504 | 2.601803 |
| H | 4.297982 | -0.169279 | 2.272739 |
| C | 5.772734 | -0.386259 | -1.322460 |
| C | 1.971225 | -3.654871 | -0.186644 |
| H | 2.682038 | -3.500902 | -0.995154 |
| C | 1.364161 | -4.904085 | -0.068507 |
| C | 1.054608 | 0.954733 | -0.753001 |
| H | 1.236032 | 1.545676 | 0.139591 |
| C | -2.823906 | 0.081904 | -1.252278 |
| C | 4.732348 | -2.137485 | 0.537308 |
| H | 4.349440 | -2.839291 | 1.276391 |
| C | 0.877720 | -2.929870 | 1.793915 |
| H | 0.677434 | -2.182557 | 2.555639 |
| F | -0.339784 | -0.624837 | -5.254271 |
| C | 1.203169 | -1.075537 | -1.980843 |
| H | 1.490624 | -2.118719 | -2.075820 |
| C | 4.424421 | -0.324702 | -0.972556 |
| H | 3.799869 | 0.428622 | -1.447899 |
| C | 0.947523 | 0.208384 | 2.306717 |
| H | 0.050583 | -0.001140 | 1.729114 |
| F | 6.463998 | -4.490592 | 0.644244 |
| C | 6.080184 | -2.201090 | 0.190629 |
| C | 0.495168 | -5.180983 | 0.981231 |
| H | 0.020840 | -6.151677 | 1.073069 |
| C | 3.173241 | 0.803882 | 3.812116 |
| C | 0.454547 | 1.566776 | -1.851827 |
| F | 5.204947 | 0.042640 | 4.754379 |
| C | 6.284248 | 0.574920 | -2.355889 |
| C | -1.839759 | -1.645515 | 0.266593 |
| H | -2.306235 | -2.593469 | 0.545015 |
| H | -2.051051 | -0.921363 | 1.055667 |
| H | -0.759797 | -1.788203 | 0.172351 |
| C | 0.810278 | 0.874081 | 3.521029 |
| C | 0.275025 | -4.180230 | 1.921082 |
| C | 1.599870 | -5.933463 | -1.136218 |
| F | 0.017243 | -2.482274 | -4.168783 |
| C | 6.618631 | -1.326910 | -0.747314 |
| H | 7.665399 | -1.378122 | -1.020694 |
| C | -4.733469 | 2.635061 | -0.234039 |
| C | -0.027133 | 2.979096 | -1.781786 |
| C | 0.240641 | 0.873695 | -3.039767 |
| H | -0.195238 | 1.365461 | -3.902808 |
| F | 0.088988 | -4.967904 | 4.151002 |
| C | 1.928052 | 1.184754 | 4.291698 |
| H | 1.827959 | 1.699405 | 5.240150 |
| B | 2.304454 | -1.096618 | 0.444863 |
| C | 0.625261 | -0.457708 | -3.089664 |
| C | -5.800018 | 1.828893 | 0.189886 |
| F | 5.173629 | 2.057424 | 3.938359 |
| C | -2.491882 | -1.979284 | -2.121164 |
| F | 6.974175 | -3.064119 | 2.203168 |
| C | -4.998355 | 3.912620 | -0.751493 |
| C | -3.875973 | 4.809035 | -1.147953 |
| C | -3.040579 | 5.358322 | -0.152821 |
| C | -3.635031 | 5.106622 | -2.500489 |
| F | 8.216904 | -3.216146 | 0.420379 |
| C | -7.119256 | 2.262421 | 0.056712 |
| H | -7.935319 | 1.626230 | 0.391773 |
| C | -3.006724 | -1.191823 | -3.113292 |
| C | -1.946807 | 6.131219 | -0.535432 |
| H | -1.304832 | 6.552673 | 0.236288 |
| C | -4.549672 | 4.638509 | -3.605200 |
| H | -5.171819 | 3.792610 | -3.305160 |
| H | -3.974489 | 4.360522 | -4.494628 |
| H | -5.233311 | 5.442704 | -3.904578 |
| C | -5.469448 | 0.513408 | 0.813267 |
| C | 6.938734 | -3.237077 | 0.858014 |
| C | -7.377748 | 3.517948 | -0.484235 |
| H | -8.401897 | 3.867070 | -0.581590 |
| C | -5.691970 | -0.687554 | 0.104107 |
| C | -1.657737 | 6.385503 | -1.875484 |
| C | -3.674770 | 1.226295 | -3.289320 |
| H | -2.899443 | 1.592548 | -3.970807 |
| H | -3.926353 | 2.011288 | -2.574920 |
| H | -4.567547 | 0.966400 | -3.865611 |
| C | 4.420239 | 1.131986 | 4.584446 |
| C | -0.550349 | 1.286500 | 3.992281 |
| C | -2.524664 | 5.880968 | -2.839842 |
| H | -2.331233 | 6.091011 | -3.890451 |
| C | -5.355018 | -1.898167 | 0.696237 |

| | | | |
|---|---|---|---|
| H | -5.531132 | -2.821455 | 0.145472 |
| C | -6.296942 | -0.671791 | -1.273899 |
| H | -5.988121 | 0.213057 | -1.836154 |
| H | -7.392062 | -0.644584 | -1.220713 |
| H | -6.013217 | -1.568920 | -1.832165 |
| C | -0.602214 | -4.458673 | 3.105779 |
| C | -6.325449 | 4.338372 | -0.878086 |
| H | -6.527452 | 5.329485 | -1.277644 |
| C | 0.498996 | -1.228551 | -4.371329 |
| C | -4.913203 | 0.466323 | 2.111918 |
| C | -3.331029 | 5.182317 | 1.317757 |
| H | -2.984627 | 6.056176 | 1.876736 |
| H | -2.812735 | 4.313140 | 1.746075 |
| H | -4.400183 | 5.052876 | 1.505366 |
| C | -4.828080 | 1.685544 | 2.999366 |
| H | -5.682245 | 1.686459 | 3.687872 |
| H | -4.867289 | 2.622176 | 2.440072 |
| H | -3.918001 | 1.669385 | 3.606010 |
| C | -3.368640 | -1.505098 | -4.518368 |
| H | -2.987544 | -2.490823 | -4.791078 |
| H | -2.937378 | -0.781803 | -5.217049 |
| H | -4.456492 | -1.509695 | -4.661925 |
| C | -4.797724 | -1.966742 | 1.978320 |
| C | -2.085012 | -3.406227 | -2.104075 |
| H | -2.164996 | -3.836851 | -3.103011 |
| H | -2.715535 | -3.996076 | -1.427654 |
| H | -1.045568 | -3.521786 | -1.775964 |
| C | -4.568563 | -0.779082 | 2.659914 |
| H | -4.140888 | -0.808698 | 3.659870 |
| C | -0.436344 | 7.173121 | -2.259544 |
| H | -0.504096 | 7.541276 | -3.286755 |
| H | 0.462300 | 6.548368 | -2.188422 |
| H | -0.290427 | 8.029926 | -1.594360 |
| C | -4.485619 | -3.297962 | 2.601492 |
| H | -5.411600 | -3.814974 | 2.880272 |
| H | -3.875293 | -3.190085 | 3.500081 |
| H | -3.950725 | -3.955161 | 1.907378 |
| F | -0.916680 | 2.508103 | 3.513023 |
| F | -0.634635 | 1.361976 | 5.337589 |
| F | -1.518474 | 0.416526 | 3.584352 |

Calculated energies and coordinates of [**3**]$^{+}$
Electronic energy ... -2098.97915889 Eh
Total Enthalpy ... -2098.13057677 Eh
Final Gibbs free energy ... -2098.25970783 Eh
CARTESIAN COORDINATES (ANGSTROEM)

| | | | |
|---|---|---|---|
| Sn | -1.421963 | 9.642426 | 3.138694 |
| O | 0.638493 | 9.427200 | 4.079652 |
| C | 0.986204 | 8.193509 | 4.174196 |
| O | 0.344996 | 7.196885 | 3.851221 |
| N | -0.770639 | 12.619960 | 4.605180 |
| N | -2.651882 | 11.819946 | 5.237642 |
| N | 3.213466 | 8.903305 | 5.222179 |
| N | 2.953730 | 6.774601 | 4.882305 |
| C | -0.681758 | 10.808159 | 1.348066 |
| C | -1.658530 | 11.655387 | 0.781464 |
| C | -1.420342 | 12.334429 | -0.415504 |
| H | -2.197323 | 12.973869 | -0.828372 |
| C | -0.213104 | 12.176151 | -1.082956 |
| H | -0.032260 | 12.692365 | -2.021346 |
| C | 0.750640 | 11.330218 | -0.553223 |
| H | 1.687210 | 11.174848 | -1.084080 |
| C | 0.528659 | 10.641112 | 0.646863 |
| C | -2.992247 | 11.833645 | 1.435469 |
| C | -3.303596 | 13.043598 | 2.086343 |
| C | -4.549417 | 13.196250 | 2.692185 |
| H | -4.780255 | 14.135895 | 3.192591 |
| C | -5.511471 | 12.187042 | 2.656676 |
| C | -5.200501 | 11.006939 | 1.987828 |
| H | -5.946585 | 10.216311 | 1.925321 |
| C | -3.967136 | 10.818119 | 1.358241 |
| C | -2.336183 | 14.197224 | 2.104544 |
| H | -1.298930 | 13.856426 | 2.091419 |
| H | -2.502416 | 14.828652 | 2.982564 |
| H | -2.469882 | 14.826510 | 1.216117 |
| C | -6.862866 | 12.382129 | 3.289172 |
| H | -6.837283 | 13.153625 | 4.064828 |
| H | -7.237320 | 11.454202 | 3.732880 |
| H | -7.597979 | 12.700227 | 2.540348 |
| C | -3.770199 | 9.577847 | 0.523109 |
| H | -4.195031 | 8.698467 | 1.015968 |
| H | -2.720087 | 9.382461 | 0.299765 |
| H | -4.287518 | 9.699936 | -0.436788 |
| C | 1.615632 | 9.712444 | 1.083350 |
| C | 2.853889 | 10.224164 | 1.515306 |
| C | 3.882329 | 9.343218 | 1.854388 |
| H | 4.838427 | 9.749721 | 2.184269 |
| C | 3.735620 | 7.962962 | 1.725690 |
| C | 2.514779 | 7.474972 | 1.267530 |
| H | 2.388478 | 6.403187 | 1.122017 |
| C | 1.450979 | 8.321407 | 0.949574 |
| C | 3.126541 | 11.706729 | 1.567421 |
| H | 3.860244 | 11.947972 | 2.344367 |
| H | 2.219301 | 12.291420 | 1.731685 |
| H | 3.548420 | 12.053683 | 0.616348 |
| C | 4.889609 | 7.033390 | 1.990710 |
| H | 4.546139 | 6.052397 | 2.336666 |
| H | 5.577485 | 7.445642 | 2.736069 |
| H | 5.466766 | 6.863131 | 1.073889 |
| C | 0.171416 | 7.718769 | 0.435773 |
| H | -0.526853 | 7.508748 | 1.257527 |
| H | 0.370023 | 6.765543 | -0.062210 |
| H | -0.332143 | 8.384187 | -0.269810 |
| C | -1.574876 | 11.540412 | 4.468320 |
| C | -1.340906 | 13.580500 | 5.433793 |
| C | -2.542586 | 13.071789 | 5.832374 |
| C | 0.535434 | 12.758573 | 3.980947 |
| H | 1.317938 | 12.816390 | 4.745795 |
| H | 0.575642 | 13.664825 | 3.368379 |
| H | 0.704373 | 11.888046 | 3.347441 |
| C | -0.678999 | 14.876847 | 5.725857 |
| H | 0.299137 | 14.737782 | 6.200538 |
| H | -1.293828 | 15.471845 | 6.403454 |
| H | -0.524692 | 15.464580 | 4.812875 |
| C | -3.606504 | 13.638512 | 6.698927 |
| H | -3.322322 | 14.633083 | 7.046912 |
| H | -3.789191 | 13.015484 | 7.581823 |
| H | -4.554289 | 13.731626 | 6.155748 |
| C | -3.798846 | 10.940188 | 5.440460 |
| H | -3.872540 | 10.657052 | 6.495516 |
| H | -3.669034 | 10.042324 | 4.833581 |
| H | -4.716368 | 11.444098 | 5.126112 |
| C | 2.353452 | 7.975868 | 4.755008 |
| C | 4.379901 | 8.287622 | 5.637665 |
| C | 4.213636 | 6.942061 | 5.425996 |
| C | 2.979172 | 10.348676 | 5.255140 |
| H | 2.086136 | 10.566068 | 5.843185 |
| H | 2.832327 | 10.711599 | 4.236744 |
| H | 3.849405 | 10.825745 | 5.705831 |
| C | 5.540032 | 9.030079 | 6.194113 |
| H | 6.339380 | 8.332778 | 6.447475 |
| H | 5.276606 | 9.574138 | 7.107861 |
| H | 5.946065 | 9.747852 | 5.472677 |
| C | 5.145165 | 5.814963 | 5.685037 |
| H | 4.711407 | 5.077718 | 6.369012 |
| H | 6.062222 | 6.188275 | 6.142186 |
| H | 5.423809 | 5.300954 | 4.758227 |
| C | 2.387697 | 5.479160 | 4.483315 |
| H | 1.505265 | 5.253652 | 5.083910 |
| H | 3.151789 | 4.716591 | 4.632537 |
| H | 2.086816 | 5.520183 | 3.436308 |

Calculated energies and coordinates of [**3**][BArF]
Electronic energy ... -5746.88680094 Eh
Total Enthalpy ... -5745.58602844 Eh
Final Gibbs free energy ... -5745.81592677 Eh
CARTESIAN COORDINATES (ANGSTROEM)

| | | | |
|---|---|---|---|
| Sn | -2.865784 | -0.624782 | -0.004643 |
| O | -1.415617 | -2.256964 | -0.671147 |
| C | -0.687717 | -2.561844 | 0.341950 |
| O | -0.800680 | -2.121438 | 1.485101 |
| N | -3.192692 | -0.941812 | -3.357695 |

| | | | |
|---|---|---|---|
| N | -2.716948 | 1.064572 | -2.786159 |
| N | 0.569378 | -4.415602 | -0.924000 |
| N | 1.315941 | -3.906454 | 1.049099 |
| C | -4.728598 | -1.921435 | -0.117229 |
| C | -5.896263 | -1.160525 | -0.334105 |
| C | -7.154620 | -1.764155 | -0.401020 |
| H | -8.033402 | -1.144834 | -0.567833 |
| C | -7.281310 | -3.136817 | -0.233767 |
| H | -8.259128 | -3.608573 | -0.276398 |
| C | -6.148562 | -3.897207 | 0.023000 |
| H | -6.239277 | -4.967477 | 0.197239 |
| C | -4.878663 | -3.307328 | 0.090429 |
| C | -5.815127 | 0.328016 | -0.454941 |
| C | -5.966206 | 0.958847 | -1.701512 |
| C | -5.854067 | 2.348221 | -1.782672 |
| H | -5.962160 | 2.828922 | -2.754493 |
| C | -5.621686 | 3.131625 | -0.654828 |
| C | -5.536188 | 2.495878 | 0.583336 |
| H | -5.390994 | 3.095050 | 1.479270 |
| C | -5.635940 | 1.111380 | 0.706484 |
| C | -6.291285 | 0.173834 | -2.944504 |
| H | -5.918695 | -0.850627 | -2.883042 |
| H | -5.870681 | 0.655384 | -3.832960 |
| H | -7.377779 | 0.110102 | -3.084615 |
| C | -5.454073 | 4.622346 | -0.759030 |
| H | -5.744975 | 4.991496 | -1.746667 |
| H | -4.410361 | 4.910378 | -0.580271 |
| H | -6.055855 | 5.142692 | -0.006922 |
| C | -5.594467 | 0.482719 | 2.074757 |
| H | -4.619737 | 0.024455 | 2.284978 |
| H | -6.345297 | -0.307499 | 2.170582 |
| H | -5.771270 | 1.235824 | 2.846703 |
| C | -3.747366 | -4.207556 | 0.471142 |
| C | -3.296633 | -5.225310 | -0.387812 |
| C | -2.280957 | -6.084923 | 0.040384 |
| H | -1.947738 | -6.880015 | -0.627473 |
| C | -1.715115 | -5.978839 | 1.307740 |
| C | -2.197404 | -4.986849 | 2.158996 |
| H | -1.787445 | -4.896589 | 3.163539 |
| C | -3.206233 | -4.107858 | 1.769160 |
| C | -3.888537 | -5.451582 | -1.757539 |
| H | -4.371985 | -6.434345 | -1.808867 |
| H | -3.111415 | -5.439556 | -2.532060 |
| H | -4.640604 | -4.703331 | -2.010440 |
| C | -0.662723 | -6.948211 | 1.776498 |
| H | 0.031720 | -6.472084 | 2.476610 |
| H | -0.084360 | -7.352874 | 0.939552 |
| H | -1.119110 | -7.796381 | 2.301493 |
| C | -3.714090 | -3.092282 | 2.754149 |
| H | -3.238952 | -2.117952 | 2.594591 |
| H | -3.475949 | -3.395430 | 3.776711 |
| H | -4.795034 | -2.952643 | 2.664578 |
| C | -2.930665 | -0.170577 | -2.278552 |
| C | -3.150815 | -0.203242 | -4.533128 |
| C | -2.851609 | 1.076666 | -4.168200 |
| C | -3.456171 | -2.369700 | -3.304143 |
| H | -4.460675 | -2.591465 | -3.681858 |
| H | -3.389441 | -2.692630 | -2.265940 |
| H | -2.719973 | -2.910869 | -3.907987 |
| C | -3.382222 | -0.812566 | -5.866196 |
| H | -3.325715 | -0.049220 | -6.644460 |
| H | -4.369485 | -1.285709 | -5.930482 |
| H | -2.628833 | -1.576493 | -6.093895 |
| C | -2.632341 | 2.300475 | -4.978340 |
| H | -1.596379 | 2.649972 | -4.901681 |
| H | -3.281834 | 3.121753 | -4.654230 |
| H | -2.842542 | 2.100155 | -6.030665 |
| C | -2.355799 | 2.247215 | -2.010975 |
| H | -1.397606 | 2.641844 | -2.359129 |
| H | -2.271132 | 1.975662 | -0.956536 |
| H | -3.128465 | 3.015160 | -2.112300 |
| C | 0.392827 | -3.578042 | 0.118523 |
| C | 1.599757 | -5.290929 | -0.647708 |
| C | 2.071901 | -4.970345 | 0.598507 |
| C | -0.195892 | -4.463645 | -2.167199 |
| H | -0.585764 | -5.475793 | -2.303715 |
| H | 0.458141 | -4.201393 | -3.003999 |

| | | | |
|---|---|---|---|
| H | -1.010256 | -3.747092 | -2.099732 |
| C | 2.022039 | -6.341897 | -1.605113 |
| H | 2.284294 | -5.911646 | -2.577489 |
| H | 1.231446 | -7.084828 | -1.765537 |
| H | 2.903058 | -6.861960 | -1.227367 |
| C | 3.140587 | -5.611773 | 1.404350 |
| H | 3.581891 | -6.435364 | 0.841095 |
| H | 2.740233 | -6.020089 | 2.339336 |
| H | 3.944448 | -4.912838 | 1.651616 |
| C | 1.547819 | -3.215101 | 2.325498 |
| H | 1.602932 | -2.139908 | 2.153466 |
| H | 2.492553 | -3.569886 | 2.737284 |
| H | 0.725149 | -3.412077 | 3.015743 |
| B | 2.126787 | 1.327969 | 0.665634 |
| F | -2.775187 | 3.818338 | 1.572214 |
| F | -1.310868 | 4.844404 | 2.811311 |
| F | -2.241310 | 5.884516 | 1.137946 |
| F | -0.088939 | 4.446043 | -3.674403 |
| F | 2.040294 | 4.619189 | -3.252376 |
| F | 0.688456 | 6.243775 | -2.726873 |
| F | 4.702486 | -3.155413 | 0.080794 |
| F | 5.545443 | -2.320991 | -1.739832 |
| F | 4.173103 | -4.002253 | -1.860490 |
| F | -0.339672 | -1.760731 | -3.815732 |
| F | 1.417459 | -1.138225 | -4.925984 |
| F | 0.111698 | 0.358713 | -4.035059 |
| F | 7.171226 | 0.988062 | -1.382782 |
| F | 6.256979 | 2.512636 | -2.633513 |
| F | 7.895364 | 3.041232 | -1.297980 |
| C | 5.315360 | 4.779615 | 2.695817 |
| F | 6.630428 | 4.915633 | 2.990982 |
| F | 4.671724 | 4.503349 | 3.852069 |
| F | 4.880872 | 6.001616 | 2.288656 |
| F | 4.977481 | 0.023598 | 4.865431 |
| F | 4.342872 | -2.030308 | 4.523917 |
| F | 3.653393 | -0.957569 | 6.289580 |
| C | -1.001536 | -0.527445 | 4.380510 |
| F | -1.202916 | -1.865824 | 4.543022 |
| F | -1.983974 | -0.067059 | 3.581653 |
| F | -1.192374 | 0.027715 | 5.605815 |
| C | 1.105229 | 2.535910 | 0.235833 |
| C | 0.111681 | 3.094098 | 1.043317 |
| H | -0.078494 | 2.686894 | 2.032165 |
| C | -0.642926 | 4.198522 | 0.631731 |
| C | -0.421380 | 4.802598 | -0.595287 |
| H | -0.993627 | 5.669959 | -0.905467 |
| C | 0.583975 | 4.277047 | -1.408406 |
| C | 1.321735 | 3.173115 | -1.002638 |
| H | 2.118252 | 2.812518 | -1.649135 |
| C | -1.731240 | 4.695553 | 1.536528 |
| C | 0.822689 | 4.899129 | -2.752201 |
| C | 2.125767 | 0.108010 | -0.432413 |
| C | 3.082677 | -0.912983 | -0.314883 |
| H | 3.740610 | -0.923136 | 0.551719 |
| C | 3.265397 | -1.874444 | -1.305495 |
| C | 2.486775 | -1.864027 | -2.459344 |
| H | 2.673333 | -2.569017 | -3.263133 |
| C | 1.500184 | -0.891124 | -2.573175 |
| C | 1.310580 | 0.060807 | -1.569878 |
| H | 0.542645 | 0.816750 | -1.704735 |
| C | 4.415026 | -2.831392 | -1.204973 |
| C | 0.680019 | -0.848032 | -3.827250 |
| C | 3.598154 | 2.061816 | 0.649643 |
| C | 4.576650 | 1.865647 | -0.325630 |
| H | 4.415165 | 1.141081 | -1.120900 |
| C | 5.779990 | 2.577398 | -0.317109 |
| C | 6.047469 | 3.514630 | 0.667706 |
| H | 6.983391 | 4.060785 | 0.681716 |
| C | 5.077300 | 3.731143 | 1.646781 |
| C | 3.879821 | 3.028282 | 1.630759 |
| H | 3.141881 | 3.227726 | 2.404939 |
| C | 6.778269 | 2.288791 | -1.399461 |
| C | 1.854976 | 0.667747 | 2.137019 |
| C | 0.569212 | 0.347118 | 2.606843 |
| H | -0.299296 | 0.533370 | 1.981896 |
| C | 0.372932 | -0.234771 | 3.856141 |
| C | 1.451433 | -0.587582 | 4.663356 |

| | | | |
|---|---|---|---|
| H | 1.292730 | -1.045986 | 5.634257 |
| C | 2.731610 | -0.335060 | 4.191595 |
| C | 2.925608 | 0.299491 | 2.964201 |
| H | 3.942616 | 0.520511 | 2.650340 |
| C | 3.921088 | -0.807084 | 4.973460 |

Calculated energies and coordinates of [**3a**]$^{+}$
Electronic energy ... -1715.59126734 Eh
Total Enthalpy ... -1714.93782488 Eh
Final Gibbs free energy ... -1715.04629365 Eh
CARTESIAN COORDINATES (ANGSTROEM)

| | | | |
|---|---|---|---|
| Sn | -1.358324 | 9.548407 | 3.279070 |
| O | 0.772151 | 9.531429 | 4.314947 |
| C | 0.941687 | 8.327618 | 3.967919 |
| O | 0.111723 | 7.690510 | 3.258428 |
| N | 3.144948 | 8.156018 | 5.180262 |
| N | 2.496821 | 6.352110 | 4.146399 |
| C | -0.596171 | 10.369707 | 1.363533 |
| C | -1.643965 | 11.094498 | 0.752705 |
| C | -1.472481 | 11.726212 | -0.476205 |
| H | -2.298878 | 12.276335 | -0.919561 |
| C | -0.244905 | 11.646710 | -1.122435 |
| H | -0.098347 | 12.135266 | -2.081554 |
| C | 0.798421 | 10.939781 | -0.537399 |
| H | 1.760824 | 10.877333 | -1.040932 |
| C | 0.642163 | 10.298658 | 0.698041 |
| C | -2.953767 | 11.168649 | 1.467513 |
| C | -3.183346 | 12.196137 | 2.403593 |
| C | -4.370064 | 12.188541 | 3.136145 |
| H | -4.546700 | 12.979135 | 3.862592 |
| C | -5.336708 | 11.198560 | 2.957596 |
| C | -5.101723 | 10.206369 | 2.006562 |
| H | -5.853366 | 9.436726 | 1.844230 |
| C | -3.928517 | 10.175881 | 1.251655 |
| C | -2.178678 | 13.301203 | 2.598058 |
| H | -1.159319 | 12.916659 | 2.715494 |
| H | -2.427235 | 13.907824 | 3.471974 |
| H | -2.155562 | 13.961012 | 1.722750 |
| C | -6.590963 | 11.192561 | 3.785698 |
| H | -6.921418 | 12.209723 | 4.014075 |
| H | -6.420337 | 10.681351 | 4.740901 |
| H | -7.404570 | 10.671784 | 3.274065 |
| C | -3.725276 | 9.107997 | 0.209294 |
| H | -4.477911 | 8.321535 | 0.301770 |
| H | -2.730735 | 8.652482 | 0.273414 |
| H | -3.801040 | 9.532329 | -0.798839 |
| C | 1.853088 | 9.579924 | 1.203458 |
| C | 2.819060 | 10.277820 | 1.947514 |
| C | 4.023468 | 9.645063 | 2.264124 |
| H | 4.784871 | 10.202678 | 2.808533 |
| C | 4.298820 | 8.341918 | 1.851175 |
| C | 3.318709 | 7.659902 | 1.130876 |
| H | 3.522961 | 6.648686 | 0.780466 |
| C | 2.102686 | 8.259252 | 0.794510 |
| C | 2.573199 | 11.697581 | 2.378975 |
| H | 3.436399 | 12.104682 | 2.912836 |
| H | 1.695530 | 11.761688 | 3.032339 |
| H | 2.371463 | 12.343253 | 1.517711 |
| C | 5.644653 | 7.718300 | 2.106512 |
| H | 5.578770 | 6.628370 | 2.182266 |
| H | 6.103144 | 8.104079 | 3.022455 |
| H | 6.334096 | 7.941906 | 1.283495 |
| C | 1.085034 | 7.504832 | -0.016455 |
| H | 0.141298 | 7.407075 | 0.532216 |
| H | 1.445303 | 6.504805 | -0.273457 |
| H | 0.853240 | 8.031185 | -0.948476 |
| C | 2.157444 | 7.627147 | 4.428297 |
| C | 4.132313 | 7.212240 | 5.364598 |
| C | 3.722511 | 6.071649 | 4.710867 |
| C | 3.185580 | 9.525843 | 5.708524 |
| H | 2.340098 | 9.691973 | 6.377988 |
| H | 3.129333 | 10.234879 | 4.881549 |
| H | 4.121054 | 9.653450 | 6.251560 |
| C | 5.371798 | 7.471596 | 6.139835 |
| H | 6.009390 | 6.587066 | 6.129954 |
| H | 5.153388 | 7.710381 | 7.186478 |
| H | 5.949050 | 8.299122 | 5.713259 |
| C | 4.395859 | 4.754564 | 4.582358 |
| H | 3.802647 | 3.949946 | 5.030520 |
| H | 5.359069 | 4.777218 | 5.092983 |
| H | 4.584120 | 4.497968 | 3.534150 |
| C | 1.705390 | 5.406461 | 3.348490 |
| H | 0.737932 | 5.236945 | 3.823749 |
| H | 2.257768 | 4.469961 | 3.284225 |
| H | 1.544636 | 5.819062 | 2.351641 |

Calculated energies and coordinates of [**3a**][BArF]
Electronic energy ... -5363.49812873 Eh
Total Enthalpy ... -5362.39235147 Eh
Final Gibbs free energy ... -5362.60276821 Eh
CARTESIAN COORDINATES (ANGSTROEM)

| | | | |
|---|---|---|---|
| Sn | -2.312789 | 2.433152 | 0.026458 |
| F | 6.074535 | 1.260299 | -2.779122 |
| F | 1.278923 | -6.749500 | 1.013093 |
| F | 7.629941 | -0.264075 | -2.846080 |
| F | 5.715696 | -0.610758 | -3.825564 |
| F | 2.668723 | -5.754605 | -0.336008 |
| F | 0.551081 | -5.860423 | -0.836596 |
| F | 1.351962 | -1.789122 | -5.006630 |
| F | -2.162863 | -3.874963 | 3.284502 |
| F | -0.854358 | 3.329856 | -3.053725 |
| F | 1.188502 | 3.488528 | -3.777192 |
| N | -2.401209 | -2.362755 | -1.638139 |
| F | 4.540105 | 2.942507 | 5.144741 |
| F | -1.653347 | -1.791089 | 3.667808 |
| N | -3.322766 | -1.315617 | -3.307106 |
| F | 0.745179 | 3.909008 | -1.688429 |
| C | 1.557976 | -1.966675 | 1.044173 |
| C | 2.291327 | 0.553958 | 1.615043 |
| C | 3.814869 | -0.955340 | 0.011923 |
| C | 1.468958 | -0.111513 | -0.884156 |
| C | 3.444615 | 0.897495 | 2.328536 |
| H | 4.389642 | 0.421198 | 2.080823 |
| C | 5.743836 | -0.677453 | -1.460424 |
| C | 1.838607 | -3.225936 | 0.487336 |
| H | 2.608801 | -3.312013 | -0.274995 |
| C | 1.176432 | -4.381677 | 0.897573 |
| C | 1.235709 | 1.239934 | -1.185474 |
| H | 1.518048 | 2.002545 | -0.466049 |
| C | -2.764340 | -1.144455 | -2.088897 |
| C | 4.625031 | -1.781260 | 0.804498 |
| H | 4.211248 | -2.228700 | 1.706954 |
| C | 0.588356 | -1.946927 | 2.056876 |
| H | 0.337502 | -1.009094 | 2.541729 |
| F | -0.771079 | -1.328823 | -4.943365 |
| C | 1.112114 | -1.032918 | -1.878165 |
| H | 1.292940 | -2.091689 | -1.713262 |
| C | 4.416089 | -0.409875 | -1.127218 |
| H | 3.840685 | 0.243612 | -1.779099 |
| C | 1.115638 | 1.228629 | 1.986723 |
| H | 0.190729 | 1.015525 | 1.453476 |
| F | 6.175563 | -4.180543 | 1.478193 |
| C | 5.951311 | -2.050254 | 0.475397 |
| C | 0.205202 | -4.334412 | 1.891429 |
| H | -0.312043 | -5.233386 | 2.210918 |
| C | 3.427600 | 1.836903 | 3.360062 |
| C | 0.669207 | 1.638514 | -2.395924 |
| F | 5.345151 | 1.028463 | 4.484433 |
| C | 6.297893 | -0.075888 | -2.719305 |
| C | -1.725590 | -2.649072 | -0.367566 |
| H | -1.396037 | -3.686937 | -0.382972 |
| H | -2.414247 | -2.473356 | 0.461652 |
| H | -0.861039 | -1.995065 | -0.256311 |
| C | 1.093776 | 2.163667 | 3.018844 |
| C | -0.081389 | -3.099735 | 2.463647 |
| C | 1.437554 | -5.683184 | 0.199481 |
| F | 0.051235 | -2.890135 | -3.658794 |
| C | 6.528904 | -1.503551 | -0.665574 |
| H | 7.559174 | -1.714393 | -0.924846 |
| C | -4.488299 | 2.694916 | 0.464460 |
| C | 0.446610 | 3.089693 | -2.714923 |
| C | 0.325430 | 0.705573 | -3.368581 |

| | | | |
|---|---|---|---|
| H | -0.099665 | 1.021621 | -4.316168 |
| F | -0.636704 | -3.378750 | 4.754481 |
| C | 2.253756 | 2.481031 | 3.721218 |
| H | 2.238507 | 3.205361 | 4.527216 |
| B | 2.280483 | -0.606202 | 0.461740 |
| C | 0.558291 | -0.637626 | -3.095403 |
| C | -5.533532 | 1.792519 | 0.717984 |
| F | 5.586064 | 2.794627 | 3.240015 |
| C | -2.739408 | -3.318446 | -2.570083 |
| F | 6.801072 | -2.456326 | 2.645130 |
| C | -4.768859 | 4.075064 | 0.493697 |
| C | -3.645511 | 5.002200 | 0.162470 |
| C | -2.890004 | 5.589731 | 1.197092 |
| C | -3.274890 | 5.199326 | -1.183843 |
| F | 8.012599 | -3.128108 | 0.963599 |
| C | -6.807976 | 2.280638 | 1.030492 |
| H | -7.606707 | 1.571102 | 1.236443 |
| C | -3.321744 | -2.657179 | -3.623880 |
| C | -1.759512 | 6.332469 | 0.867918 |
| H | -1.160850 | 6.762473 | 1.668422 |
| C | -4.111979 | 4.651900 | -2.312451 |
| H | -4.369148 | 3.598138 | -2.165821 |
| H | -3.587247 | 4.752470 | -3.265685 |
| H | -5.061152 | 5.196960 | -2.382382 |
| C | -5.362614 | 0.312436 | 0.629838 |
| C | 6.740716 | -2.949169 | 1.382946 |
| C | -7.061165 | 3.646804 | 1.079856 |
| H | -8.056534 | 4.005322 | 1.327467 |
| C | -5.792423 | -0.356251 | -0.528287 |
| C | -1.356751 | 6.512841 | -0.456514 |
| C | -3.810143 | -0.285845 | -4.227557 |
| H | -3.191770 | -0.298405 | -5.129853 |
| H | -3.739329 | 0.685321 | -3.744919 |
| H | -4.848566 | -0.502385 | -4.491694 |
| C | 4.718653 | 2.151462 | 4.061770 |
| C | -0.171112 | 2.891892 | 3.348263 |
| C | -2.132186 | 5.952045 | -1.466989 |
| H | -1.835905 | 6.088507 | -2.504449 |
| C | -5.669422 | -1.745134 | -0.600060 |
| H | -6.006293 | -2.261535 | -1.498764 |
| C | -6.378788 | 0.408266 | -1.685758 |
| H | -5.714659 | 1.221217 | -2.000668 |
| H | -7.332819 | 0.873831 | -1.415122 |
| H | -6.560475 | -0.254394 | -2.537340 |
| C | -1.123399 | -3.029236 | 3.541766 |
| C | -6.042487 | 4.550638 | 0.799037 |
| H | -6.231802 | 5.621594 | 0.810796 |
| C | 0.305953 | -1.655703 | -4.165230 |
| C | -4.823493 | -0.416601 | 1.700797 |
| C | -3.276753 | 5.385261 | 2.636655 |
| H | -4.247411 | 5.844546 | 2.854812 |
| H | -2.530321 | 5.817841 | 3.306379 |
| H | -3.376402 | 4.320048 | 2.875995 |
| C | -4.318666 | 0.275770 | 2.935614 |
| H | -4.289538 | -0.409939 | 3.786094 |
| H | -4.933315 | 1.142113 | 3.195499 |
| H | -3.294975 | 0.638642 | 2.782132 |
| C | -3.826363 | -3.162551 | -4.923433 |
| H | -3.741681 | -4.249032 | -4.962091 |
| H | -3.245388 | -2.751552 | -5.757230 |
| H | -4.878334 | -2.900333 | -5.081792 |
| C | -5.154796 | -2.487642 | 0.461968 |
| C | -2.426038 | -4.759080 | -2.406047 |
| H | -2.840290 | -5.326203 | -3.240830 |
| H | -2.847880 | -5.163943 | -1.480635 |
| H | -1.344071 | -4.928714 | -2.388061 |
| C | -4.735663 | -1.805687 | 1.602857 |
| H | -4.328378 | -2.367287 | 2.441939 |
| C | -0.095321 | 7.265841 | -0.773184 |
| H | -0.035902 | 7.518629 | -1.834880 |
| H | 0.784066 | 6.659763 | -0.525555 |
| H | -0.028936 | 8.190978 | -0.191783 |
| C | -5.084231 | -3.989749 | 0.396854 |
| H | -5.917639 | -4.443100 | 0.946795 |
| H | -4.161601 | -4.368823 | 0.850839 |
| H | -5.142044 | -4.347049 | -0.636413 |
| F | -0.314324 | 4.025199 | 2.607125 |
| F | -0.239687 | 3.266920 | 4.642201 |
| F | -1.283919 | 2.138158 | 3.099001 |
| O | -2.896221 | 1.219068 | -1.899861 |
| C | -2.642625 | 0.117600 | -1.322580 |
| O | -2.295717 | 0.047782 | -0.114246 |

Calculated energies and coordinates of [**4**]$^{+}$
Electronic energy ... -2287.56604510 Eh
Total Enthalpy ... -2286.70013232 Eh
Final Gibbs free energy ... -2286.83832856 Eh
CARTESIAN COORDINATES (ANGSTROEM)

| | | | |
|---|---|---|---|
| Sn | 3.390065 | 11.977764 | 8.063714 |
| O | 1.687531 | 13.180963 | 8.932262 |
| O | 3.036134 | 12.827292 | 10.687914 |
| O | 2.367100 | 12.437654 | 6.064367 |
| O | 0.381805 | 11.460423 | 6.567078 |
| C | 4.669595 | 13.767370 | 7.589197 |
| C | 4.521501 | 15.138350 | 7.867123 |
| C | 5.420372 | 16.066954 | 7.322798 |
| H | 5.286239 | 17.122409 | 7.551976 |
| C | 6.475505 | 15.660444 | 6.516820 |
| H | 7.163299 | 16.394599 | 6.106449 |
| C | 6.656454 | 14.307369 | 6.261881 |
| H | 7.494134 | 13.964630 | 5.658513 |
| C | 5.766921 | 13.373189 | 6.795319 |
| C | 3.479049 | 15.720228 | 8.771915 |
| C | 3.799358 | 15.958436 | 10.119061 |
| C | 2.879369 | 16.624735 | 10.932807 |
| H | 3.145978 | 16.833078 | 11.968406 |
| C | 1.651365 | 17.066716 | 10.444230 |
| C | 1.345791 | 16.808116 | 9.108775 |
| H | 0.399150 | 17.161406 | 8.700772 |
| C | 2.241127 | 16.146457 | 8.267314 |
| C | 5.116660 | 15.503730 | 10.684400 |
| H | 5.207671 | 14.413761 | 10.617267 |
| H | 5.220519 | 15.800971 | 11.731958 |
| H | 5.957095 | 15.926890 | 10.124566 |
| C | 0.712094 | 17.861061 | 11.311891 |
| H | -0.335375 | 17.657653 | 11.065994 |
| H | 0.873056 | 18.937194 | 11.174209 |
| H | 0.865992 | 17.644517 | 12.373648 |
| C | 1.867332 | 15.871914 | 6.838569 |
| H | 0.953024 | 16.404715 | 6.559586 |
| H | 1.707630 | 14.795907 | 6.699606 |
| H | 2.669987 | 16.170567 | 6.155402 |
| C | 6.005755 | 11.918377 | 6.538455 |
| C | 5.669028 | 11.339597 | 5.303240 |
| C | 5.868076 | 9.971349 | 5.113275 |
| H | 5.598013 | 9.526492 | 4.156956 |
| C | 6.417979 | 9.166834 | 6.107392 |
| C | 6.782670 | 9.764419 | 7.314142 |
| H | 7.234233 | 9.154525 | 8.094353 |
| C | 6.585978 | 11.122644 | 7.548695 |
| C | 5.126885 | 12.177602 | 4.176450 |
| H | 5.944240 | 12.584267 | 3.567448 |
| H | 4.552950 | 13.022324 | 4.561005 |
| H | 4.488274 | 11.578281 | 3.518438 |
| C | 6.607470 | 7.689769 | 5.898361 |
| H | 6.553161 | 7.424886 | 4.838995 |
| H | 5.833003 | 7.120012 | 6.425872 |
| H | 7.574959 | 7.355841 | 6.285875 |
| C | 6.986266 | 11.720517 | 8.871545 |
| H | 7.480884 | 12.687748 | 8.741084 |
| H | 7.658464 | 11.048201 | 9.410548 |
| H | 6.113301 | 11.898351 | 9.515051 |
| C | 1.980334 | 13.224330 | 10.172318 |
| C | 1.224620 | 11.928924 | 5.804815 |
| N | -0.336533 | 14.121186 | 10.687547 |
| H | -0.484774 | 14.678290 | 8.666282 |
| H | -1.999119 | 14.100927 | 9.432190 |
| H | -0.707012 | 12.948963 | 8.973513 |
| H | -1.763198 | 10.178856 | 1.804325 |
| N | 1.062407 | 14.105082 | 12.352313 |
| H | 3.135185 | 14.312393 | 12.602308 |
| H | 2.446351 | 12.853297 | 13.333330 |
| H | 2.154998 | 14.446934 | 14.093681 |
| H | 2.396401 | 14.038746 | 4.572468 |

| | | | |
|---|---|---|---|
| C | 0.921016 | 13.796409 | 11.048599 |
| H | -1.433032 | 9.992702 | 4.749620 |
| C | -0.998040 | 14.663477 | 11.773139 |
| H | -2.532504 | 15.916538 | 10.948933 |
| H | -2.690226 | 15.572935 | 12.672034 |
| H | -3.101951 | 14.326942 | 11.493454 |
| H | 0.543397 | 14.208997 | 1.025549 |
| C | -0.114758 | 14.652516 | 12.824659 |
| H | 0.395442 | 15.935863 | 14.468324 |
| H | -0.117471 | 14.305576 | 14.943025 |
| H | -1.305684 | 15.479352 | 14.374360 |
| C | -0.922930 | 13.948469 | 9.351438 |
| C | -0.742394 | 10.356818 | 1.450103 |
| H | -0.807351 | 10.827628 | 0.468170 |
| H | -0.255064 | 9.383885 | 1.318331 |
| C | 0.900644 | 13.179459 | 0.912992 |
| H | 1.958209 | 13.221379 | 0.628734 |
| H | 0.352825 | 12.724967 | 0.086516 |
| C | 1.994072 | 14.024045 | 3.561809 |
| H | 2.816568 | 14.066013 | 2.843342 |
| H | 1.327260 | 14.877698 | 3.404256 |
| C | -2.402659 | 15.141637 | 11.712395 |
| H | -0.461888 | 8.949875 | 3.666050 |
| C | -0.288618 | 15.114988 | 14.225177 |
| H | 0.189508 | 9.485668 | 5.242257 |
| C | 2.280201 | 13.916938 | 13.149585 |
| N | 0.148837 | 10.945456 | 3.733036 |
| N | 1.246855 | 12.778519 | 3.370664 |
| C | 0.898104 | 11.894602 | 4.325971 |
| C | 0.014598 | 11.229793 | 2.382391 |
| C | 0.701273 | 12.392335 | 2.155398 |
| C | -0.425821 | 9.765993 | 4.389533 |

Calculated energies and coordinates of [**4**][BArF]
Electronic energy ... -5935.48159210 Eh
Total Enthalpy ... -5934.16336500 Eh
Final Gibbs free energy ... -5934.39940847 Eh
CARTESIAN COORDINATES (ANGSTROEM)

| | | | |
|---|---|---|---|
| Sn | -4.367022 | 1.282271 | 0.664131 |
| F | 3.098727 | -2.519958 | 4.977083 |
| F | 3.781768 | -3.778354 | 3.334340 |
| F | 5.098295 | -2.255087 | 4.160881 |
| F | 0.970859 | 2.159259 | -5.352165 |
| F | 1.848459 | 0.273678 | -5.998057 |
| F | 2.918803 | 2.142381 | -6.324397 |
| F | -1.494899 | -5.792929 | -0.745920 |
| F | 0.288733 | -6.104515 | -1.952200 |
| F | 0.476496 | -5.789486 | 0.188495 |
| F | -1.939046 | 0.146122 | -2.830841 |
| F | -2.708419 | -1.749712 | -3.567523 |
| F | -3.248159 | -1.071486 | -1.577269 |
| F | 3.859541 | -5.844031 | -3.734189 |
| F | 5.848273 | -5.470630 | -4.541463 |
| F | 4.153153 | -4.277432 | -5.212334 |
| O | -3.078969 | -0.270524 | 1.580766 |
| O | -4.789394 | -1.619662 | 1.043502 |
| O | -2.578556 | 2.648982 | 1.069414 |
| O | -1.190994 | 1.367117 | -0.182506 |
| C | -5.255619 | 1.983057 | 2.618928 |
| C | -5.194053 | 1.433388 | 3.912151 |
| C | -5.814449 | 2.083990 | 4.987337 |
| H | -5.750973 | 1.637745 | 5.978010 |
| C | -6.507395 | 3.273372 | 4.802208 |
| H | -6.983542 | 3.765469 | 5.646110 |
| C | -6.599885 | 3.818076 | 3.527564 |
| H | -7.156183 | 4.737700 | 3.357679 |
| C | -5.987482 | 3.176592 | 2.449269 |
| C | -4.513808 | 0.141157 | 4.235750 |
| C | -3.168453 | 0.130138 | 4.635084 |
| C | -2.579278 | -1.071834 | 5.026429 |
| H | -1.538896 | -1.071828 | 5.350239 |
| C | -3.297950 | -2.267289 | 5.034792 |
| C | -4.626874 | -2.239729 | 4.617041 |
| H | -5.205884 | -3.162358 | 4.617072 |
| C | -5.248651 | -1.053633 | 4.219757 |
| C | -2.361504 | 1.396966 | 4.600008 |
| H | -1.357272 | 1.241075 | 5.004159 |
| H | -2.849559 | 2.196636 | 5.168154 |
| H | -2.269781 | 1.748823 | 3.565316 |
| C | -2.671425 | -3.540997 | 5.536988 |
| H | -3.174903 | -4.422320 | 5.126518 |
| H | -2.744180 | -3.605825 | 6.629698 |
| H | -1.609255 | -3.599394 | 5.277910 |
| C | -6.683105 | -1.066756 | 3.768017 |
| H | -6.755351 | -0.774123 | 2.714644 |
| H | -7.288965 | -0.357379 | 4.341697 |
| H | -7.118549 | -2.064008 | 3.875875 |
| C | -6.137516 | 3.765219 | 1.081349 |
| C | -7.113201 | 3.248969 | 0.205542 |
| C | -7.227161 | 3.782190 | -1.076234 |
| H | -7.979494 | 3.377925 | -1.751032 |
| C | -6.397733 | 4.812683 | -1.517756 |
| C | -5.455029 | 5.326890 | -0.631239 |
| H | -4.810494 | 6.143364 | -0.953190 |
| C | -5.319055 | 4.827829 | 0.664899 |
| C | -8.011867 | 2.120641 | 0.634149 |
| H | -8.396969 | 2.277302 | 1.646431 |
| H | -7.469977 | 1.164822 | 0.650395 |
| H | -8.853310 | 2.010630 | -0.054769 |
| C | -6.507305 | 5.335539 | -2.924112 |
| H | -7.549867 | 5.370762 | -3.254909 |
| H | -5.965805 | 4.685916 | -3.622669 |
| H | -6.085117 | 6.340783 | -3.010838 |
| C | -4.321147 | 5.446474 | 1.605948 |
| H | -4.800733 | 6.203293 | 2.239734 |
| H | -3.516888 | 5.936438 | 1.047038 |
| H | -3.887704 | 4.694008 | 2.267986 |
| N | -1.395887 | -2.477431 | 1.979643 |
| N | -3.065944 | -3.857382 | 1.840927 |
| C | -2.715045 | -2.560067 | 1.711923 |
| C | -0.908746 | -3.728244 | 2.302183 |
| C | -1.962075 | -4.600969 | 2.208444 |
| C | -0.561543 | -1.270687 | 1.932438 |
| H | 0.480526 | -1.587185 | 1.922704 |
| H | -0.766556 | -0.648770 | 2.807504 |
| H | -0.782164 | -0.694046 | 1.034400 |
| C | 0.499758 | -3.968415 | 2.699017 |
| H | 1.195346 | -3.728354 | 1.888194 |
| H | 0.642725 | -5.018147 | 2.957924 |
| H | 0.775701 | -3.362462 | 3.569510 |
| C | -2.030397 | -6.062312 | 2.452096 |
| H | -2.482709 | -6.586977 | 1.604759 |
| H | -2.615301 | -6.295387 | 3.350270 |
| H | -1.025647 | -6.463731 | 2.588911 |
| C | -4.397182 | -4.436260 | 1.654052 |
| H | -4.413327 | -5.019620 | 0.727513 |
| H | -5.122881 | -3.627879 | 1.588443 |
| H | -4.618321 | -5.091722 | 2.500529 |
| N | -0.328629 | 4.562766 | 1.125436 |
| N | 0.193417 | 3.800034 | -0.836155 |
| C | -0.585108 | 3.579143 | 0.241346 |
| C | 0.643208 | 5.406257 | 0.612604 |
| C | 0.962634 | 4.933169 | -0.631457 |
| C | -0.868395 | 4.687552 | 2.479592 |
| H | -1.611133 | 3.908572 | 2.636240 |
| H | -1.340763 | 5.667282 | 2.592172 |
| H | -0.054980 | 4.584198 | 3.203712 |
| C | 1.185706 | 6.551139 | 1.384138 |
| H | 0.401942 | 7.267215 | 1.655574 |
| H | 1.932952 | 7.080222 | 0.791056 |
| H | 1.668590 | 6.212696 | 2.308223 |
| C | 1.893394 | 5.458634 | -1.660693 |
| H | 2.582136 | 6.179009 | -1.216580 |
| H | 1.352682 | 5.960116 | -2.472802 |
| H | 2.491514 | 4.656357 | -2.099943 |
| C | 0.239213 | 3.000449 | -2.067067 |
| H | -0.685191 | 2.437252 | -2.174226 |
| H | 1.079383 | 2.299957 | -2.020500 |
| H | 0.374716 | 3.672225 | -2.916554 |
| C | -3.627334 | -1.412679 | 1.412693 |
| C | -1.523015 | 2.406131 | 0.386886 |
| C | 2.833889 | -0.865066 | 0.523848 |
| C | 2.300614 | 0.326486 | 1.028942 |

| | | | |
|---|---|---|---|
| H | 1.876098 | 1.054436 | 0.340413 |
| C | 2.294311 | 0.610368 | 2.393849 |
| C | 2.779677 | -0.305295 | 3.319620 |
| H | 2.769816 | -0.088704 | 4.382022 |
| C | 3.275562 | -1.514763 | 2.839872 |
| C | 3.312879 | -1.781283 | 1.472869 |
| H | 3.727247 | -2.727852 | 1.134419 |
| C | 1.694860 | 1.900358 | 2.852398 |
| F | 1.994236 | 2.205432 | 4.132549 |
| F | 2.092976 | 2.954825 | 2.089383 |
| F | 0.330916 | 1.875924 | 2.769417 |
| C | 3.815136 | -2.516385 | 3.820849 |
| C | 3.002647 | 0.125976 | -1.982692 |
| C | 2.508488 | 0.234755 | -3.292877 |
| H | 1.888344 | -0.560480 | -3.698395 |
| C | 2.792647 | 1.335304 | -4.097145 |
| C | 3.607322 | 2.369315 | -3.639948 |
| H | 3.836706 | 3.218911 | -4.273451 |
| C | 4.129063 | 2.266262 | -2.356536 |
| C | 3.830674 | 1.168887 | -1.548687 |
| H | 4.266539 | 1.127672 | -0.552823 |
| C | 2.146089 | 1.464603 | -5.445603 |
| C | 5.038793 | 3.325430 | -1.806274 |
| F | 4.592041 | 3.786148 | -0.602430 |
| F | 6.291273 | 2.866292 | -1.598977 |
| F | 5.136351 | 4.404116 | -2.621168 |
| C | 1.325256 | -1.928323 | -1.296903 |
| C | 1.140662 | -3.285740 | -1.001124 |
| H | 1.991236 | -3.880447 | -0.672251 |
| C | -0.097953 | -3.912716 | -1.151234 |
| C | -1.199185 | -3.206384 | -1.616918 |
| H | -2.153960 | -3.696620 | -1.777197 |
| C | -1.038830 | -1.853607 | -1.905288 |
| C | 0.189005 | -1.225005 | -1.725873 |
| H | 0.260507 | -0.163325 | -1.936214 |
| C | -0.206982 | -5.389875 | -0.917869 |
| C | -2.223901 | -1.119691 | -2.464623 |
| C | 4.011227 | -2.189520 | -1.528900 |
| C | 3.878392 | -3.115855 | -2.569852 |
| H | 2.909405 | -3.256133 | -3.044885 |
| C | 4.956816 | -3.873437 | -3.023582 |
| C | 6.219210 | -3.730665 | -2.460101 |
| H | 7.057103 | -4.321225 | -2.809715 |
| C | 6.375139 | -2.799005 | -1.439321 |
| C | 5.295292 | -2.043624 | -0.988777 |
| H | 5.465490 | -1.318512 | -0.194831 |
| C | 4.714860 | -4.863716 | -4.125940 |
| C | 7.708539 | -2.601771 | -0.778263 |
| F | 7.692383 | -3.029817 | 0.511880 |
| F | 8.704528 | -3.267994 | -1.402154 |
| F | 8.062522 | -1.293168 | -0.742003 |
| B | 2.781131 | -1.214606 | -1.071541 |

Calculated energies and coordinates of **TS(2_3)**

Electronic energy ... -2098.92836904 Eh
Total Enthalpy ... -2098.08225296 Eh
Final Gibbs free energy ... -2098.21135727 Eh
CARTESIAN COORDINATES (ANGSTROEM)

| | | | |
|---|---|---|---|
| Sn | -0.956589 | -0.755452 | -0.022301 |
| O | 0.485229 | -0.105657 | 3.701418 |
| C | -0.095057 | -0.985842 | 3.119054 |
| O | -1.099869 | -1.657148 | 3.128254 |
| N | -0.700064 | 2.299405 | 1.366265 |
| N | -2.365575 | 1.188691 | 2.136757 |
| N | 2.298955 | -1.139440 | 1.729774 |
| N | 1.130081 | -2.938469 | 1.559218 |
| C | -0.248032 | 0.379242 | -1.833361 |
| C | -1.301484 | 1.097408 | -2.440084 |
| C | -1.120305 | 1.748631 | -3.660182 |
| H | -1.950874 | 2.290987 | -4.106211 |
| C | 0.105974 | 1.679022 | -4.309801 |
| H | 0.248399 | 2.174878 | -5.265527 |
| C | 1.137543 | 0.940006 | -3.747819 |
| H | 2.083394 | 0.843030 | -4.275349 |
| C | 0.973969 | 0.280138 | -2.520937 |
| C | -2.647032 | 1.112685 | -1.787445 |
| C | -3.142369 | 2.279593 | -1.172561 |
| C | -4.369696 | 2.237988 | -0.514259 |
| H | -4.739117 | 3.143522 | -0.034832 |
| C | -5.139170 | 1.075883 | -0.460089 |
| C | -4.659376 | -0.059334 | -1.105701 |
| H | -5.262300 | -0.965778 | -1.111329 |
| C | -3.439414 | -0.059308 | -1.789819 |
| C | -2.400116 | 3.587471 | -1.236346 |
| H | -1.344479 | 3.447312 | -1.472250 |
| H | -2.495204 | 4.135196 | -0.293051 |
| H | -2.820912 | 4.224599 | -2.023847 |
| C | -6.474371 | 1.063444 | 0.231978 |
| H | -6.530819 | 1.827864 | 1.012749 |
| H | -6.686020 | 0.088125 | 0.680701 |
| H | -7.279729 | 1.271855 | -0.482426 |
| C | -3.092646 | -1.261877 | -2.634344 |
| H | -3.400569 | -2.191242 | -2.146463 |
| H | -2.029901 | -1.321810 | -2.874928 |
| H | -3.632942 | -1.196970 | -3.587186 |
| C | 2.124024 | -0.557142 | -2.065493 |
| C | 3.349149 | 0.046053 | -1.726627 |
| C | 4.466814 | -0.755599 | -1.486488 |
| H | 5.417312 | -0.276015 | -1.255059 |
| C | 4.409729 | -2.143519 | -1.583469 |
| C | 3.184734 | -2.726982 | -1.900184 |
| H | 3.125765 | -3.808904 | -2.008402 |
| C | 2.046602 | -1.962274 | -2.156318 |
| C | 3.509515 | 1.545022 | -1.677278 |
| H | 4.314408 | 1.829511 | -0.992240 |
| H | 2.588635 | 2.049282 | -1.373885 |
| H | 3.771912 | 1.945873 | -2.663387 |
| C | 5.644445 | -2.987059 | -1.422002 |
| H | 5.415914 | -3.953730 | -0.961687 |
| H | 6.405052 | -2.483673 | -0.818448 |
| H | 6.093677 | -3.197396 | -2.399978 |
| C | 0.795087 | -2.655384 | -2.627704 |
| H | 0.017545 | -2.691098 | -1.852424 |
| H | 1.012197 | -3.686201 | -2.919588 |
| H | 0.357258 | -2.137468 | -3.486843 |
| C | -1.339028 | 1.108926 | 1.260214 |
| C | -1.315192 | 3.118527 | 2.303858 |
| C | -2.376411 | 2.411654 | 2.791133 |
| C | 0.479447 | 2.701903 | 0.613683 |
| H | 1.326330 | 2.869195 | 1.286860 |
| H | 0.281812 | 3.628617 | 0.067724 |
| H | 0.717153 | 1.916312 | -0.105518 |
| C | -0.813508 | 4.477154 | 2.629094 |
| H | 0.209724 | 4.446414 | 3.020618 |
| H | -1.444197 | 4.938673 | 3.390516 |
| H | -0.816214 | 5.134224 | 1.751368 |
| C | -3.397957 | 2.761732 | 3.808907 |
| H | -3.210217 | 3.761121 | 4.204426 |
| H | -3.382542 | 2.060096 | 4.650204 |
| H | -4.408775 | 2.753992 | 3.384753 |
| C | -3.364331 | 0.153726 | 2.389982 |
| H | -3.276989 | -0.205057 | 3.418613 |
| H | -3.192336 | -0.683530 | 1.712923 |
| H | -4.363294 | 0.558983 | 2.209359 |
| C | 1.003552 | -1.575911 | 1.690344 |
| C | 3.194713 | -2.175036 | 1.652231 |
| C | 2.448192 | -3.323302 | 1.524306 |
| C | 2.693162 | 0.251813 | 1.891501 |
| H | 3.340389 | 0.359038 | 2.766872 |
| H | 1.794368 | 0.838501 | 2.056425 |
| H | 3.219472 | 0.589156 | 0.992417 |
| C | 4.657876 | -1.982798 | 1.790732 |
| H | 5.188009 | -2.911003 | 1.573248 |
| H | 4.916064 | -1.679560 | 2.812761 |
| H | 5.024429 | -1.215107 | 1.103027 |
| C | 2.878003 | -4.734482 | 1.370655 |
| H | 2.586815 | -5.346742 | 2.232133 |
| H | 3.963973 | -4.787925 | 1.278007 |
| H | 2.440820 | -5.188336 | 0.474119 |
| C | 0.018181 | -3.875959 | 1.428739 |
| H | -0.884496 | -3.405116 | 1.811781 |
| H | 0.234645 | -4.777331 | 2.007135 |

| | | | |
|---|---|---|---|
| H | -0.120931 | -4.144507 | 0.374632 |

Calculated energies and coordinates of **TS(3_4)**

Electronic energy ... -2287.52986583 Eh
Total Enthalpy ... -2286.66645624 Eh
Final Gibbs free energy ... -2286.80125225 Eh

CARTESIAN COORDINATES (ANGSTROEM)

| | | | |
|---|---|---|---|
| Sn | 0.658985 | -1.257593 | 0.201077 |
| O | -1.075959 | 0.156470 | 0.739824 |
| O | -0.005579 | -0.533203 | 2.563227 |
| O | -3.396481 | -1.245895 | -0.922361 |
| O | -2.156917 | -2.576732 | 0.481773 |
| C | 1.991910 | 0.479271 | -0.337772 |
| C | 2.069562 | 1.794436 | 0.167137 |
| C | 3.114713 | 2.639315 | -0.225070 |
| H | 3.149405 | 3.649788 | 0.176784 |
| C | 4.103161 | 2.205230 | -1.098705 |
| H | 4.910448 | 2.872886 | -1.386020 |
| C | 4.054414 | 0.909063 | -1.591175 |
| H | 4.825346 | 0.544372 | -2.266480 |
| C | 3.014635 | 0.056398 | -1.213827 |
| C | 1.102834 | 2.390950 | 1.141197 |
| C | 1.356290 | 2.303465 | 2.520361 |
| C | 0.512033 | 2.971335 | 3.410165 |
| H | 0.727997 | 2.926740 | 4.476823 |
| C | -0.572817 | 3.727477 | 2.968041 |
| C | -0.805334 | 3.804189 | 1.595710 |
| H | -1.630808 | 4.412821 | 1.227297 |
| C | 0.025026 | 3.160015 | 0.677111 |
| C | 2.530253 | 1.518870 | 3.037292 |
| H | 2.428729 | 0.455486 | 2.794157 |
| H | 2.621659 | 1.615426 | 4.122559 |
| H | 3.465284 | 1.861847 | 2.582198 |
| C | -1.425535 | 4.498354 | 3.939935 |
| H | -1.049915 | 5.522081 | 4.056970 |
| H | -1.420954 | 4.037911 | 4.932900 |
| H | -2.462516 | 4.572412 | 3.596799 |
| C | -0.208583 | 3.333711 | -0.798384 |
| H | 0.428859 | 4.132295 | -1.198014 |
| H | -1.247112 | 3.607961 | -1.010526 |
| H | 0.047087 | 2.424771 | -1.348013 |
| C | 3.018101 | -1.331185 | -1.774371 |
| C | 2.538444 | -1.554849 | -3.075739 |
| C | 2.590086 | -2.843123 | -3.609848 |
| H | 2.242391 | -3.005086 | -4.628567 |
| C | 3.112706 | -3.914735 | -2.888230 |
| C | 3.606685 | -3.669901 | -1.605917 |
| H | 4.046531 | -4.488986 | -1.040148 |
| C | 3.582279 | -2.395501 | -1.041499 |
| C | 2.038506 | -0.411274 | -3.915397 |
| H | 2.869379 | 0.224439 | -4.242919 |
| H | 1.359391 | 0.235823 | -3.349983 |
| H | 1.527389 | -0.781763 | -4.807306 |
| C | 3.155291 | -5.300284 | -3.473392 |
| H | 2.976355 | -5.285178 | -4.552111 |
| H | 2.398541 | -5.949839 | -3.014639 |
| H | 4.126391 | -5.773655 | -3.298540 |
| C | 4.183522 | -2.161305 | 0.317677 |
| H | 4.886330 | -1.322050 | 0.297554 |
| H | 4.708288 | -3.051860 | 0.670946 |
| H | 3.420677 | -1.908214 | 1.067188 |
| C | -0.950960 | 0.059032 | 2.002268 |
| C | -2.494030 | -1.880838 | -0.439578 |
| N | -3.115483 | 1.308242 | 2.416067 |
| H | -3.612952 | 0.552558 | 0.508412 |
| H | -2.743991 | 2.109035 | 0.514340 |
| H | -4.461131 | 2.043728 | 1.006817 |
| H | -1.440123 | -4.803181 | -4.505886 |
| N | -2.015743 | 0.694134 | 4.191193 |
| H | -0.013510 | 0.514034 | 4.798400 |
| H | -0.970686 | -0.970739 | 4.950768 |
| H | -1.237373 | 0.375475 | 6.097190 |
| H | -1.612970 | 0.708985 | -1.597239 |
| C | -2.005722 | 0.671870 | 2.842083 |
| H | -0.860097 | -5.071491 | -2.044404 |
| C | -3.832368 | 1.754568 | 3.506914 |
| H | -4.975235 | 3.437091 | 2.824550 |
| H | -5.472563 | 2.775709 | 4.381905 |
| H | -5.885240 | 1.915172 | 2.897979 |
| H | -2.414244 | -0.627394 | -5.414676 |
| C | -3.138617 | 1.365048 | 4.628560 |
| H | -2.677879 | 2.159602 | 6.569516 |
| H | -3.567237 | 0.624039 | 6.597056 |
| H | -4.392912 | 2.120786 | 6.162769 |
| C | -3.509350 | 1.513833 | 1.016223 |
| C | -0.615317 | -4.083935 | -4.578518 |
| H | -0.545496 | -3.764513 | -5.620038 |
| H | 0.311731 | -4.607630 | -4.322974 |
| C | -1.369439 | -0.940343 | -5.301387 |
| H | -0.742246 | -0.048861 | -5.410807 |
| H | -1.134339 | -1.619373 | -6.122932 |
| C | -1.685906 | 0.434543 | -2.649037 |
| H | -1.042019 | 1.089987 | -3.244095 |
| H | -2.724788 | 0.541063 | -2.977380 |
| C | -5.106568 | 2.508138 | 3.390126 |
| H | 0.685941 | -4.289586 | -1.587278 |
| C | -3.454303 | 1.574477 | 6.064344 |
| H | -0.760046 | -4.098092 | -0.553279 |
| C | -0.991424 | 0.114190 | 5.068510 |
| N | -0.791196 | -2.994363 | -2.320017 |
| N | -1.256905 | -0.948779 | -2.791846 |
| C | -1.055232 | -1.785757 | -1.733881 |
| C | -0.836487 | -2.913498 | -3.693436 |
| C | -1.145842 | -1.608037 | -3.995175 |
| C | -0.407104 | -4.192328 | -1.579876 |

Calculated energies and coordinates of **TS1**

Electronic energy ... -3041.42260597 Eh
Total Enthalpy ... -3040.34613708 Eh
Final Gibbs free energy ... -3040.50636656 Eh

CARTESIAN COORDINATES (ANGSTROEM)

| | | | |
|---|---|---|---|
| Sn | 1.670829 | -0.103240 | 0.307410 |
| O | -0.123629 | 0.881114 | 1.353828 |
| O | 1.268136 | -0.003426 | 2.850411 |
| C | 2.909428 | 1.756179 | 0.119703 |
| C | 3.119995 | 2.827005 | 1.005703 |
| C | 4.056846 | 3.820736 | 0.695533 |
| H | 4.202569 | 4.641514 | 1.394857 |
| C | 4.797960 | 3.766611 | -0.477790 |
| H | 5.523017 | 4.544283 | -0.701018 |
| C | 4.607596 | 2.711513 | -1.360432 |
| H | 5.181660 | 2.651068 | -2.282411 |
| C | 3.676302 | 1.716130 | -1.064059 |
| C | 2.395806 | 2.985650 | 2.303153 |
| C | 2.967126 | 2.501650 | 3.489914 |
| C | 2.330200 | 2.759028 | 4.704752 |
| H | 2.796992 | 2.414217 | 5.626250 |
| C | 1.134065 | 3.471579 | 4.770051 |
| C | 0.574191 | 3.932463 | 3.577682 |
| H | -0.350662 | 4.508027 | 3.609246 |
| C | 1.197036 | 3.714378 | 2.346849 |
| C | 4.240332 | 1.703641 | 3.460373 |
| H | 4.080874 | 0.743831 | 2.952168 |
| H | 4.600285 | 1.496450 | 4.471422 |
| H | 5.027967 | 2.225786 | 2.909994 |
| C | 0.502098 | 3.786855 | 6.099529 |
| H | -0.585065 | 3.886297 | 6.017536 |
| H | 0.886270 | 4.733586 | 6.498067 |
| H | 0.727215 | 3.014173 | 6.842564 |
| C | 0.594196 | 4.249602 | 1.077448 |
| H | -0.287189 | 4.865135 | 1.280825 |
| H | 0.301628 | 3.422315 | 0.420651 |
| H | 1.316372 | 4.857183 | 0.521272 |
| C | 3.474715 | 0.597640 | -2.035676 |
| C | 2.623180 | 0.781924 | -3.138108 |
| C | 2.409537 | -0.283651 | -4.013572 |
| H | 1.744350 | -0.136821 | -4.862863 |
| C | 3.029103 | -1.518307 | -3.829597 |
| C | 3.895945 | -1.673328 | -2.748630 |
| H | 4.401807 | -2.625115 | -2.600982 |

| | | | |
|---|---|---|---|
| C | 4.140406 | -0.631745 | -1.855101 |
| C | 1.965236 | 2.110452 | -3.387069 |
| H | 2.695373 | 2.845271 | -3.748087 |
| H | 1.540727 | 2.522620 | -2.465830 |
| H | 1.173941 | 2.020198 | -4.136069 |
| C | 2.785639 | -2.653718 | -4.787667 |
| H | 2.613675 | -3.596930 | -4.256899 |
| H | 3.649164 | -2.804962 | -5.446449 |
| H | 1.918763 | -2.445786 | -5.421568 |
| C | 5.104363 | -0.823528 | -0.715139 |
| H | 5.803230 | 0.016301 | -0.642588 |
| H | 5.673490 | -1.747971 | -0.837084 |
| H | 4.589628 | -0.880304 | 0.253623 |
| C | 0.200536 | 0.566148 | 2.543067 |
| N | -1.925802 | 1.577398 | 3.472577 |
| H | -1.676180 | 2.846911 | 1.816226 |
| H | -3.337761 | 2.728257 | 2.455155 |
| H | -2.640933 | 1.402579 | 1.487021 |
| N | -0.658902 | 0.548976 | 4.913770 |
| H | 1.383210 | 0.335526 | 5.349531 |
| H | 0.529211 | -1.187633 | 5.029616 |
| H | 0.250059 | -0.327305 | 6.571196 |
| C | -0.777022 | 0.885534 | 3.612463 |
| C | -2.538702 | 1.697225 | 4.703163 |
| H | -3.740639 | 3.476009 | 4.641715 |
| H | -4.123838 | 2.357129 | 5.950845 |
| H | -4.628539 | 1.979425 | 4.302742 |
| C | -1.739778 | 1.046028 | 5.613698 |
| H | -1.092980 | 1.317538 | 7.639237 |
| H | -1.978843 | -0.194660 | 7.352470 |
| H | -2.839510 | 1.344751 | 7.401661 |
| C | -2.427763 | 2.175406 | 2.227661 |
| C | -3.823562 | 2.415758 | 4.903926 |
| C | -1.915946 | 0.864485 | 7.076754 |
| C | 0.449499 | -0.209409 | 5.506566 |
| H | 0.268976 | 0.690370 | -1.298555 |
| Si | -0.985018 | 0.076500 | -1.991628 |
| H | -0.520913 | 0.173022 | -3.391142 |
| O | -2.755507 | -0.946848 | -3.345143 |
| C | -3.429336 | -1.961391 | -3.027353 |
| O | -4.460753 | -2.059880 | -2.347499 |
| C | -0.953515 | -1.665861 | -1.297886 |
| C | -1.722347 | -2.059641 | -0.197311 |
| C | -0.088350 | -2.607124 | -1.875083 |
| C | -1.626487 | -3.352334 | 0.313693 |
| H | -2.409288 | -1.352856 | 0.262805 |
| C | 0.005112 | -3.900030 | -1.371524 |
| H | 0.543584 | -2.316452 | -2.713566 |
| C | -0.763594 | -4.274785 | -0.271025 |
| H | -2.230049 | -3.641179 | 1.170395 |
| H | 0.691412 | -4.610457 | -1.825074 |
| H | -0.680583 | -5.279395 | 0.135641 |
| C | -2.280381 | 1.307556 | -1.427217 |
| C | -1.878318 | 2.642633 | -1.289235 |
| C | -3.619216 | 0.985609 | -1.160470 |
| C | -2.777422 | 3.632823 | -0.904802 |
| H | -0.849130 | 2.920893 | -1.502749 |
| C | -4.514816 | 1.972420 | -0.754295 |
| H | -3.979814 | -0.027782 | -1.311760 |
| C | -4.099879 | 3.296094 | -0.628292 |
| H | -2.447840 | 4.666337 | -0.830841 |
| H | -5.550761 | 1.706036 | -0.563598 |
| H | -4.810007 | 4.066406 | -0.337606 |
| C | -2.843700 | -3.271376 | -3.546626 |
| C | -1.558466 | -4.730658 | -4.655312 |
| C | -2.223221 | -5.416905 | -3.676262 |
| N | -1.960820 | -3.409127 | -4.557752 |
| N | -3.018465 | -4.496917 | -3.013957 |
| C | -1.474248 | -2.337005 | -5.430997 |
| H | -2.249306 | -1.583676 | -5.556790 |
| H | -0.601120 | -1.856039 | -4.981546 |
| H | -1.206968 | -2.774402 | -6.394814 |
| C | -3.905945 | -4.851059 | -1.904023 |
| H | -4.251387 | -3.931580 | -1.434459 |
| H | -4.767053 | -5.405209 | -2.290292 |
| H | -3.353287 | -5.471169 | -1.194185 |
| C | -0.584441 | -5.205098 | -5.670141 |
| H | 0.352482 | -4.639375 | -5.625335 |
| H | -0.349582 | -6.257234 | -5.497198 |
| H | -0.978915 | -5.118689 | -6.688674 |
| C | -2.168437 | -6.846522 | -3.281806 |
| H | -1.743436 | -6.965836 | -2.277266 |
| H | -3.163251 | -7.304447 | -3.280118 |
| H | -1.544724 | -7.408009 | -3.978508 |

Calculated energies and coordinates of **INT_A**
Electronic energy ... -3041.42447827 Eh
Total Enthalpy ... -3040.34726393 Eh
Final Gibbs free energy ... -3040.51006015 Eh
CARTESIAN COORDINATES (ANGSTROEM)

| | | | |
|---|---|---|---|
| Sn | 1.937681 | -0.088136 | 0.465927 |
| O | 0.247046 | 0.763463 | 1.700263 |
| O | 1.191804 | -0.384622 | 3.396922 |
| C | 2.915831 | 1.919460 | 0.160174 |
| C | 2.960801 | 3.047651 | 0.998234 |
| C | 3.737952 | 4.157653 | 0.641630 |
| H | 3.769703 | 5.015764 | 1.309926 |
| C | 4.484346 | 4.163477 | -0.529694 |
| H | 5.087526 | 5.029460 | -0.787610 |
| C | 4.472969 | 3.045320 | -1.354615 |
| H | 5.070431 | 3.022867 | -2.263537 |
| C | 3.704038 | 1.933647 | -1.008243 |
| C | 2.260579 | 3.124848 | 2.316082 |
| C | 2.795575 | 2.469660 | 3.436567 |
| C | 2.188262 | 2.646015 | 4.682534 |
| H | 2.632608 | 2.165309 | 5.552972 |
| C | 1.056886 | 3.440953 | 4.844121 |
| C | 0.528259 | 4.070257 | 3.716127 |
| H | -0.351734 | 4.704472 | 3.821580 |
| C | 1.122837 | 3.938131 | 2.461484 |
| C | 4.009069 | 1.587577 | 3.323531 |
| H | 3.724560 | 0.579893 | 2.992368 |
| H | 4.503731 | 1.480624 | 4.292905 |
| H | 4.728598 | 1.982033 | 2.600847 |
| C | 0.460705 | 3.666981 | 6.208029 |
| H | -0.614274 | 3.867314 | 6.149593 |
| H | 0.927140 | 4.529894 | 6.698707 |
| H | 0.619683 | 2.803069 | 6.862791 |
| C | 0.547721 | 4.663357 | 1.274500 |
| H | -0.437864 | 5.079633 | 1.505020 |
| H | 0.459976 | 3.993670 | 0.412620 |
| H | 1.194363 | 5.490521 | 0.961110 |
| C | 3.736019 | 0.718150 | -1.879512 |
| C | 2.928978 | 0.645543 | -3.026522 |
| C | 2.936240 | -0.522588 | -3.790760 |
| H | 2.300960 | -0.578081 | -4.672910 |
| C | 3.736914 | -1.610945 | -3.454958 |
| C | 4.562025 | -1.507510 | -2.335006 |
| H | 5.210054 | -2.341197 | -2.071037 |
| C | 4.584377 | -0.358635 | -1.546825 |
| C | 2.088895 | 1.819418 | -3.446304 |
| H | 2.707732 | 2.585597 | -3.929047 |
| H | 1.617304 | 2.298601 | -2.583768 |
| H | 1.311982 | 1.518169 | -4.153934 |
| C | 3.743340 | -2.859667 | -4.294809 |
| H | 2.884952 | -2.888077 | -4.972287 |
| H | 3.721080 | -3.758514 | -3.669647 |
| H | 4.651050 | -2.914734 | -4.907517 |
| C | 5.517440 | -0.267937 | -0.369103 |
| H | 6.130755 | 0.637732 | -0.423937 |
| H | 6.178032 | -1.137258 | -0.328314 |
| H | 4.972342 | -0.214943 | 0.582058 |
| C | 0.328527 | 0.346694 | 2.908229 |
| N | -1.829993 | 1.594042 | 3.470743 |
| H | -1.277111 | 2.753139 | 1.810540 |
| H | -3.020768 | 2.743188 | 2.201753 |
| H | -2.260599 | 1.336632 | 1.415785 |
| N | -0.860391 | 0.572836 | 5.126543 |
| H | 1.099488 | 0.216605 | 5.789567 |
| H | 0.119934 | -1.239627 | 5.578447 |
| H | -0.202615 | -0.153245 | 6.964294 |
| C | -0.772581 | 0.827749 | 3.803876 |
| C | -2.593152 | 1.839933 | 4.596011 |

| | | | |
|---|---|---|---|
| H | -3.613894 | 3.699919 | 4.271912 |
| H | -4.266063 | 2.704383 | 5.574435 |
| H | -4.579884 | 2.261003 | 3.895429 |
| C | -1.982725 | 1.192742 | 5.641521 |
| H | -1.608912 | 1.584399 | 7.718773 |
| H | -2.515897 | 0.090596 | 7.407127 |
| H | -3.302802 | 1.659446 | 7.234460 |
| C | -2.115852 | 2.140814 | 2.138470 |
| C | -3.825833 | 2.669120 | 4.577107 |
| C | -2.366388 | 1.123515 | 7.074702 |
| C | 0.101253 | -0.203136 | 5.918715 |
| H | 0.559527 | 0.272872 | -0.922382 |
| Si | -0.699003 | -0.459054 | -1.776066 |
| H | 0.153594 | -0.368752 | -2.979905 |
| O | -1.985335 | -1.372158 | -2.995898 |
| C | -2.797875 | -2.315300 | -2.670741 |
| O | -3.507468 | -2.429227 | -1.679964 |
| C | -0.565991 | -2.030398 | -0.751847 |
| C | -1.171788 | -2.166463 | 0.501210 |
| C | 0.183292 | -3.105545 | -1.249077 |
| C | -1.030118 | -3.337538 | 1.240267 |
| H | -1.767910 | -1.349289 | 0.901719 |
| C | 0.332580 | -4.276366 | -0.509845 |
| H | 0.679912 | -3.017218 | -2.214743 |
| C | -0.275450 | -4.394407 | 0.737859 |
| H | -1.508271 | -3.426924 | 2.211934 |
| H | 0.936708 | -5.091338 | -0.900097 |
| H | -0.153291 | -5.303201 | 1.320661 |
| C | -1.861449 | 0.962797 | -1.352193 |
| C | -1.343937 | 2.262009 | -1.258230 |
| C | -3.243354 | 0.791014 | -1.182233 |
| C | -2.175203 | 3.356591 | -1.040963 |
| H | -0.272403 | 2.419512 | -1.356405 |
| C | -4.073440 | 1.881271 | -0.930549 |
| H | -3.674555 | -0.203554 | -1.245330 |
| C | -3.544699 | 3.167885 | -0.872688 |
| H | -1.753550 | 4.357344 | -1.001030 |
| H | -5.140906 | 1.725067 | -0.798077 |
| H | -4.196823 | 4.021069 | -0.704605 |
| C | -2.826832 | -3.452456 | -3.658194 |
| C | -2.827150 | -4.692440 | -5.514165 |
| C | -2.999719 | -5.533497 | -4.446167 |
| N | -2.711192 | -3.410204 | -4.998095 |
| N | -3.000007 | -4.740088 | -3.309035 |
| C | -2.556424 | -2.208202 | -5.821066 |
| H | -2.481470 | -1.341394 | -5.167225 |
| H | -1.641495 | -2.290614 | -6.414339 |
| H | -3.416684 | -2.110780 | -6.489438 |
| C | -3.118174 | -5.239274 | -1.932309 |
| H | -2.467529 | -4.661076 | -1.274903 |
| H | -4.150138 | -5.141280 | -1.586938 |
| H | -2.812397 | -6.286171 | -1.920947 |
| C | -2.766518 | -4.965442 | -6.971901 |
| H | -1.811049 | -4.645550 | -7.402335 |
| H | -2.874503 | -6.034289 | -7.160452 |
| H | -3.567229 | -4.448306 | -7.512548 |
| C | -3.155560 | -7.009347 | -4.398256 |
| H | -2.303467 | -7.492202 | -3.906317 |
| H | -4.066038 | -7.300393 | -3.863937 |
| H | -3.221457 | -7.409813 | -5.410870 |

Calculated energies and coordinates of **TS2**

Electronic energy ... -3041.42230439 Eh
Total Enthalpy ... -3040.34637604 Eh
Final Gibbs free energy ... -3040.50402540 Eh
CARTESIAN COORDINATES (ANGSTROEM)

| | | | |
|---|---|---|---|
| Sn | 1.439176 | -0.063164 | -0.463597 |
| O | 0.414277 | 0.263946 | 1.499414 |
| O | 0.813342 | -1.777153 | 2.416558 |
| C | 2.708083 | 1.760509 | -0.315306 |
| C | 3.097182 | 2.491251 | 0.818285 |
| C | 3.993549 | 3.560038 | 0.692743 |
| H | 4.289265 | 4.108719 | 1.584709 |
| C | 4.511787 | 3.915958 | -0.545890 |
| H | 5.210400 | 4.743512 | -0.627841 |
| C | 4.131228 | 3.204732 | -1.677467 |
| H | 4.525132 | 3.472247 | -2.655697 |
| C | 3.243377 | 2.134632 | -1.561272 |
| C | 2.583388 | 2.189435 | 2.187143 |
| C | 3.149071 | 1.159795 | 2.951190 |
| C | 2.691525 | 0.948918 | 4.254603 |
| H | 3.164362 | 0.175200 | 4.858191 |
| C | 1.681107 | 1.729456 | 4.811371 |
| C | 1.106033 | 2.727283 | 4.022818 |
| H | 0.315204 | 3.348629 | 4.442990 |
| C | 1.547204 | 2.972722 | 2.723449 |
| C | 4.222275 | 0.273867 | 2.381367 |
| H | 3.783031 | -0.492700 | 1.727115 |
| H | 4.768073 | -0.243387 | 3.175242 |
| H | 4.932460 | 0.842762 | 1.775472 |
| C | 1.263895 | 1.550761 | 6.247342 |
| H | 0.195189 | 1.751528 | 6.382425 |
| H | 1.808319 | 2.244625 | 6.899309 |
| H | 1.480005 | 0.538717 | 6.605084 |
| C | 0.904476 | 4.051498 | 1.893514 |
| H | 0.069614 | 4.516807 | 2.426027 |
| H | 0.542488 | 3.645802 | 0.940035 |
| H | 1.620501 | 4.839277 | 1.636268 |
| C | 2.843543 | 1.379632 | -2.788385 |
| C | 1.746979 | 1.821364 | -3.550155 |
| C | 1.379969 | 1.107741 | -4.690258 |
| H | 0.533272 | 1.461929 | -5.274799 |
| C | 2.088060 | -0.018415 | -5.106889 |
| C | 3.174891 | -0.441789 | -4.342018 |
| H | 3.750283 | -1.308892 | -4.664018 |
| C | 3.568408 | 0.241880 | -3.189553 |
| C | 1.006128 | 3.070134 | -3.163481 |
| H | 1.633354 | 3.955458 | -3.321892 |
| H | 0.739653 | 3.054194 | -2.101830 |
| H | 0.090441 | 3.185406 | -3.748752 |
| C | 1.721478 | -0.726713 | -6.382829 |
| H | 2.097234 | -1.754015 | -6.397176 |
| H | 2.157088 | -0.216709 | -7.250581 |
| H | 0.637564 | -0.744384 | -6.535475 |
| C | 4.764831 | -0.218324 | -2.403134 |
| H | 5.540588 | 0.553829 | -2.385963 |
| H | 5.193034 | -1.127631 | -2.832817 |
| H | 4.501550 | -0.418220 | -1.356625 |
| C | 0.302870 | -0.657512 | 2.372343 |
| N | -1.633949 | 0.629604 | 3.417461 |
| H | -1.253042 | 2.284356 | 2.173033 |
| H | -2.978750 | 1.846971 | 2.393471 |
| H | -1.918227 | 0.865464 | 1.344355 |
| N | -0.665225 | -0.819862 | 4.709206 |
| H | 1.270410 | -1.612261 | 4.810390 |
| H | -0.019275 | -2.822445 | 4.873925 |
| H | 0.289345 | -1.777818 | 6.295466 |
| C | -0.638878 | -0.272560 | 3.480425 |
| C | -2.296861 | 0.669564 | 4.632957 |
| H | -3.165703 | 2.627564 | 4.738930 |
| H | -3.761216 | 1.476707 | 5.938616 |
| H | -4.301649 | 1.349451 | 4.264134 |
| C | -1.683938 | -0.246080 | 5.449821 |
| H | -1.119389 | -0.323192 | 7.518925 |
| H | -2.127682 | -1.680206 | 6.984298 |
| H | -2.842795 | -0.084563 | 7.221200 |
| C | -1.967595 | 1.461622 | 2.256615 |
| C | -3.440133 | 1.578231 | 4.900889 |
| C | -1.952943 | -0.606250 | 6.865082 |
| C | 0.278425 | -1.825272 | 5.206117 |
| H | -0.022670 | 0.829358 | -1.109634 |
| Si | -1.621449 | -0.310268 | -2.539814 |
| H | -0.233746 | -0.711066 | -2.778093 |
| O | -2.440106 | -1.264173 | -3.777970 |
| C | -2.597034 | -2.571490 | -3.841638 |
| O | -3.371626 | -3.135493 | -4.576681 |
| C | -2.405168 | -0.869539 | -0.934916 |
| C | -3.733554 | -0.496812 | -0.674048 |
| C | -1.750280 | -1.679278 | 0.004652 |
| C | -4.384189 | -0.909978 | 0.485396 |
| H | -4.263740 | 0.136174 | -1.383247 |
| C | -2.397797 | -2.095373 | 1.165971 |

| | | | |
|---|---|---|---|
| H | -0.714595 | -1.973690 | -0.159352 |
| C | -3.715754 | -1.710215 | 1.408877 |
| H | -5.413039 | -0.609848 | 0.665034 |
| H | -1.860652 | -2.705838 | 1.887590 |
| H | -4.220982 | -2.034830 | 2.315385 |
| C | -2.181400 | 1.380404 | -3.047317 |
| C | -2.350105 | 2.395334 | -2.096763 |
| C | -2.405184 | 1.678181 | -4.397616 |
| C | -2.721464 | 3.677256 | -2.485591 |
| H | -2.178225 | 2.178562 | -1.045505 |
| C | -2.770011 | 2.961474 | -4.788200 |
| H | -2.303038 | 0.900661 | -5.151369 |
| C | -2.925651 | 3.963352 | -3.833990 |
| H | -2.847443 | 4.456244 | -1.739459 |
| H | -2.939251 | 3.179647 | -5.838640 |
| H | -3.209101 | 4.966132 | -4.140166 |
| C | -1.761245 | -3.395201 | -2.895400 |
| C | -0.055955 | -4.352313 | -1.827117 |
| C | -1.217951 | -4.776163 | -1.237221 |
| N | -0.422320 | -3.499167 | -2.859361 |
| N | -2.260977 | -4.188378 | -1.935886 |
| C | 0.518963 | -2.949908 | -3.840352 |
| H | -0.022124 | -2.332249 | -4.558550 |
| H | 1.267991 | -2.329206 | -3.335453 |
| H | 1.005443 | -3.776002 | -4.367769 |
| C | -3.673380 | -4.285008 | -1.564782 |
| H | -4.280827 | -3.855851 | -2.360005 |
| H | -3.942505 | -5.337118 | -1.440705 |
| H | -3.839807 | -3.740285 | -0.630006 |
| C | 1.364604 | -4.627818 | -1.507789 |
| H | 1.881890 | -3.701190 | -1.227687 |
| H | 1.433050 | -5.319526 | -0.668924 |
| H | 1.892584 | -5.077126 | -2.358625 |
| C | -1.450630 | -5.666628 | -0.073713 |
| H | -2.059699 | -5.170925 | 0.687147 |
| H | -1.962177 | -6.589222 | -0.368431 |
| H | -0.497257 | -5.941157 | 0.383906 |

Calculated energies and coordinates of **INT_B**

Electronic energy ... -1325.04745581 Eh
Total Enthalpy ... -1324.63125306 Eh
Final Gibbs free energy ... -1324.70989275 Eh
CARTESIAN COORDINATES (ANGSTROEM)

| | | | |
|---|---|---|---|
| O | -0.049662 | -0.314659 | 0.571652 |
| O | -0.958281 | 1.548539 | 1.479133 |
| C | -1.039511 | 0.516550 | 0.847425 |
| H | -4.286104 | -2.993418 | -1.547206 |
| H | -4.816142 | 1.792242 | 1.750204 |
| H | -0.780887 | -1.134571 | -1.709116 |
| H | -6.340921 | 0.149383 | 1.048389 |
| C | -4.524030 | -1.959354 | -1.817960 |
| H | -5.609771 | -1.859948 | -1.812296 |
| H | -4.182454 | -1.784291 | -2.844198 |
| C | -5.964918 | 0.331660 | 0.035625 |
| H | -6.138542 | 1.384234 | -0.211426 |
| H | -6.561227 | -0.270731 | -0.650070 |
| C | -3.745278 | 1.703711 | 1.572831 |
| H | -3.228133 | 1.485591 | 2.507175 |
| H | -3.350664 | 2.636626 | 1.165635 |
| H | -1.096128 | -2.433809 | -0.541533 |
| H | -2.049543 | -2.338448 | -2.047133 |
| N | -2.568860 | -0.917987 | -0.611365 |
| N | -3.528775 | 0.607874 | 0.614241 |
| C | -2.337206 | 0.060352 | 0.293230 |
| C | -3.923557 | -0.991214 | -0.865550 |
| C | -4.529643 | -0.027678 | -0.088944 |
| C | -1.556229 | -1.760816 | -1.267184 |
| Si | 1.605244 | 0.087067 | 0.993462 |
| H | 1.582022 | 0.343236 | 2.449586 |
| C | 2.103451 | 1.587615 | 0.032020 |
| C | 2.480326 | -1.472026 | 0.499352 |
| C | 3.737297 | -1.424481 | -0.119825 |
| C | 1.924228 | -2.728121 | 0.789761 |
| C | 2.597673 | -3.898264 | 0.460565 |
| C | 3.842521 | -3.832261 | -0.161315 |
| C | 4.412660 | -2.596226 | -0.447774 |
| H | 4.195257 | -0.464686 | -0.347112 |
| H | 4.371057 | -4.746346 | -0.415498 |
| H | 5.386299 | -2.543610 | -0.925598 |
| H | 0.961682 | -2.795289 | 1.293721 |
| H | 2.159090 | -4.863474 | 0.696696 |
| C | 2.333026 | 1.514103 | -1.351347 |
| H | 2.249991 | 0.559751 | -1.868511 |
| C | 2.686855 | 2.648653 | -2.071851 |
| C | 2.820166 | 3.872081 | -1.419186 |
| H | 2.868206 | 2.579696 | -3.140429 |
| C | 2.595437 | 3.961631 | -0.048550 |
| H | 3.104361 | 4.757250 | -1.980853 |
| C | 2.235918 | 2.828034 | 0.672058 |
| H | 2.702240 | 4.915611 | 0.459011 |
| H | 2.054104 | 2.908391 | 1.741054 |

Calculated energies and coordinates of **INT_C**

Electronic energy ... -1716.33206638 Eh
Total Enthalpy ... -1715.67322741 Eh
Final Gibbs free energy ... -1715.78268516 Eh
CARTESIAN COORDINATES (ANGSTROEM)

| | | | |
|---|---|---|---|
| Sn | 3.829209 | 11.931927 | 8.576658 |
| O | 2.220643 | 13.128437 | 9.652846 |
| O | 3.104913 | 13.121067 | 11.748449 |
| C | 4.887783 | 13.734116 | 7.710236 |
| C | 4.773188 | 15.104639 | 7.996605 |
| C | 5.615824 | 16.038953 | 7.377439 |
| H | 5.498381 | 17.094159 | 7.618986 |
| C | 6.579862 | 15.637335 | 6.462553 |
| H | 7.225022 | 16.371424 | 5.987303 |
| C | 6.703485 | 14.286674 | 6.158508 |
| H | 7.445238 | 13.948066 | 5.437303 |
| C | 5.872245 | 13.351768 | 6.776432 |
| C | 3.754783 | 15.673470 | 8.931050 |
| C | 4.101457 | 15.966101 | 10.257928 |
| C | 3.177552 | 16.619933 | 11.076160 |
| H | 3.465940 | 16.874853 | 12.095326 |
| C | 1.912435 | 16.975721 | 10.613688 |
| C | 1.571082 | 16.641461 | 9.302921 |
| H | 0.585269 | 16.909957 | 8.922267 |
| C | 2.474443 | 15.998578 | 8.455058 |
| C | 5.439737 | 15.552500 | 10.803014 |
| H | 5.533700 | 14.460564 | 10.780021 |
| H | 5.563542 | 15.884695 | 11.837601 |
| H | 6.260066 | 15.957944 | 10.201676 |
| C | 0.963165 | 17.749837 | 11.490091 |
| H | -0.082031 | 17.528746 | 11.248496 |
| H | 1.106439 | 18.830385 | 11.364331 |
| H | 1.127854 | 17.525733 | 12.550169 |
| C | 2.075951 | 15.626374 | 7.054256 |
| H | 1.080570 | 16.010562 | 6.809369 |
| H | 2.078731 | 14.534316 | 6.935543 |
| H | 2.790405 | 16.016859 | 6.321812 |
| C | 6.017394 | 11.907310 | 6.417725 |
| C | 5.272509 | 11.372359 | 5.352698 |
| C | 5.405685 | 10.017782 | 5.044017 |
| H | 4.821275 | 9.603669 | 4.224313 |
| C | 6.266112 | 9.187035 | 5.757285 |
| C | 7.014887 | 9.740198 | 6.795550 |
| H | 7.696110 | 9.104888 | 7.358946 |
| C | 6.905271 | 11.085952 | 7.137819 |
| C | 4.361854 | 12.252006 | 4.539800 |
| H | 4.940763 | 12.954847 | 3.928247 |
| H | 3.713308 | 12.846928 | 5.190034 |
| H | 3.734280 | 11.653587 | 3.873725 |
| C | 6.368015 | 7.719647 | 5.437964 |
| H | 6.022049 | 7.507807 | 4.422112 |
| H | 5.753468 | 7.128105 | 6.127770 |
| H | 7.398696 | 7.362989 | 5.532146 |
| C | 7.716684 | 11.653092 | 8.269736 |
| H | 8.347922 | 12.481815 | 7.930242 |
| H | 8.355505 | 10.886317 | 8.715881 |
| H | 7.062136 | 12.055253 | 9.052734 |
| C | 2.229983 | 13.325854 | 10.909616 |

| | | | |
|---|---|---|---|
| N | -0.230318 | 14.010254 | 10.700241 |
| H | 0.154037 | 14.238759 | 8.645854 |
| H | -1.499249 | 13.693150 | 9.079017 |
| H | -0.108403 | 12.570523 | 9.164416 |
| N | 0.696246 | 14.413393 | 12.620991 |
| H | 2.623349 | 14.897736 | 13.286050 |
| H | 1.886425 | 13.525710 | 14.119092 |
| H | 1.298221 | 15.184719 | 14.460078 |
| C | 0.920887 | 13.904186 | 11.394155 |
| C | -1.195003 | 14.608478 | 11.494015 |
| H | -2.572684 | 15.558283 | 10.149834 |
| H | -3.143301 | 15.386235 | 11.810856 |
| H | -3.111042 | 13.977995 | 10.747786 |
| C | -0.611227 | 14.860066 | 12.707105 |
| H | -0.675519 | 16.453469 | 14.144035 |
| H | -1.069750 | 14.854566 | 14.805101 |
| H | -2.232170 | 15.704161 | 13.784468 |
| C | -0.436642 | 13.600174 | 9.305467 |
| C | -2.574360 | 14.893459 | 11.020980 |
| C | -1.169875 | 15.499499 | 13.925454 |
| C | 1.689691 | 14.512764 | 13.694938 |
| H | 2.631239 | 11.961092 | 7.238805 |

Calculated energies and coordinates of **TS3**

Electronic energy ... -3041.42997837 Eh
Total Enthalpy ... -3040.35489622 Eh
Final Gibbs free energy ... -3040.51343766 Eh
CARTESIAN COORDINATES (ANGSTROEM)

| | | | |
|---|---|---|---|
| Sn | 0.649834 | 1.221211 | 0.581754 |
| O | -1.034665 | -0.047720 | 1.298486 |
| O | -2.083215 | 0.859373 | -0.463813 |
| C | 1.568246 | -0.315825 | -0.754138 |
| C | 1.027355 | -1.461615 | -1.365736 |
| C | 1.777570 | -2.170326 | -2.314489 |
| H | 1.342638 | -3.059819 | -2.765636 |
| C | 3.055781 | -1.765294 | -2.673837 |
| H | 3.619778 | -2.326821 | -3.413255 |
| C | 3.608763 | -0.643298 | -2.070274 |
| H | 4.614516 | -0.315760 | -2.325595 |
| C | 2.872934 | 0.069942 | -1.123374 |
| C | -0.312623 | -2.046461 | -1.050813 |
| C | -1.376636 | -1.889053 | -1.954337 |
| C | -2.570943 | -2.576628 | -1.728560 |
| H | -3.384541 | -2.470524 | -2.445240 |
| C | -2.731203 | -3.428139 | -0.636630 |
| C | -1.671457 | -3.553130 | 0.260326 |
| H | -1.770337 | -4.226001 | 1.111060 |
| C | -0.467294 | -2.872163 | 0.074705 |
| C | -1.241073 | -0.982821 | -3.147079 |
| H | -0.471634 | -1.341748 | -3.838835 |
| H | -0.945803 | 0.024113 | -2.833120 |
| H | -2.184019 | -0.913344 | -3.696772 |
| C | -3.990918 | -4.234188 | -0.463825 |
| H | -4.194901 | -4.443882 | 0.591305 |
| H | -3.907085 | -5.200542 | -0.975998 |
| H | -4.859058 | -3.718758 | -0.887266 |
| C | 0.644184 | -3.018908 | 1.076665 |
| H | 0.753445 | -2.093903 | 1.657126 |
| H | 1.602376 | -3.210185 | 0.581916 |
| H | 0.437878 | -3.835446 | 1.775044 |
| C | 3.521464 | 1.241483 | -0.457706 |
| C | 4.290194 | 1.032512 | 0.700450 |
| C | 4.934306 | 2.117355 | 1.295430 |
| H | 5.564738 | 1.938694 | 2.165182 |
| C | 4.828452 | 3.406580 | 0.774510 |
| C | 4.061292 | 3.594882 | -0.374485 |
| H | 3.981362 | 4.591584 | -0.803813 |
| C | 3.417399 | 2.532506 | -1.008461 |
| C | 4.456716 | -0.354044 | 1.261392 |
| H | 5.024394 | -0.327422 | 2.196374 |
| H | 4.987933 | -1.002864 | 0.555890 |
| H | 3.482686 | -0.824203 | 1.442020 |
| C | 5.527683 | 4.568937 | 1.427200 |
| H | 5.875531 | 5.290819 | 0.682675 |
| H | 6.396355 | 4.239529 | 2.005818 |
| H | 4.855849 | 5.112048 | 2.105098 |
| C | 2.642717 | 2.759636 | -2.276803 |
| H | 3.072472 | 2.185113 | -3.104800 |
| H | 2.639913 | 3.816888 | -2.551927 |
| H | 1.602410 | 2.427505 | -2.175570 |
| C | -2.051440 | 0.153658 | 0.548424 |
| N | -3.534750 | -1.301105 | 2.056319 |
| H | -1.616470 | -1.237333 | 2.915990 |
| H | -2.461880 | -2.799491 | 3.047056 |
| H | -2.968648 | -1.422252 | 4.066745 |
| N | -4.477883 | -0.454311 | 0.292284 |
| H | -4.571488 | 1.347240 | -0.794167 |
| H | -5.718688 | 0.067683 | -1.294571 |
| H | -3.979669 | -0.048093 | -1.707598 |
| C | -3.313742 | -0.528353 | 0.973436 |
| C | -4.849191 | -1.721022 | 2.061392 |
| H | -5.361661 | -2.078462 | 4.110228 |
| H | -4.840006 | -3.519810 | 3.227417 |
| H | -6.439915 | -2.832141 | 2.928395 |
| C | -5.446172 | -1.187034 | 0.949231 |
| H | -7.420730 | -1.927357 | 1.160695 |
| H | -6.898078 | -1.786417 | -0.517573 |
| H | -7.340649 | -0.335913 | 0.402447 |
| C | -2.578355 | -1.712637 | 3.087613 |
| C | -5.399056 | -2.581874 | 3.137015 |
| C | -6.845529 | -1.311133 | 0.468335 |
| C | -4.702818 | 0.276427 | -0.958980 |
| H | 1.398333 | 0.559841 | 2.189446 |
| C | 0.978669 | 0.286197 | 3.823654 |
| O | 0.508585 | -0.843384 | 3.946180 |
| O | 0.228000 | 1.427700 | 3.987748 |
| C | 2.403708 | 0.551620 | 4.178619 |
| Si | -1.194349 | 1.552992 | 4.888065 |
| N | 3.024947 | 1.742607 | 4.297228 |
| N | 3.284917 | -0.403387 | 4.536225 |
| H | -1.365704 | 0.329436 | 5.707785 |
| C | -2.677670 | 1.821271 | 3.790853 |
| C | -0.877575 | 3.041706 | 5.967527 |
| C | 4.315250 | 1.542804 | 4.764179 |
| C | 2.484514 | 3.050526 | 3.905288 |
| C | 3.048716 | -1.851986 | 4.507523 |
| C | 4.482373 | 0.191910 | 4.909654 |
| C | -2.586271 | 2.545445 | 2.595118 |
| C | -3.939723 | 1.369454 | 4.201928 |
| C | -1.556100 | 4.253574 | 5.791032 |
| C | 0.108547 | 2.967948 | 6.964622 |
| C | 5.243341 | 2.664248 | 5.056022 |
| H | 3.324634 | 3.728908 | 3.757672 |
| H | 1.939099 | 2.948863 | 2.966432 |
| H | 1.815602 | 3.437948 | 4.678928 |
| H | 2.375343 | -2.145208 | 5.315462 |
| H | 2.592014 | -2.131174 | 3.558050 |
| H | 4.010377 | -2.352675 | 4.620773 |
| C | 5.662622 | -0.584769 | 5.367022 |
| H | -1.618015 | 2.897752 | 2.243595 |
| C | -3.719261 | 2.804878 | 1.829979 |
| C | -5.075146 | 1.624351 | 3.438202 |
| H | -4.041506 | 0.819054 | 5.136421 |
| C | -1.255008 | 5.360782 | 6.580616 |
| H | -2.329936 | 4.332829 | 5.030709 |
| C | 0.410634 | 4.071043 | 7.754663 |
| H | 0.641874 | 2.032434 | 7.133179 |
| H | 4.810867 | 3.358838 | 5.784793 |
| H | 6.175238 | 2.280044 | 5.473118 |
| H | 5.492597 | 3.234851 | 4.154648 |
| H | 6.461977 | 0.096269 | 5.662000 |
| H | 5.426388 | -1.214258 | 6.231817 |
| H | 6.057780 | -1.230319 | 4.573946 |
| C | -4.964456 | 2.344187 | 2.250886 |
| H | -3.623868 | 3.364412 | 0.903813 |
| H | -6.047299 | 1.273835 | 3.774509 |
| C | -0.271578 | 5.271048 | 7.560402 |
| H | -1.791197 | 6.293764 | 6.433071 |
| H | 1.168310 | 3.995954 | 8.529702 |
| H | -5.852060 | 2.556955 | 1.660251 |
| H | -0.040968 | 6.132999 | 8.179914 |

Calculated energies and coordinates of **INT_D**

Electronic energy ... -1325.80488896 Eh
Total Enthalpy ... -1325.38013281 Eh
Final Gibbs free energy ... -1325.45809480 Eh
CARTESIAN COORDINATES (ANGSTROEM)

| | | | |
|---|---|---|---|
| O | -0.348945 | -1.388986 | 1.128033 |
| O | -0.615975 | -1.355306 | -1.191575 |
| C | 0.132839 | -1.796623 | -0.233560 |
| H | 5.315963 | 0.845862 | -1.094996 |
| H | 1.314599 | -2.577635 | 1.989112 |
| H | 2.062531 | -0.046619 | -3.198011 |
| H | 5.272229 | -2.073875 | 1.267576 |
| C | 4.335495 | 0.752412 | -1.565521 |
| H | 4.486288 | 0.405295 | -2.594088 |
| H | 3.884375 | 1.750606 | -1.613347 |
| C | 4.958862 | -1.028289 | 1.168064 |
| H | 5.780372 | -0.477621 | 0.706378 |
| H | 4.814489 | -0.626215 | 2.177981 |
| C | 2.379695 | -2.468470 | 1.786231 |
| H | 2.831463 | -3.441045 | 1.561596 |
| H | 2.864136 | -2.041543 | 2.668180 |
| H | 0.481581 | -0.194467 | -2.327451 |
| H | 1.482995 | 1.282997 | -2.143277 |
| N | 2.157958 | -0.428576 | -1.139921 |
| N | 2.547990 | -1.558691 | 0.655665 |
| C | 1.593297 | -1.255291 | -0.246945 |
| C | 3.480138 | -0.177838 | -0.787796 |
| C | 3.731394 | -0.899312 | 0.342123 |
| C | 1.500821 | 0.199209 | -2.291362 |
| H | 0.227779 | -2.909380 | -0.138563 |
| Si | -1.326980 | -0.041046 | 1.101890 |
| H | -1.426252 | 0.275479 | 2.557643 |
| C | -3.108093 | -0.147084 | 0.516001 |
| C | -0.445575 | 1.434877 | 0.320923 |
| C | -1.008955 | 2.189890 | -0.715058 |
| C | 0.814769 | 1.819998 | 0.800275 |
| C | 1.495333 | 2.908159 | 0.259776 |
| C | 0.914545 | 3.648617 | -0.768464 |
| C | -0.341646 | 3.287891 | -1.251940 |
| H | -1.983885 | 1.907888 | -1.106837 |
| H | 1.433963 | 4.508317 | -1.184058 |
| H | -0.800507 | 3.864006 | -2.051299 |
| H | 1.273151 | 1.257962 | 1.614127 |
| H | 2.469796 | 3.190322 | 0.652007 |
| C | -3.537290 | -0.783297 | -0.657848 |
| H | -2.799613 | -1.298139 | -1.266590 |
| C | -4.878819 | -0.757225 | -1.028932 |
| C | -5.816110 | -0.090419 | -0.244002 |
| H | -5.195125 | -1.261057 | -1.938844 |
| C | -5.408081 | 0.548196 | 0.923128 |
| H | -6.862351 | -0.072138 | -0.538343 |
| C | -4.068271 | 0.512991 | 1.298821 |
| H | -6.133696 | 1.066380 | 1.544593 |
| H | -3.765132 | 1.005100 | 2.221465 |

Calculated energies and coordinates of **TS4**
Electronic energy ... -1325.79283035 Eh
Total Enthalpy ... -1325.36937631 Eh
Final Gibbs free energy ... -1325.44736048 Eh
CARTESIAN COORDINATES (ANGSTROEM)

| | | | |
|---|---|---|---|
| O | -0.622189 | -1.959877 | 0.565532 |
| O | -0.829826 | -1.448482 | -1.638875 |
| C | -0.328814 | -2.182739 | -0.777433 |
| H | 4.722426 | 1.565709 | -1.110927 |
| H | 1.484087 | -2.916572 | 1.511433 |
| H | 2.592682 | -0.839122 | -3.633664 |
| H | 4.939933 | -0.758992 | 1.735414 |
| C | 3.974965 | 1.088881 | -1.748858 |
| H | 4.491986 | 0.685500 | -2.628045 |
| H | 3.284369 | 1.868364 | -2.091285 |
| C | 4.277860 | 0.043587 | 1.387951 |
| H | 4.905797 | 0.871125 | 1.050684 |
| H | 3.701261 | 0.396335 | 2.251829 |
| C | 2.243707 | -2.155688 | 1.684507 |
| H | 3.178854 | -2.629060 | 2.003469 |
| H | 1.891645 | -1.488413 | 2.480076 |
| H | 0.935843 | -1.260975 | -3.084551 |
| H | 1.452859 | 0.435661 | -3.114218 |
| N | 2.241109 | -0.737736 | -1.561435 |
| N | 2.444483 | -1.419224 | 0.446624 |
| C | 1.728690 | -1.631947 | -0.685115 |
| C | 3.256878 | 0.029774 | -0.996457 |
| C | 3.386351 | -0.404519 | 0.288461 |
| C | 1.783964 | -0.593243 | -2.936090 |
| H | -0.128321 | -3.247795 | -0.936268 |
| Si | -1.228550 | -0.451686 | 0.999448 |
| H | -1.118868 | -0.485546 | 2.483407 |
| C | -3.034688 | -0.167754 | 0.594471 |
| C | -0.146051 | 0.983171 | 0.445744 |
| C | -0.261625 | 1.568161 | -0.824072 |
| C | 0.762911 | 1.549223 | 1.349165 |
| C | 1.536443 | 2.655241 | 1.002609 |
| C | 1.403594 | 3.223356 | -0.259388 |
| C | 0.502504 | 2.676519 | -1.172226 |
| H | -0.957566 | 1.142396 | -1.541752 |
| H | 1.992799 | 4.096061 | -0.529691 |
| H | 0.386918 | 3.126258 | -2.155528 |
| H | 0.857508 | 1.128973 | 2.349476 |
| H | 2.231517 | 3.080739 | 1.722083 |
| C | -3.650371 | -0.524740 | -0.614074 |
| H | -3.066675 | -1.028061 | -1.378751 |
| C | -4.994245 | -0.239097 | -0.837097 |
| C | -5.746905 | 0.413085 | 0.135699 |
| H | -5.456477 | -0.527437 | -1.777501 |
| C | -5.152052 | 0.775999 | 1.340337 |
| H | -6.795846 | 0.634489 | -0.042606 |
| C | -3.810568 | 0.483575 | 1.565736 |
| H | -5.734379 | 1.280438 | 2.106656 |
| H | -3.362375 | 0.762348 | 2.518053 |

Calculated energies and coordinates of $IMe_4$
Electronic energy ... -383.32167062 Eh
Total Enthalpy ... -383.12957541 Eh
Final Gibbs free energy ... -383.17542336 Eh
CARTESIAN COORDINATES (ANGSTROEM)

| | | | |
|---|---|---|---|
| N | -1.058056 | -0.708079 | 0.000141 |
| N | 1.058061 | -0.708052 | 0.000183 |
| C | -2.436046 | -1.163330 | 0.000211 |
| H | -2.969552 | -0.811088 | -0.890945 |
| H | -2.969696 | -0.810324 | 0.890977 |
| H | -2.417824 | -2.254157 | 0.000669 |
| C | -0.680794 | 0.636085 | -0.000149 |
| C | 0.680765 | 0.636102 | -0.000130 |
| C | 2.436062 | -1.163268 | 0.000169 |
| H | 2.969862 | -0.809906 | 0.890702 |
| H | 2.969400 | -0.811355 | -0.891220 |
| H | 2.417868 | -2.254096 | 0.001036 |
| C | 1.659771 | 1.754891 | -0.000212 |
| H | 2.308634 | 1.729860 | 0.884111 |
| H | 1.140485 | 2.716391 | -0.001491 |
| H | 2.310166 | 1.728347 | -0.883353 |
| C | -1.659828 | 1.754850 | -0.000281 |
| H | -2.309997 | 1.728454 | -0.883595 |
| H | -1.140566 | 2.716363 | -0.001244 |
| H | -2.308916 | 1.729638 | 0.883870 |
| C | 0.000013 | -1.571661 | 0.000375 |

Calculated energies and coordinates of $IMe_4CO_2$
Electronic energy ... -571.91928470 Eh
Total Enthalpy ... -571.70945168 Eh
Final Gibbs free energy ... -571.76191821 Eh
CARTESIAN COORDINATES (ANGSTROEM)

| | | | |
|---|---|---|---|
| O | 0.816022 | 9.338188 | 3.651725 |
| C | 0.954329 | 8.242167 | 4.219692 |
| O | 0.166653 | 7.314350 | 4.467501 |
| N | 3.303293 | 8.900886 | 5.122384 |
| N | 3.016385 | 6.780601 | 4.833655 |
| C | 2.405154 | 7.978151 | 4.718984 |
| C | 4.489562 | 8.284340 | 5.501059 |

| | | | |
|---|---|---|---|
| C | 4.312213 | 6.945027 | 5.307064 |
| C | 3.067951 | 10.342673 | 5.209336 |
| H | 3.080303 | 10.656068 | 6.258187 |
| H | 2.100108 | 10.545475 | 4.746631 |
| H | 3.853593 | 10.871922 | 4.662015 |
| C | 5.653529 | 9.051886 | 6.011723 |
| H | 6.467334 | 8.372963 | 6.272720 |
| H | 5.395554 | 9.625764 | 6.909222 |
| H | 6.032654 | 9.758594 | 5.264541 |
| C | 5.232370 | 5.797484 | 5.509878 |
| H | 4.847069 | 5.095549 | 6.258536 |
| H | 6.206268 | 6.150982 | 5.853122 |
| H | 5.388099 | 5.237642 | 4.580575 |
| C | 2.439033 | 5.487130 | 4.465849 |
| H | 2.521790 | 4.798675 | 5.312149 |
| H | 2.976623 | 5.073964 | 3.606336 |
| H | 1.385617 | 5.652868 | 4.232144 |

Calculated energies and coordinates of $H_2SiPh_2$
Electronic energy ... -753.85890538 Eh
Total Enthalpy ... -753.64849461 Eh
Final Gibbs free energy ... -753.69840992 Eh
CARTESIAN COORDINATES (ANGSTROEM)

| | | | |
|---|---|---|---|
| H | -1.106527 | -0.496432 | -1.322883 |
| Si | -2.487240 | 0.033120 | -1.149112 |
| H | -2.424945 | 1.516897 | -1.255968 |
| C | -3.099338 | -0.510000 | 0.538980 |
| C | -3.637311 | -0.587169 | -2.495194 |
| C | -4.166881 | -1.884640 | -2.441172 |
| C | -3.979906 | 0.222875 | -3.585777 |
| C | -4.817272 | -0.248071 | -4.593424 |
| C | -5.329714 | -1.539948 | -4.524639 |
| C | -5.004228 | -2.358218 | -3.445629 |
| H | -3.929165 | -2.530029 | -1.597634 |
| H | -5.985505 | -1.908306 | -5.308709 |
| H | -5.405963 | -3.366001 | -3.386457 |
| H | -3.591552 | 1.237277 | -3.647751 |
| H | -5.073740 | 0.395218 | -5.430624 |
| C | -4.464947 | -0.459228 | 0.854152 |
| H | -5.180053 | -0.125575 | 0.104551 |
| C | -4.924203 | -0.841284 | 2.110100 |
| C | -4.023833 | -1.284231 | 3.076017 |
| H | -5.985977 | -0.796153 | 2.336175 |
| C | -2.665520 | -1.345531 | 2.780335 |
| H | -4.381866 | -1.584998 | 4.056765 |
| C | -2.209443 | -0.962839 | 1.521783 |
| H | -1.960746 | -1.696087 | 3.529269 |
| H | -1.145775 | -1.021992 | 1.300936 |

Calculated energies and coordinates of $HCO_2SiHPh_2$
Electronic energy ... -942.45802637 Eh
Total Enthalpy ... -942.22813805 Eh
Final Gibbs free energy ... -942.28542377 Eh
CARTESIAN COORDINATES (ANGSTROEM)

| | | | |
|---|---|---|---|
| H | 1.018553 | -3.616374 | -1.699797 |
| C | 1.127897 | -2.897388 | -0.870504 |
| O | 0.071791 | -2.076395 | -0.805280 |
| O | 2.076233 | -2.862401 | -0.125856 |
| Si | 0.019559 | -0.864238 | 0.409367 |
| H | 0.198580 | -1.530019 | 1.720426 |
| C | -1.687323 | -0.142756 | 0.206464 |
| C | 1.335124 | 0.427517 | 0.144071 |
| C | -2.035685 | 0.997364 | 0.946054 |
| C | -2.647541 | -0.701218 | -0.646732 |
| C | 2.601259 | 0.293130 | 0.731763 |
| C | 1.081660 | 1.553957 | -0.651713 |
| H | -1.305352 | 1.459126 | 1.608797 |
| C | -3.302929 | 1.559110 | 0.840411 |
| C | -3.916101 | -0.138161 | -0.756029 |
| H | -2.400299 | -1.581477 | -1.233405 |
| C | 3.583419 | 1.256380 | 0.530019 |
| H | 2.821195 | -0.580656 | 1.339714 |
| C | 2.065101 | 2.516055 | -0.857117 |
| H | 0.102647 | 1.683773 | -1.108228 |
| C | -4.245616 | 0.990658 | -0.012599 |
| H | -3.555321 | 2.441967 | 1.420874 |
| H | -4.648861 | -0.581814 | -1.424283 |
| C | 3.316171 | 2.367893 | -0.265118 |
| H | 4.560005 | 1.139288 | 0.991147 |
| H | 1.854892 | 3.383399 | -1.476596 |
| H | -5.235957 | 1.429163 | -0.098293 |
| H | 4.084136 | 3.120331 | -0.422633 |

Calculated energies and coordinates of $[BArF]^-$
Electronic energy ... -3647.80715843 Eh
Total Enthalpy ... -3647.35676866 Eh
Final Gibbs free energy ... -3647.48969397 Eh
CARTESIAN COORDINATES (ANGSTROEM)

| | | | |
|---|---|---|---|
| F | -1.545305 | -2.882373 | 8.060532 |
| F | 2.296378 | -1.423679 | 0.829475 |
| F | -2.358456 | -3.056745 | 6.050870 |
| F | -0.211089 | -2.860052 | 6.337852 |
| F | 2.457838 | 0.552458 | -0.060054 |
| F | 1.198431 | -0.960557 | -0.993260 |
| F | 4.077953 | 4.211132 | 2.484189 |
| F | -3.834863 | 1.023600 | 0.374767 |
| F | 5.297659 | 0.611899 | 7.509212 |
| F | 3.628913 | -0.772215 | 7.296910 |
| F | -1.134221 | 7.715542 | 6.558850 |
| F | -4.154713 | -0.246824 | 2.110469 |
| F | 3.426082 | 0.985080 | 8.558907 |
| C | -0.325027 | 1.144843 | 3.065756 |
| C | -0.560775 | 3.514561 | 4.329508 |
| C | -0.963181 | 1.179548 | 5.617332 |
| C | 1.502361 | 1.974455 | 4.866879 |
| C | -0.258899 | 4.417377 | 5.364372 |
| H | 0.295684 | 4.061134 | 6.230187 |
| C | -1.542259 | -0.930768 | 6.711423 |
| C | 0.696099 | 0.698234 | 2.223735 |
| H | 1.733926 | 0.873948 | 2.497046 |
| C | 0.426453 | 0.017011 | 1.033538 |
| C | 2.063959 | 1.267468 | 5.930380 |
| H | 1.428108 | 0.655457 | 6.565960 |
| C | -1.851505 | 1.805413 | 6.494084 |
| H | -2.001407 | 2.880814 | 6.431805 |
| C | -1.638419 | 0.854485 | 2.656766 |
| H | -2.468262 | 1.156431 | 3.292689 |
| F | 5.740898 | 2.949611 | 3.108053 |
| C | 2.387114 | 2.761555 | 4.104302 |
| H | 1.992178 | 3.348094 | 3.278474 |
| C | -0.839886 | -0.215631 | 5.748382 |
| H | -0.174390 | -0.751798 | 5.075269 |
| C | -1.252442 | 4.039543 | 3.235439 |
| H | -1.501417 | 3.394546 | 2.395966 |
| F | -4.516986 | 2.418949 | 7.650238 |
| C | -2.567559 | 1.087673 | 7.456968 |
| C | -0.877147 | -0.245451 | 0.638621 |
| H | -1.086891 | -0.775366 | -0.282851 |
| C | -0.647708 | 5.751654 | 5.316929 |
| C | 3.432914 | 1.325209 | 6.215011 |
| F | -0.223169 | 6.051869 | 7.631344 |
| C | -1.407199 | -2.422518 | 6.790027 |
| C | -1.636115 | 5.382487 | 3.179903 |
| C | -1.910600 | 0.181002 | 1.470853 |
| C | 1.583429 | -0.451039 | 0.202129 |
| F | 5.194361 | 4.688530 | 4.291811 |
| C | -2.421645 | -0.285460 | 7.580289 |
| H | -2.971090 | -0.842069 | 8.330466 |
| C | 3.949903 | 0.543332 | 7.385523 |
| C | 4.289667 | 2.093644 | 5.444717 |
| H | 5.351447 | 2.137925 | 5.660957 |
| F | -3.468595 | -1.116652 | 0.234251 |
| C | -1.341665 | 6.254388 | 4.217579 |
| H | -1.644914 | 7.293898 | 4.178414 |
| B | -0.086557 | 1.952956 | 4.470256 |
| C | 3.746938 | 2.814464 | 4.379554 |
| F | 0.961790 | 7.228840 | 6.238354 |
| F | -2.880229 | 2.844176 | 9.014755 |
| F | -4.085410 | 1.050445 | 9.288848 |
| C | -3.507264 | 1.839918 | 8.351340 |
| C | -0.267566 | 6.678303 | 6.433555 |

| | | | |
|---|---|---|---|
| C | -2.359058 | 5.867375 | 1.958657 |
| C | -3.334041 | -0.043994 | 1.054866 |
| C | 4.674959 | 3.663026 | 3.562452 |
| F | -2.815405 | 7.136950 | 2.088850 |
| F | -3.430889 | 5.091203 | 1.661849 |
| F | -1.559875 | 5.850989 | 0.859034 |

Calculated energies and coordinates of $CO_2$

Electronic energy ... -188.57006287 Eh

Total Enthalpy ... -188.55480755 Eh

Final Gibbs free energy ... -188.57907474 Eh

CARTESIAN COORDINATES (ANGSTROEM)

| | | | |
|---|---|---|---|
| C | -3.264744 | -2.136338 | -0.259115 |
| O | -4.428170 | -2.136338 | -0.259115 |
| O | -2.101318 | -2.136338 | -0.259115 |

Calculated energies and coordinates of **M**

Electronic energy ... -1911.69641591 Eh

Total Enthalpy ... -1910.85136300 Eh

Final Gibbs free energy ... -1910.97358793 Eh

CARTESIAN COORDINATES (ANGSTROEM)

| | | | |
|---|---|---|---|
| Sn | 1.543211 | 10.468882 | 3.114231 |
| N | -0.861915 | 10.094645 | 5.328656 |
| N | 2.498200 | 7.497353 | 3.449037 |
| N | 2.709940 | 8.455998 | 5.520237 |
| N | -1.416580 | 9.960257 | 3.086467 |
| C | -0.557349 | 10.733572 | 4.026461 |
| C | -0.746674 | 11.007259 | 6.446001 |
| H | -0.848506 | 10.471812 | 7.397806 |
| H | -1.495807 | 11.824670 | 6.404200 |
| H | 0.243941 | 11.469559 | 6.421097 |
| C | 1.884838 | 8.543233 | 4.301141 |
| C | 3.050445 | 6.554756 | 4.351160 |
| C | 2.078893 | 9.008161 | 6.696754 |
| H | 1.907267 | 10.077883 | 6.533914 |
| H | 2.734950 | 8.897625 | 7.568016 |
| H | 1.097859 | 8.541106 | 6.917976 |
| C | 2.941988 | 12.090720 | 3.912440 |
| C | 4.341779 | 11.872344 | 3.873003 |
| C | -2.103492 | 9.428061 | 5.175357 |
| C | 2.535605 | 13.437787 | 4.079961 |
| C | 1.098223 | 13.843848 | 4.064396 |
| C | 3.168490 | 7.113650 | 5.572368 |
| C | 0.431705 | 14.047587 | 2.843036 |
| C | 0.430356 | 14.108070 | 5.269280 |
| C | 1.588694 | 6.993608 | 2.437262 |
| H | 1.280457 | 7.825655 | 1.793162 |
| H | 0.672278 | 6.548322 | 2.879921 |
| H | 2.086700 | 6.241544 | 1.815092 |
| C | 5.255876 | 12.916855 | 4.047040 |
| H | 6.320878 | 12.690784 | 4.012770 |
| C | -2.433099 | 9.359757 | 3.871360 |
| C | -0.906554 | 14.439799 | 2.854348 |
| H | -1.423890 | 14.571818 | 1.904955 |
| C | 1.152053 | 13.991958 | 6.586689 |
| H | 1.857856 | 13.155860 | 6.580630 |
| H | 0.443772 | 13.859924 | 7.411136 |
| H | 1.742960 | 14.894523 | 6.788363 |
| C | 4.940447 | 10.524856 | 3.620899 |
| C | 4.824227 | 14.222768 | 4.243204 |
| H | 5.539747 | 15.031591 | 4.370514 |
| C | 5.404510 | 9.758474 | 4.701385 |
| C | -1.598377 | 14.657235 | 4.044534 |
| C | -1.826042 | 10.742254 | 1.936083 |
| H | -2.410436 | 11.642173 | 2.226626 |
| H | -2.422478 | 10.132246 | 1.248128 |
| H | -0.926132 | 11.075656 | 1.403921 |
| C | -0.907873 | 14.504161 | 5.243000 |
| H | -1.426377 | 14.679665 | 6.184975 |
| C | 6.023913 | 8.534108 | 4.457048 |
| H | 6.363645 | 7.934364 | 5.300734 |
| C | 5.288080 | 10.280346 | 6.107593 |
| H | 6.035449 | 11.062641 | 6.297721 |
| H | 5.442353 | 9.477456 | 6.834384 |
| H | 4.305848 | 10.725599 | 6.282253 |
| C | 3.458867 | 14.475209 | 4.251661 |
| H | 3.089441 | 15.491917 | 4.381533 |
| C | 5.142656 | 10.071835 | 2.307111 |
| C | 1.151282 | 13.862129 | 1.535486 |
| H | 2.091063 | 14.425346 | 1.521928 |
| H | 0.526966 | 14.190272 | 0.698704 |
| H | 1.412114 | 12.805131 | 1.380855 |
| C | 3.586309 | 5.266997 | 3.836496 |
| H | 2.790540 | 4.586111 | 3.504297 |
| H | 4.170297 | 4.746736 | 4.600986 |
| H | 4.239635 | 5.447853 | 2.970346 |
| C | 4.724052 | 10.908035 | 1.128933 |
| H | 3.627913 | 10.971154 | 1.072831 |
| H | 5.093266 | 10.473606 | 0.194567 |
| H | 5.102783 | 11.932605 | 1.216822 |
| C | -3.513165 | 8.582704 | 3.204651 |
| H | -4.210663 | 9.227611 | 2.653350 |
| H | -4.096258 | 8.015023 | 3.934819 |
| H | -3.085769 | 7.873824 | 2.480063 |
| C | 6.198365 | 8.052190 | 3.163499 |
| C | -2.717527 | 8.746684 | 6.347559 |
| H | -1.976466 | 8.107216 | 6.849604 |
| H | -3.558016 | 8.118207 | 6.041111 |
| H | -3.091415 | 9.458375 | 7.096052 |
| C | 3.867314 | 6.599229 | 6.780088 |
| H | 4.669740 | 7.281593 | 7.097461 |
| H | 4.317010 | 5.622480 | 6.580387 |
| H | 3.189714 | 6.482417 | 7.637152 |
| C | 5.752823 | 8.833544 | 2.101155 |
| H | 5.885203 | 8.475226 | 1.081074 |
| C | -3.068765 | 14.975254 | 4.034749 |
| H | -3.358011 | 15.504458 | 3.120886 |
| H | -3.353278 | 15.590194 | 4.895106 |
| H | -3.658618 | 14.049927 | 4.080280 |
| C | 6.890181 | 6.735798 | 2.922299 |
| H | 7.980993 | 6.858721 | 2.888207 |
| H | 6.579244 | 6.292850 | 1.970510 |
| H | 6.664221 | 6.021146 | 3.720693 |
| H | 0.843013 | 8.207319 | 4.564413 |
| H | -0.921865 | 11.789807 | 4.053993 |

## 5. References


(S1) 1) Kuhn, N.; Kratz, T. Synthesis of imidazol-2-ylidenes by reduction of imidazole-2 (3H)-thiones. Synthesis 1993, 1993(06), 561-562; 2) Schulz, Axel, Max Thomas, and Alexander Villinger. "Tetrazastannoles versus distannadiazanes–a question of the tin(II) source." *Dalton Transactions* 48.1 (2019): 125-132; 3) Martínez-Martínez, Antonio J., and Andrew S. Weller. "Solvent-free anhydrous $Li^+$, $Na^+$ and $K^+$ salts of $[B(3,5-(CF_3)_2C_6H_3)_4]^-$, $[BArF_4]^-$. Improved synthesis and solid-state structures." Dalton Transactions 48.11 (2019): 3551-3554; 4)

Krossing I. The facile preparation of weakly coordinating anions: structure and characterisation of silverpolyfluoroalkoxyaluminates $AgAl(ORF)_4$, calculation of the alkoxide ion affinity. I. Krossing, *Chem.-Eur. J.*, 2001, **7**, 490-502.

(S2) Schulz, A.; Thomas, M.; Villinger, A. Tetrazastannoles versus distannadiazanes - a question of the tin(ii) source. Dalton Trans 2018, 48(1), 125-132. DOI: 10.1039/c8dt04295k From NLM PubMed-not-MEDLINE.

(S3) Brookhart, M.; Grant, B.; Volpe, A. F. $[(3,5-(CF_3)_2C_6H_3)_4B]-[H(OEt_2)_2]^+$: a convenient reagent for generation and stabilization of cationic, highly electrophilic organometallic complexes. Organometallics 2002, 11(11), 3920-3922. DOI: 10.1021/om00059a071.

(S4) APEX suite of crystallographic software, APEX 4 version 2021.10-0; Bruker AXS Inc.: Madison, Wisconsin, USA, 2021.

(S5) CrysAlisPRO, Oxford Diffraction /Agilent Technologies UK Ltd, Yarnton, England.

(S6) SAINT, Version 7.56a and SADABS Version 2008/1; Bruker AXS Inc.: Madison, Wisconsin, USA, 2008.

(S7) Sheldrick, G. M. SHELXL-2014, University of Göttingen, Göttingen, Germany, 2014.

(S8) Hübschle, C. B.; Sheldrick, G. M.; Dittrich, B. J. Appl. Cryst. 2011, 44, 1281-1284.

(S9) Sheldrick, G. M. SHELXL-97, University of Göttingen, Göttingen, Germany, 1998.

(S10) Wilson, A. J. C. International Tables for Crystallography, Vol. C, Tables 6.1.1.4 (pp. 500-502), 4.2.6.8 (pp. 219-222), and 4.2.4.2 (pp. 193-199); Kluwer Academic Publishers: Dordrecht, The Netherlands, 1992.

(S11) Macrae, C. F.; Bruno, I. J.; Chisholm, J. A.; Edgington, P. R.; McCabe, P.; Pidcock, E.; Rodriguez-Monge, L.; Taylor, R.; van de Streek, J.; Wood, P. A. J. Appl. Cryst. 2008, 41, 466-470.

(S12) Neese, F. WIREs Comput Mol Sci. 2022, 12, e1606.

(S13) Furness, J. W.; Kaplan, A. D.; Ning, J.; Perdew, J. P.; Sun, J. J. Phys. Chem. Lett. 2020, 11, 8208-8215.

(S14) Furness, J. W.; Kaplan, A. D.; Ning, J.; Perdew, J. P.; Sun, J. J. Phys. Chem. Lett. 2020, 11, 9248.

(S15) Kruse, H.; Grimme, S. J. Chem. Phys. 2012, 136, 154101.

(S16) Caldeweyher, E.; Mewes, J.-M.; Ehlert, S.; Grimme, S. Phys. Chem. Chem. Phys. 2020, 22, 8499-8512.

(S17) Caldeweyher, E.; Ehlert, S.; Hansen, A.; Neugebauer, H.; Spicher, S.; Bannwarth, C.; Grimme, S. J. Chem. Phys. 2019, 150, 154122.

(S18) Caldeweyher, E.; Bannwarth, C.; Grimme, S. J. Chem. Phys. 2017, 147, 034112.

(S19) Grimme, S.; Hansen, A.; Ehlert, S.; Mewes, J.-M. J. Chem. Phys. 2021, 154, 064103.

(S20) B. Metz, H. Stoll, M. Dolg, J. Chem. Phys., 2000, 113, 2563-2569.

(S21) Marenich, A. V.; Cramer, C. J.; Truhlar, D. G. J. Phys. Chem. B 2009, 113, 6378-6396.

(S22) Y. Zhao, D. G. Truhlar, J. Phys. Chem. A 2005, 109, 5656-5667.

(S23) F. Weigend and R. Ahlrichs, Phys. Chem. Chem. Phys. 7, 3297 (2005).

(S24) F. Weigend, Phys. Chem. Chem. Phys. 8, 1057 (2006).

(S25) A. Hellweg, C. Hattig, S. Hofener and W. Klopper, Theor. Chem. Acc. 117, 587 (2007).

(S26) NBO 7.0. E. D. Glendening, J, K. Badenhoop, A. E. Reed, J. E. Carpenter, J. A. Bohmann, C. M. Morales, P. Karafiloglou, C. R. Landis, and F. Weinhold, Theoretical Chemistry Institute, University of Wisconsin, Madison (2018).

(S27) C. Adamo, V. Barone, The Journal of Chemical Physics 1999, 110, 6158-6170.

(S28) F. Weigend and R. Ahlrichs, Phys. Chem. Chem. Phys. 7, 3297 (2005).
